# Supplementary figures and images for: The critical role of the proto-oncogene c-Kit in TSC renal cystogenesis (part 1 of 2)
Source: EMBO Mol Med. 2025 Dec 22;18(2):575–98. doi: 10.1038/s44321-025-00360-x (PMC12905254; doi:10.1038/s44321-025-00360-x)

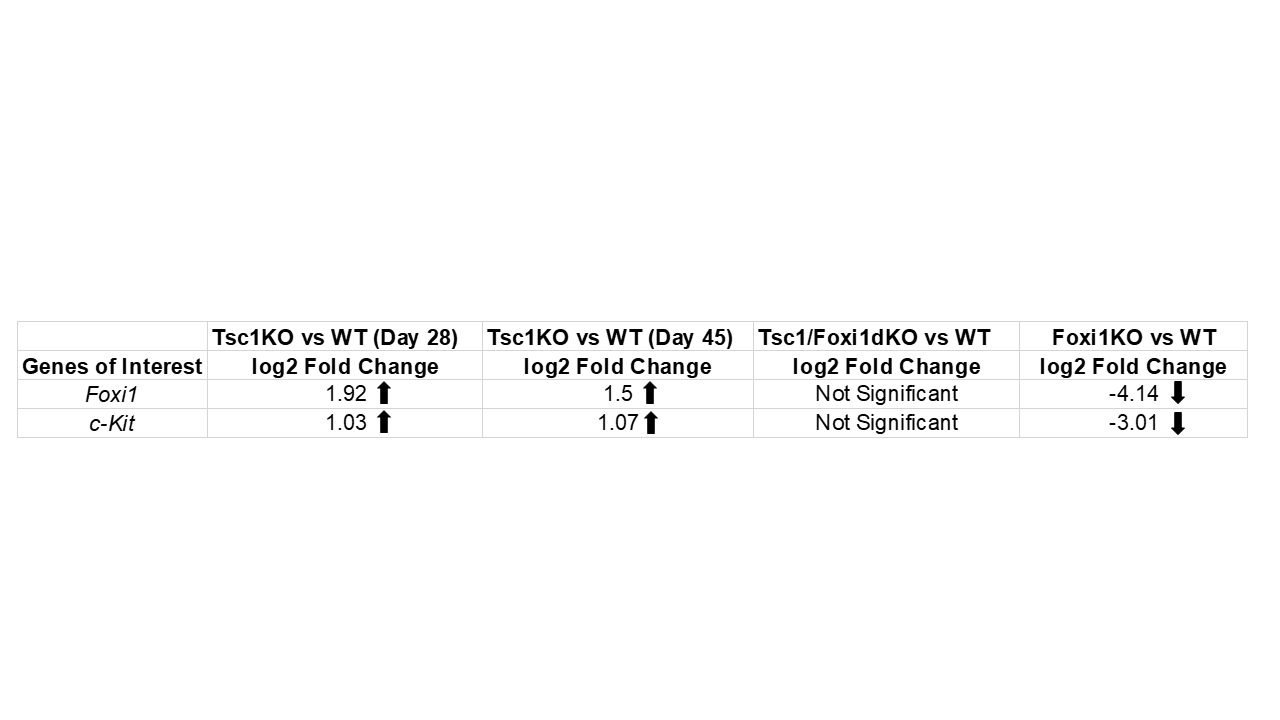

Supplement: Supplementary file 9 — Source data Fig. 1 [file 44321_2025_360_MOESM9_ESM.zip › EMM-2025-22130_SourceDataForFigure1A-F 10-28-25/1A/Figure 1A RNASeq Table.tif]

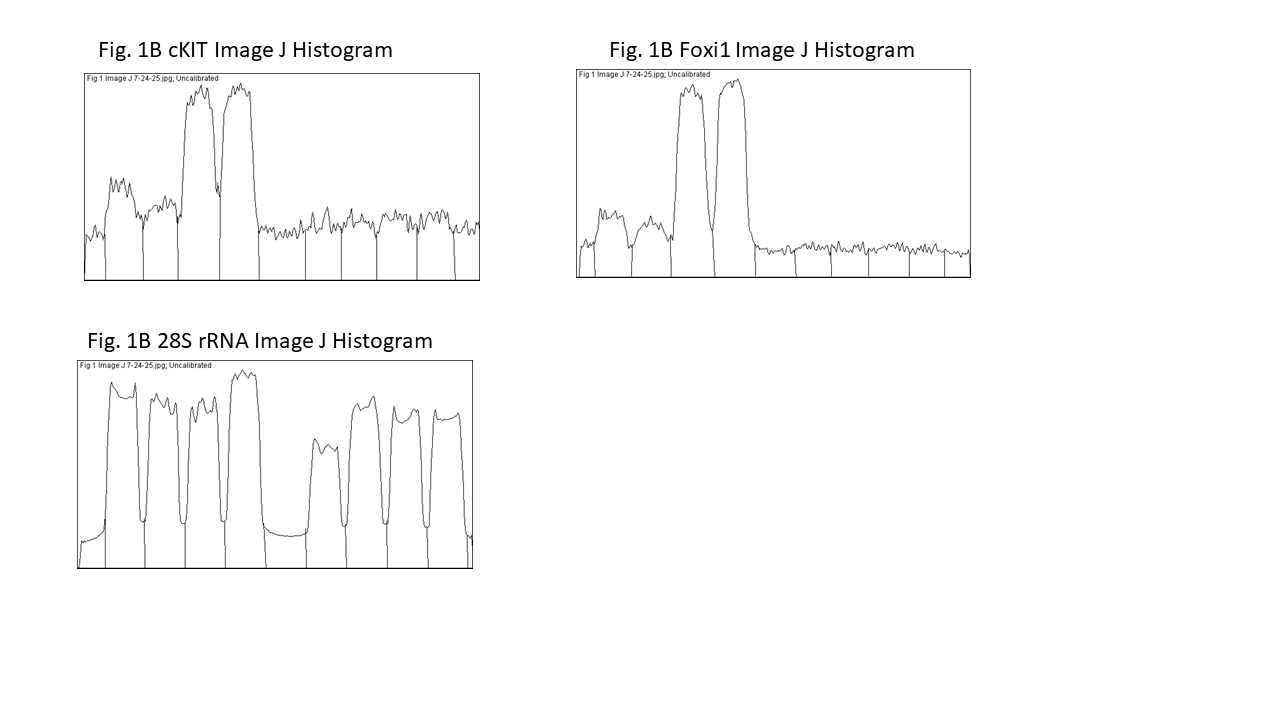

Supplement: Supplementary file 9 — Source data Fig. 1 [file 44321_2025_360_MOESM9_ESM.zip › EMM-2025-22130_SourceDataForFigure1A-F 10-28-25/1B/Figure 1B Image J Histograms.tif]

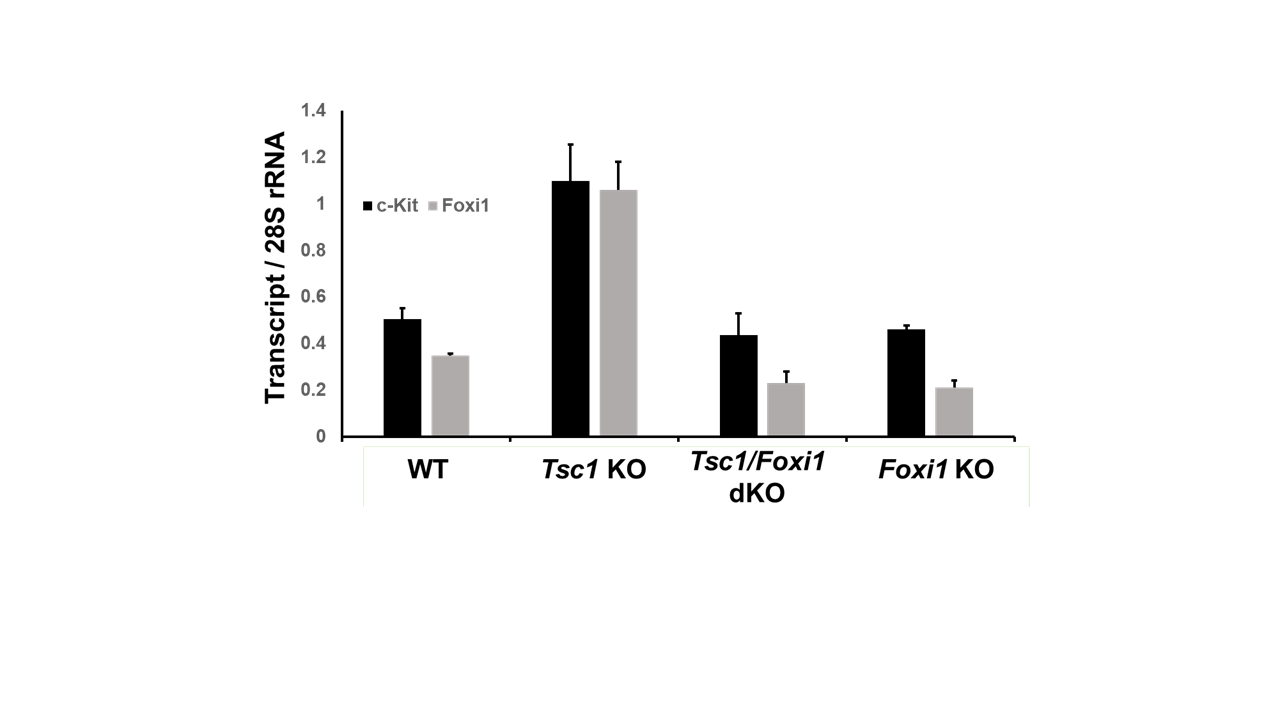

Supplement: Supplementary file 9 — Source data Fig. 1 [file 44321_2025_360_MOESM9_ESM.zip › EMM-2025-22130_SourceDataForFigure1A-F 10-28-25/1B/Figure 1B Northern Blot Quantification.tif]

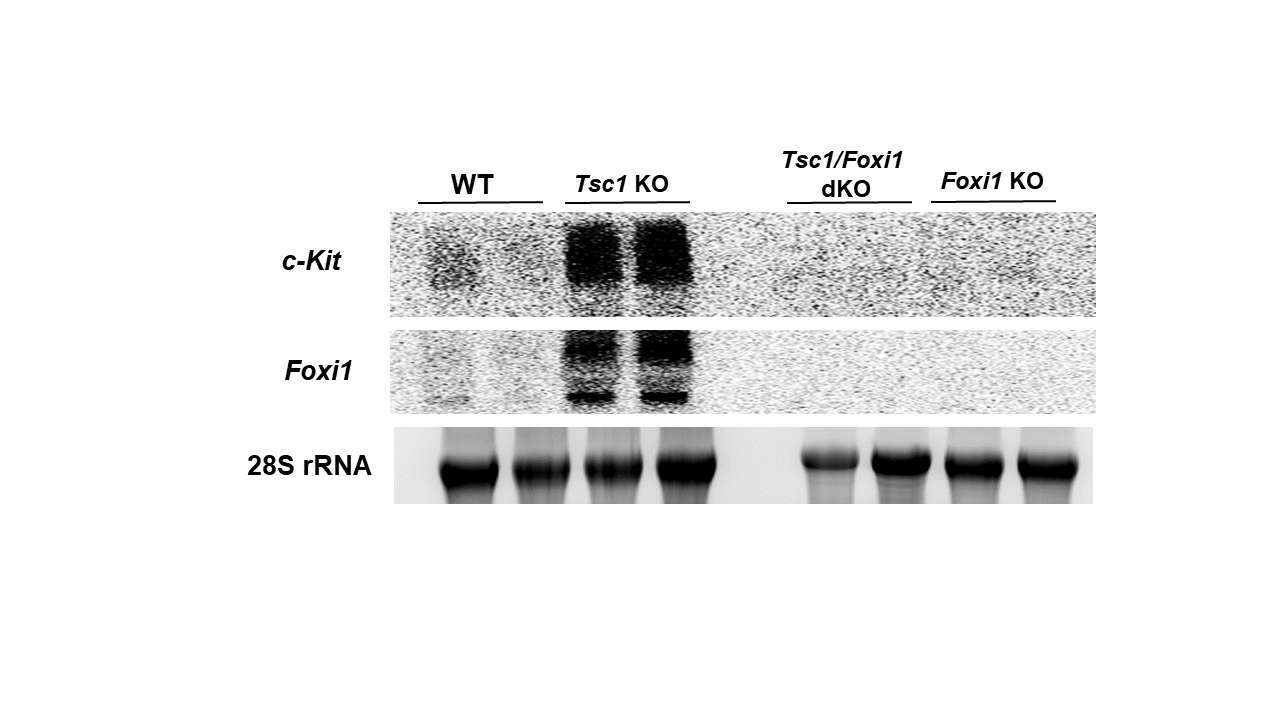

Supplement: Supplementary file 9 — Source data Fig. 1 [file 44321_2025_360_MOESM9_ESM.zip › EMM-2025-22130_SourceDataForFigure1A-F 10-28-25/1B/Figure 1B Northern Blot.tif]

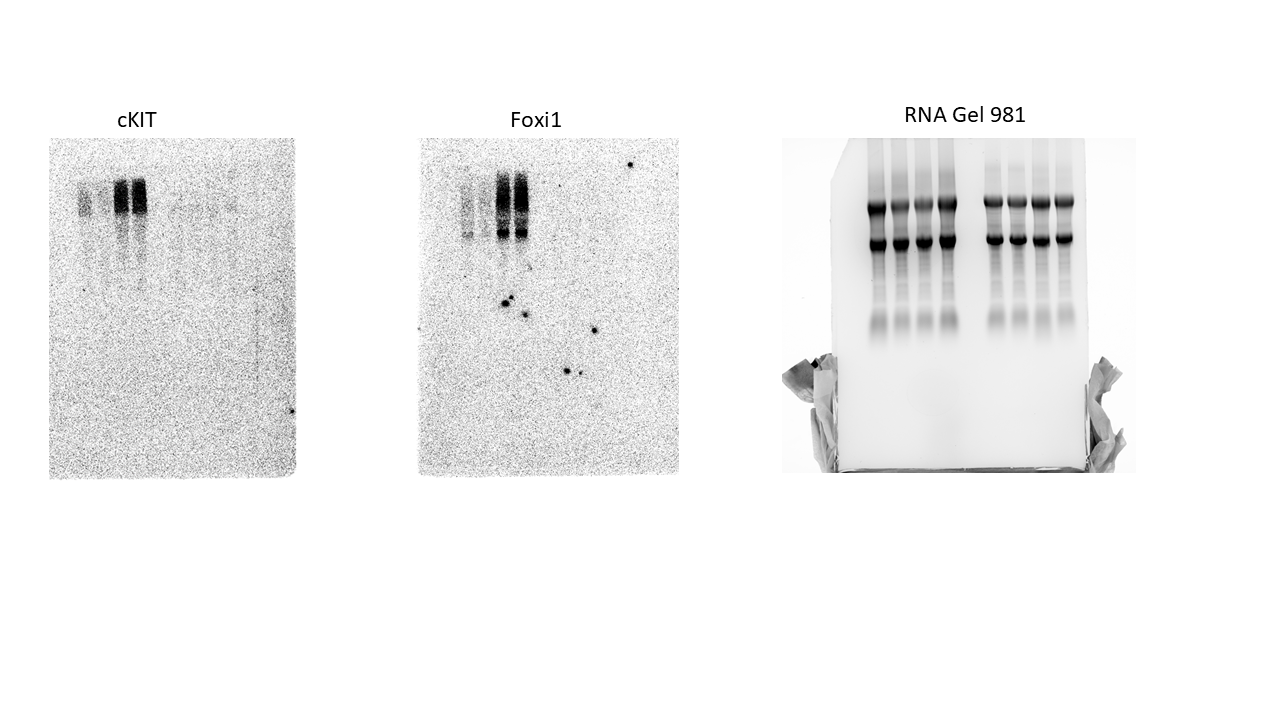

Supplement: Supplementary file 9 — Source data Fig. 1 [file 44321_2025_360_MOESM9_ESM.zip › EMM-2025-22130_SourceDataForFigure1A-F 10-28-25/1B/Figure 1B Source Data Northern Blots.tif]

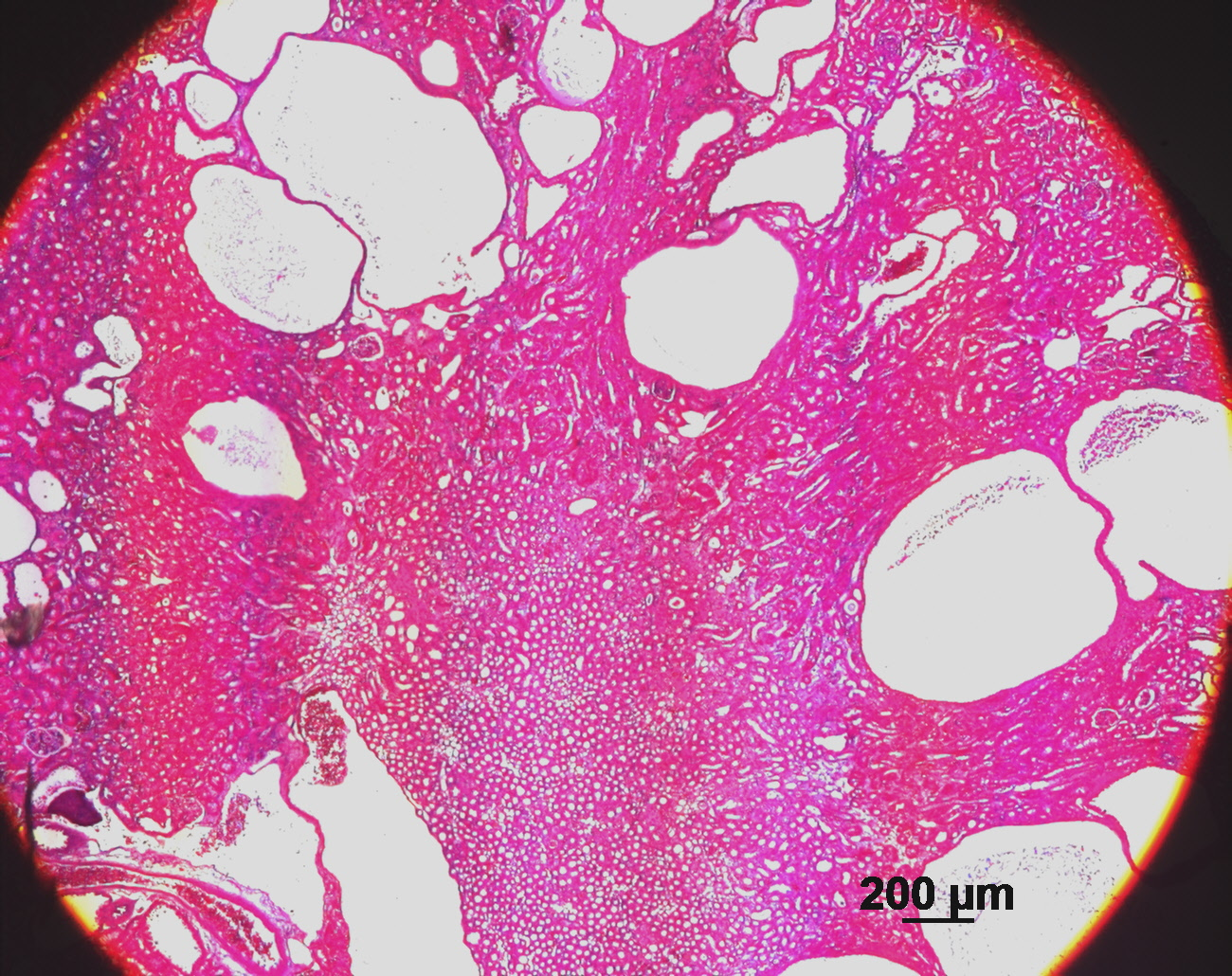

Supplement: Supplementary file 9 — Source data Fig. 1 [file 44321_2025_360_MOESM9_ESM.zip › EMM-2025-22130_SourceDataForFigure1A-F 10-28-25/1C/Tsc1 KO H&E 4X-1.tif]

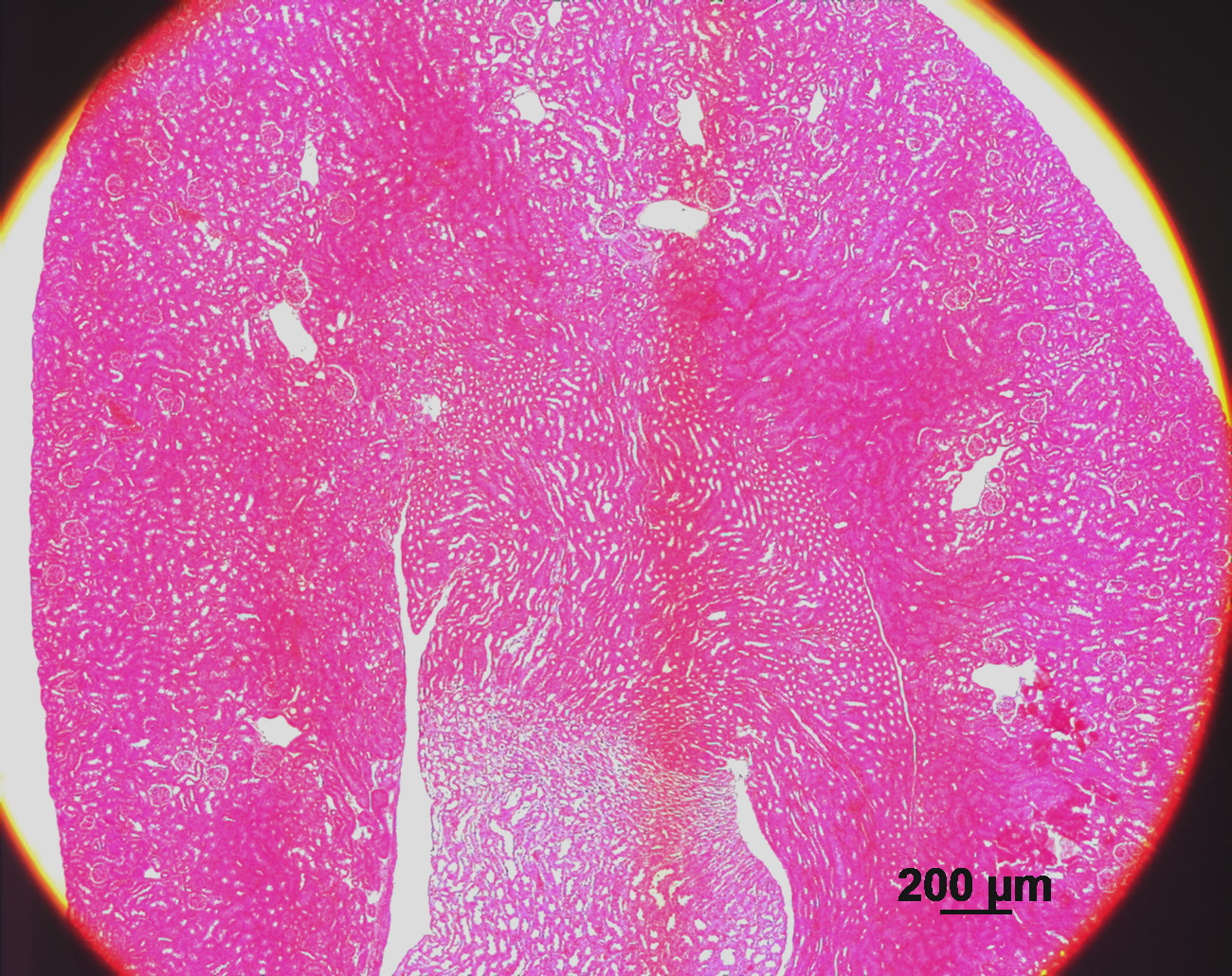

Supplement: Supplementary file 9 — Source data Fig. 1 [file 44321_2025_360_MOESM9_ESM.zip › EMM-2025-22130_SourceDataForFigure1A-F 10-28-25/1C/Tsc1-Foxi dKO H&E 4X-1.tif]

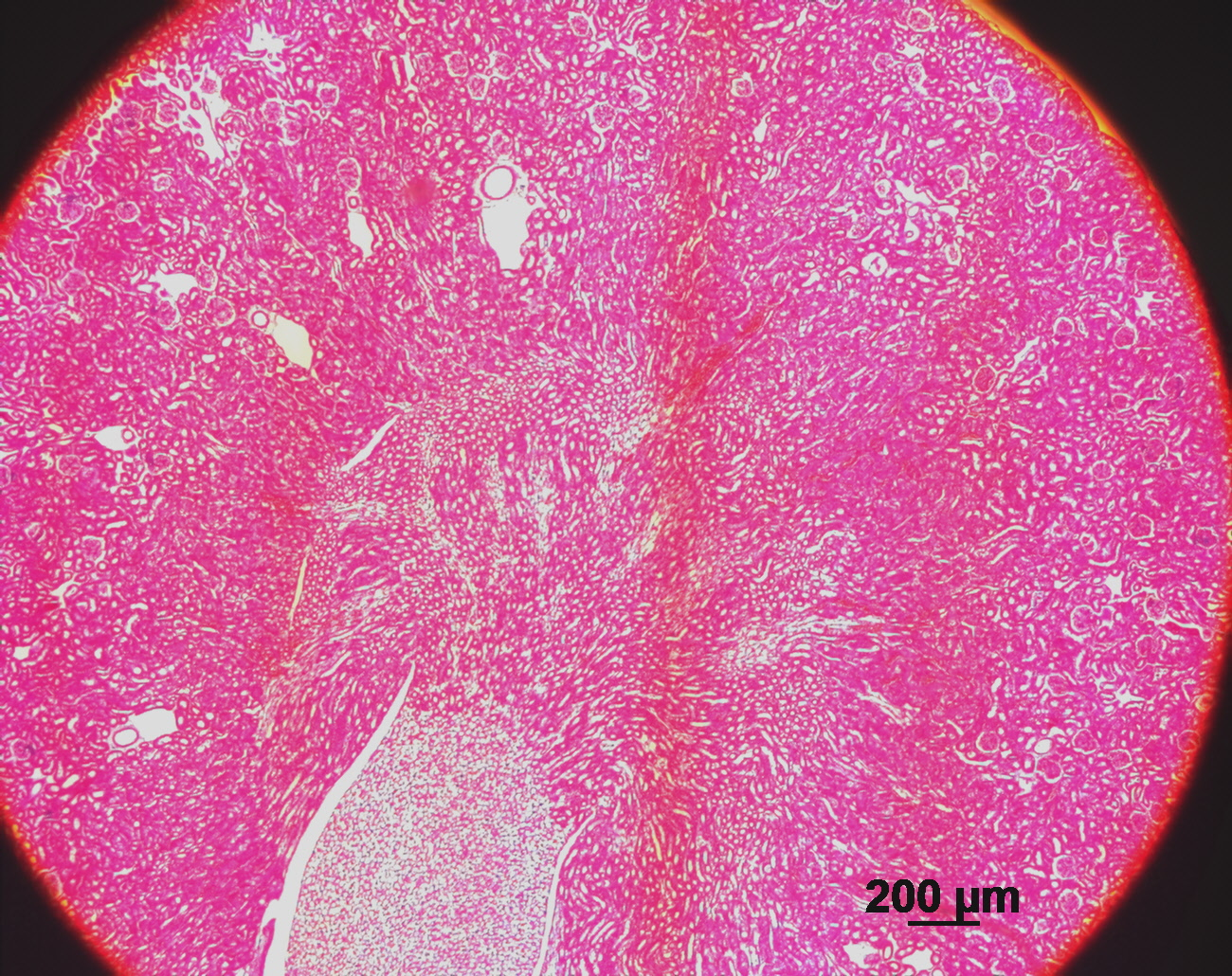

Supplement: Supplementary file 9 — Source data Fig. 1 [file 44321_2025_360_MOESM9_ESM.zip › EMM-2025-22130_SourceDataForFigure1A-F 10-28-25/1C/WT H&E 4X-1.tif]

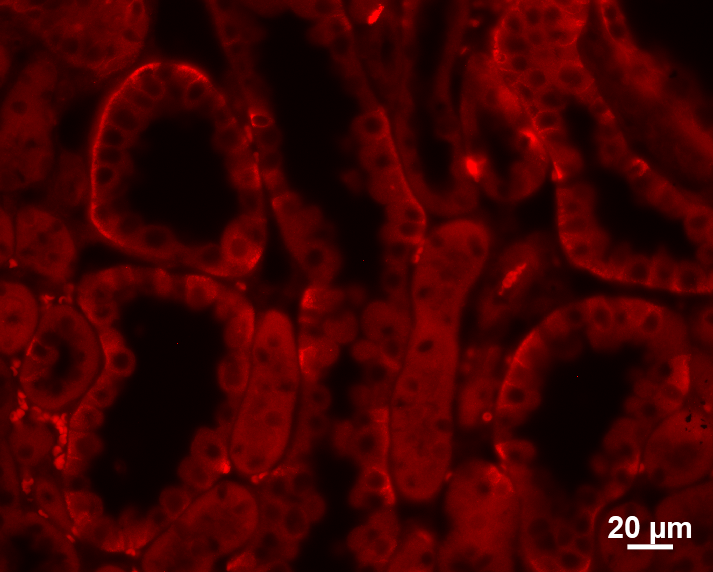

Supplement: Supplementary file 9 — Source data Fig. 1 [file 44321_2025_360_MOESM9_ESM.zip › EMM-2025-22130_SourceDataForFigure1A-F 10-28-25/1D/Tsc1 KO c-KIT 40X.tif]

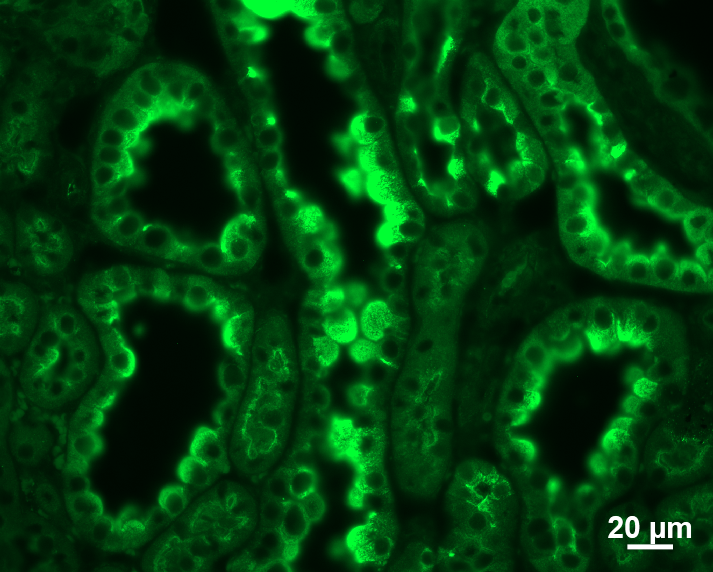

Supplement: Supplementary file 9 — Source data Fig. 1 [file 44321_2025_360_MOESM9_ESM.zip › EMM-2025-22130_SourceDataForFigure1A-F 10-28-25/1D/Tsc1 KO HATPase 40X.tif]

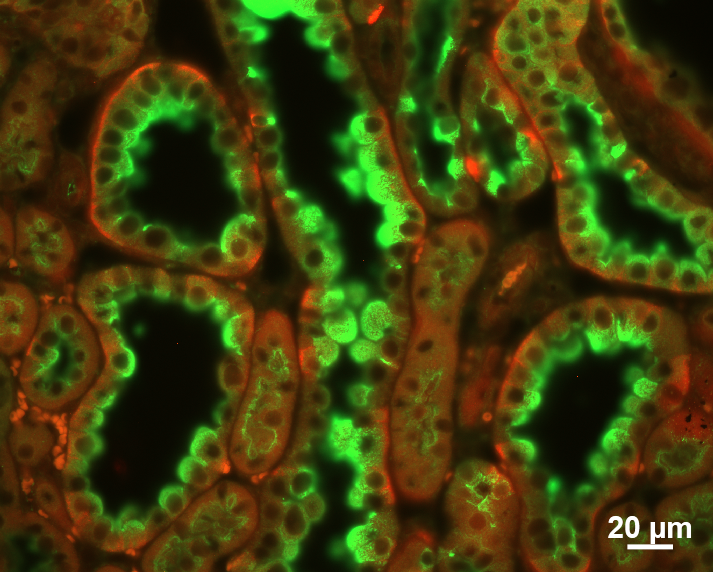

Supplement: Supplementary file 9 — Source data Fig. 1 [file 44321_2025_360_MOESM9_ESM.zip › EMM-2025-22130_SourceDataForFigure1A-F 10-28-25/1D/Tsc1 KO Merged 40X.tif]

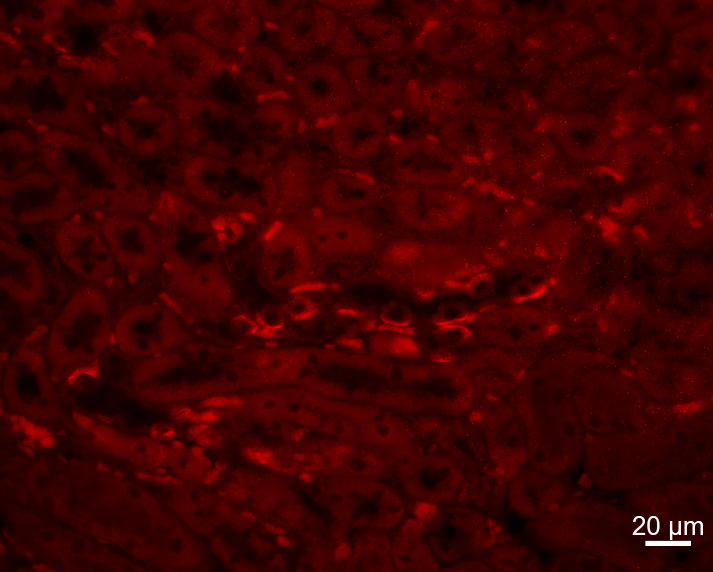

Supplement: Supplementary file 9 — Source data Fig. 1 [file 44321_2025_360_MOESM9_ESM.zip › EMM-2025-22130_SourceDataForFigure1A-F 10-28-25/1D/WT c-KIT 40X.tif]

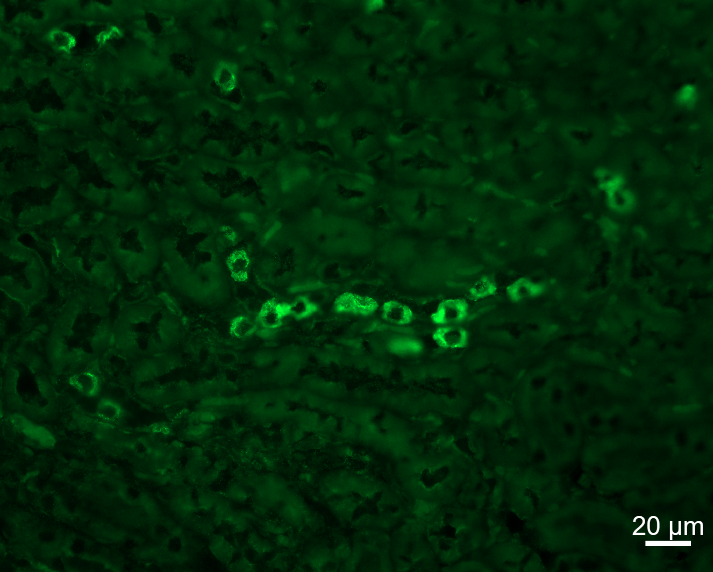

Supplement: Supplementary file 9 — Source data Fig. 1 [file 44321_2025_360_MOESM9_ESM.zip › EMM-2025-22130_SourceDataForFigure1A-F 10-28-25/1D/WT HATPase 40X.tif]

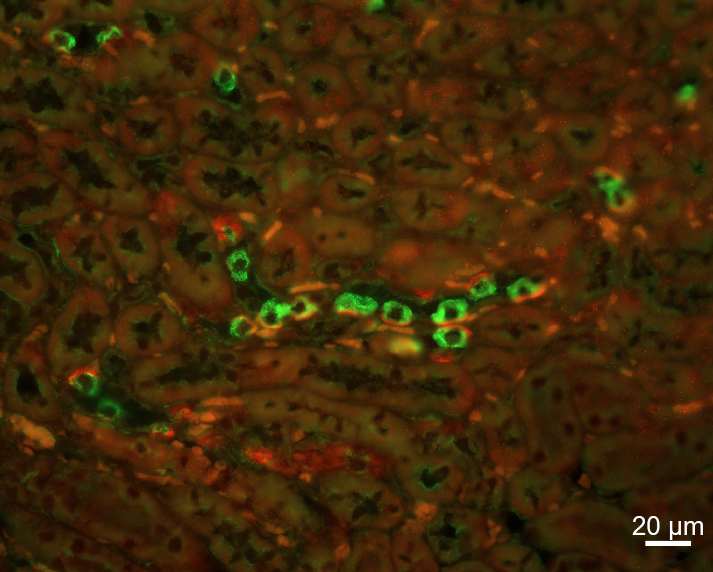

Supplement: Supplementary file 9 — Source data Fig. 1 [file 44321_2025_360_MOESM9_ESM.zip › EMM-2025-22130_SourceDataForFigure1A-F 10-28-25/1D/WT Merged 40X.tif]

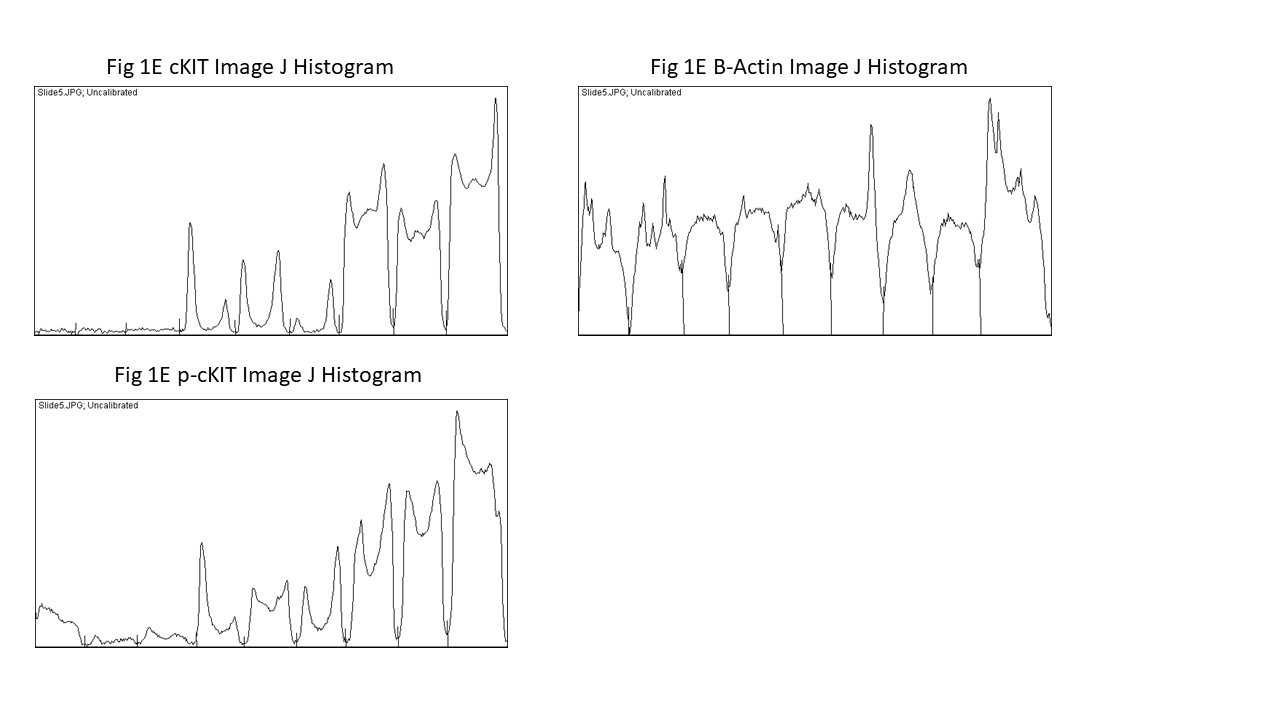

Supplement: Supplementary file 9 — Source data Fig. 1 [file 44321_2025_360_MOESM9_ESM.zip › EMM-2025-22130_SourceDataForFigure1A-F 10-28-25/1E/Western Blot Image J Histograms.tif]

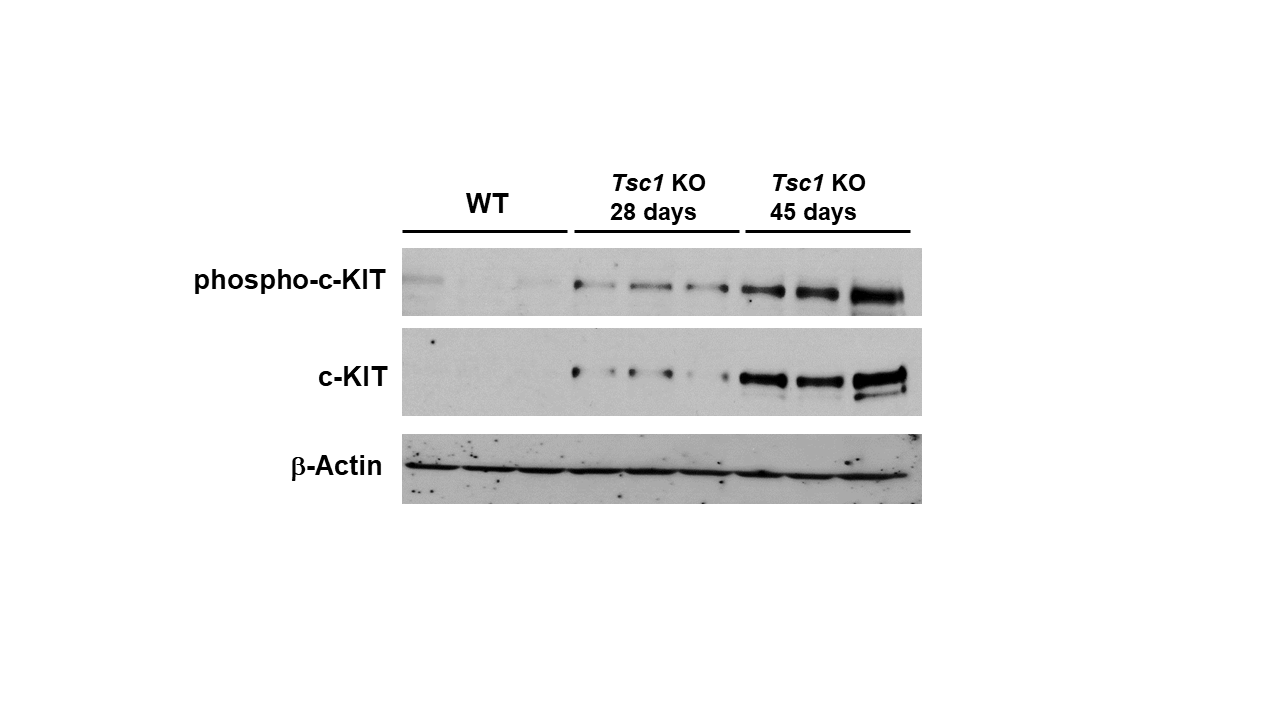

Supplement: Supplementary file 9 — Source data Fig. 1 [file 44321_2025_360_MOESM9_ESM.zip › EMM-2025-22130_SourceDataForFigure1A-F 10-28-25/1E/Western Blot p-cKIT and cKIT.tif]

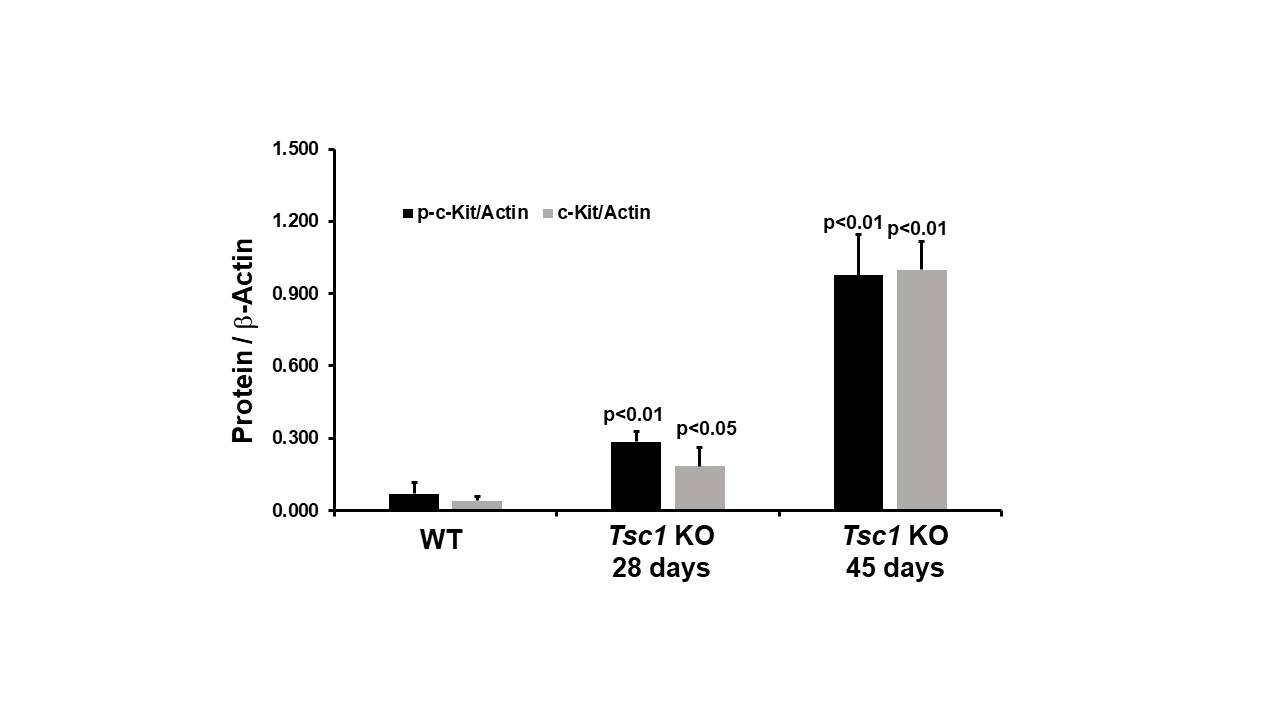

Supplement: Supplementary file 9 — Source data Fig. 1 [file 44321_2025_360_MOESM9_ESM.zip › EMM-2025-22130_SourceDataForFigure1A-F 10-28-25/1E/Western Blot Quantificiation.tif]

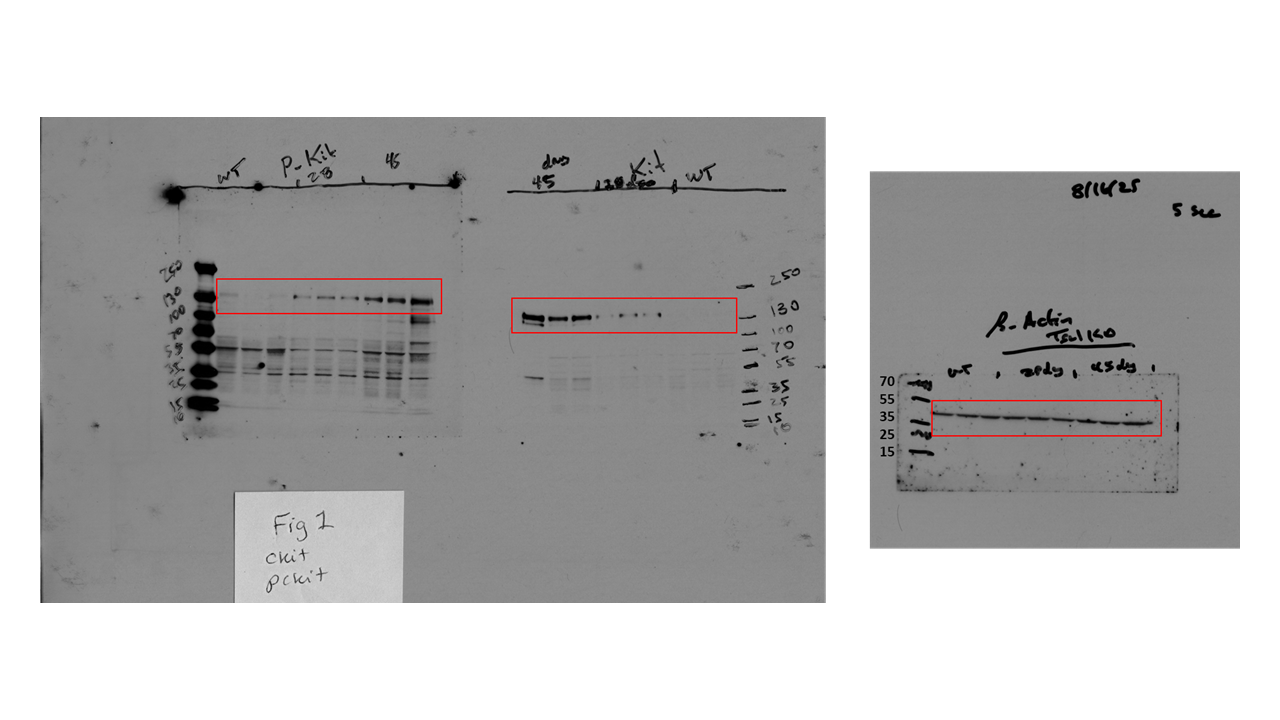

Supplement: Supplementary file 9 — Source data Fig. 1 [file 44321_2025_360_MOESM9_ESM.zip › EMM-2025-22130_SourceDataForFigure1A-F 10-28-25/1E/Western Blot Source Data 10-28-25.tif]

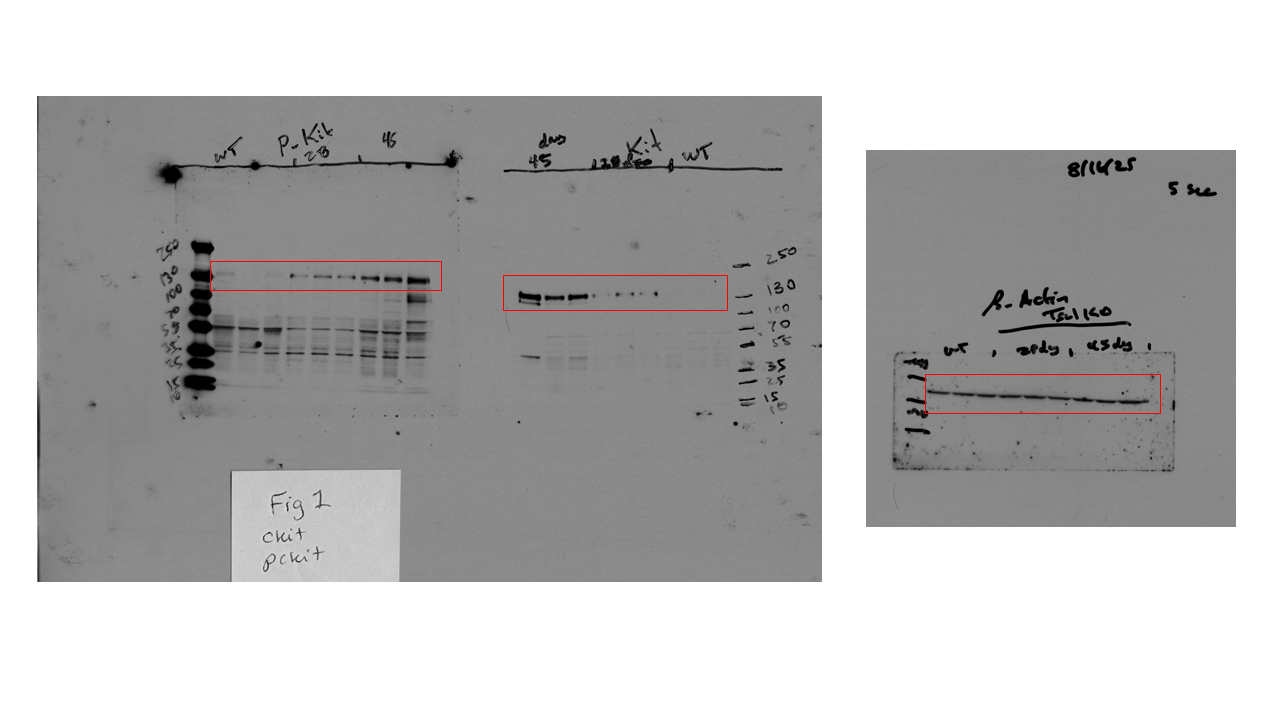

Supplement: Supplementary file 9 — Source data Fig. 1 [file 44321_2025_360_MOESM9_ESM.zip › EMM-2025-22130_SourceDataForFigure1A-F 10-28-25/1E/Western Blot Source Data.tif]

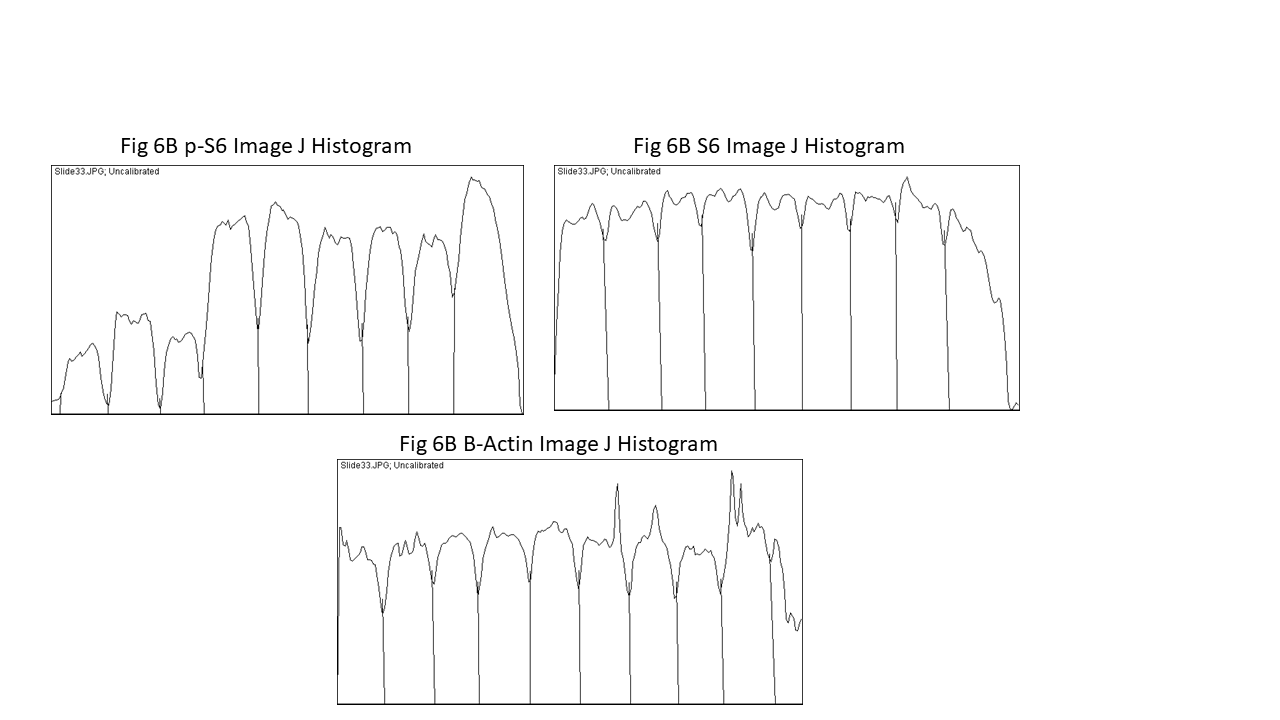

Supplement: Supplementary file 9 — Source data Fig. 1 [file 44321_2025_360_MOESM9_ESM.zip › EMM-2025-22130_SourceDataForFigure1A-F 10-28-25/1F/Western Blot Image J Histograms.tif]

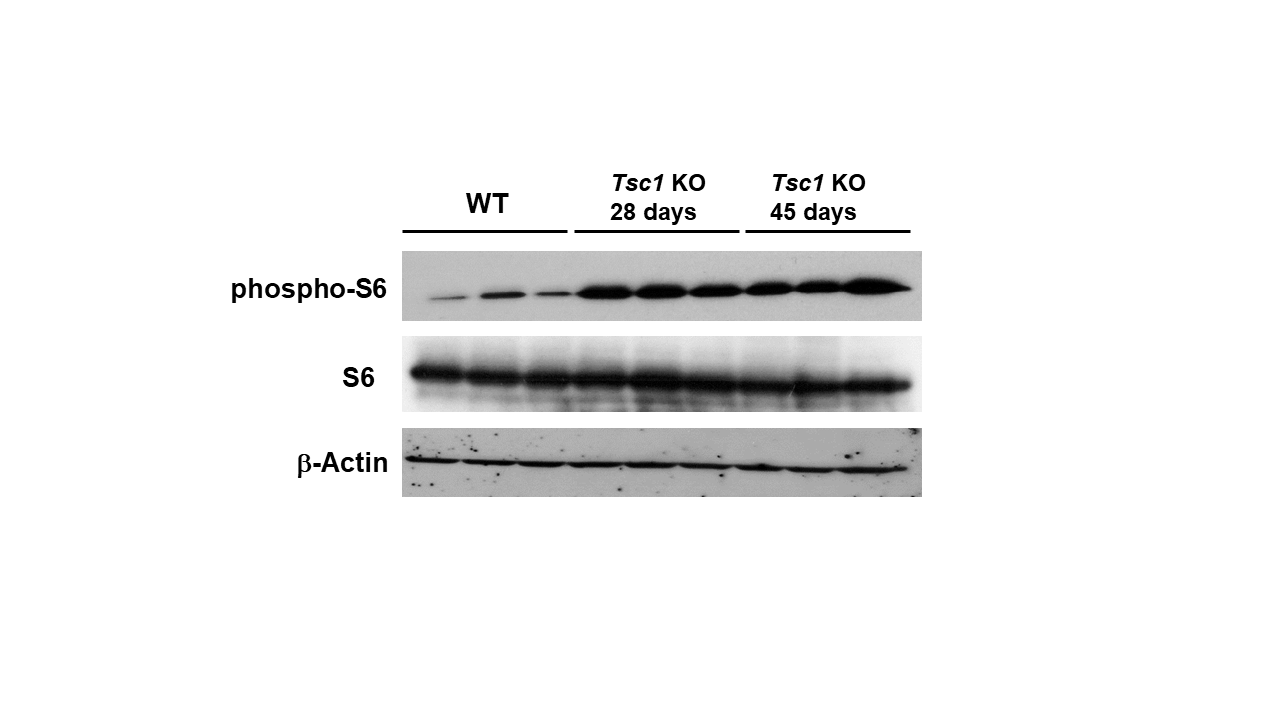

Supplement: Supplementary file 9 — Source data Fig. 1 [file 44321_2025_360_MOESM9_ESM.zip › EMM-2025-22130_SourceDataForFigure1A-F 10-28-25/1F/Western Blot pS6 and S6.tif]

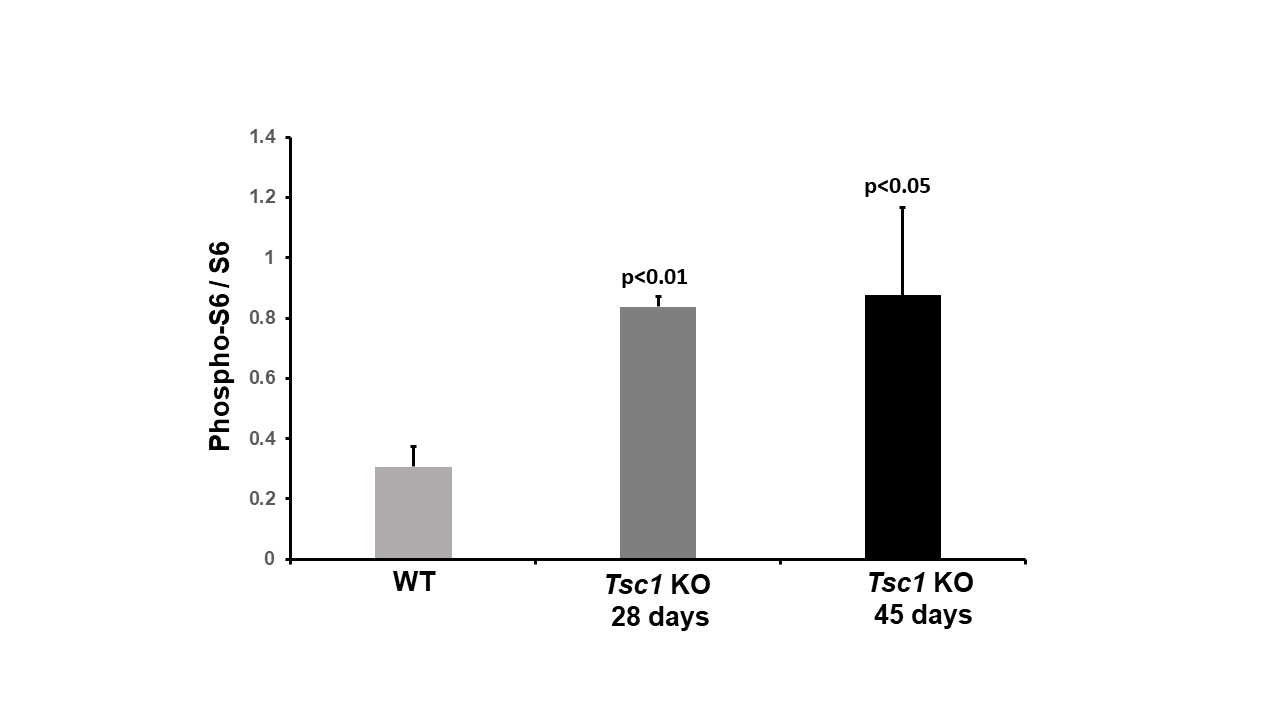

Supplement: Supplementary file 9 — Source data Fig. 1 [file 44321_2025_360_MOESM9_ESM.zip › EMM-2025-22130_SourceDataForFigure1A-F 10-28-25/1F/Western Blot Quantification.tif]

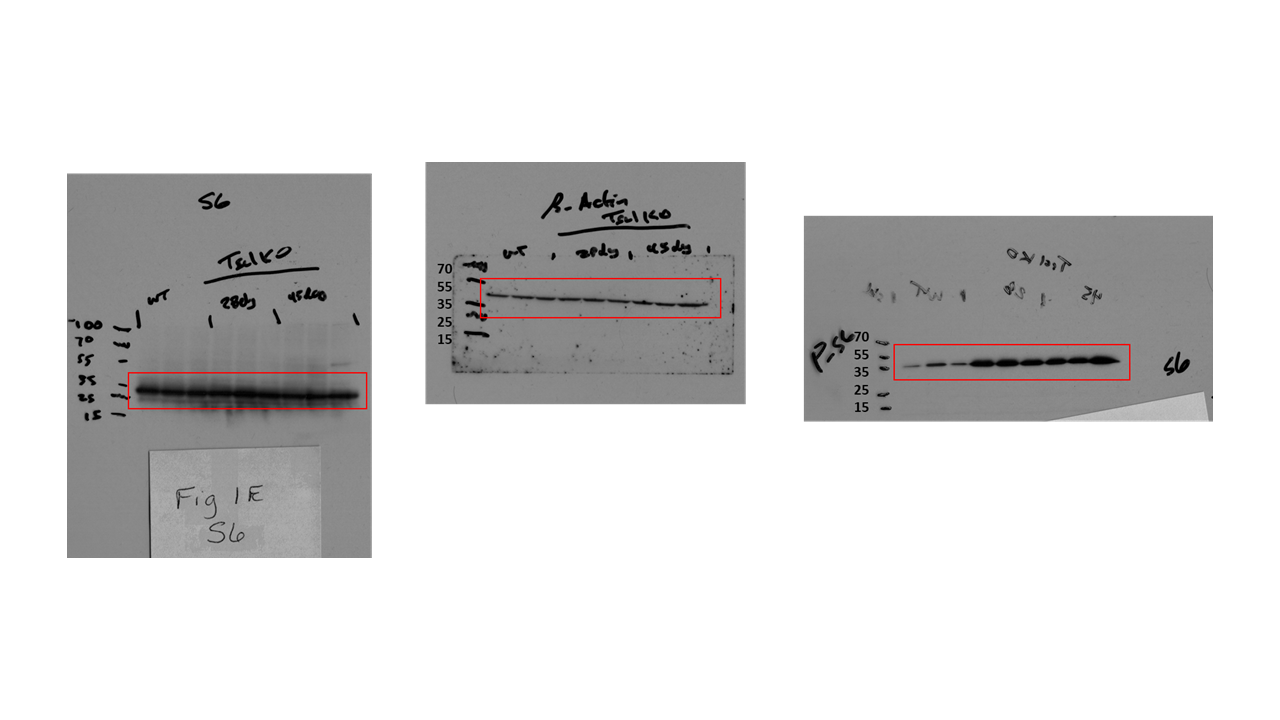

Supplement: Supplementary file 9 — Source data Fig. 1 [file 44321_2025_360_MOESM9_ESM.zip › EMM-2025-22130_SourceDataForFigure1A-F 10-28-25/1F/Western Blot Source Data 10-28-25.tif]

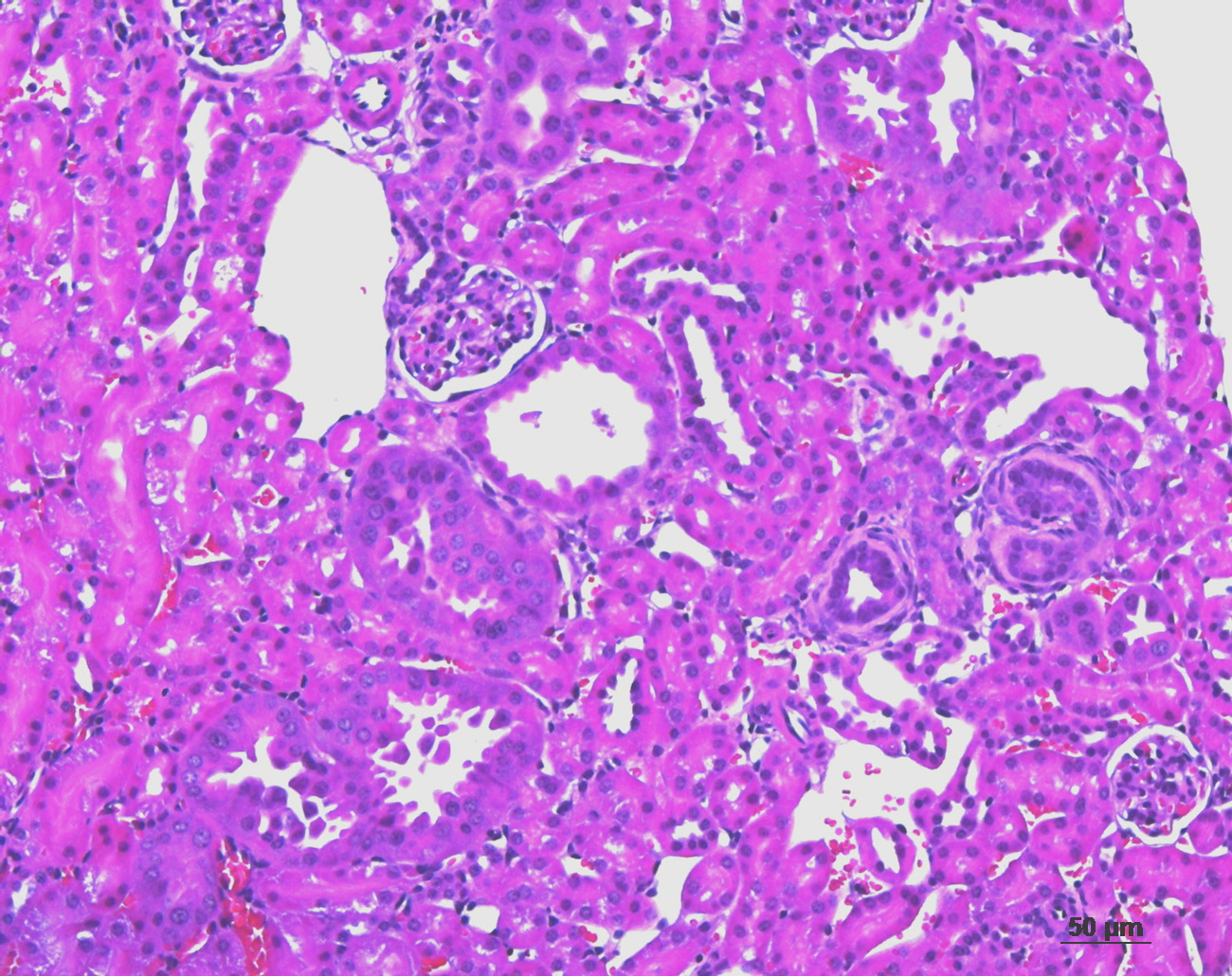

Supplement: Supplementary file 10 — Source data Fig. 2 [file 44321_2025_360_MOESM10_ESM.zip › EMM-2025-22130_SourceDataForFigure 2A-C 10-28-25/2A/TSC1 ECE 140 days H&E 20X.tif]

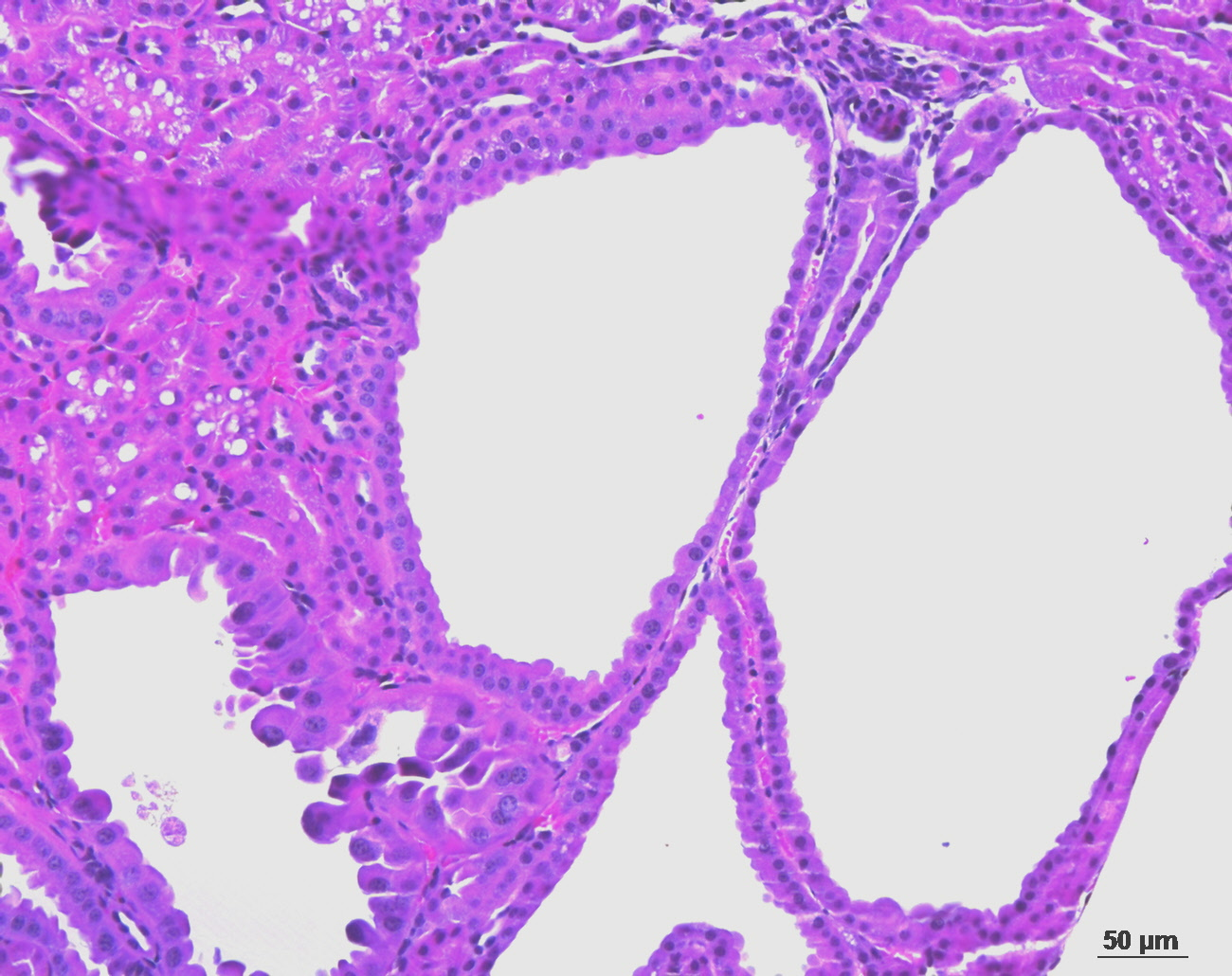

Supplement: Supplementary file 10 — Source data Fig. 2 [file 44321_2025_360_MOESM10_ESM.zip › EMM-2025-22130_SourceDataForFigure 2A-C 10-28-25/2A/TSC1 ECE 230 days H&E 20X.tif]

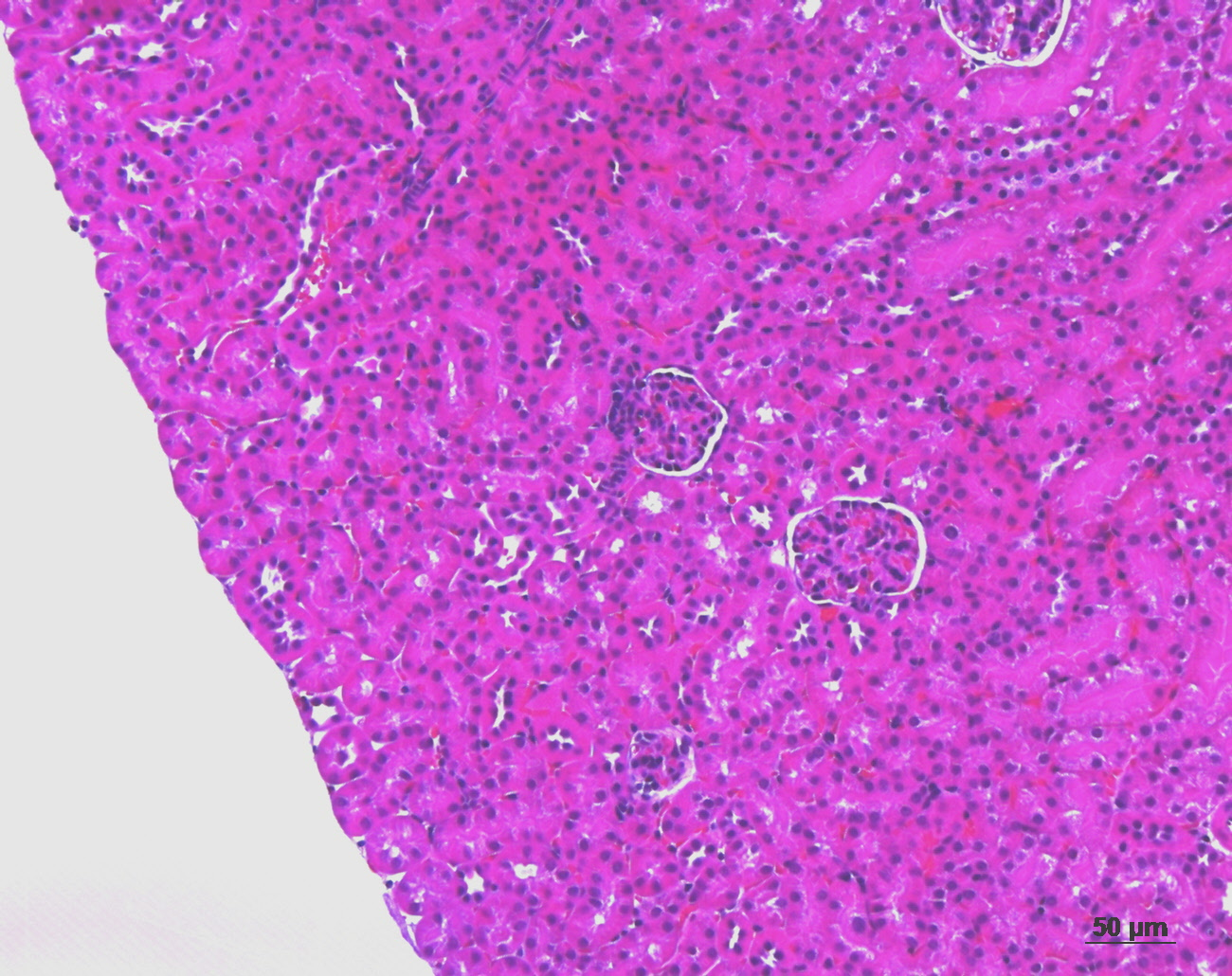

Supplement: Supplementary file 10 — Source data Fig. 2 [file 44321_2025_360_MOESM10_ESM.zip › EMM-2025-22130_SourceDataForFigure 2A-C 10-28-25/2A/TSC1 ECE 30 days H&E 20X.tif]

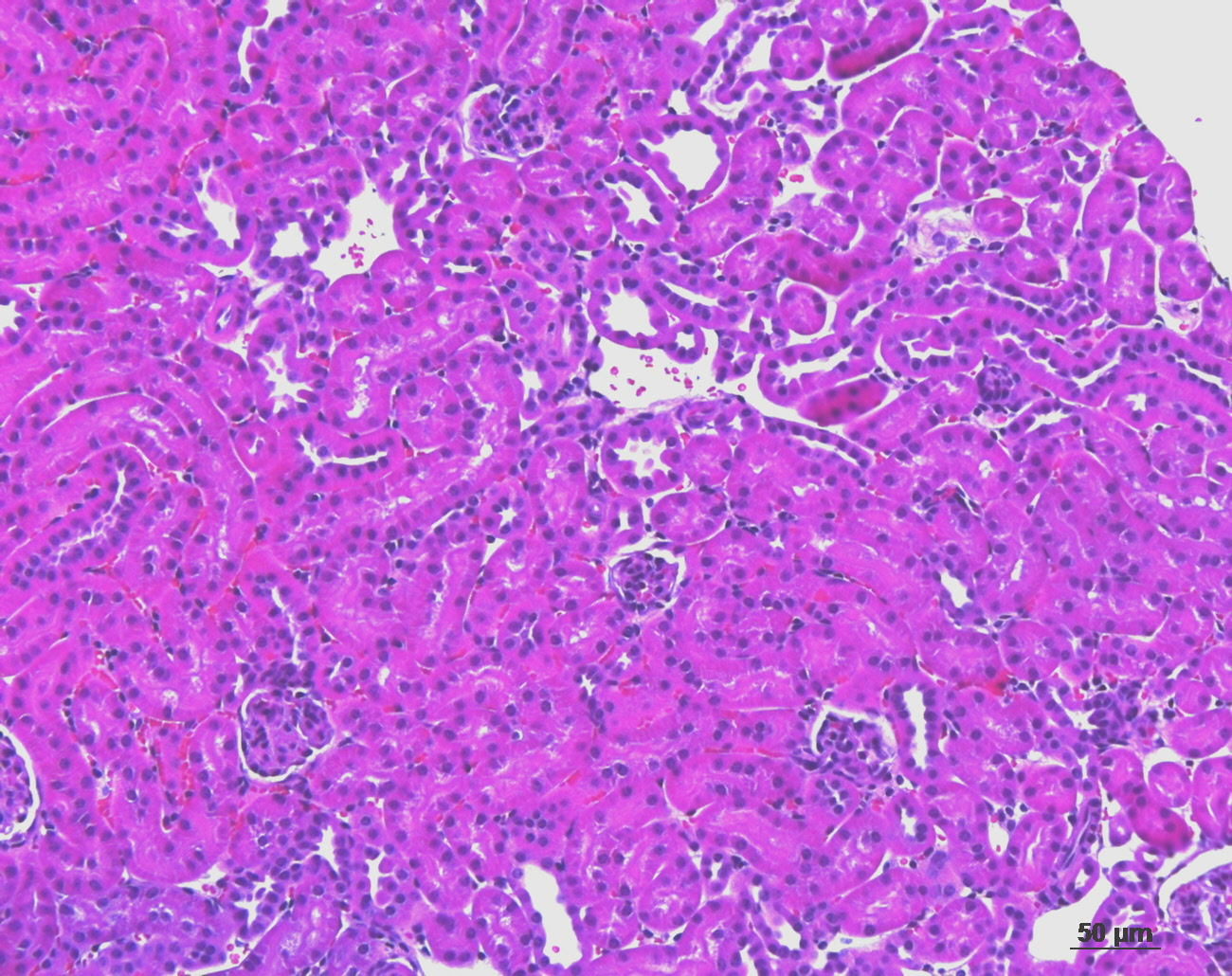

Supplement: Supplementary file 10 — Source data Fig. 2 [file 44321_2025_360_MOESM10_ESM.zip › EMM-2025-22130_SourceDataForFigure 2A-C 10-28-25/2A/TSC1 ECE 75 days H&E 20X.tif]

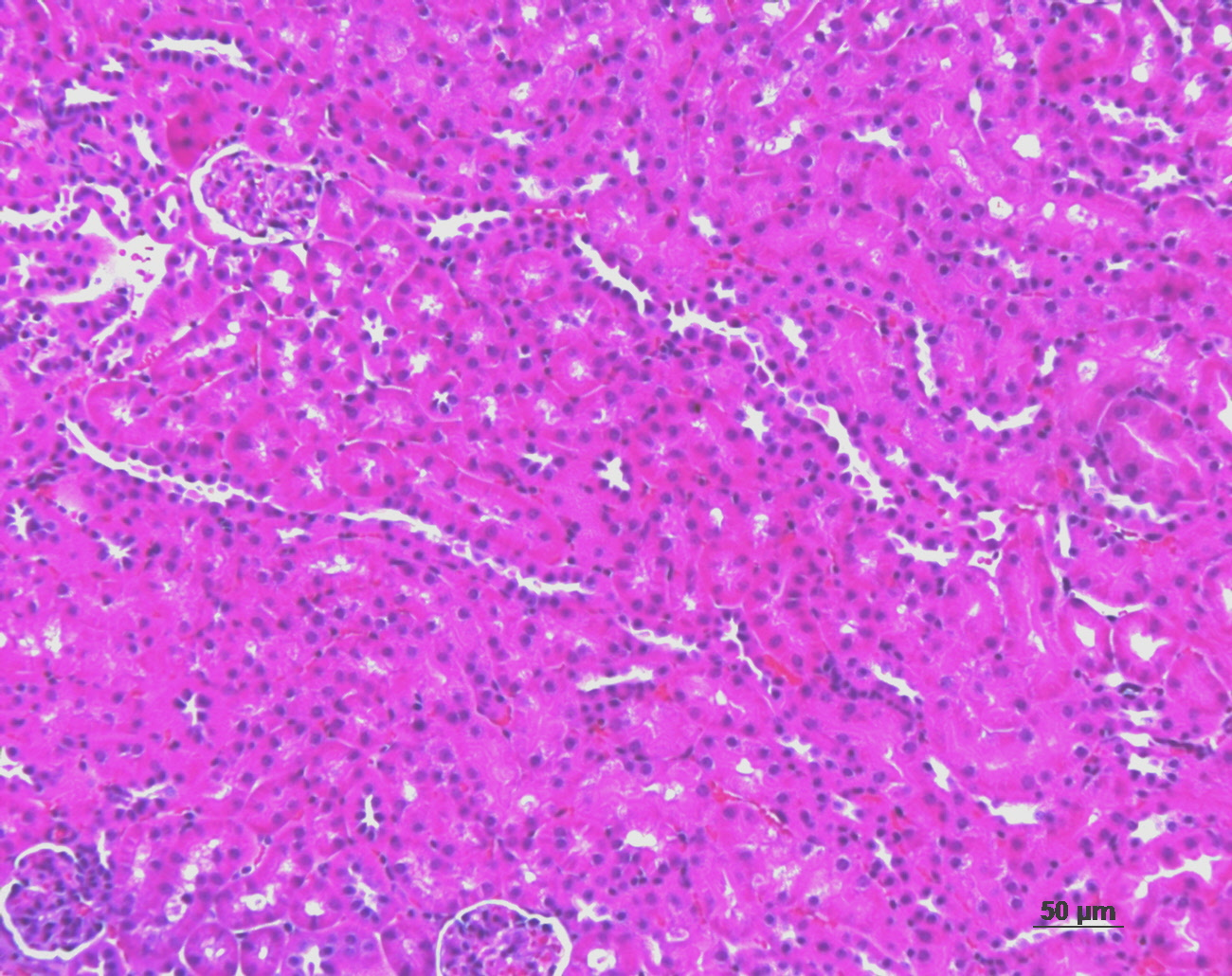

Supplement: Supplementary file 10 — Source data Fig. 2 [file 44321_2025_360_MOESM10_ESM.zip › EMM-2025-22130_SourceDataForFigure 2A-C 10-28-25/2A/WT H&E 20X.tif]

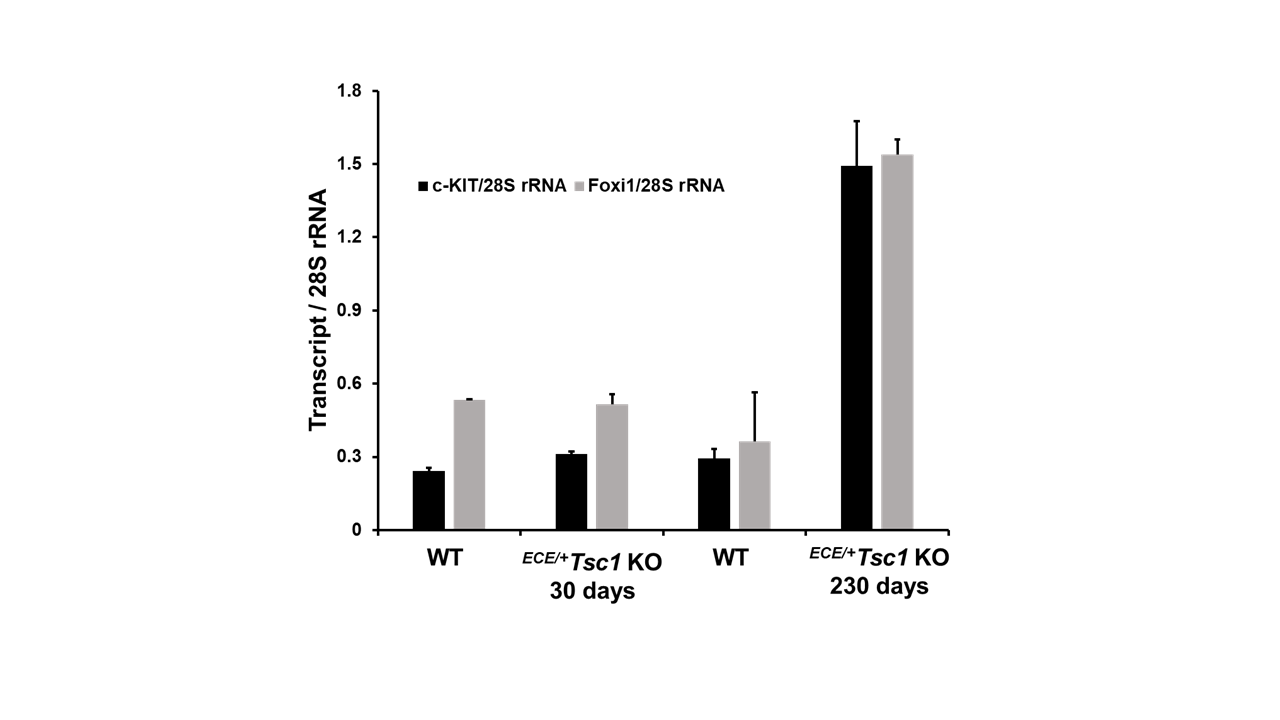

Supplement: Supplementary file 10 — Source data Fig. 2 [file 44321_2025_360_MOESM10_ESM.zip › EMM-2025-22130_SourceDataForFigure 2A-C 10-28-25/2B/Northern Blot Quantification.tif]

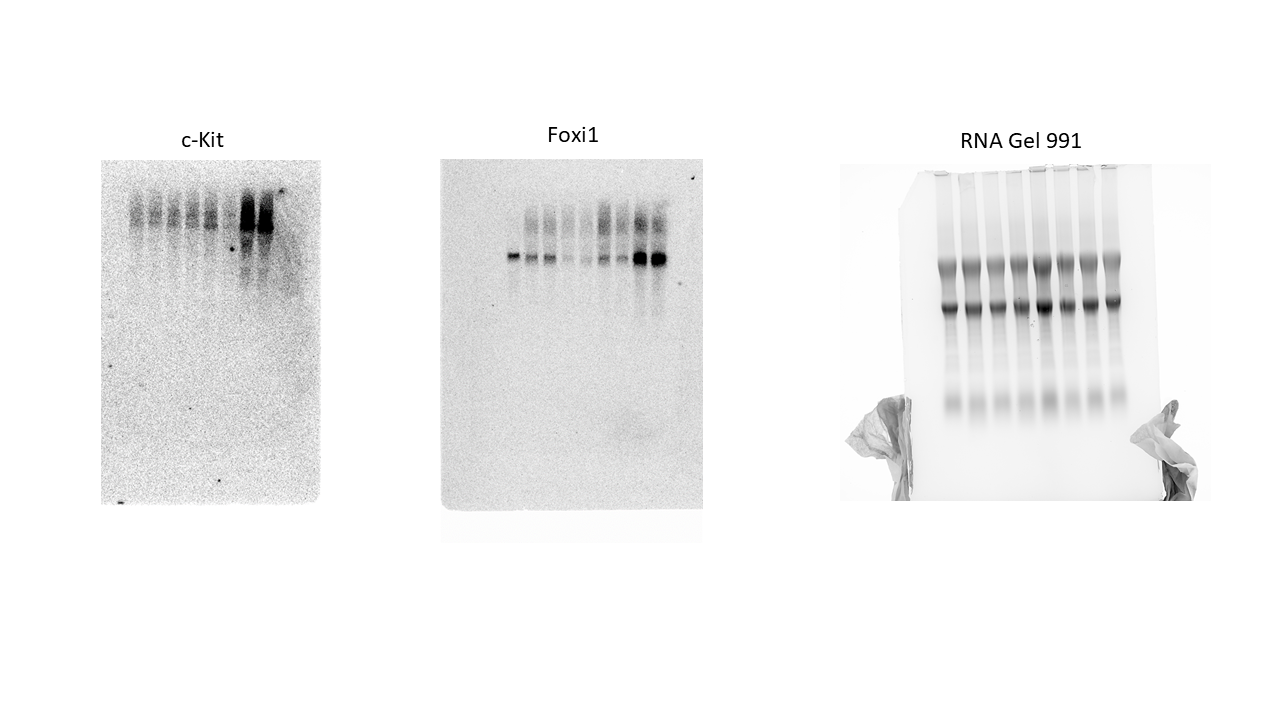

Supplement: Supplementary file 10 — Source data Fig. 2 [file 44321_2025_360_MOESM10_ESM.zip › EMM-2025-22130_SourceDataForFigure 2A-C 10-28-25/2B/Northern Blot Source Data.tif]

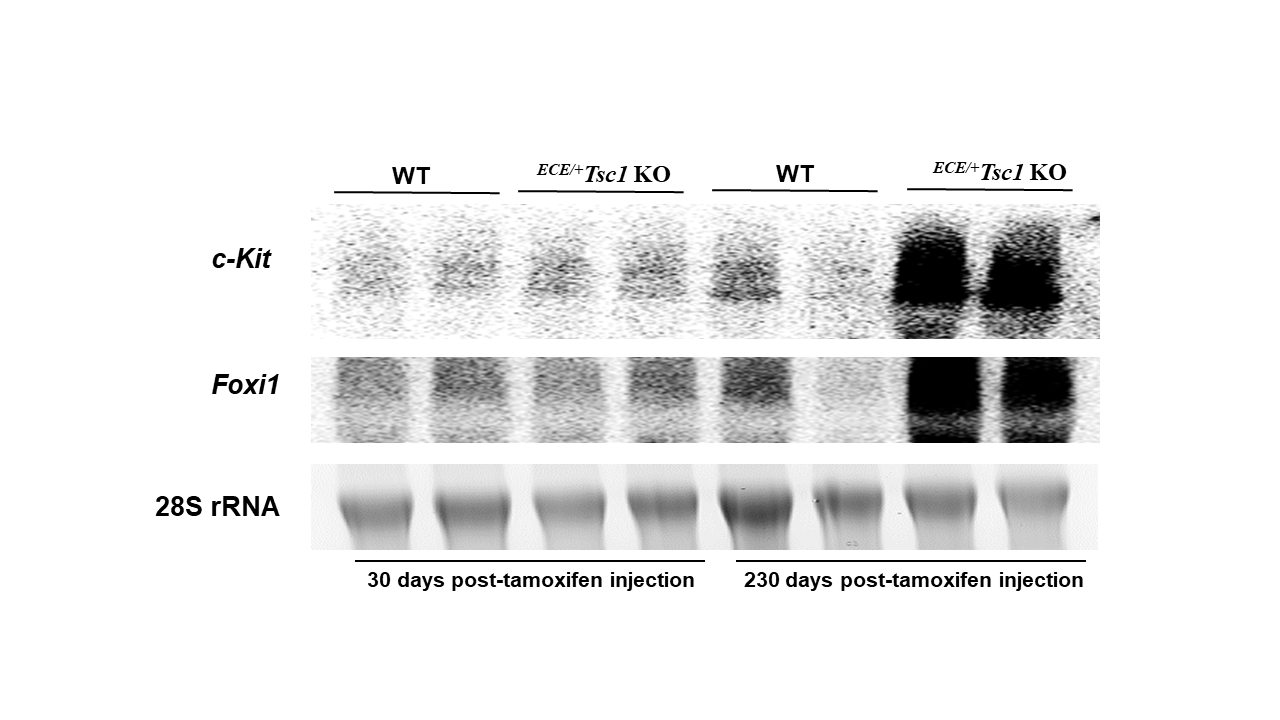

Supplement: Supplementary file 10 — Source data Fig. 2 [file 44321_2025_360_MOESM10_ESM.zip › EMM-2025-22130_SourceDataForFigure 2A-C 10-28-25/2B/Northern Blot.tif]

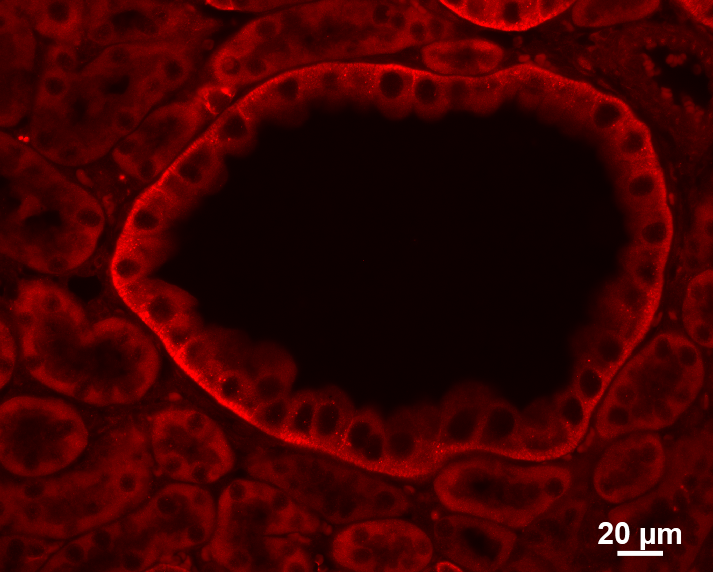

Supplement: Supplementary file 10 — Source data Fig. 2 [file 44321_2025_360_MOESM10_ESM.zip › EMM-2025-22130_SourceDataForFigure 2A-C 10-28-25/2C/c-KIT 40X.tif]

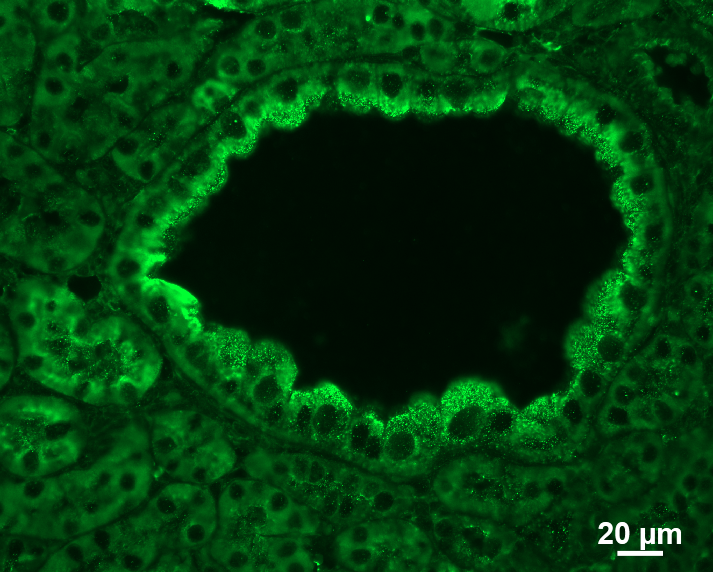

Supplement: Supplementary file 10 — Source data Fig. 2 [file 44321_2025_360_MOESM10_ESM.zip › EMM-2025-22130_SourceDataForFigure 2A-C 10-28-25/2C/HATPase Image 40X.tif]

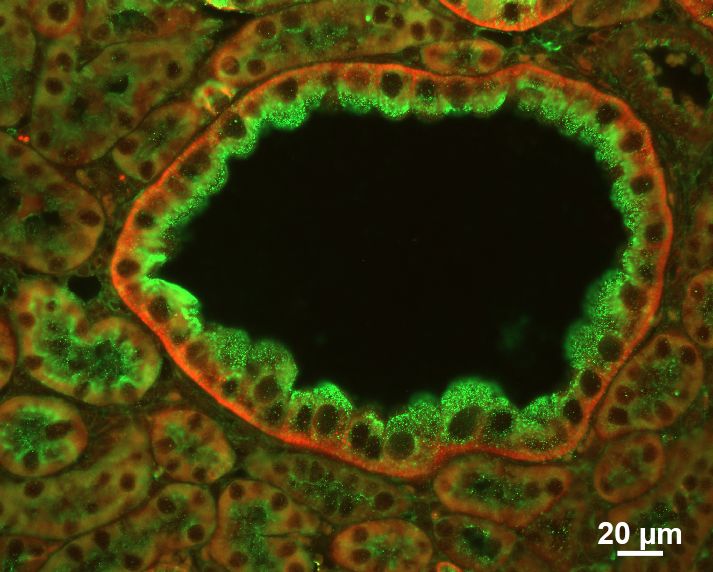

Supplement: Supplementary file 10 — Source data Fig. 2 [file 44321_2025_360_MOESM10_ESM.zip › EMM-2025-22130_SourceDataForFigure 2A-C 10-28-25/2C/Merged Image 40X.tif]

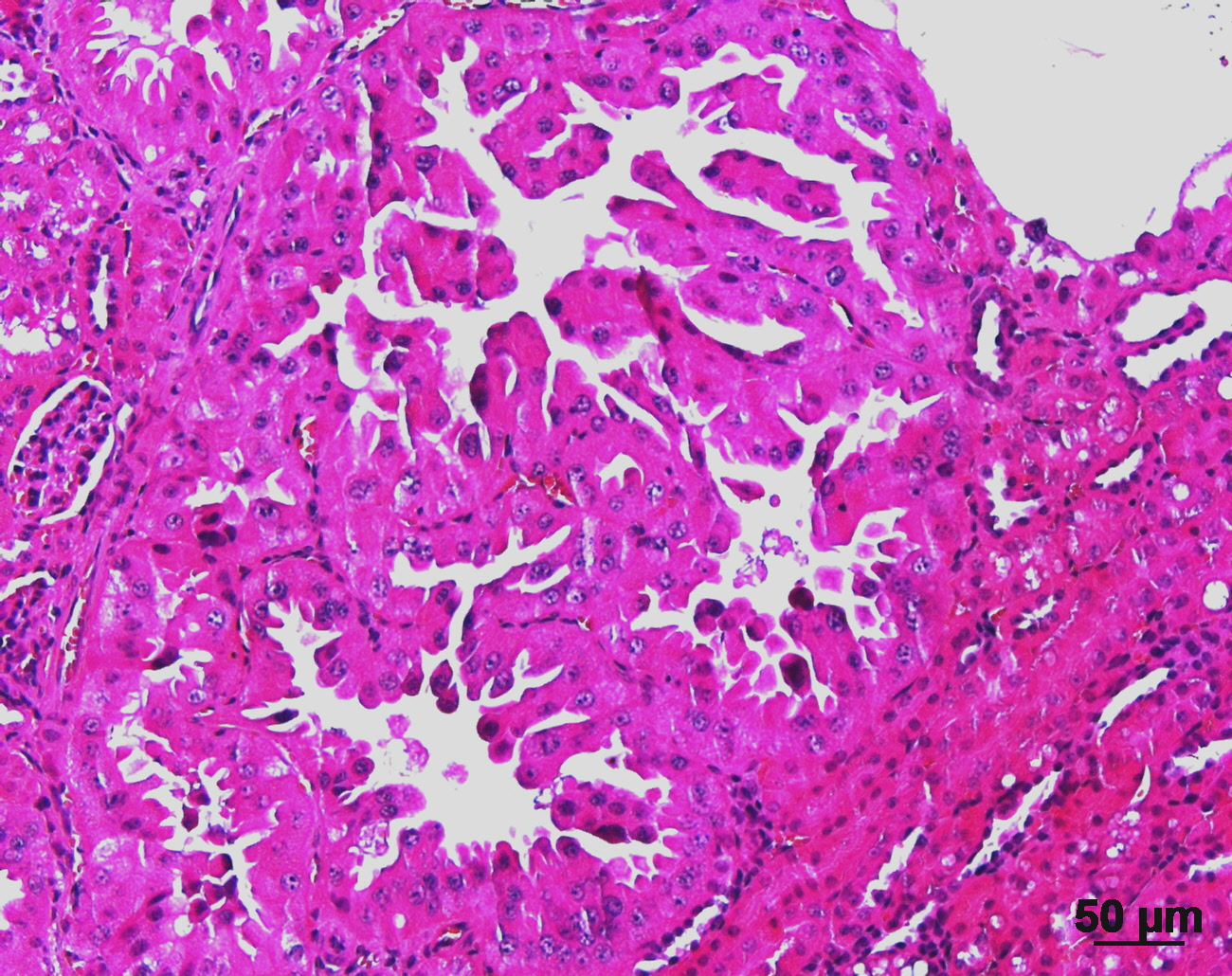

Supplement: Supplementary file 11 — Source data Fig. 3 [file 44321_2025_360_MOESM11_ESM.zip › EMM-2025-22130_SourceDataForFigure 3A-C 10-28-25/3A/Tsc2 Het 10 months H&E 20X.tif]

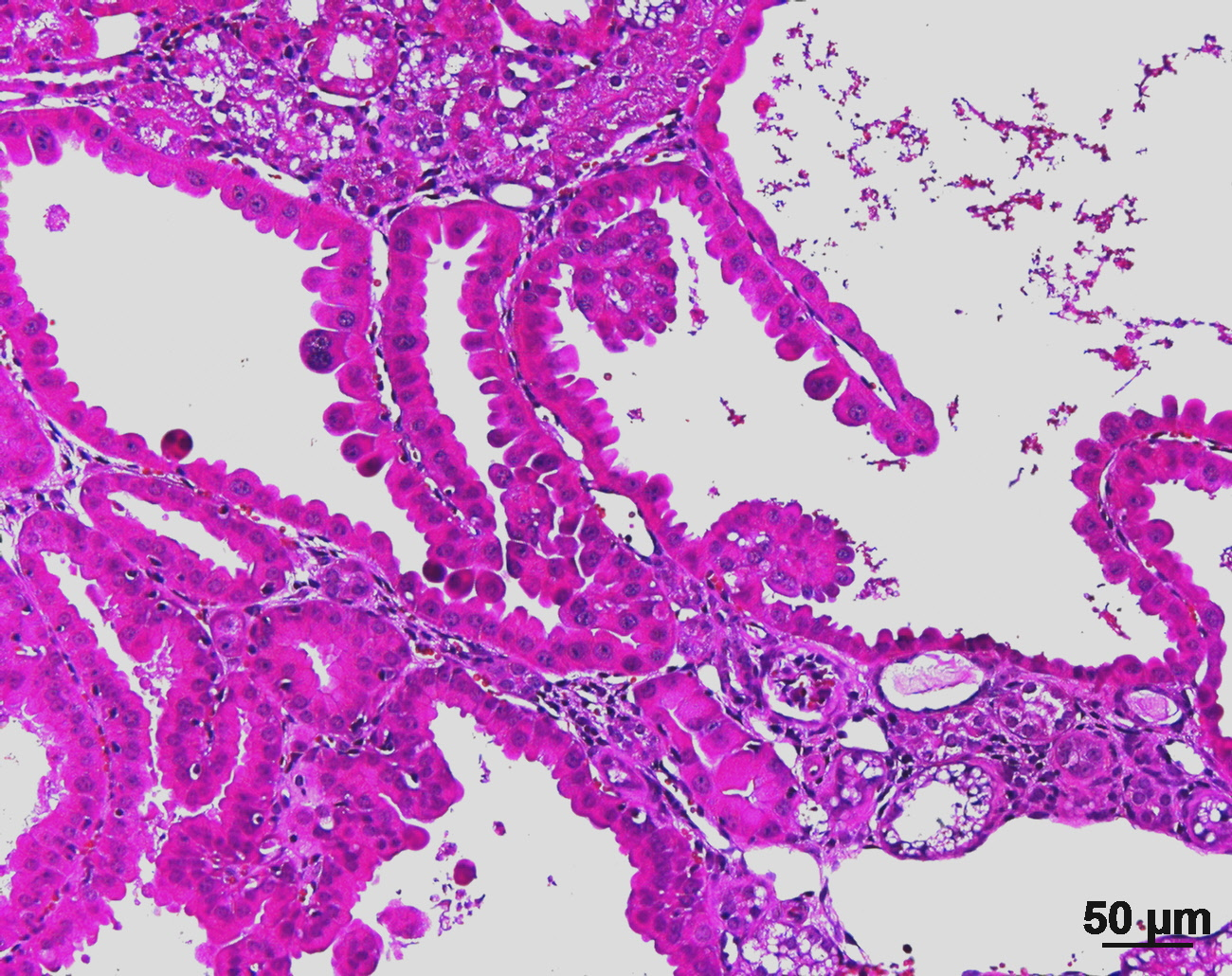

Supplement: Supplementary file 11 — Source data Fig. 3 [file 44321_2025_360_MOESM11_ESM.zip › EMM-2025-22130_SourceDataForFigure 3A-C 10-28-25/3A/Tsc2 Het 15 months H&E 20X.tif]

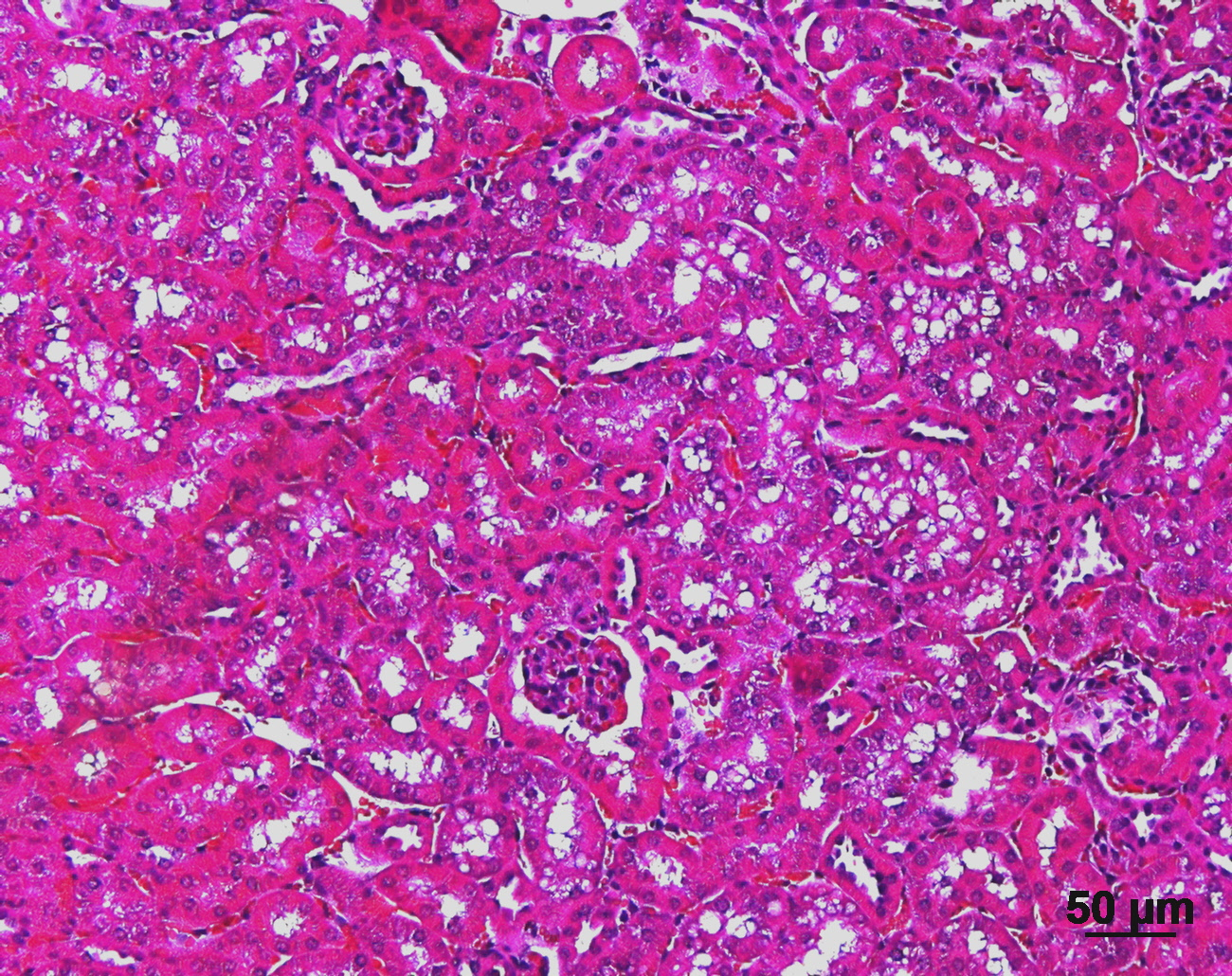

Supplement: Supplementary file 11 — Source data Fig. 3 [file 44321_2025_360_MOESM11_ESM.zip › EMM-2025-22130_SourceDataForFigure 3A-C 10-28-25/3A/Tsc2 Het 6 months H&E 20X.tif]

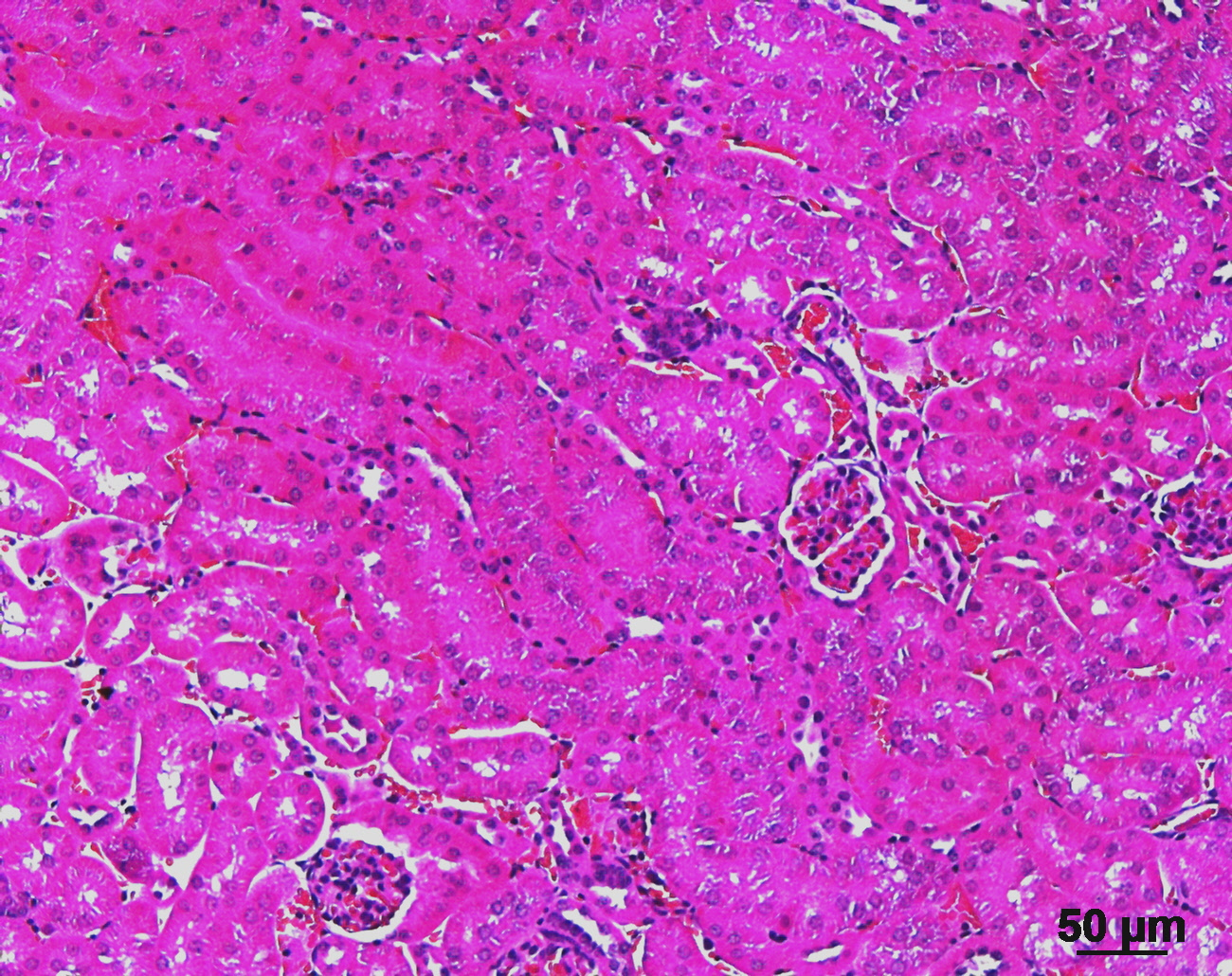

Supplement: Supplementary file 11 — Source data Fig. 3 [file 44321_2025_360_MOESM11_ESM.zip › EMM-2025-22130_SourceDataForFigure 3A-C 10-28-25/3A/WT H&E 20X.tif]

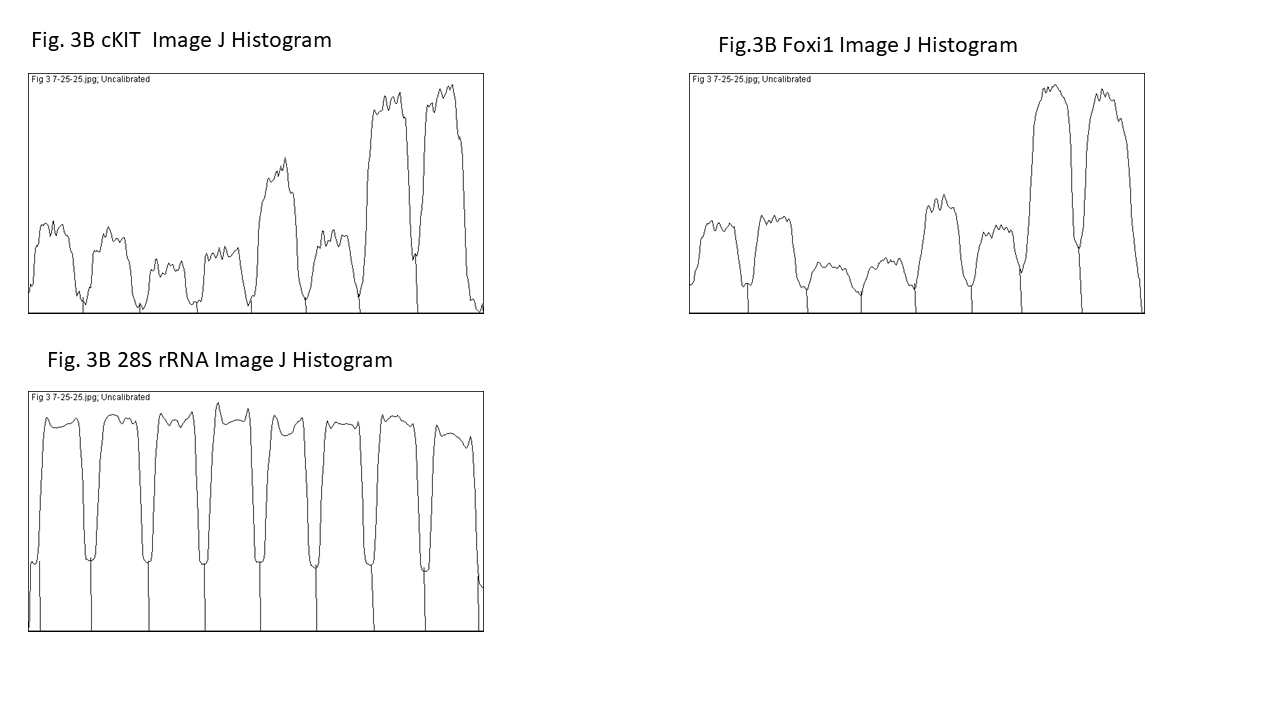

Supplement: Supplementary file 11 — Source data Fig. 3 [file 44321_2025_360_MOESM11_ESM.zip › EMM-2025-22130_SourceDataForFigure 3A-C 10-28-25/3B/Northern Blot Image J Histograms.tif]

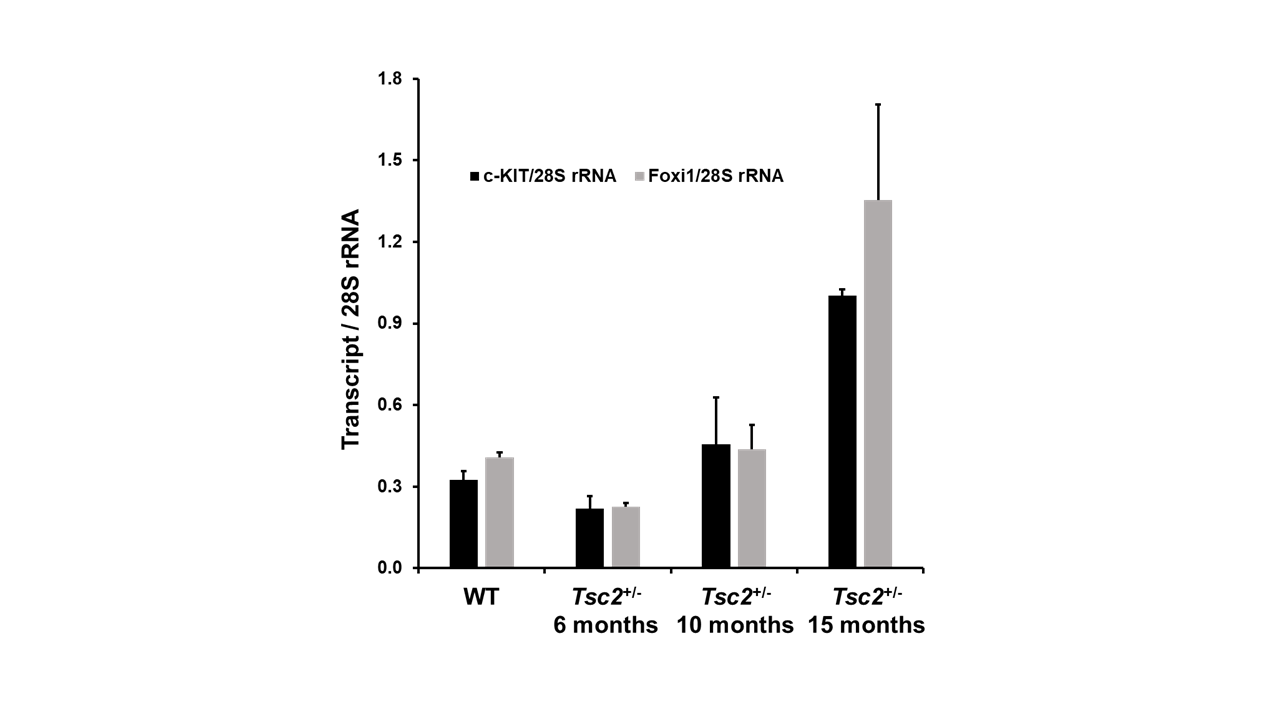

Supplement: Supplementary file 11 — Source data Fig. 3 [file 44321_2025_360_MOESM11_ESM.zip › EMM-2025-22130_SourceDataForFigure 3A-C 10-28-25/3B/Northern Blot Quantification.tif]

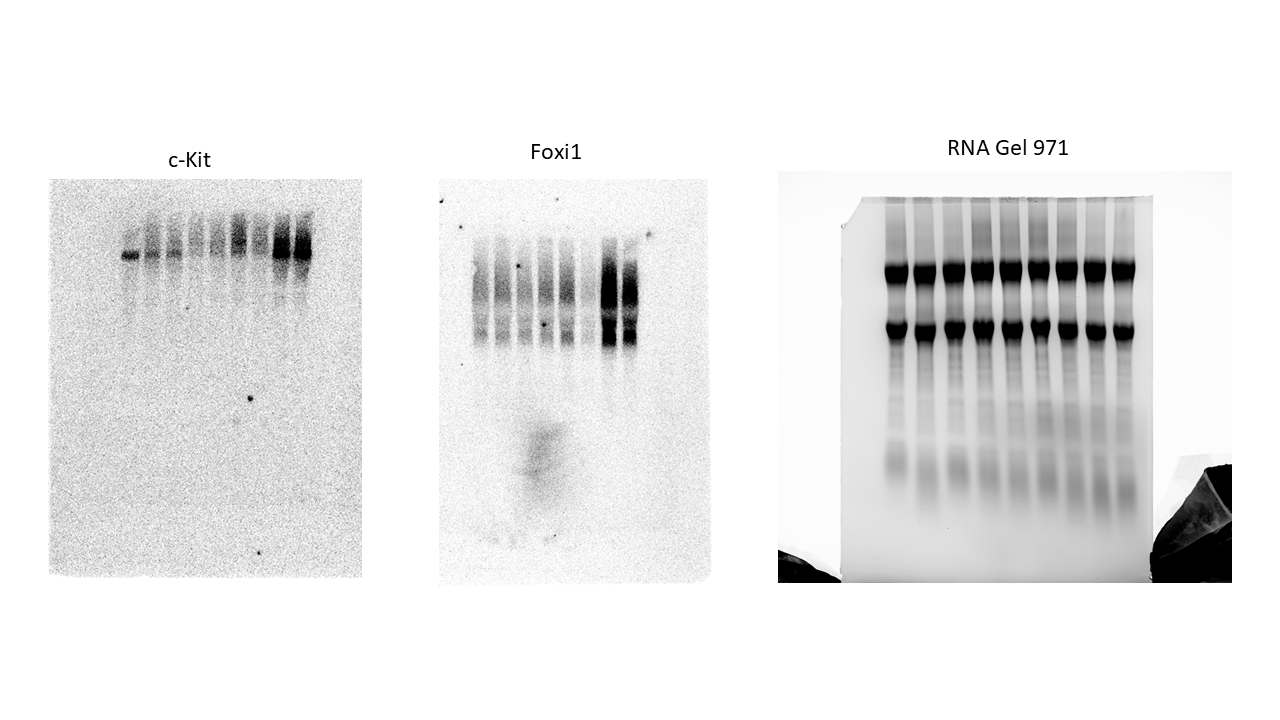

Supplement: Supplementary file 11 — Source data Fig. 3 [file 44321_2025_360_MOESM11_ESM.zip › EMM-2025-22130_SourceDataForFigure 3A-C 10-28-25/3B/Northern Blot Source Data.tif]

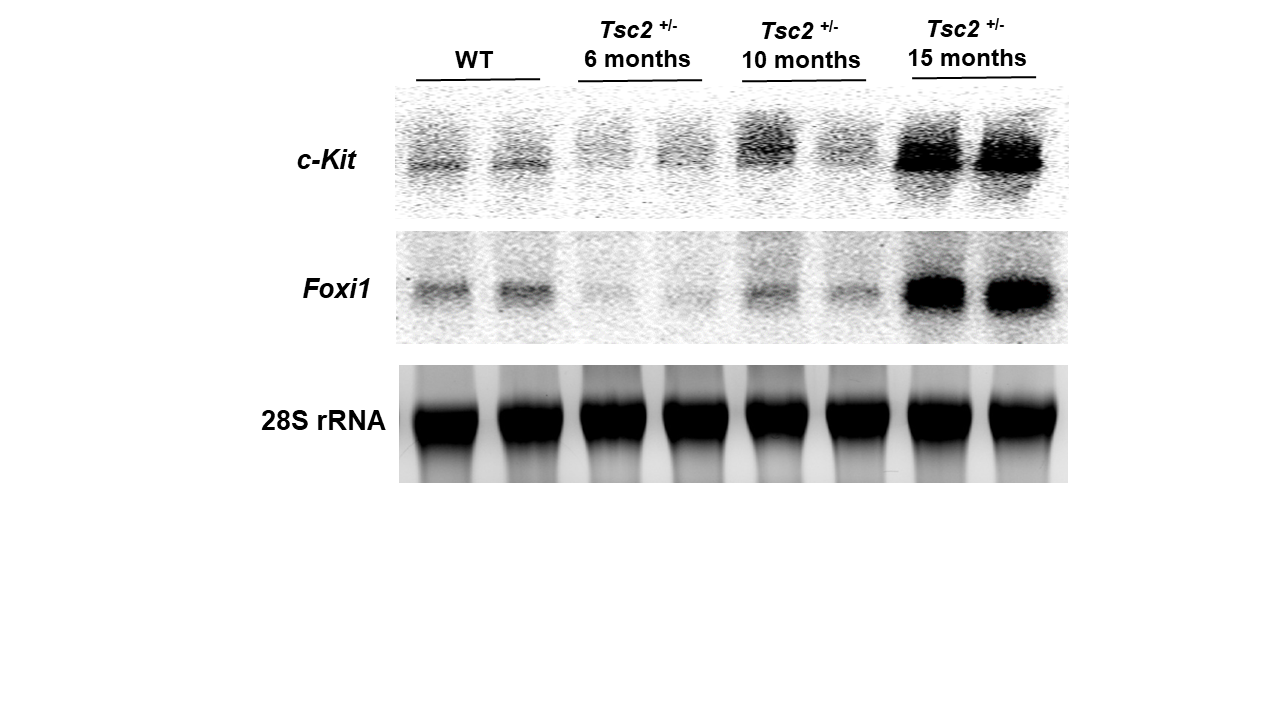

Supplement: Supplementary file 11 — Source data Fig. 3 [file 44321_2025_360_MOESM11_ESM.zip › EMM-2025-22130_SourceDataForFigure 3A-C 10-28-25/3B/Northern Blot.tif]

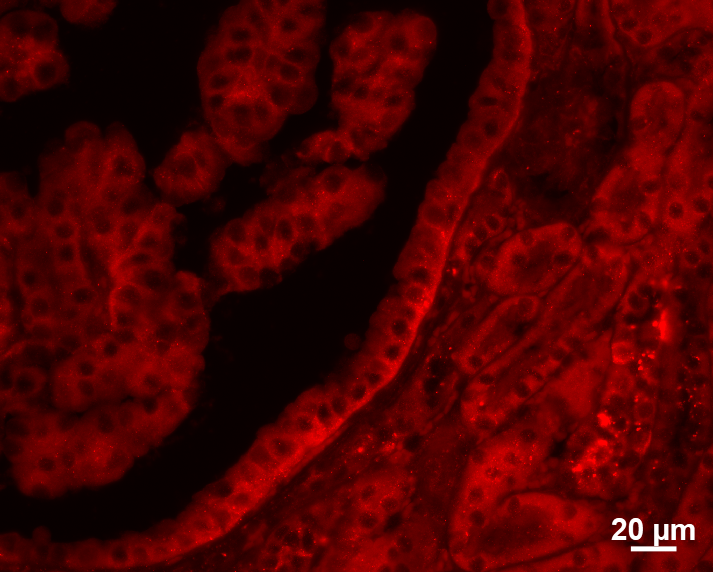

Supplement: Supplementary file 11 — Source data Fig. 3 [file 44321_2025_360_MOESM11_ESM.zip › EMM-2025-22130_SourceDataForFigure 3A-C 10-28-25/3C/c-KIT Image 40X.tif]

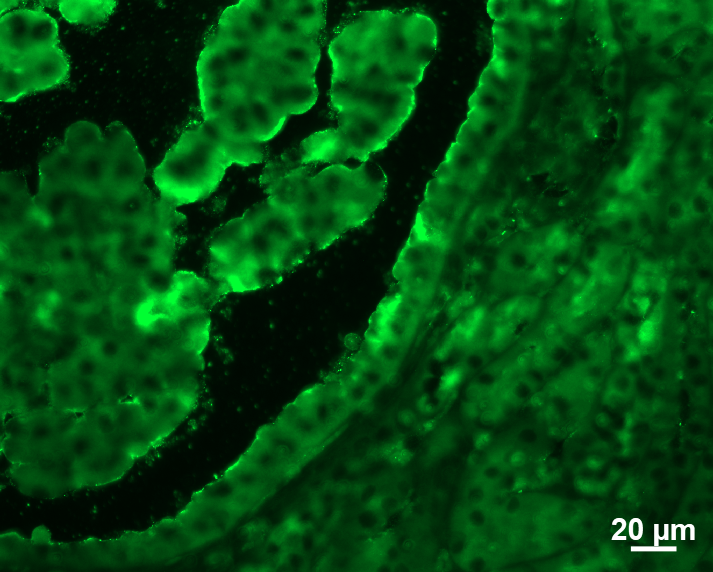

Supplement: Supplementary file 11 — Source data Fig. 3 [file 44321_2025_360_MOESM11_ESM.zip › EMM-2025-22130_SourceDataForFigure 3A-C 10-28-25/3C/HATPase Image 40X.tif]

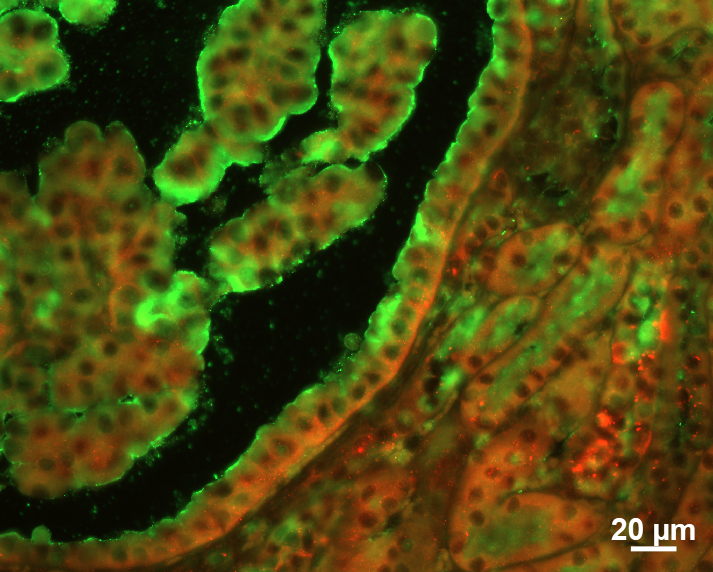

Supplement: Supplementary file 11 — Source data Fig. 3 [file 44321_2025_360_MOESM11_ESM.zip › EMM-2025-22130_SourceDataForFigure 3A-C 10-28-25/3C/Merged Image 40X.tif]

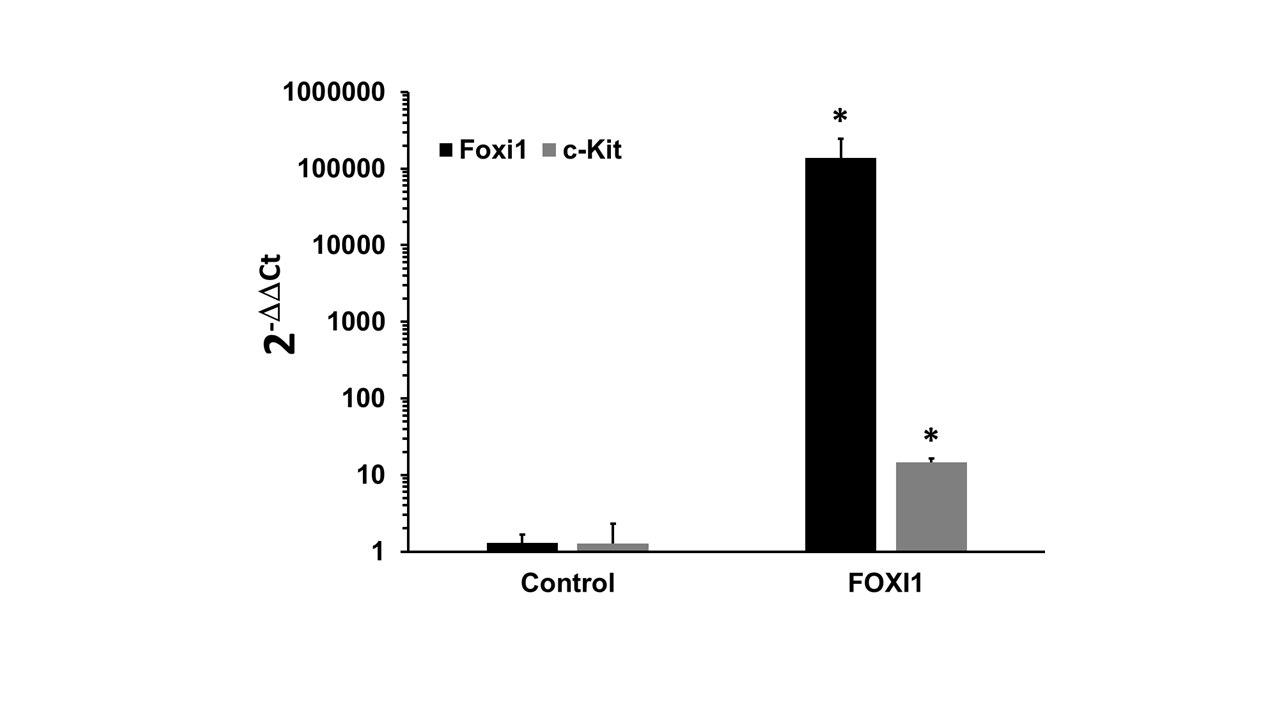

Supplement: Supplementary file 12 — Source data Fig. 4 [file 44321_2025_360_MOESM12_ESM.zip › EMM-2025-22130_Source DataForFigure 4A-D 10-28-25/4A/Graph-Numerical Data.tif]

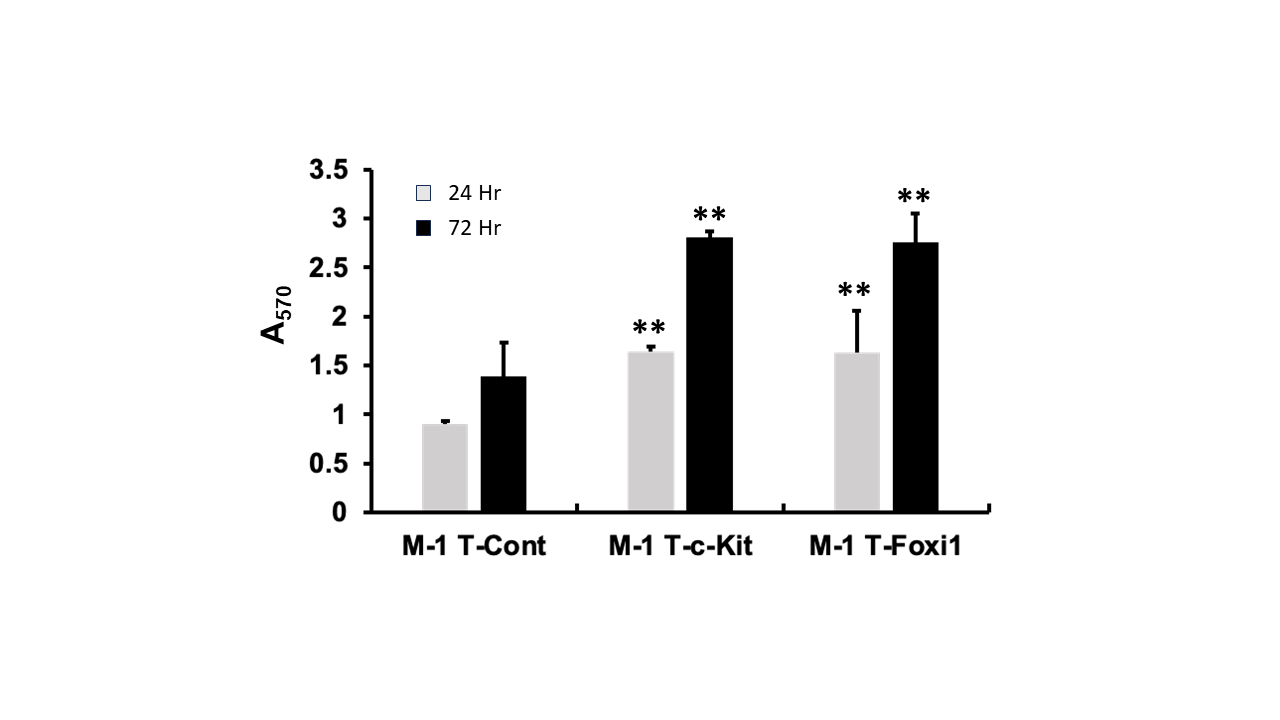

Supplement: Supplementary file 12 — Source data Fig. 4 [file 44321_2025_360_MOESM12_ESM.zip › EMM-2025-22130_Source DataForFigure 4A-D 10-28-25/4B/Graph--Numerical Data.tif]

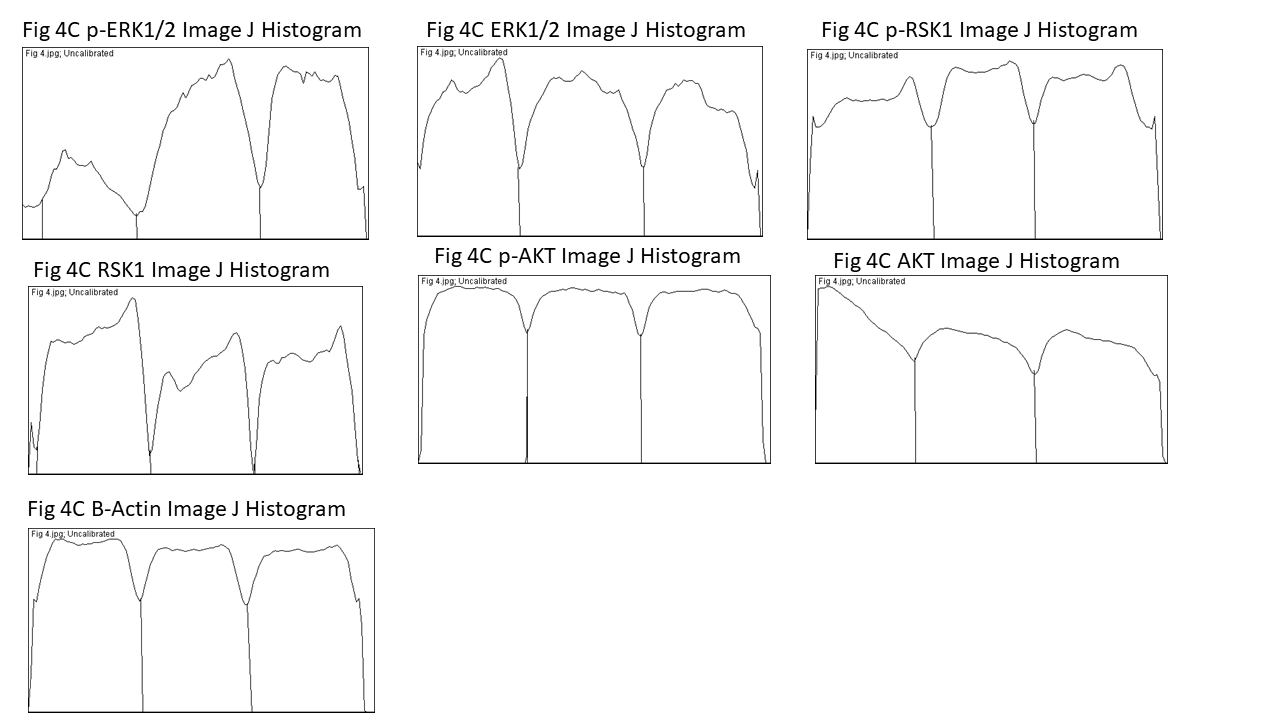

Supplement: Supplementary file 12 — Source data Fig. 4 [file 44321_2025_360_MOESM12_ESM.zip › EMM-2025-22130_Source DataForFigure 4A-D 10-28-25/4C/Western Blot Image J Histograms.tif]

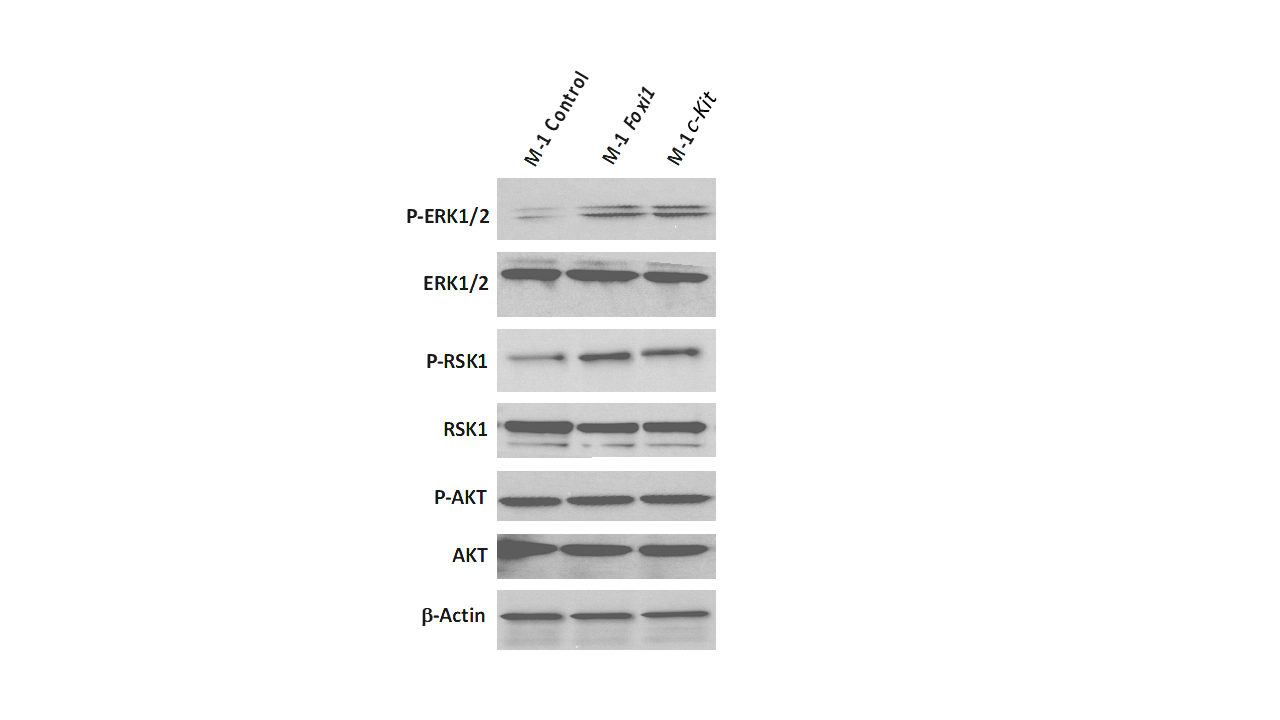

Supplement: Supplementary file 12 — Source data Fig. 4 [file 44321_2025_360_MOESM12_ESM.zip › EMM-2025-22130_Source DataForFigure 4A-D 10-28-25/4C/Western Blots.tif]

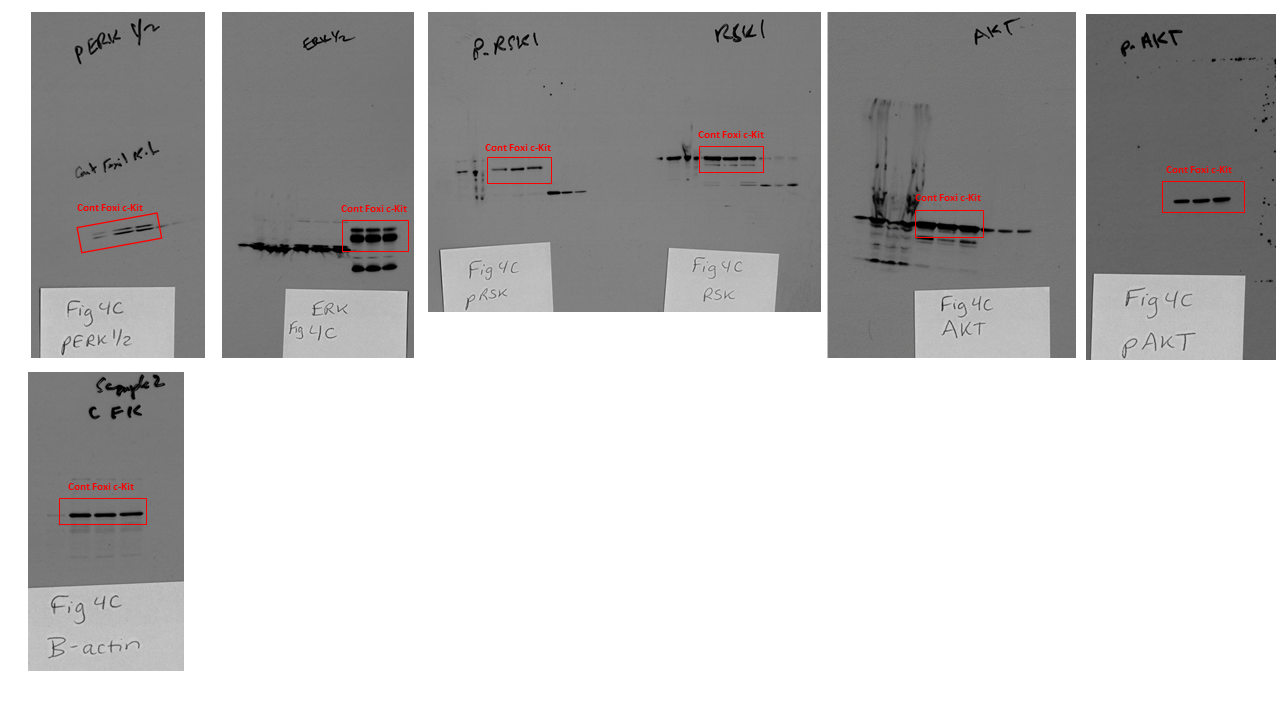

Supplement: Supplementary file 12 — Source data Fig. 4 [file 44321_2025_360_MOESM12_ESM.zip › EMM-2025-22130_Source DataForFigure 4A-D 10-28-25/4C/Western Source Data/Slide1.TIF]

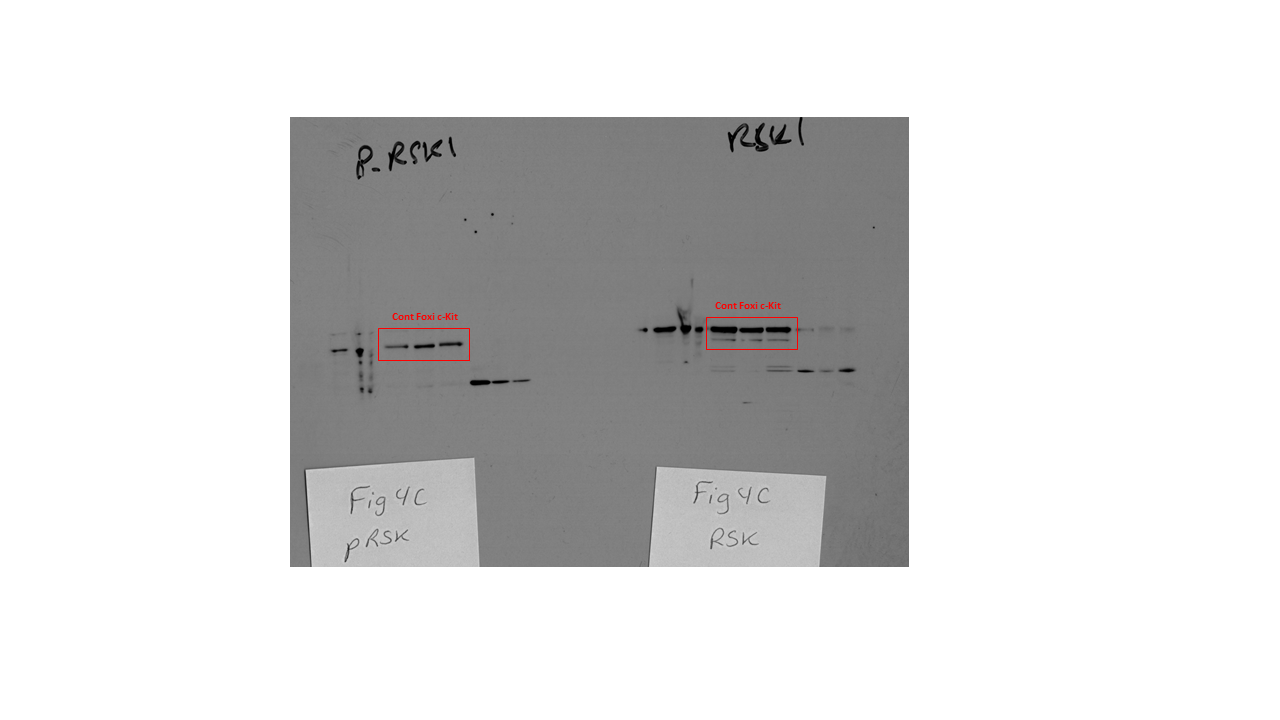

Supplement: Supplementary file 12 — Source data Fig. 4 [file 44321_2025_360_MOESM12_ESM.zip › EMM-2025-22130_Source DataForFigure 4A-D 10-28-25/4C/Western Source Data/Slide2.TIF]

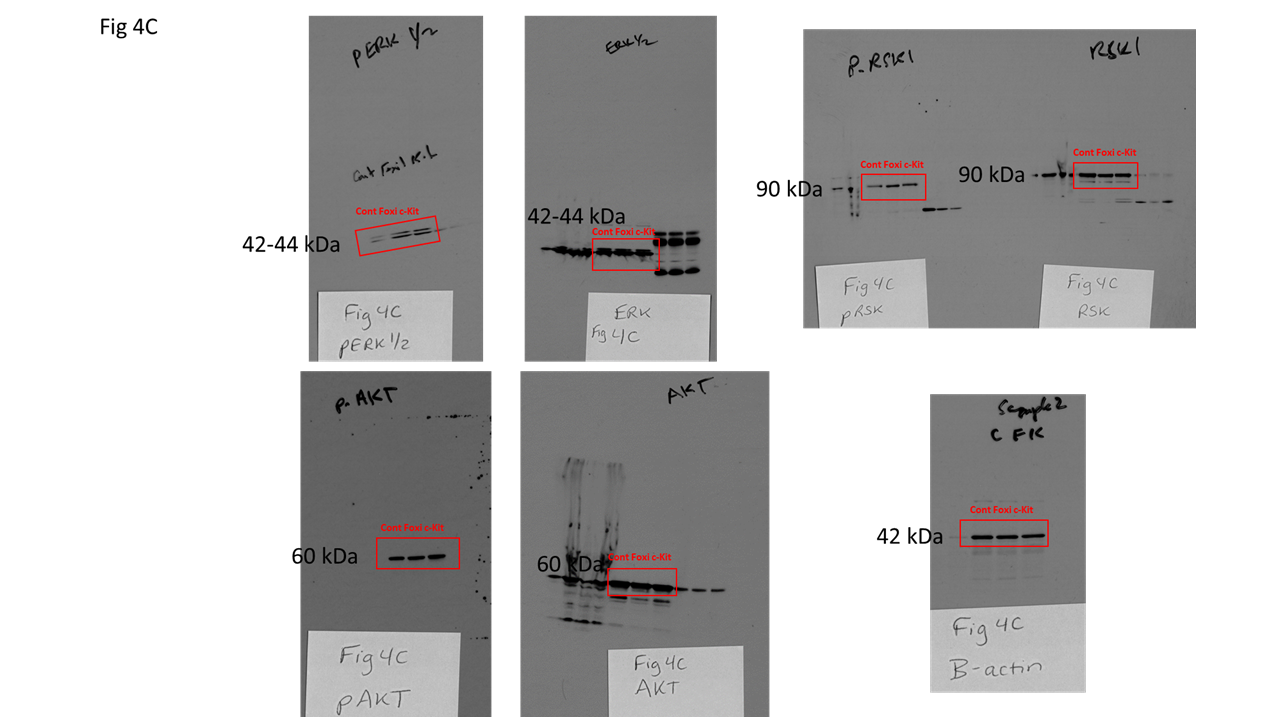

Supplement: Supplementary file 12 — Source data Fig. 4 [file 44321_2025_360_MOESM12_ESM.zip › EMM-2025-22130_Source DataForFigure 4A-D 10-28-25/4C/Western Source Data 10-29-25.tif]

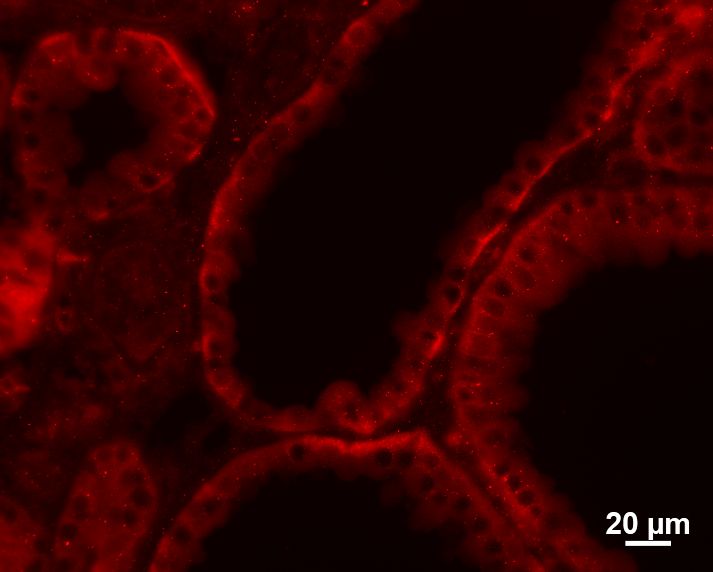

Supplement: Supplementary file 12 — Source data Fig. 4 [file 44321_2025_360_MOESM12_ESM.zip › EMM-2025-22130_Source DataForFigure 4A-D 10-28-25/4D/c-KIT Image 40X.tif]

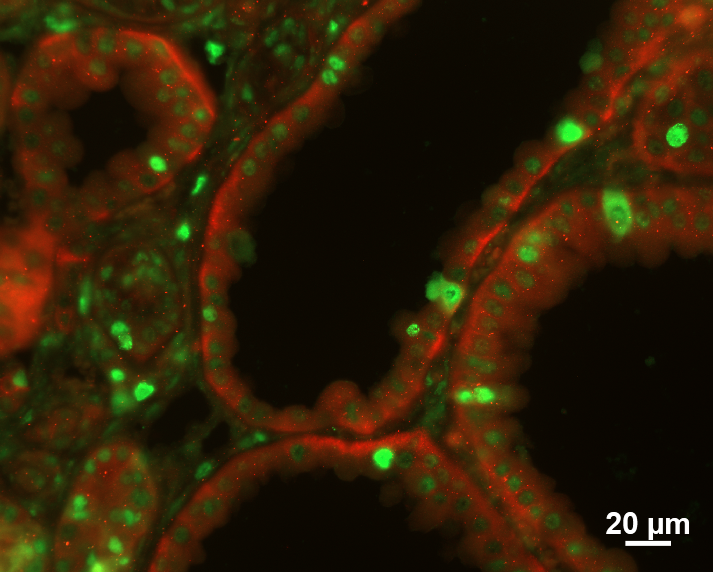

Supplement: Supplementary file 12 — Source data Fig. 4 [file 44321_2025_360_MOESM12_ESM.zip › EMM-2025-22130_Source DataForFigure 4A-D 10-28-25/4D/Merged Image 40X.tif]

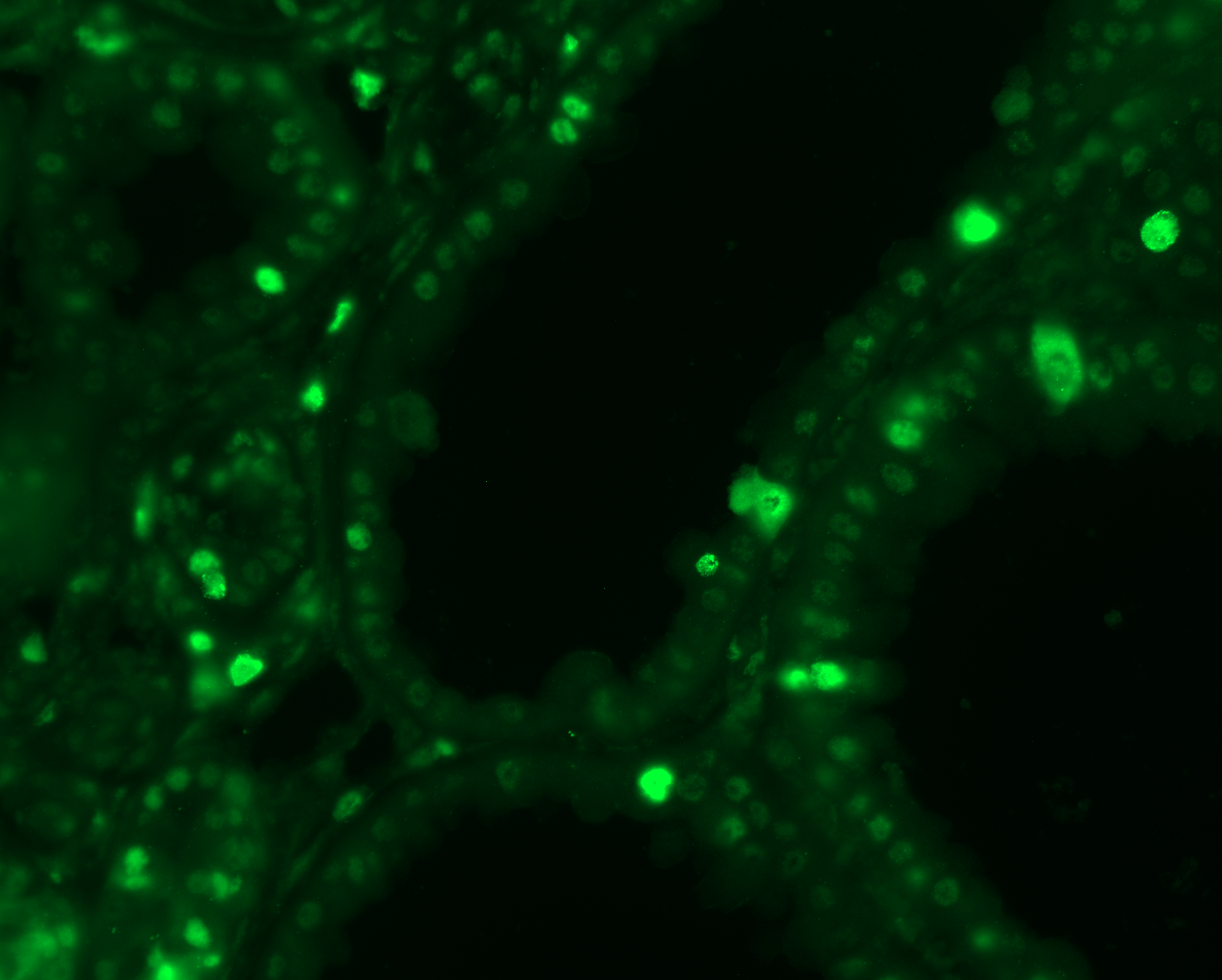

Supplement: Supplementary file 12 — Source data Fig. 4 [file 44321_2025_360_MOESM12_ESM.zip › EMM-2025-22130_Source DataForFigure 4A-D 10-28-25/4D/PCNA Image 40X.tif]

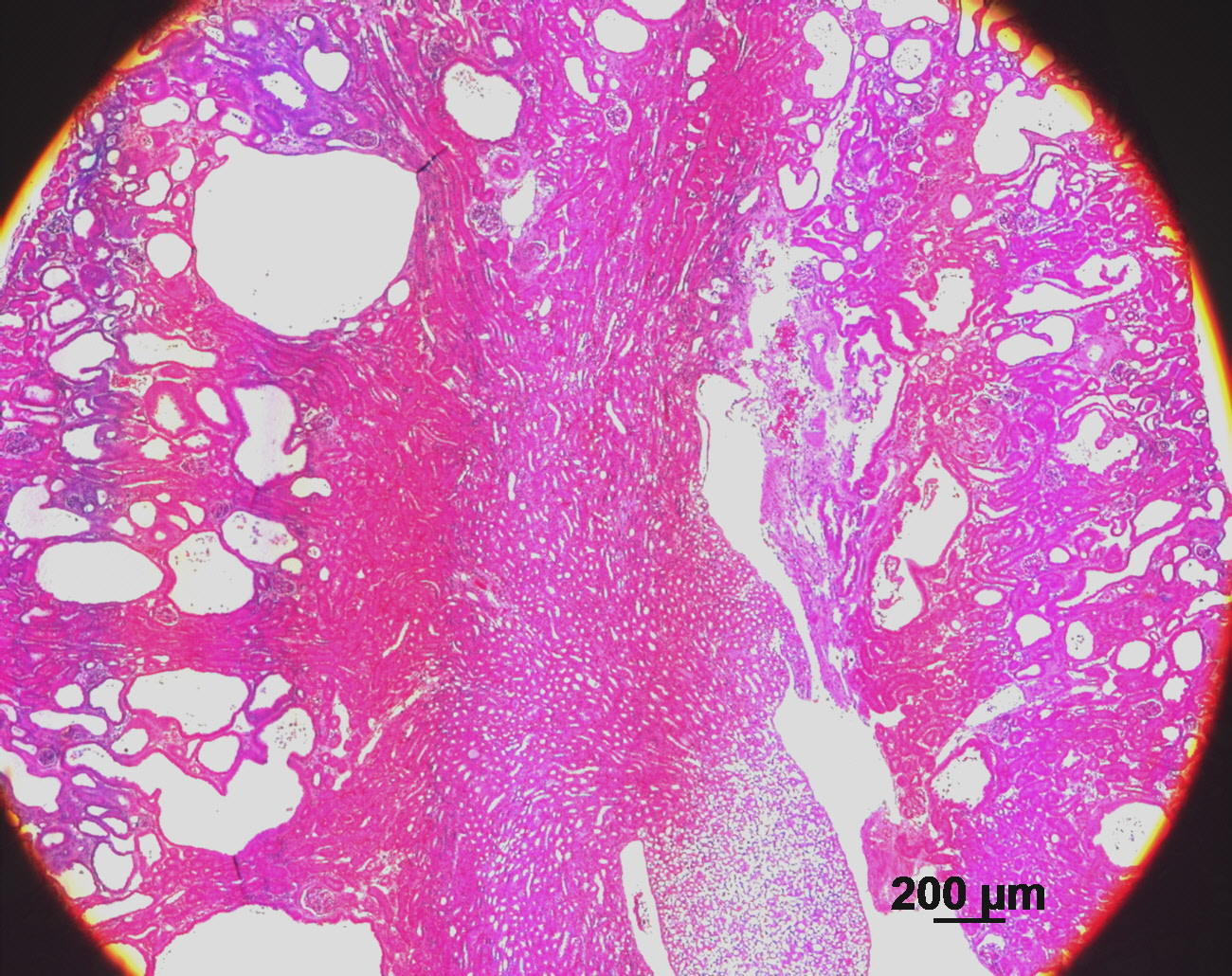

Supplement: Supplementary file 13 — Source data Fig. 5 [file 44321_2025_360_MOESM13_ESM.zip › EMM-2025-22130_SourceDataForFigure 5A-E 10-28-25/5A/Tsc1 KO H&E 4X.tif]

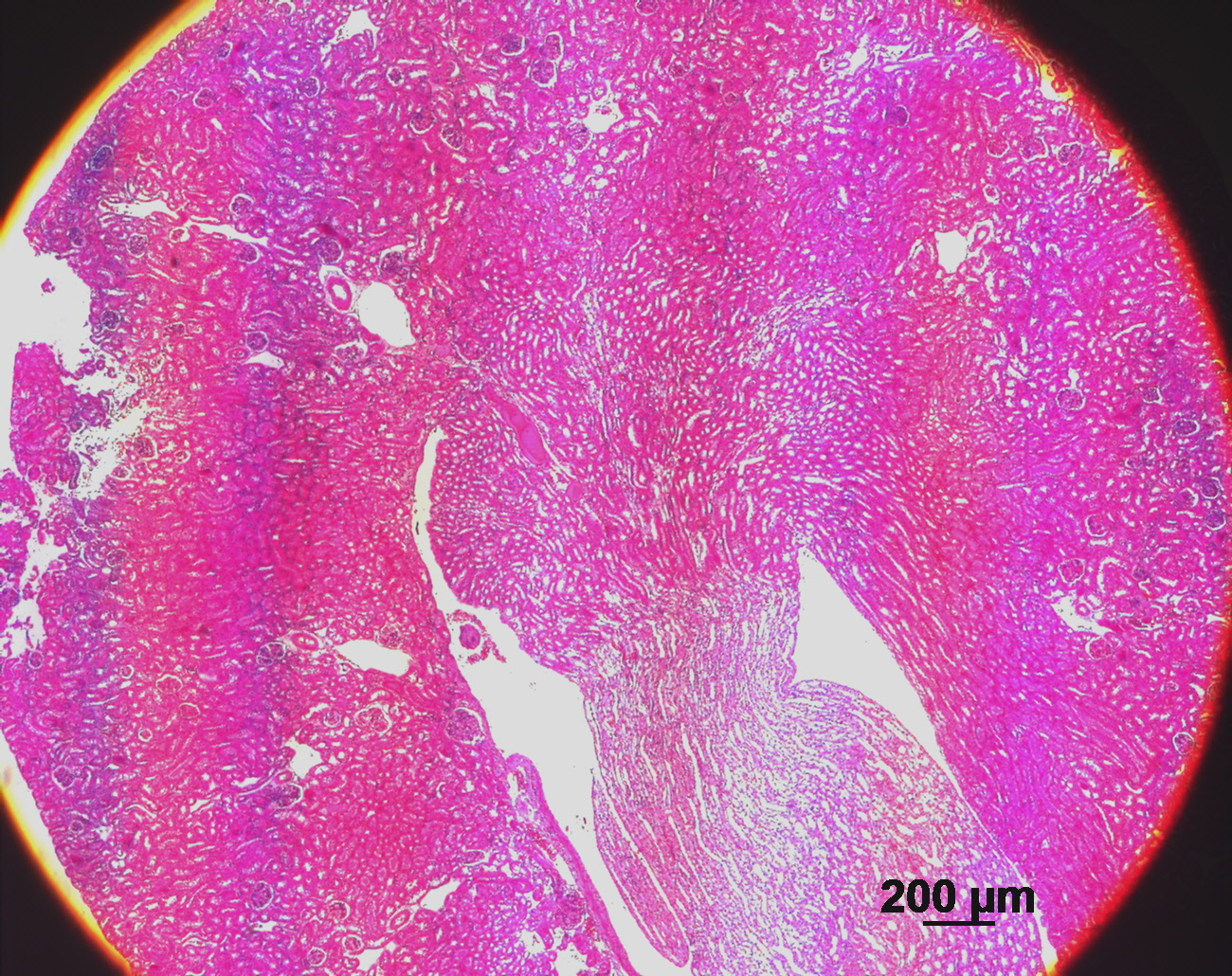

Supplement: Supplementary file 13 — Source data Fig. 5 [file 44321_2025_360_MOESM13_ESM.zip › EMM-2025-22130_SourceDataForFigure 5A-E 10-28-25/5A/Tsc1-cKIT dKO 45 days H&E 4X.tif]

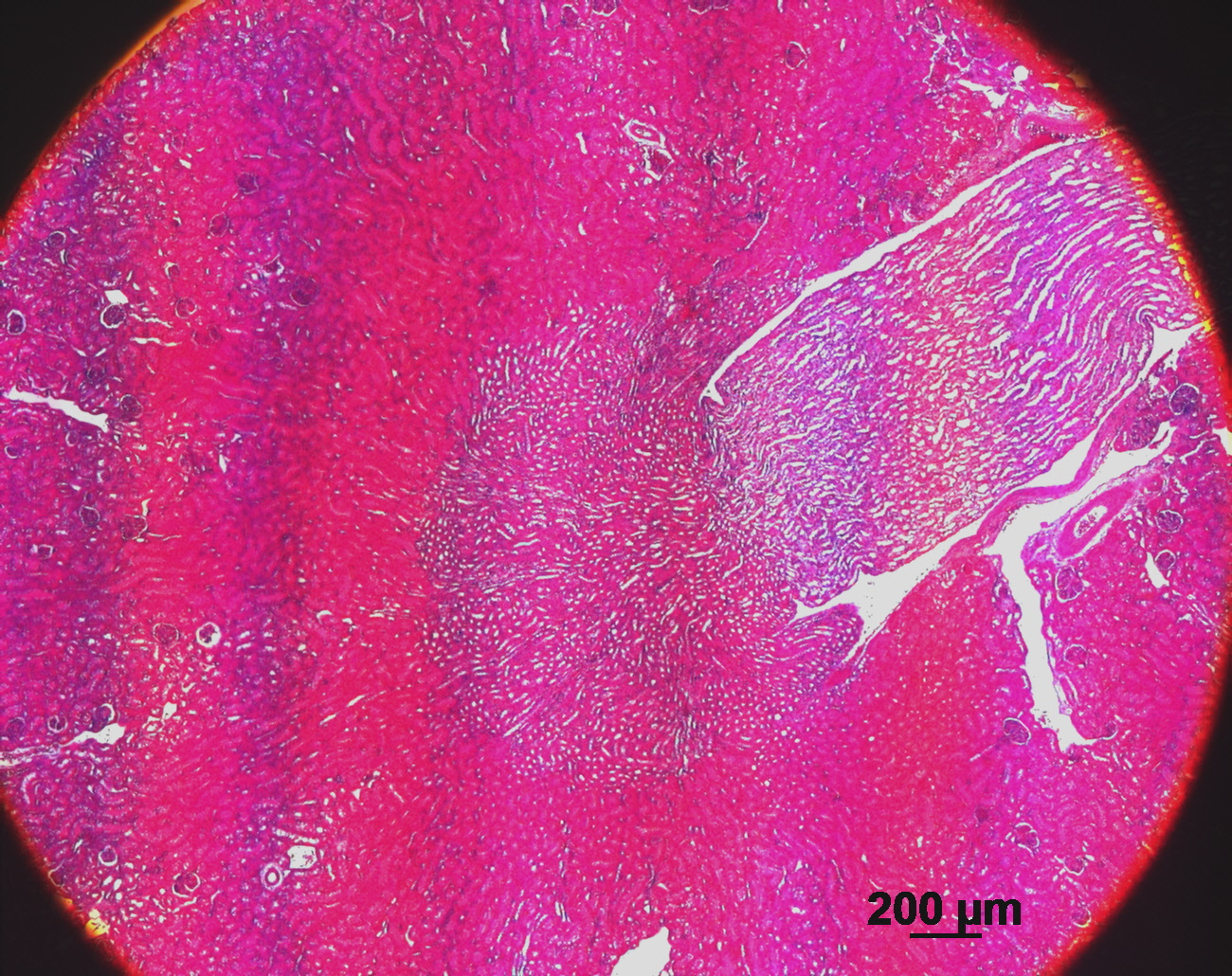

Supplement: Supplementary file 13 — Source data Fig. 5 [file 44321_2025_360_MOESM13_ESM.zip › EMM-2025-22130_SourceDataForFigure 5A-E 10-28-25/5A/Tsc1-cKIT dKO 56 days H&E 4X.tif]

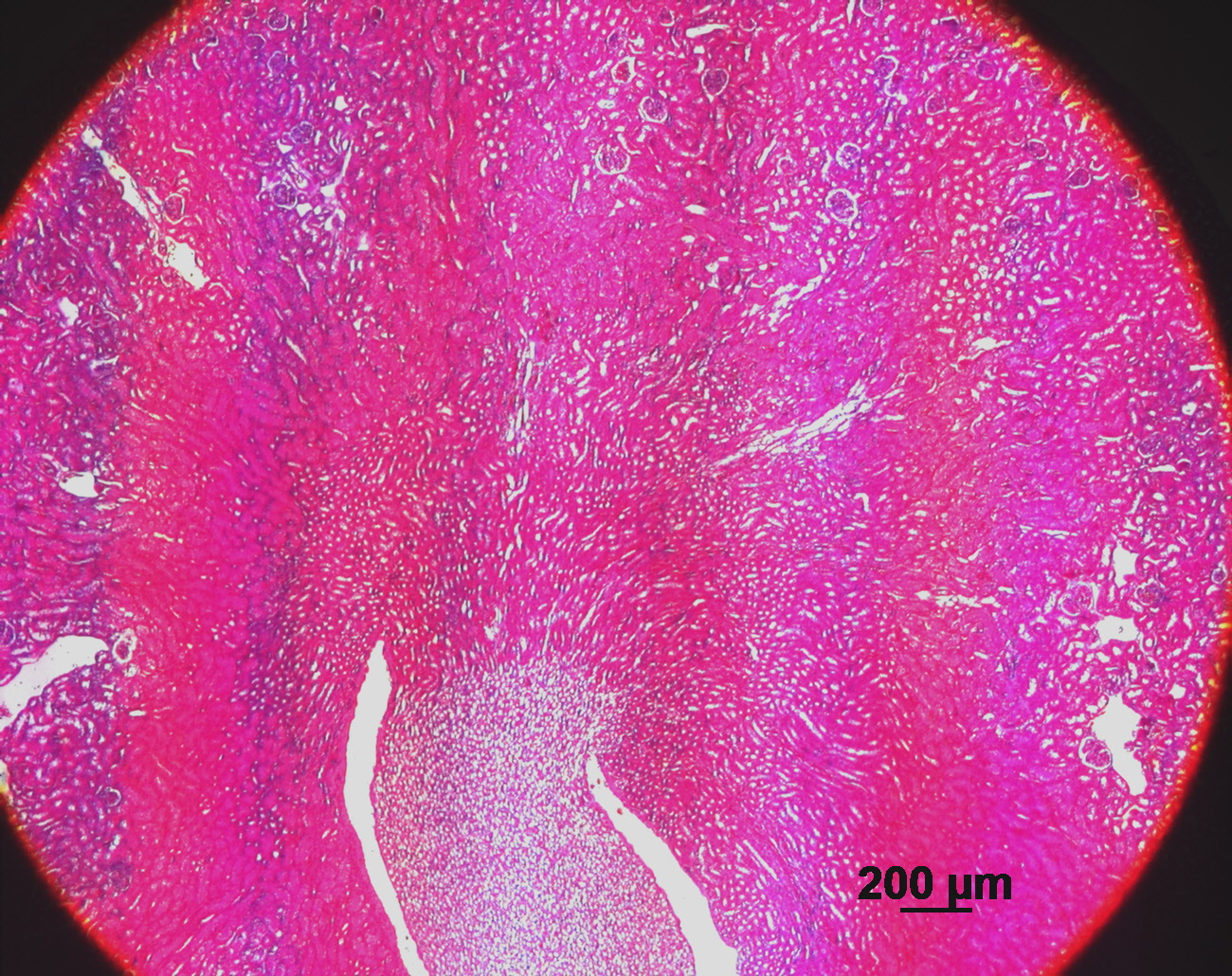

Supplement: Supplementary file 13 — Source data Fig. 5 [file 44321_2025_360_MOESM13_ESM.zip › EMM-2025-22130_SourceDataForFigure 5A-E 10-28-25/5A/Tsc1-cKIT dKO 90 days H&E 4X.tif]

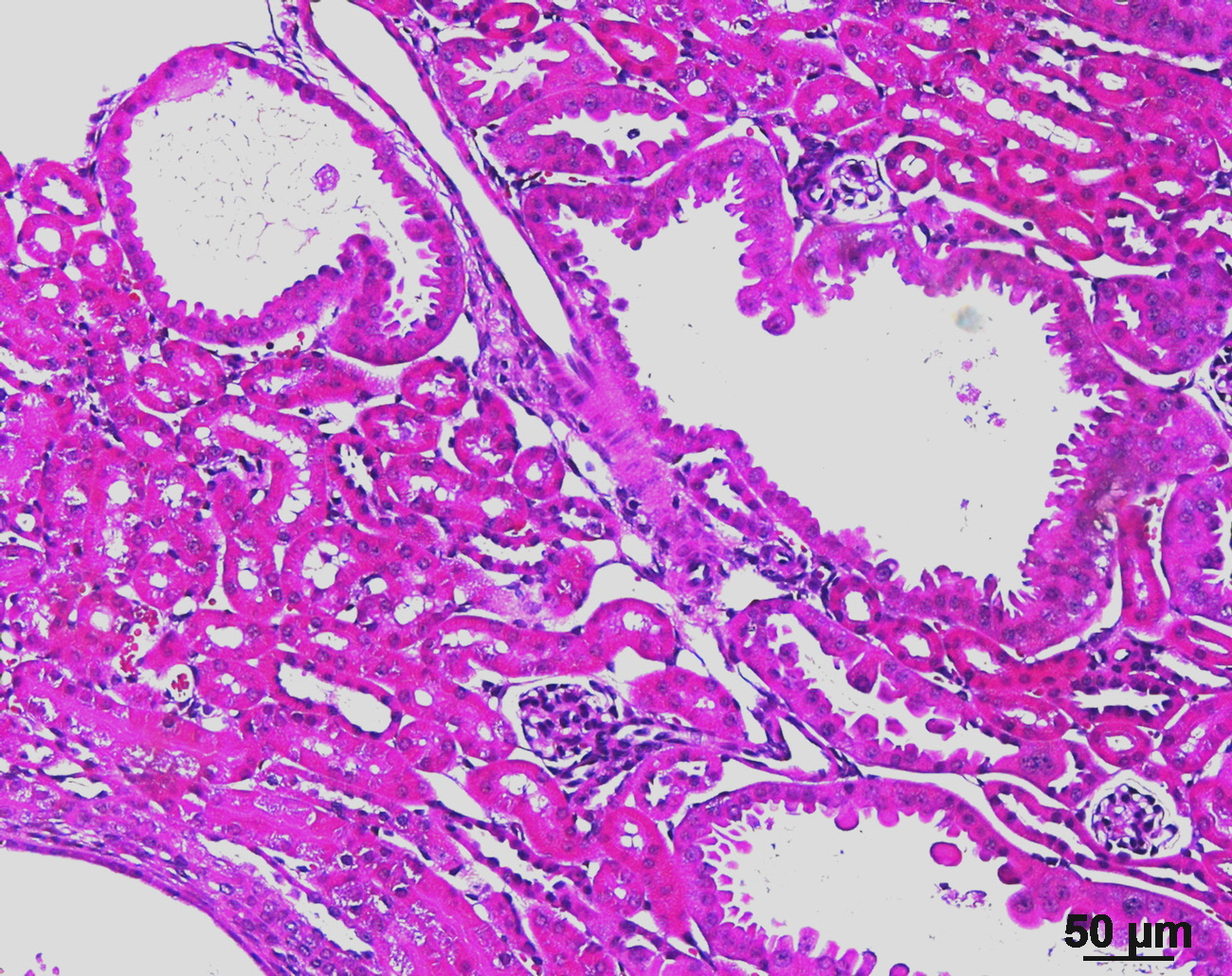

Supplement: Supplementary file 13 — Source data Fig. 5 [file 44321_2025_360_MOESM13_ESM.zip › EMM-2025-22130_SourceDataForFigure 5A-E 10-28-25/5B/Tsc1 KO 45 days H&E 20X.tif]

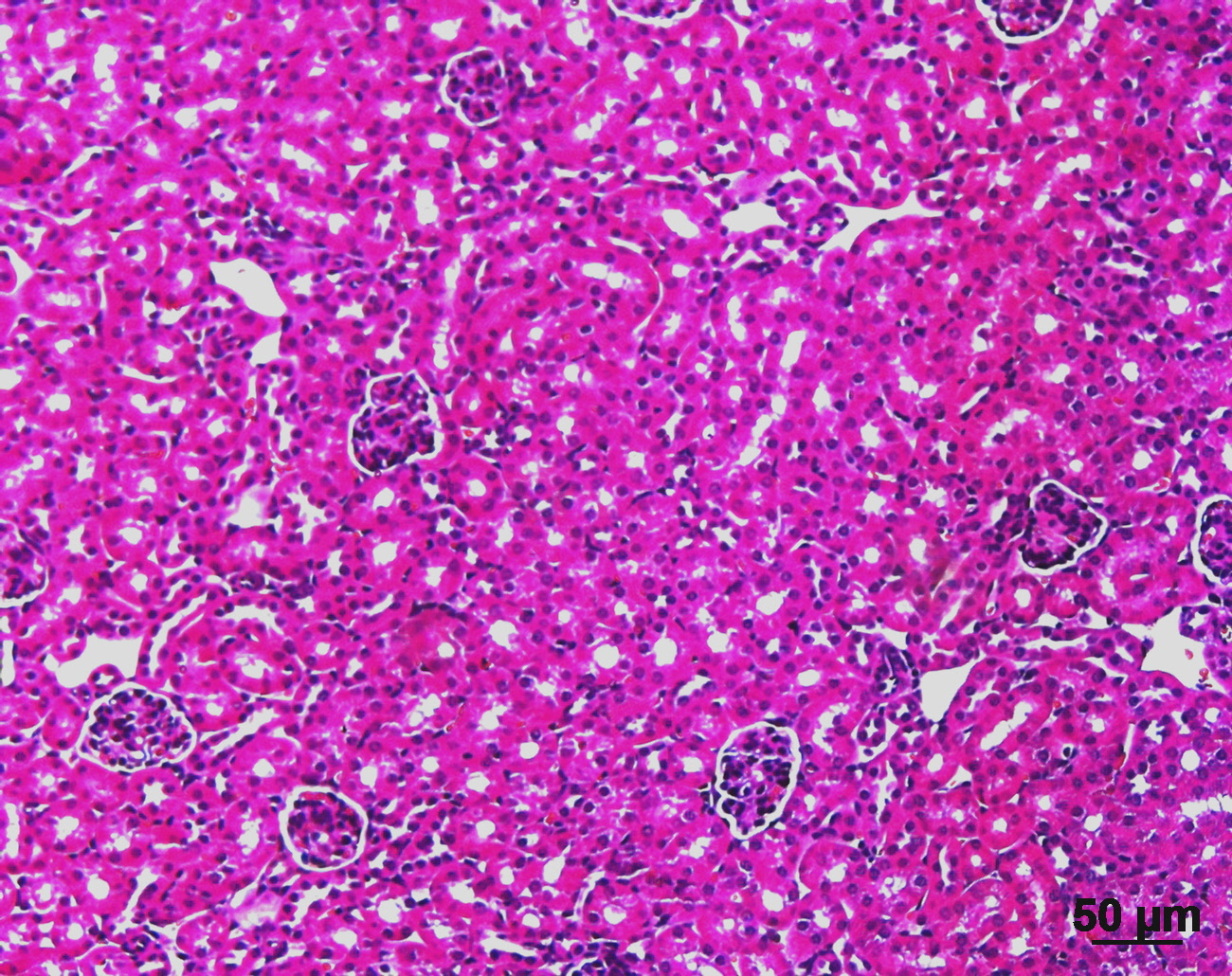

Supplement: Supplementary file 13 — Source data Fig. 5 [file 44321_2025_360_MOESM13_ESM.zip › EMM-2025-22130_SourceDataForFigure 5A-E 10-28-25/5B/Tsc1-cKIT dKO 45 days H&E 20X.tif]

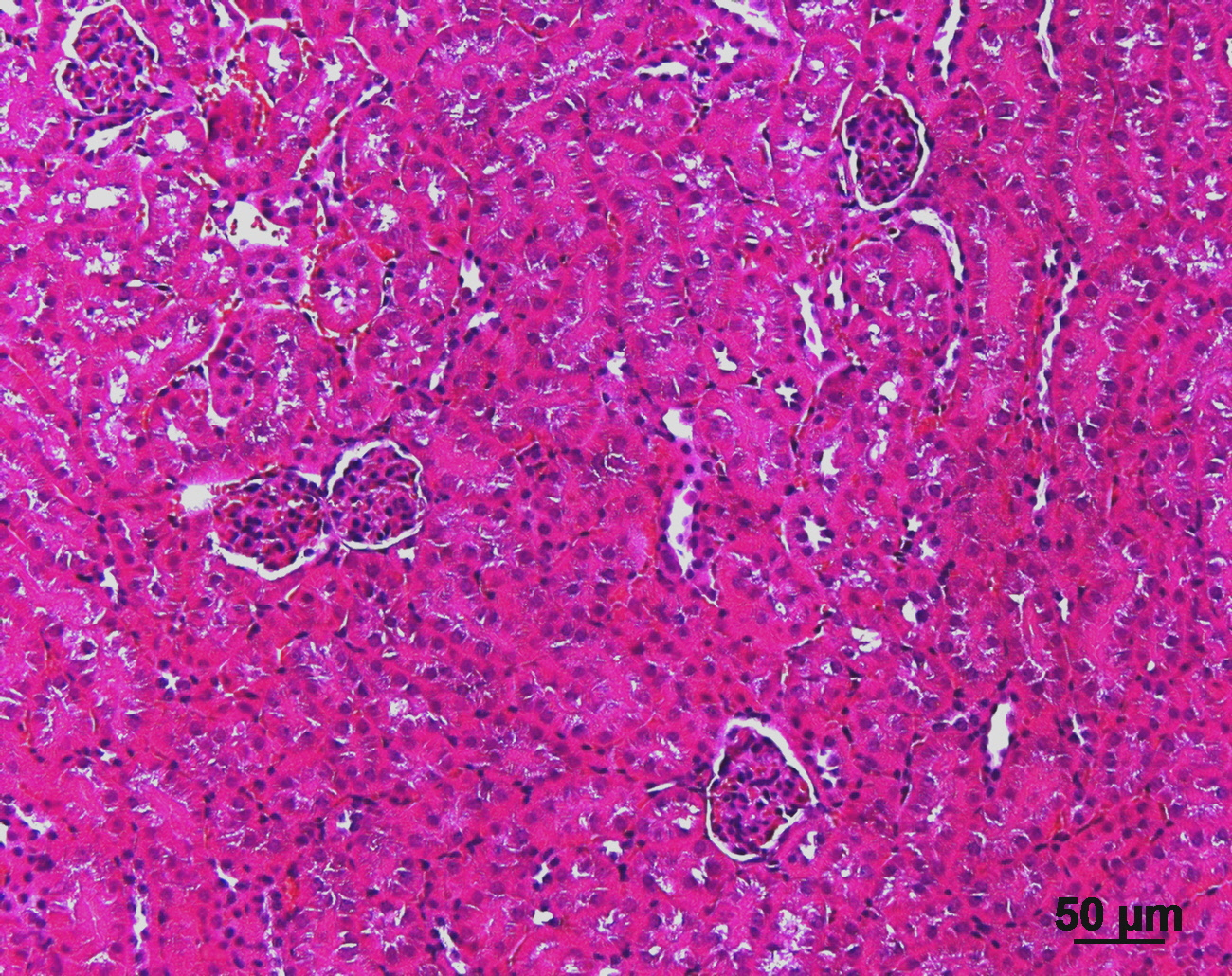

Supplement: Supplementary file 13 — Source data Fig. 5 [file 44321_2025_360_MOESM13_ESM.zip › EMM-2025-22130_SourceDataForFigure 5A-E 10-28-25/5B/Tsc1-cKIT dKO 56 days H&E 20X.tif]

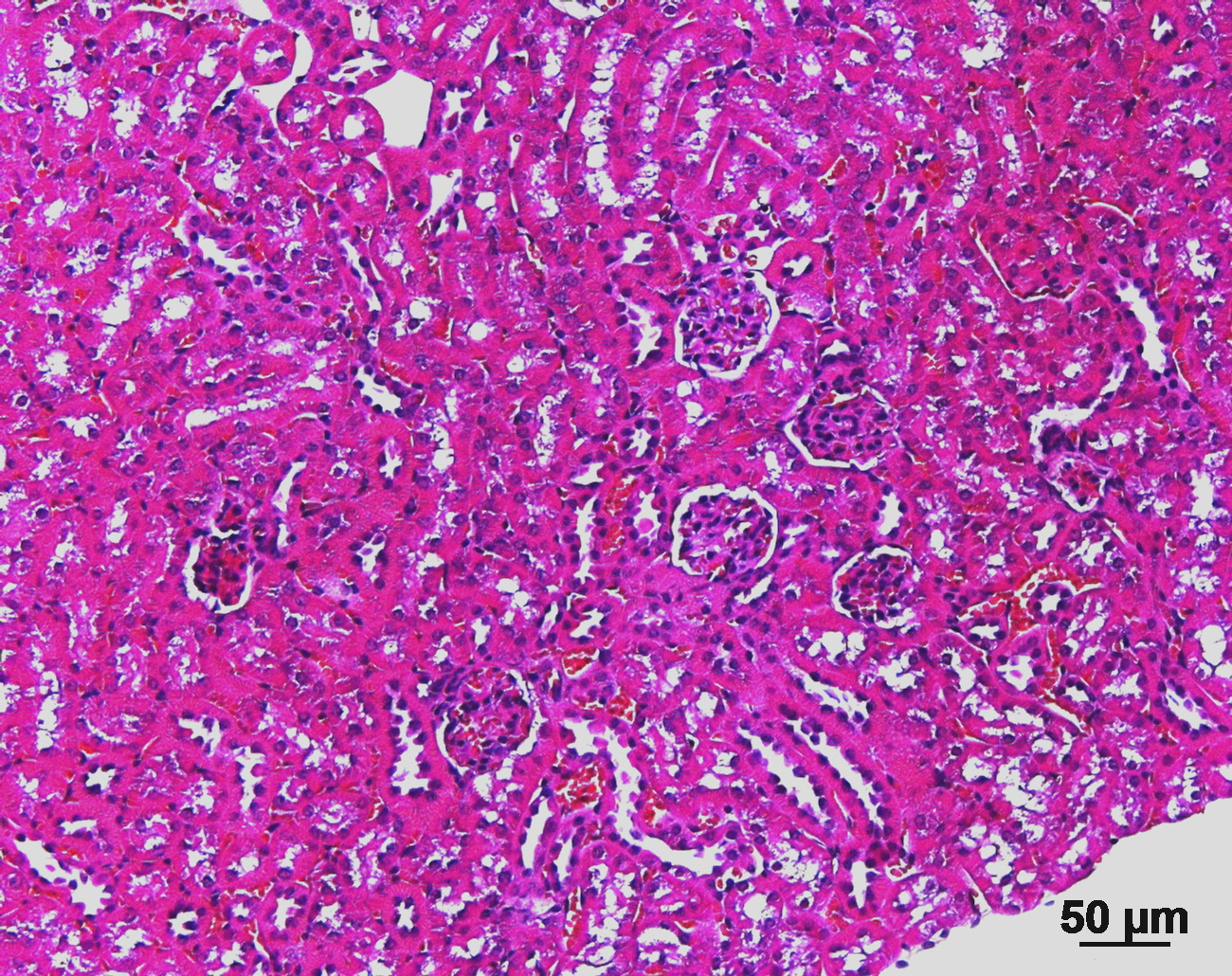

Supplement: Supplementary file 13 — Source data Fig. 5 [file 44321_2025_360_MOESM13_ESM.zip › EMM-2025-22130_SourceDataForFigure 5A-E 10-28-25/5B/Tsc1-cKIT dKO 90 days H&E 20X.tif]

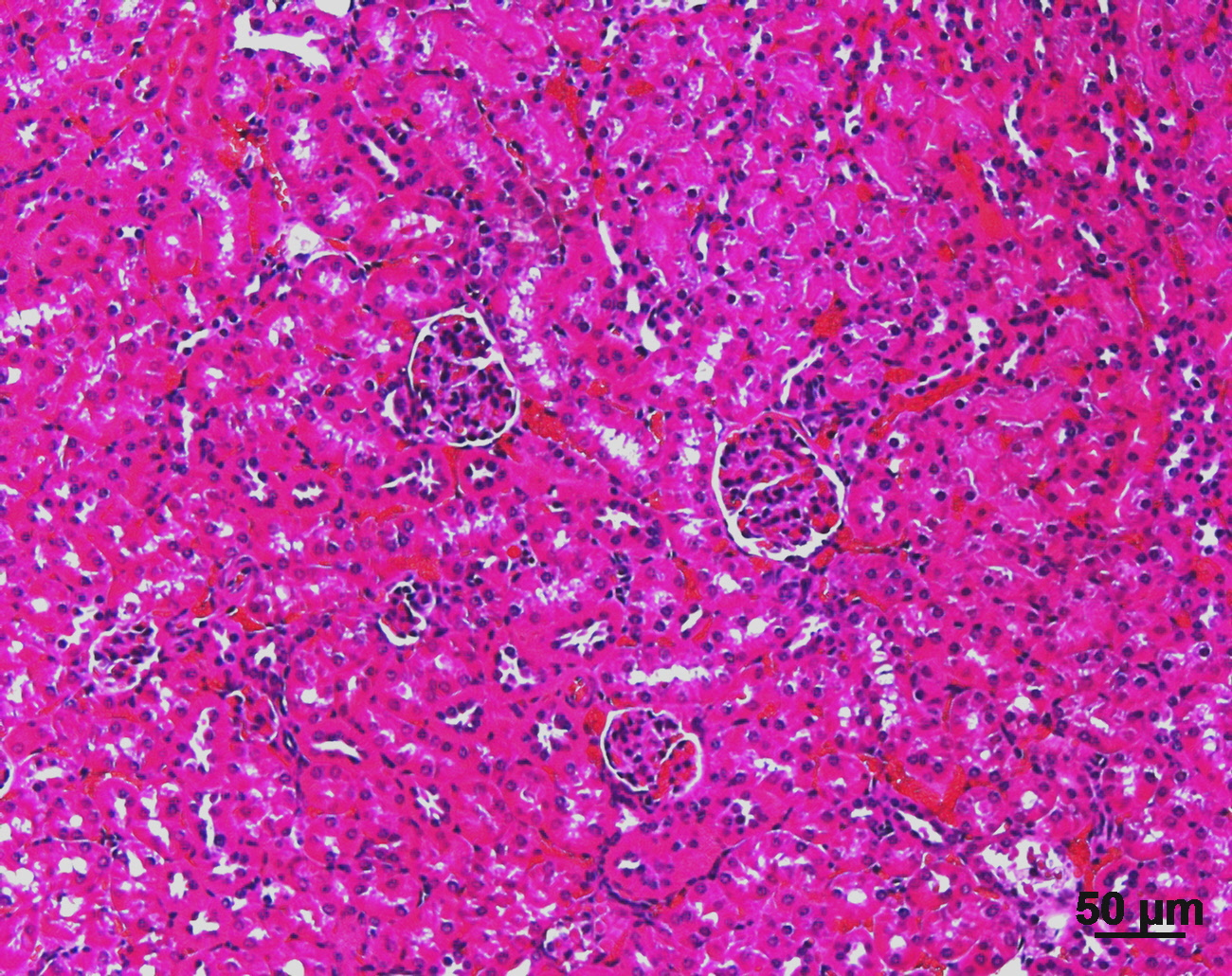

Supplement: Supplementary file 13 — Source data Fig. 5 [file 44321_2025_360_MOESM13_ESM.zip › EMM-2025-22130_SourceDataForFigure 5A-E 10-28-25/5B/WT H&E 20X.tif]

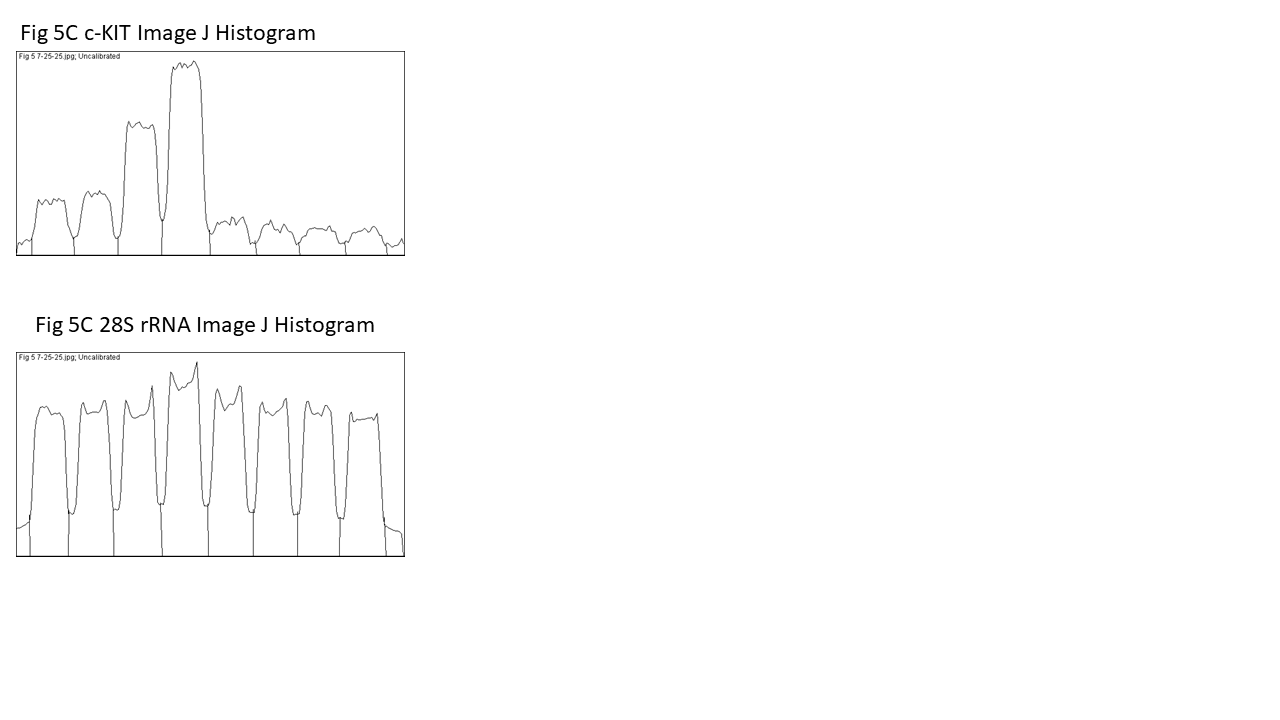

Supplement: Supplementary file 13 — Source data Fig. 5 [file 44321_2025_360_MOESM13_ESM.zip › EMM-2025-22130_SourceDataForFigure 5A-E 10-28-25/5C/Northern Blot Image J Histograms.tif]

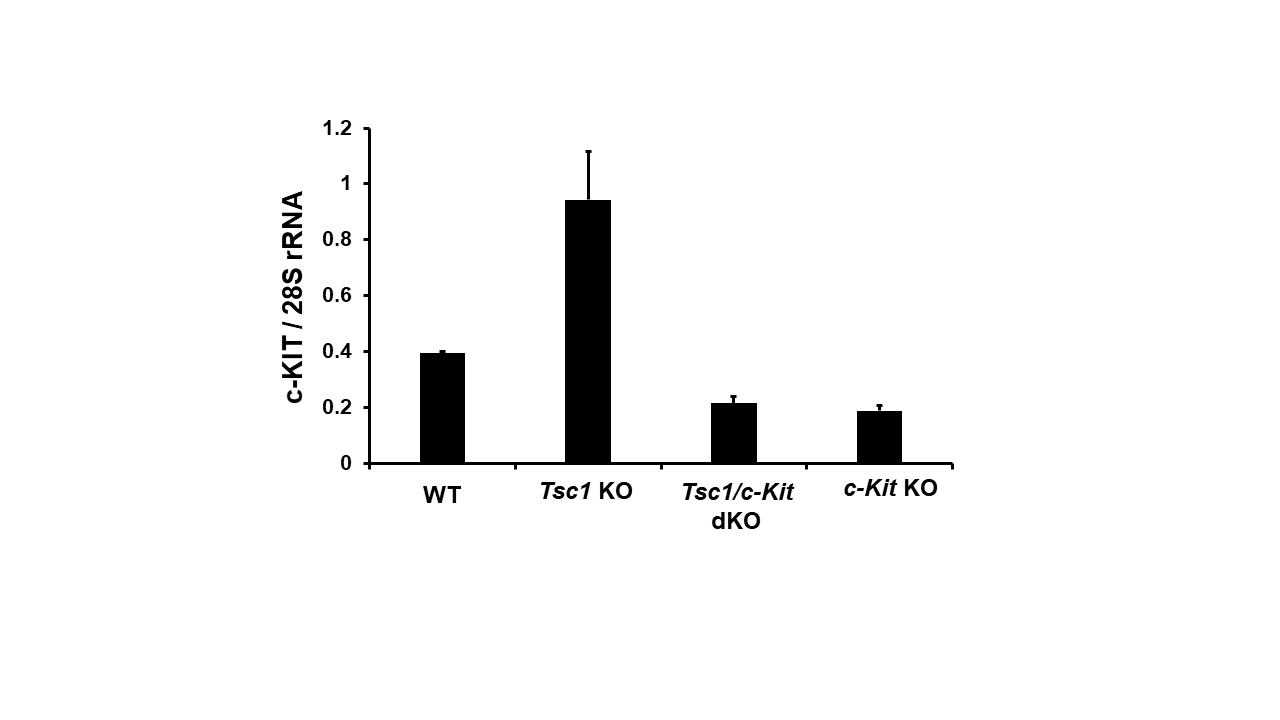

Supplement: Supplementary file 13 — Source data Fig. 5 [file 44321_2025_360_MOESM13_ESM.zip › EMM-2025-22130_SourceDataForFigure 5A-E 10-28-25/5C/Northern Blot Quantification.tif]

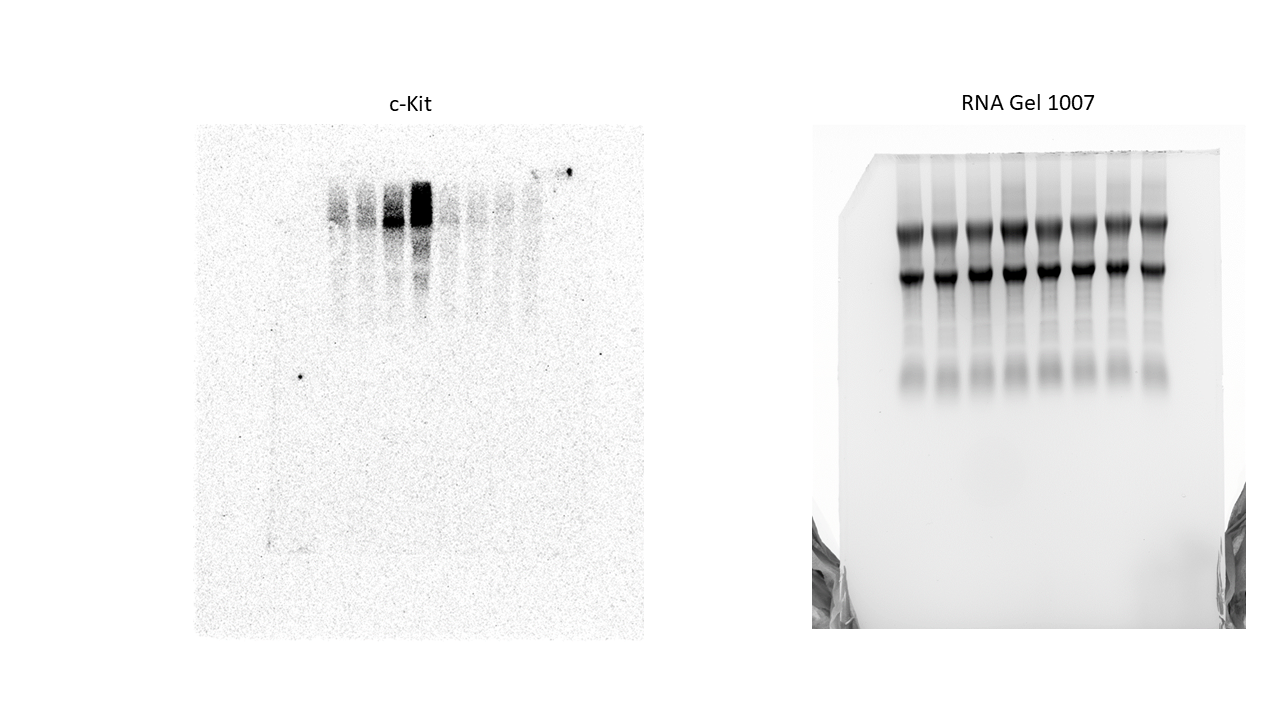

Supplement: Supplementary file 13 — Source data Fig. 5 [file 44321_2025_360_MOESM13_ESM.zip › EMM-2025-22130_SourceDataForFigure 5A-E 10-28-25/5C/Northern Blot Source Data.tif]

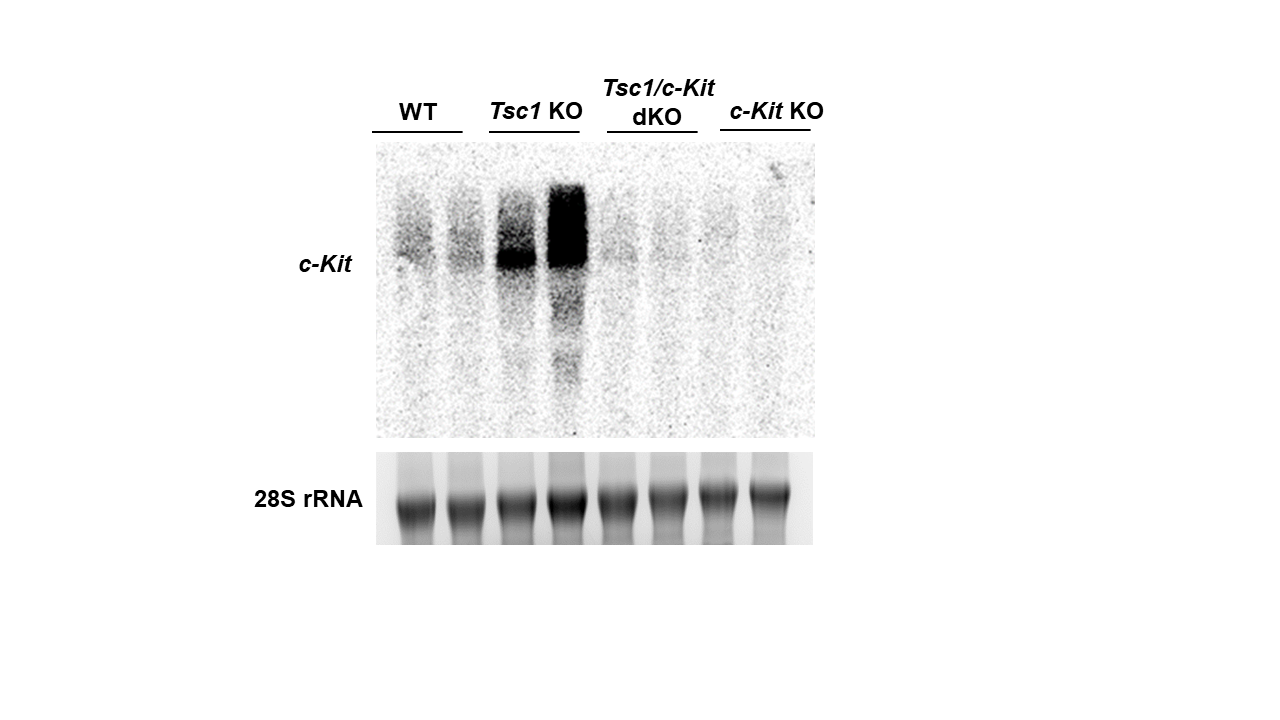

Supplement: Supplementary file 13 — Source data Fig. 5 [file 44321_2025_360_MOESM13_ESM.zip › EMM-2025-22130_SourceDataForFigure 5A-E 10-28-25/5C/Northern Blot.tif]

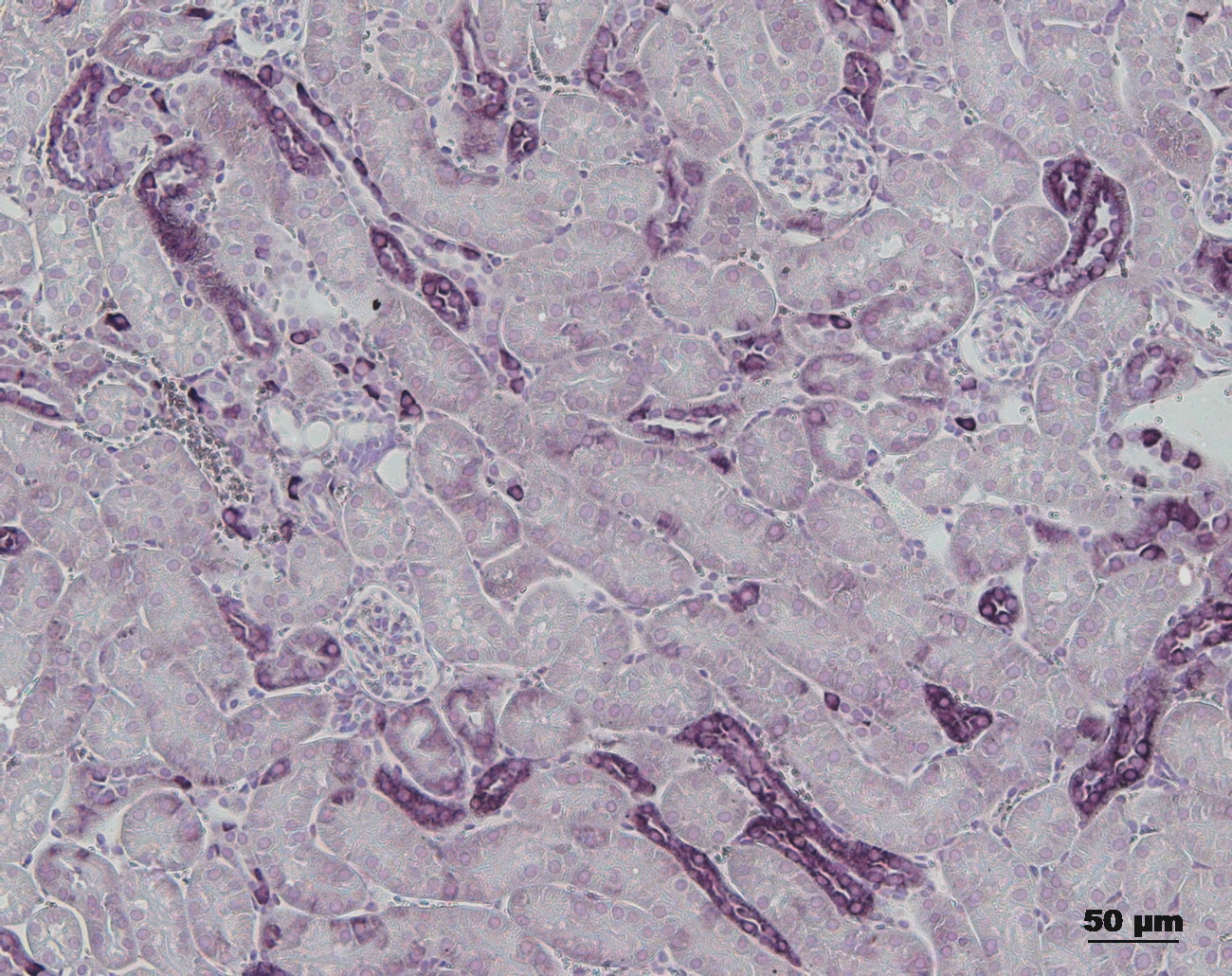

Supplement: Supplementary file 13 — Source data Fig. 5 [file 44321_2025_360_MOESM13_ESM.zip › EMM-2025-22130_SourceDataForFigure 5A-E 10-28-25/5D/cKIT KO pS6 20X.tif]

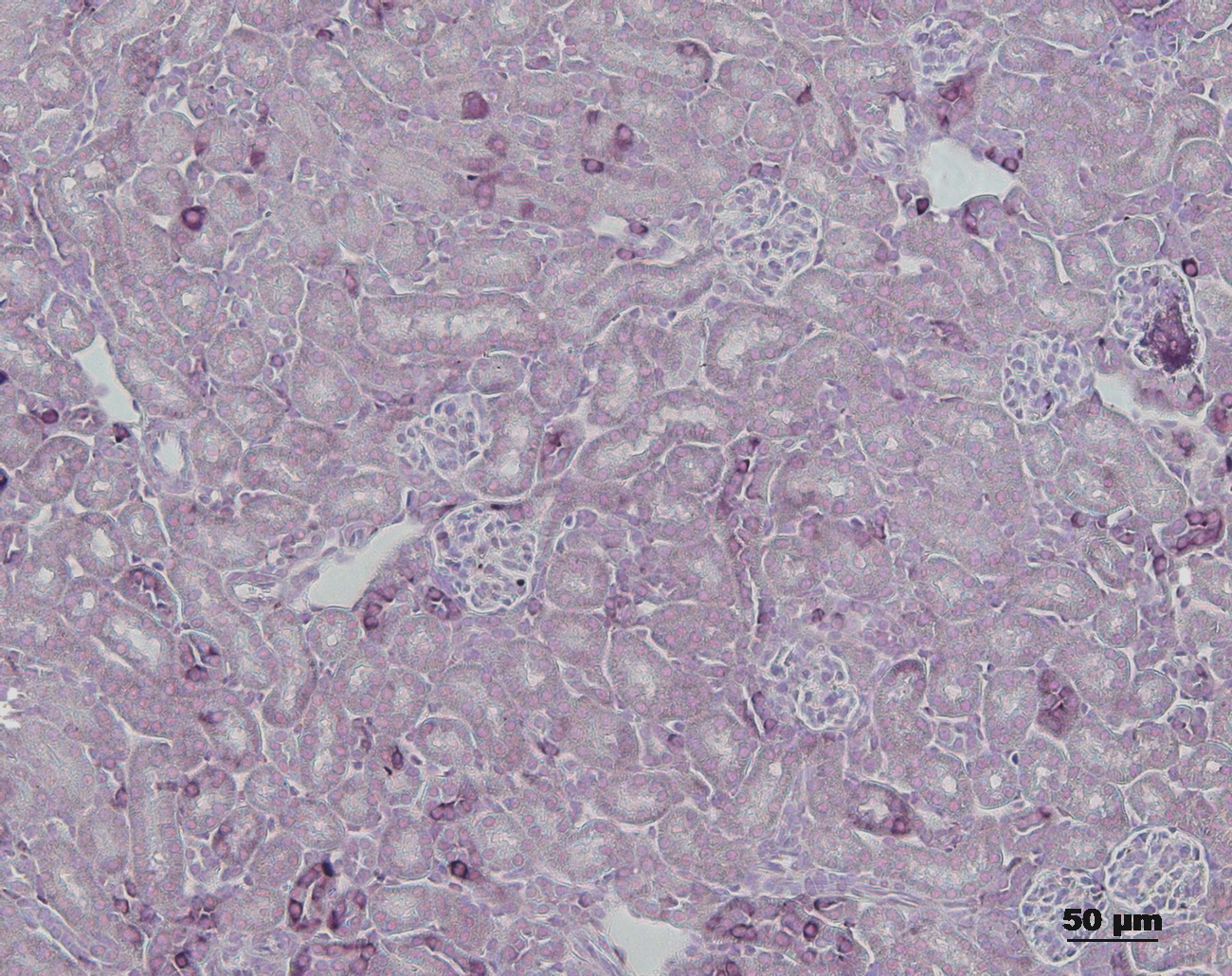

Supplement: Supplementary file 13 — Source data Fig. 5 [file 44321_2025_360_MOESM13_ESM.zip › EMM-2025-22130_SourceDataForFigure 5A-E 10-28-25/5D/Tsc1 cKIT dKO pS6 20X.tif]

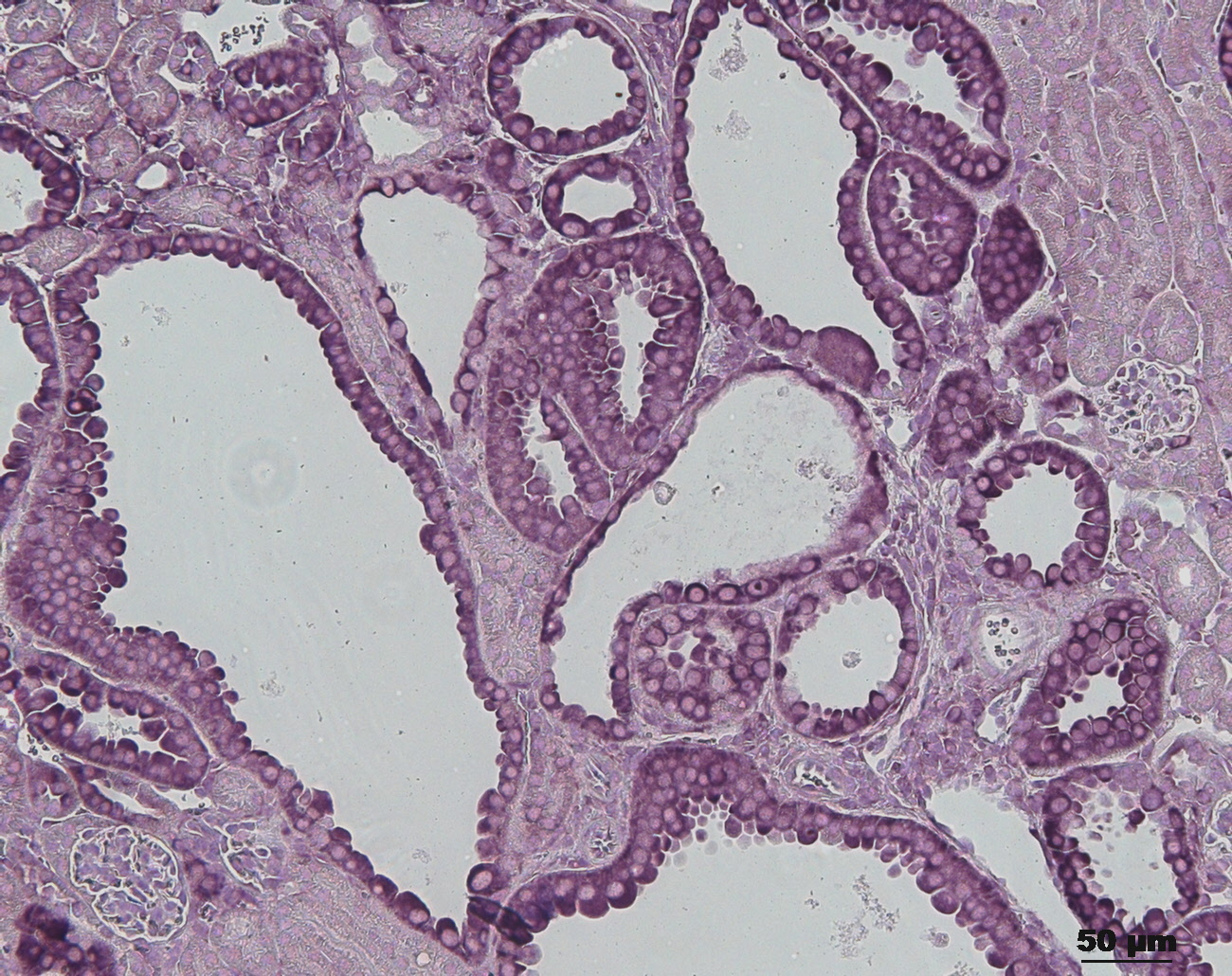

Supplement: Supplementary file 13 — Source data Fig. 5 [file 44321_2025_360_MOESM13_ESM.zip › EMM-2025-22130_SourceDataForFigure 5A-E 10-28-25/5D/Tsc1 KO pS6 20X.tif]

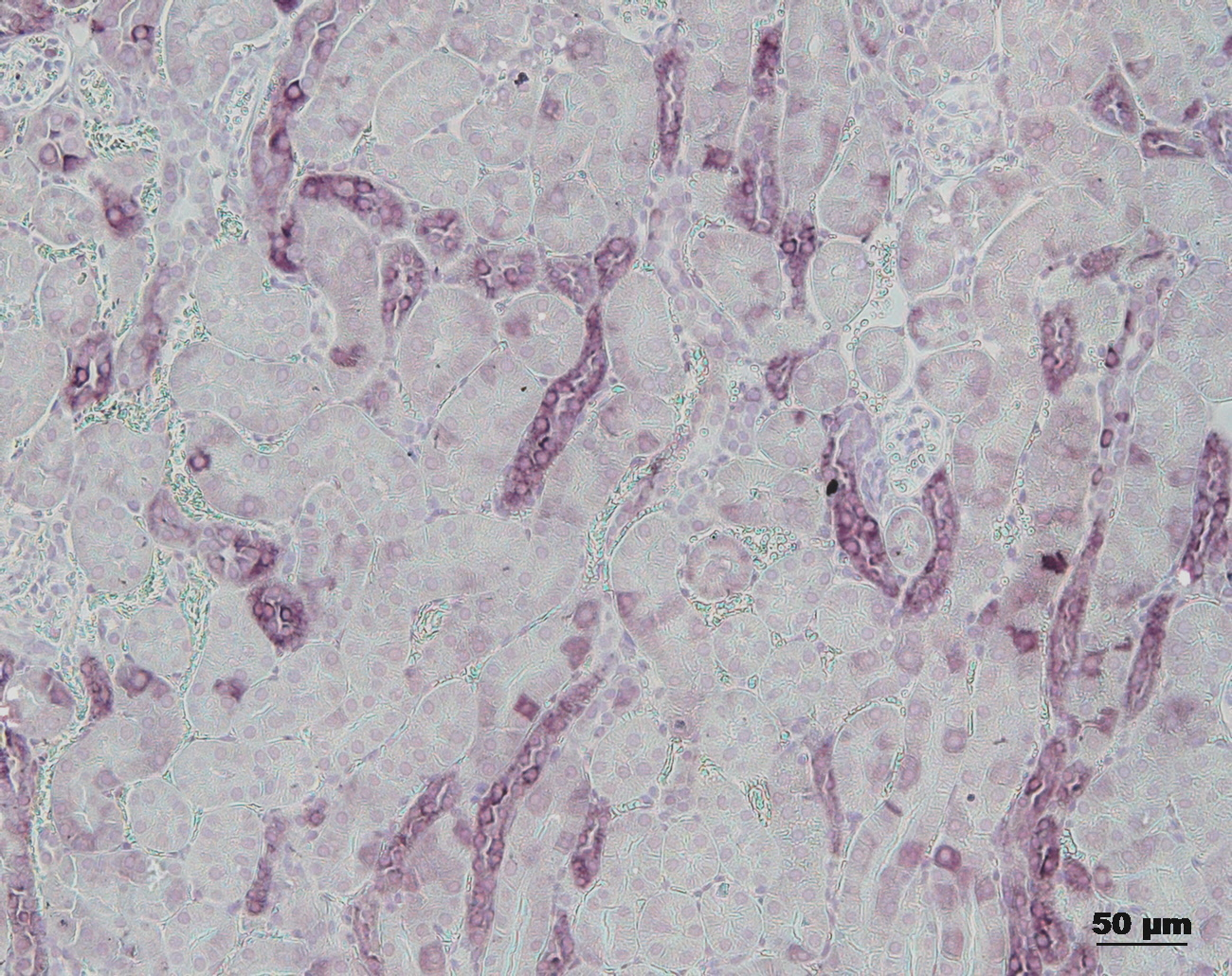

Supplement: Supplementary file 13 — Source data Fig. 5 [file 44321_2025_360_MOESM13_ESM.zip › EMM-2025-22130_SourceDataForFigure 5A-E 10-28-25/5D/WT pS6 20X.tif]

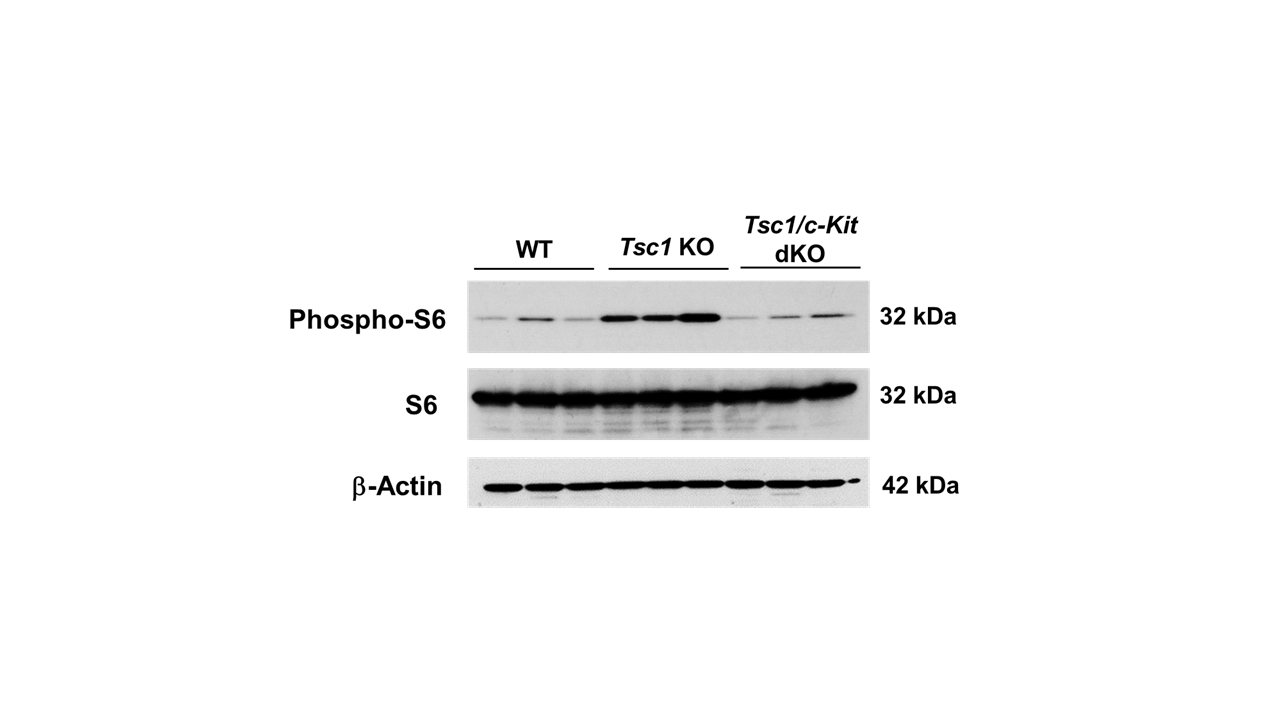

Supplement: Supplementary file 13 — Source data Fig. 5 [file 44321_2025_360_MOESM13_ESM.zip › EMM-2025-22130_SourceDataForFigure 5A-E 10-28-25/5E/Western Blot 10-29-25.tif]

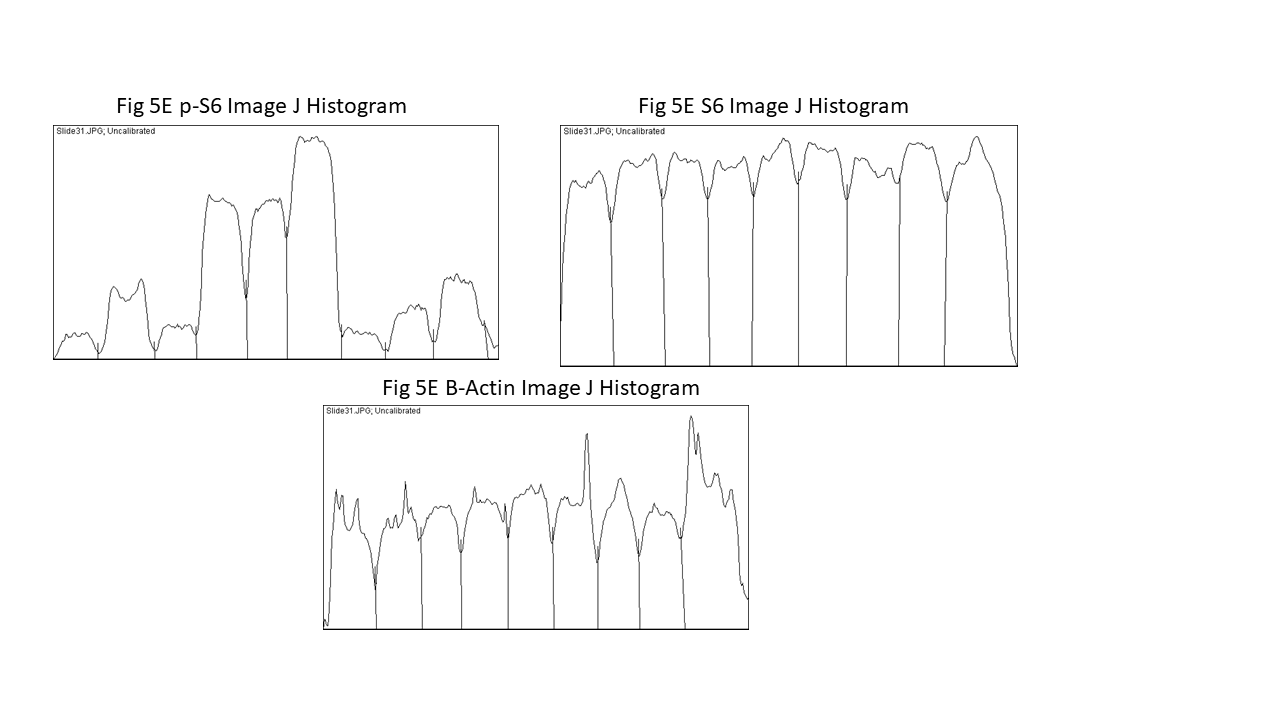

Supplement: Supplementary file 13 — Source data Fig. 5 [file 44321_2025_360_MOESM13_ESM.zip › EMM-2025-22130_SourceDataForFigure 5A-E 10-28-25/5E/Western Blot Image J Histograms.tif]

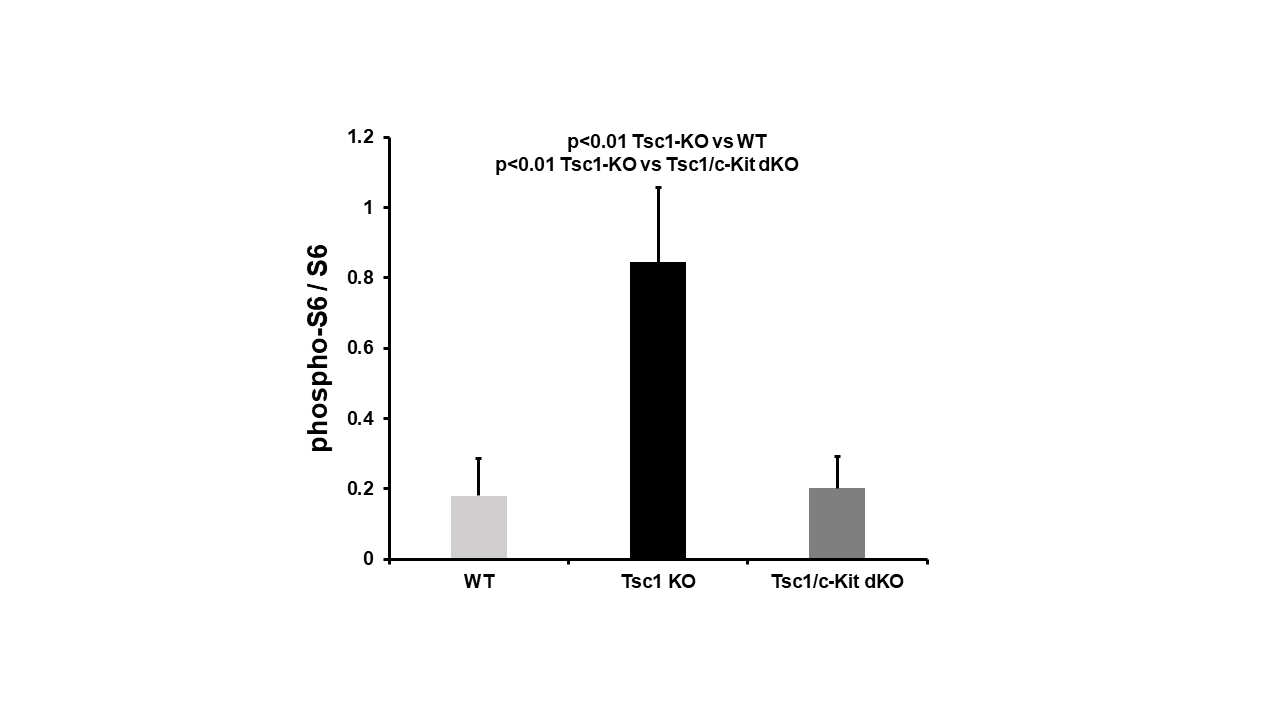

Supplement: Supplementary file 13 — Source data Fig. 5 [file 44321_2025_360_MOESM13_ESM.zip › EMM-2025-22130_SourceDataForFigure 5A-E 10-28-25/5E/Western Blot Quantification.tif]

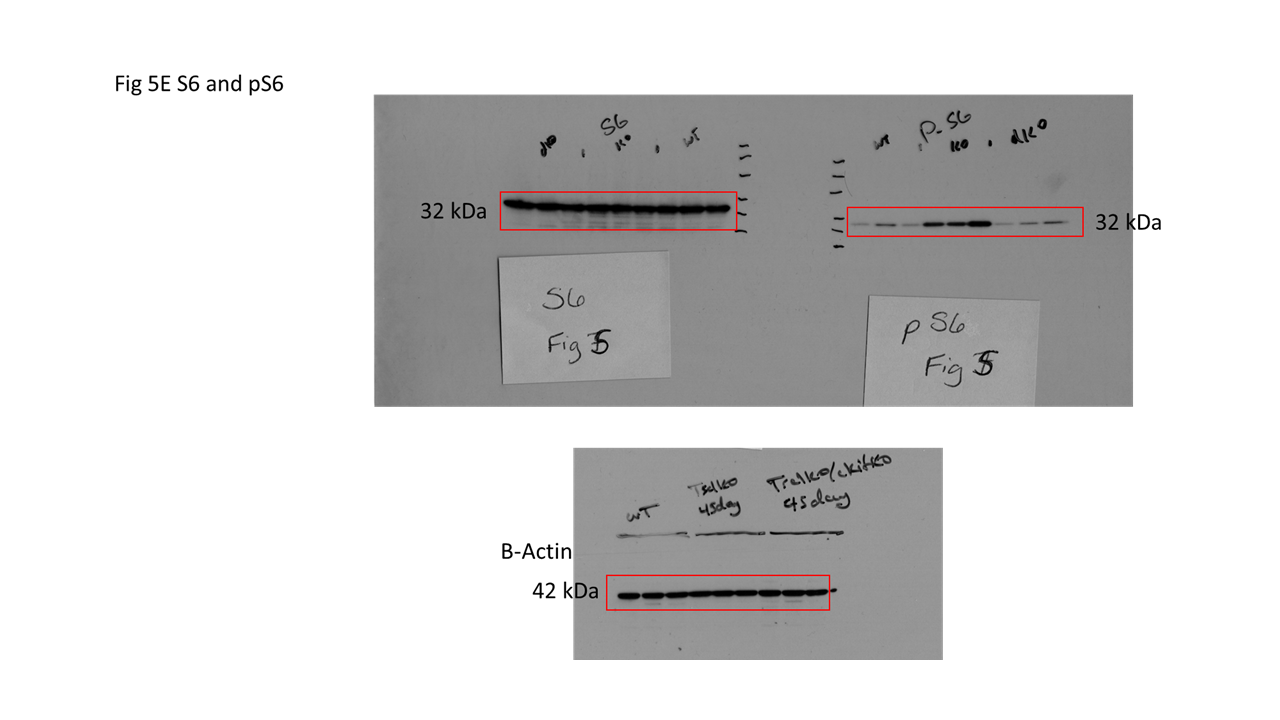

Supplement: Supplementary file 13 — Source data Fig. 5 [file 44321_2025_360_MOESM13_ESM.zip › EMM-2025-22130_SourceDataForFigure 5A-E 10-28-25/5E/Western Source Data 10-29-25.tif]

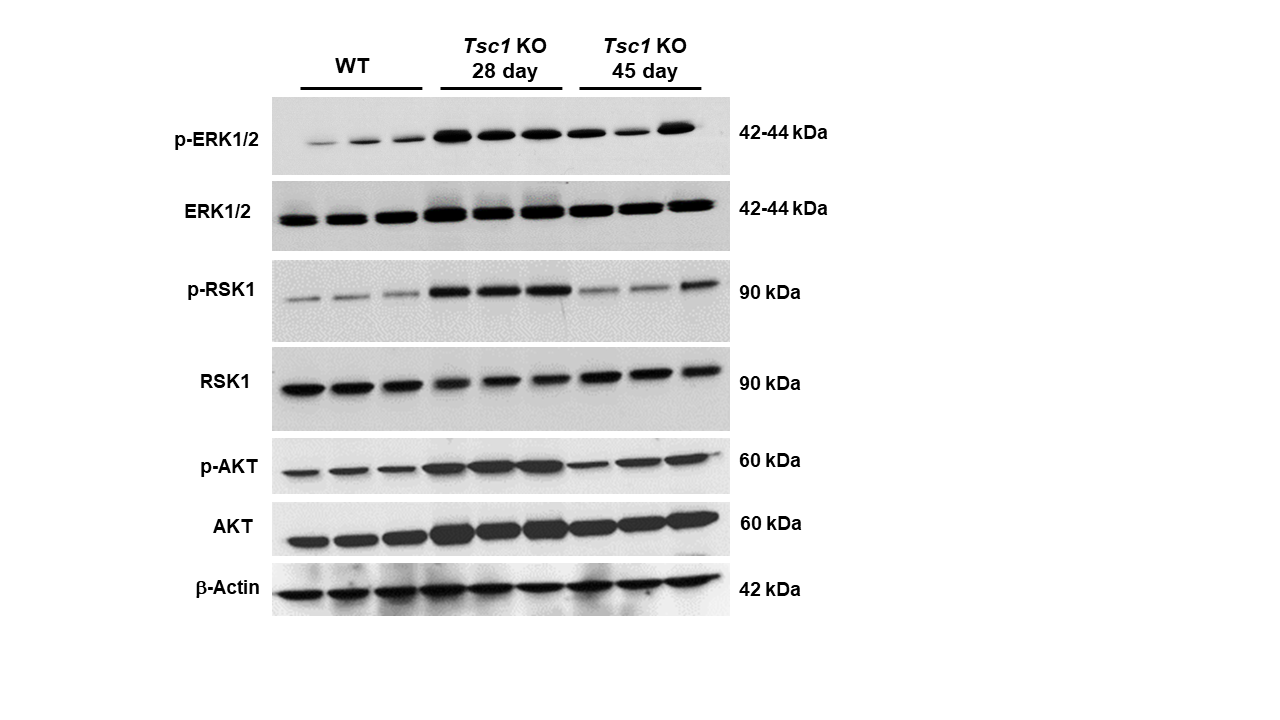

Supplement: Supplementary file 14 — Source data Fig. 6 [file 44321_2025_360_MOESM14_ESM.zip › EMM-2025-22130_SourceDataForFigure 6A-C 10-28-25/6A/Fig 6A Western Blot 10-29-25.tif]

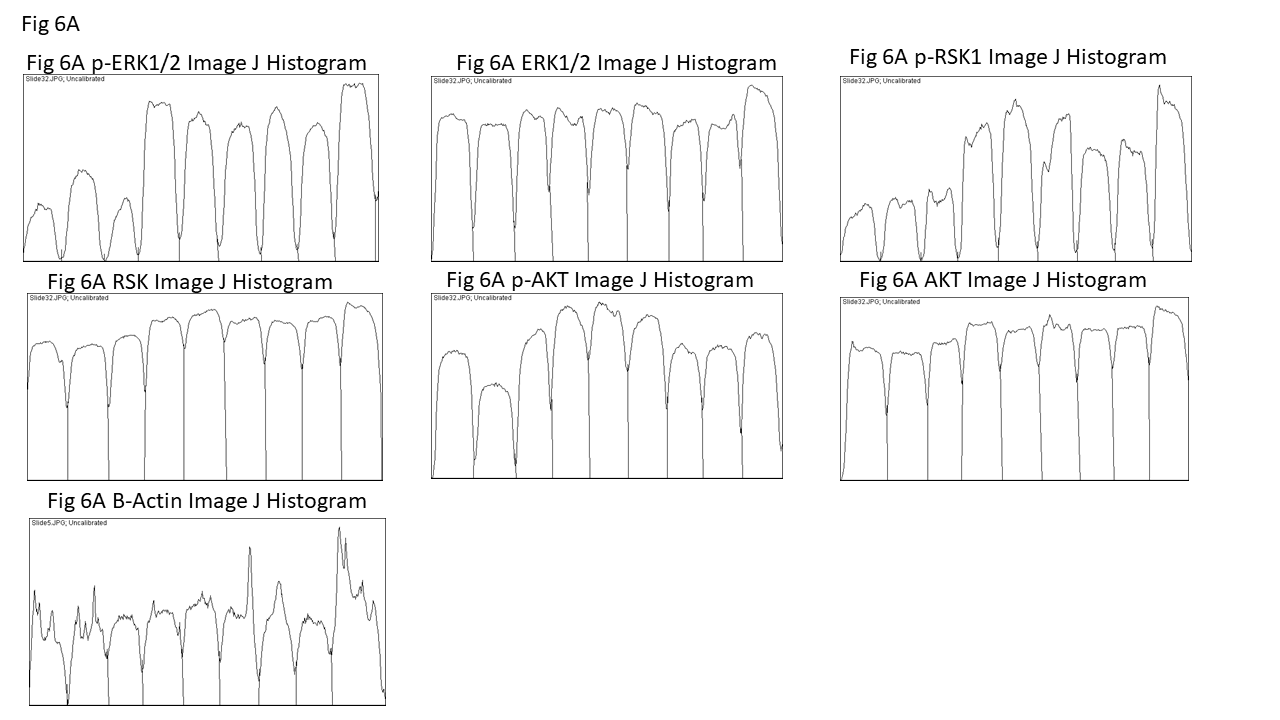

Supplement: Supplementary file 14 — Source data Fig. 6 [file 44321_2025_360_MOESM14_ESM.zip › EMM-2025-22130_SourceDataForFigure 6A-C 10-28-25/6A/Western Blot Image J Histograms/Slide1.TIF]

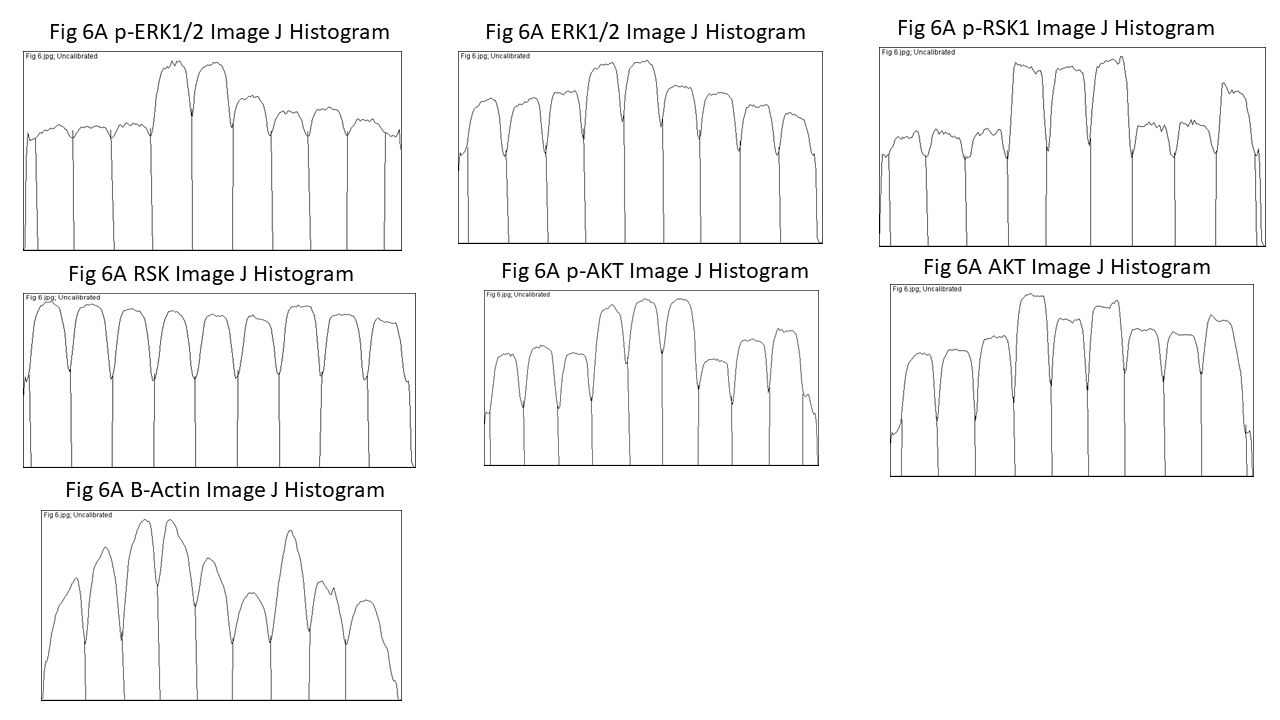

Supplement: Supplementary file 14 — Source data Fig. 6 [file 44321_2025_360_MOESM14_ESM.zip › EMM-2025-22130_SourceDataForFigure 6A-C 10-28-25/6A/Western Blot Image J Histograms/Slide2.TIF]

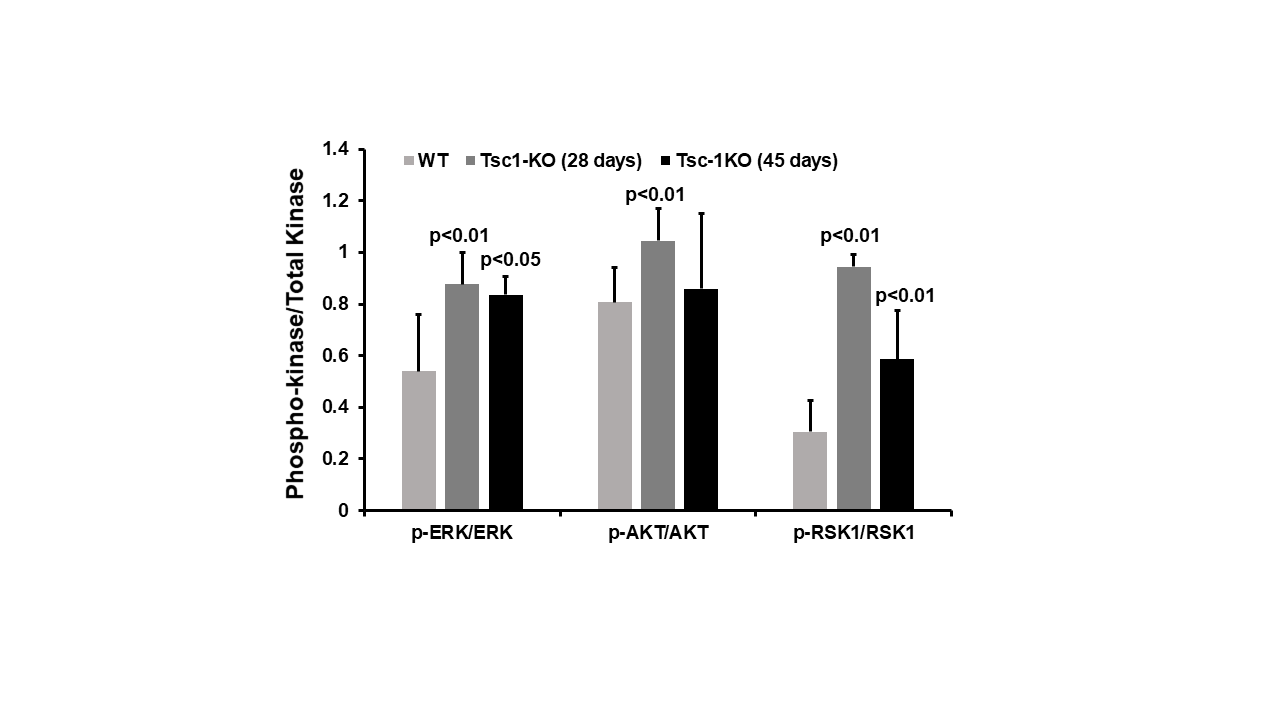

Supplement: Supplementary file 14 — Source data Fig. 6 [file 44321_2025_360_MOESM14_ESM.zip › EMM-2025-22130_SourceDataForFigure 6A-C 10-28-25/6A/Western Blot Quantification.tif]

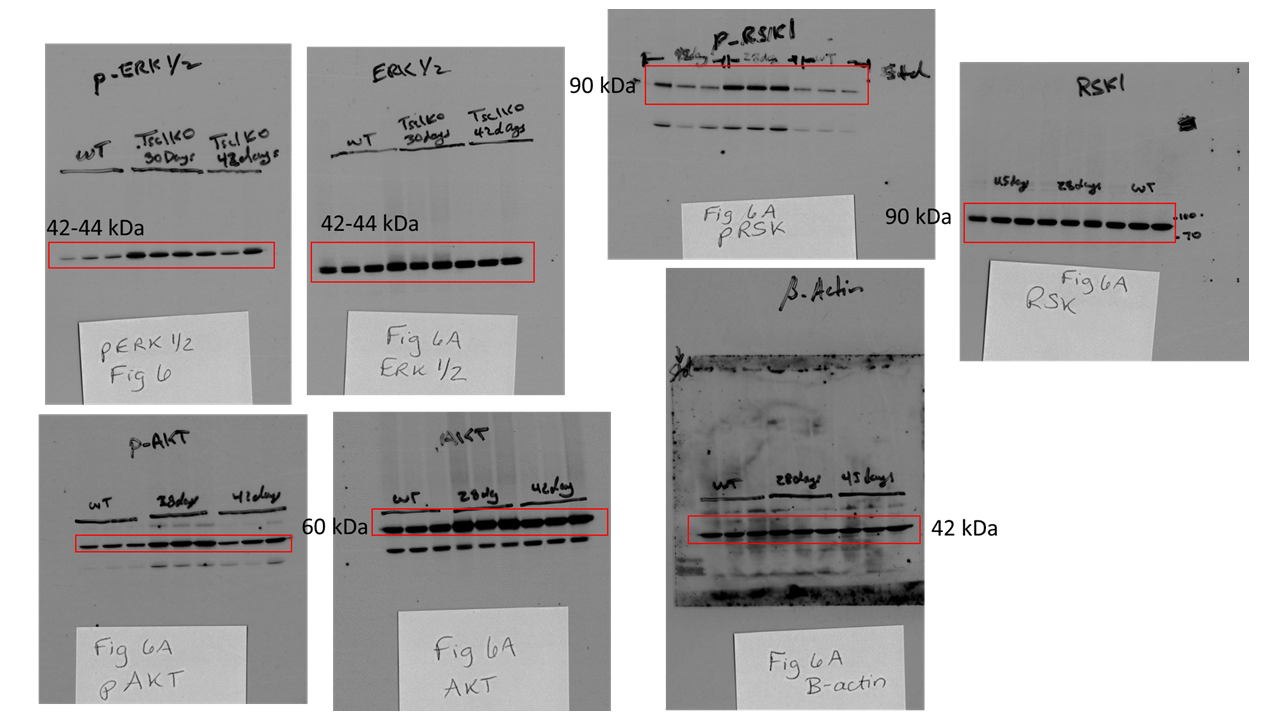

Supplement: Supplementary file 14 — Source data Fig. 6 [file 44321_2025_360_MOESM14_ESM.zip › EMM-2025-22130_SourceDataForFigure 6A-C 10-28-25/6A/Western Source Data 10-28-25/Slide1.TIF]

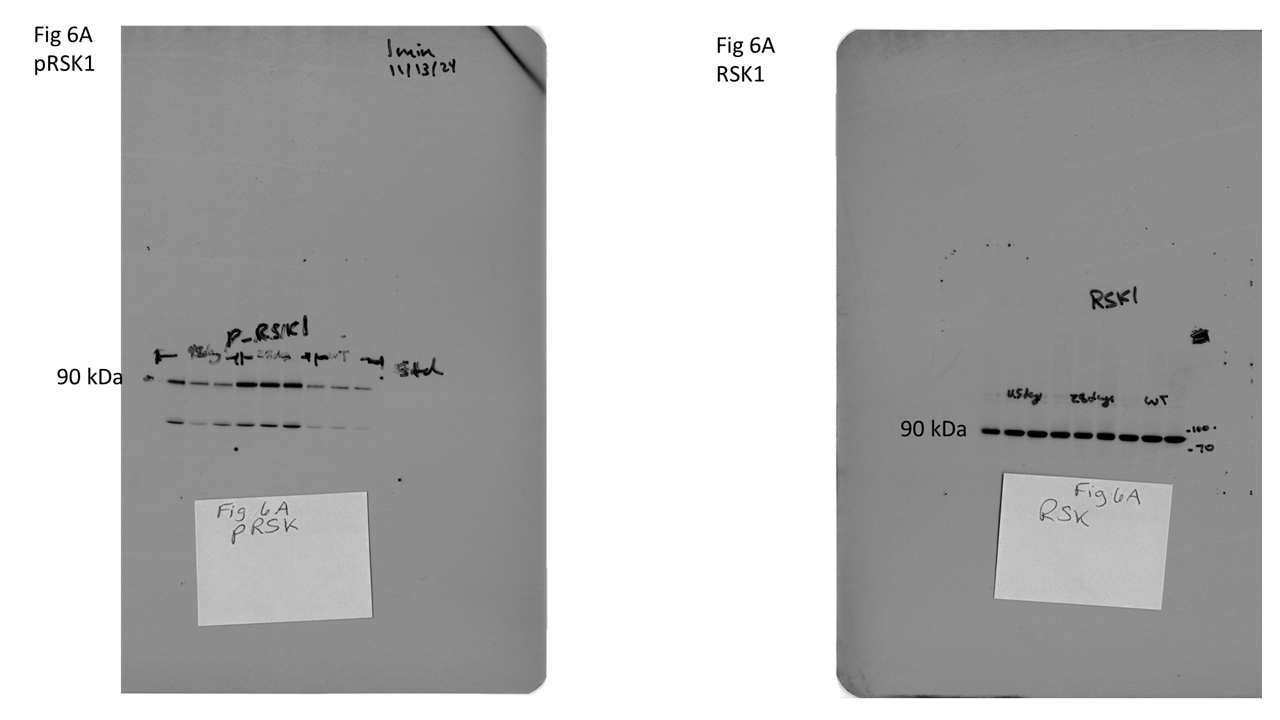

Supplement: Supplementary file 14 — Source data Fig. 6 [file 44321_2025_360_MOESM14_ESM.zip › EMM-2025-22130_SourceDataForFigure 6A-C 10-28-25/6A/Western Source Data 10-28-25/Slide2.TIF]

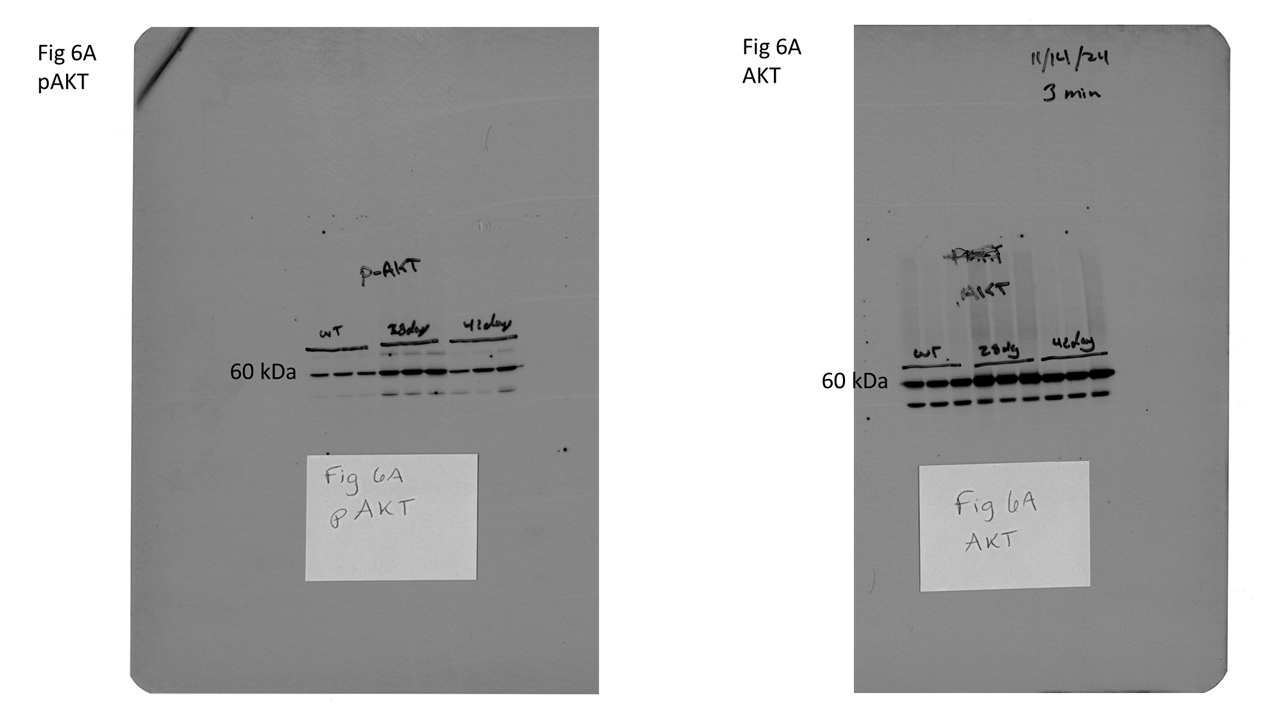

Supplement: Supplementary file 14 — Source data Fig. 6 [file 44321_2025_360_MOESM14_ESM.zip › EMM-2025-22130_SourceDataForFigure 6A-C 10-28-25/6A/Western Source Data 10-28-25/Slide3.TIF]

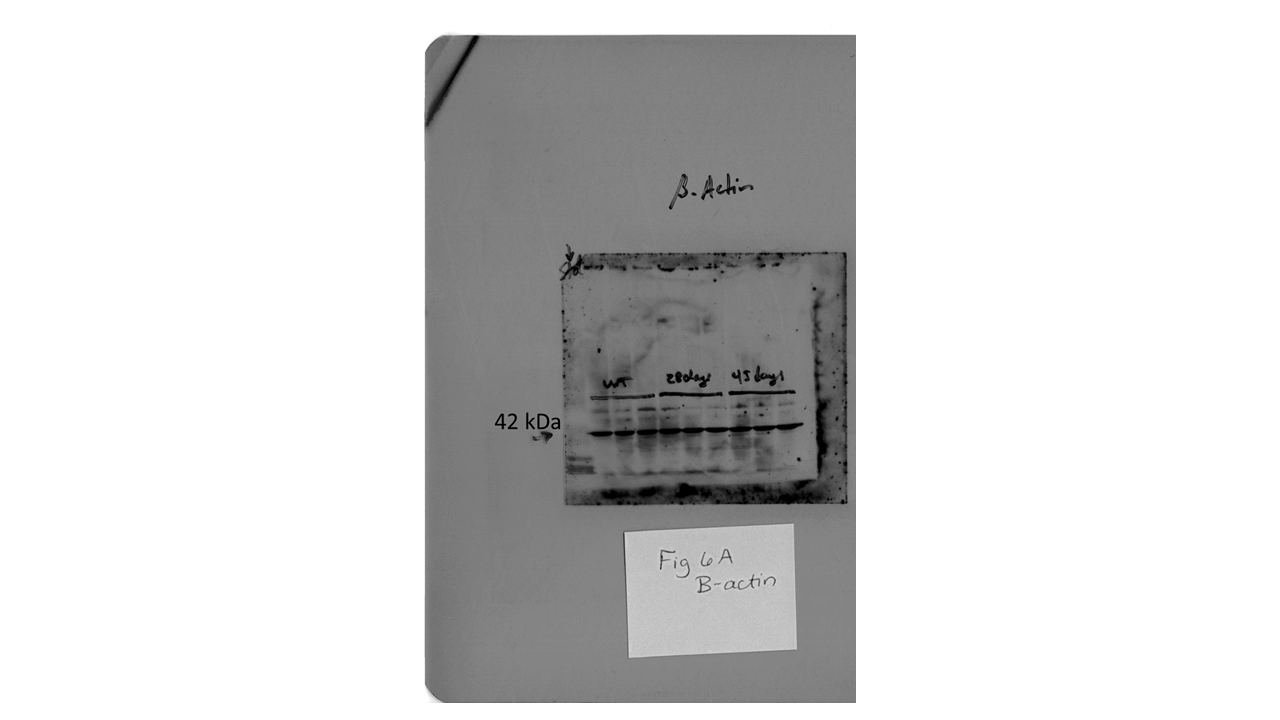

Supplement: Supplementary file 14 — Source data Fig. 6 [file 44321_2025_360_MOESM14_ESM.zip › EMM-2025-22130_SourceDataForFigure 6A-C 10-28-25/6A/Western Source Data 10-28-25/Slide4.TIF]

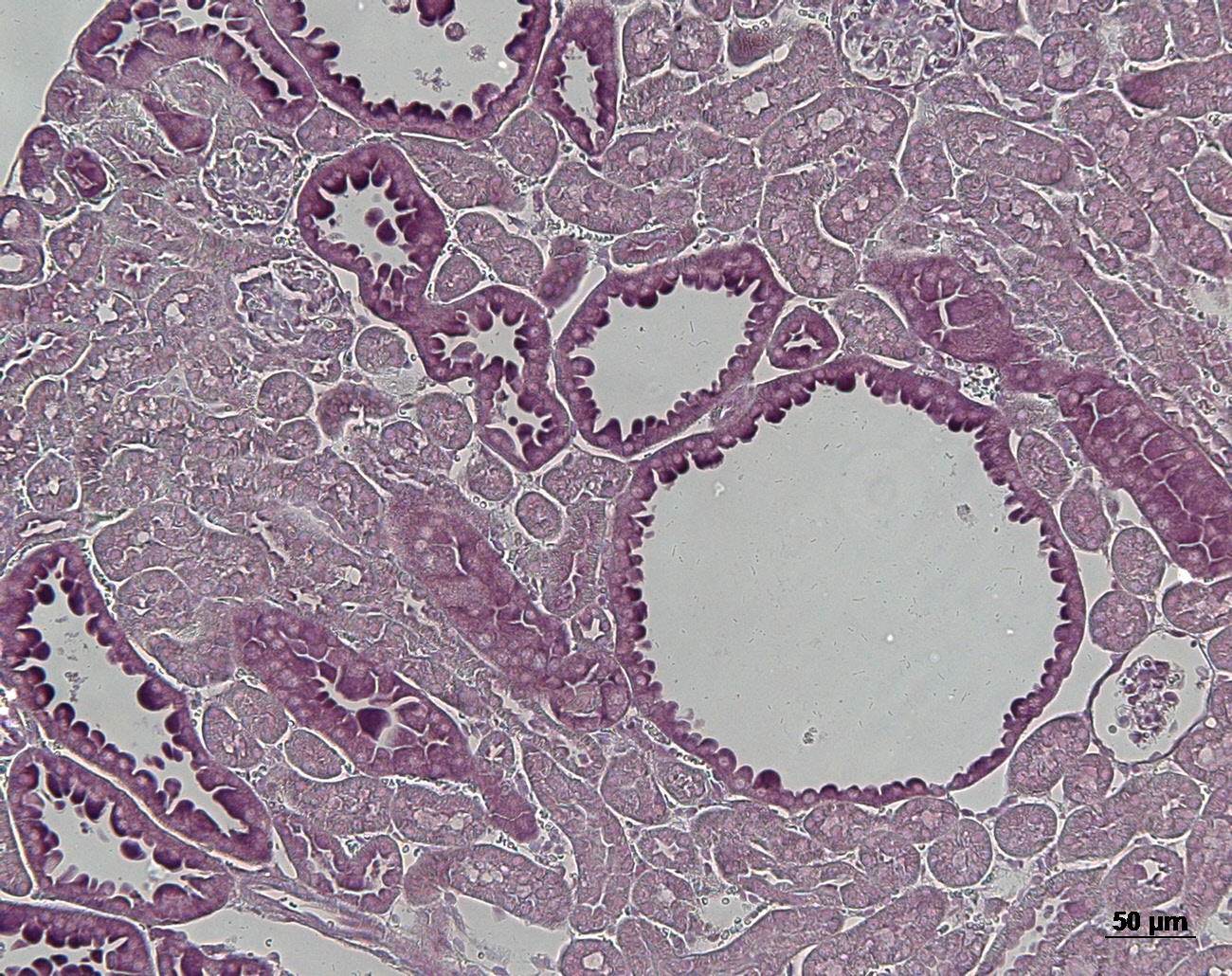

Supplement: Supplementary file 14 — Source data Fig. 6 [file 44321_2025_360_MOESM14_ESM.zip › EMM-2025-22130_SourceDataForFigure 6A-C 10-28-25/6B/Tsc1 KO pAKT 20X.tif]

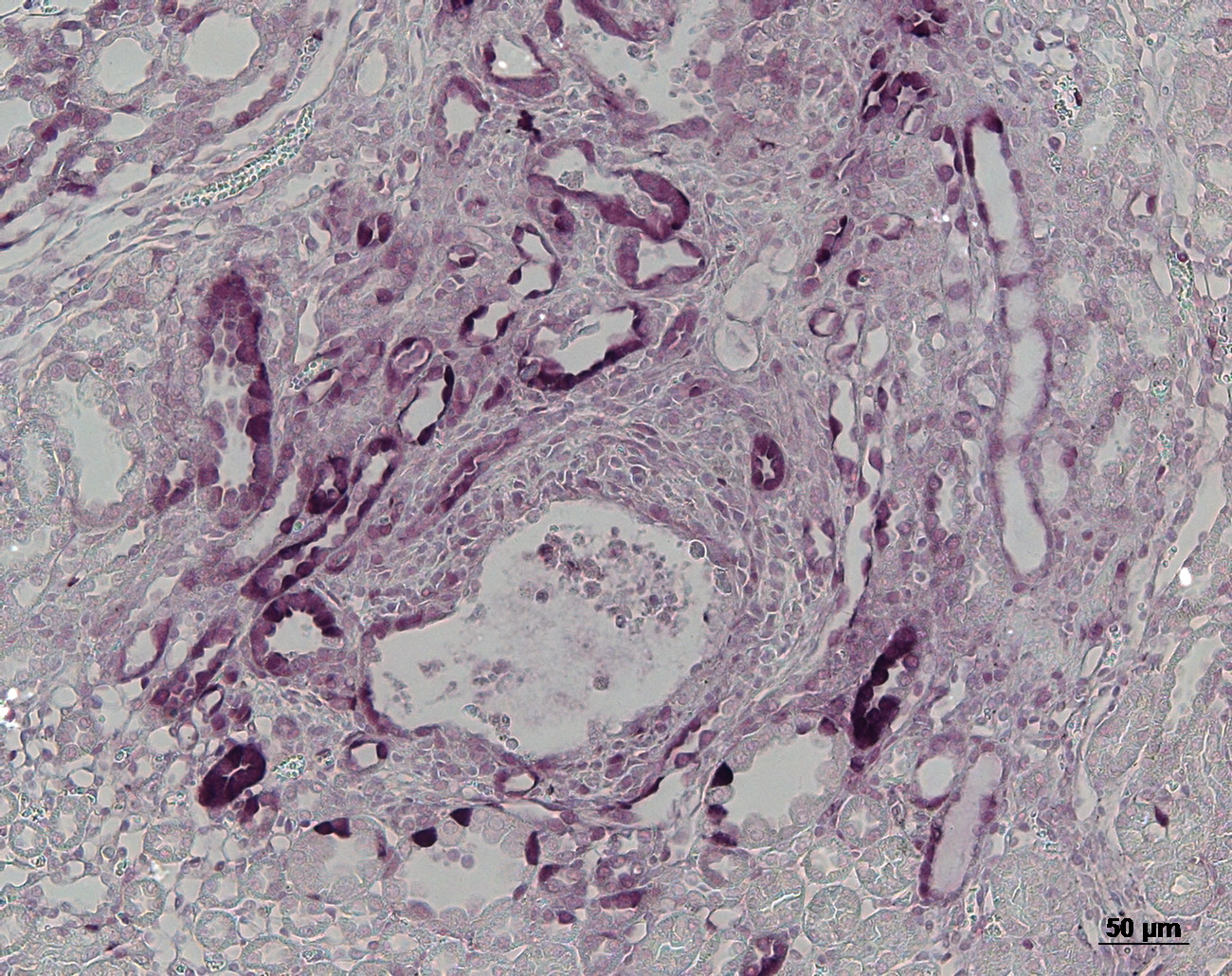

Supplement: Supplementary file 14 — Source data Fig. 6 [file 44321_2025_360_MOESM14_ESM.zip › EMM-2025-22130_SourceDataForFigure 6A-C 10-28-25/6B/Tsc1 KO pERK1-2 20X.tif]

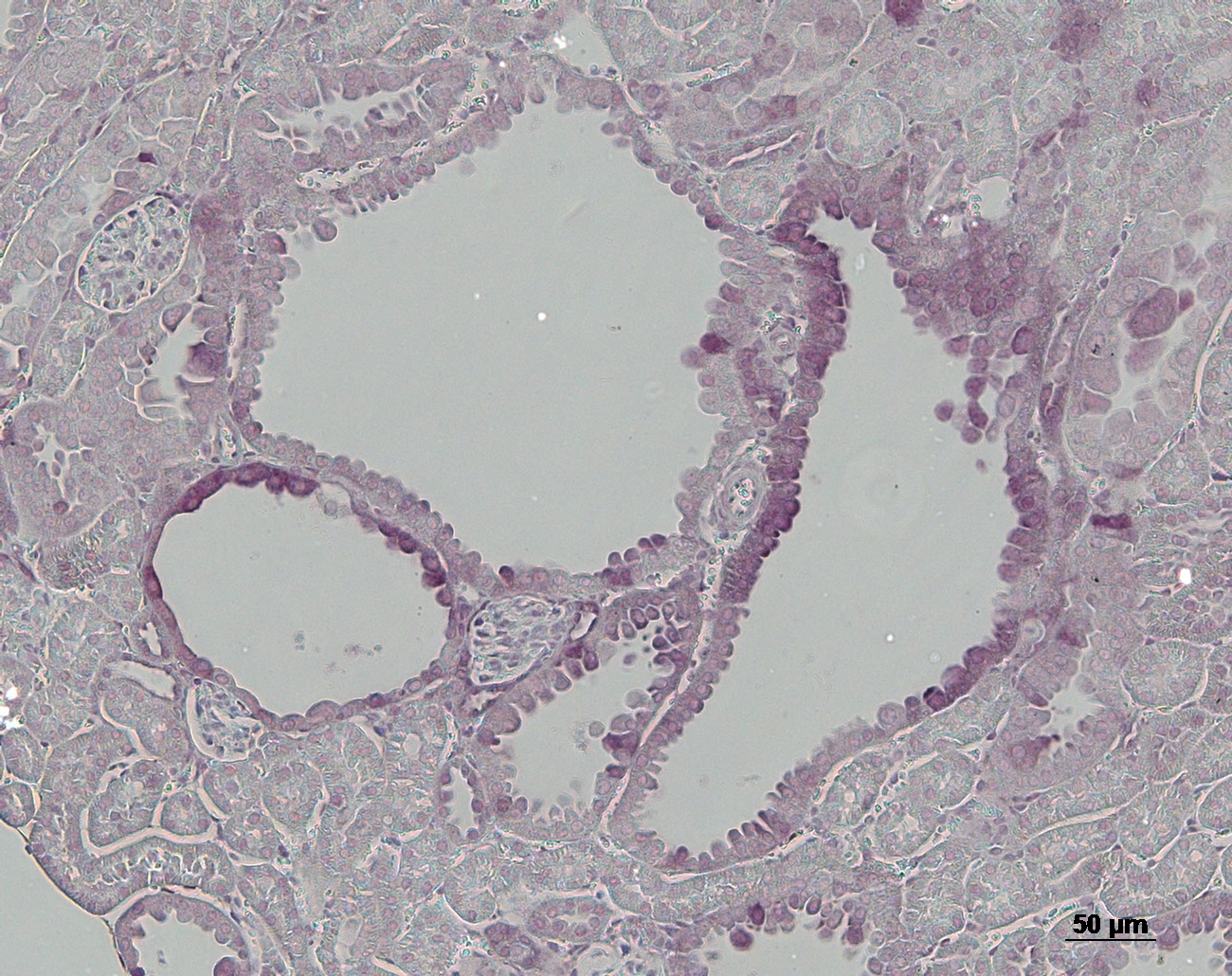

Supplement: Supplementary file 14 — Source data Fig. 6 [file 44321_2025_360_MOESM14_ESM.zip › EMM-2025-22130_SourceDataForFigure 6A-C 10-28-25/6B/Tsc1 KO pRSK 20X.tif]

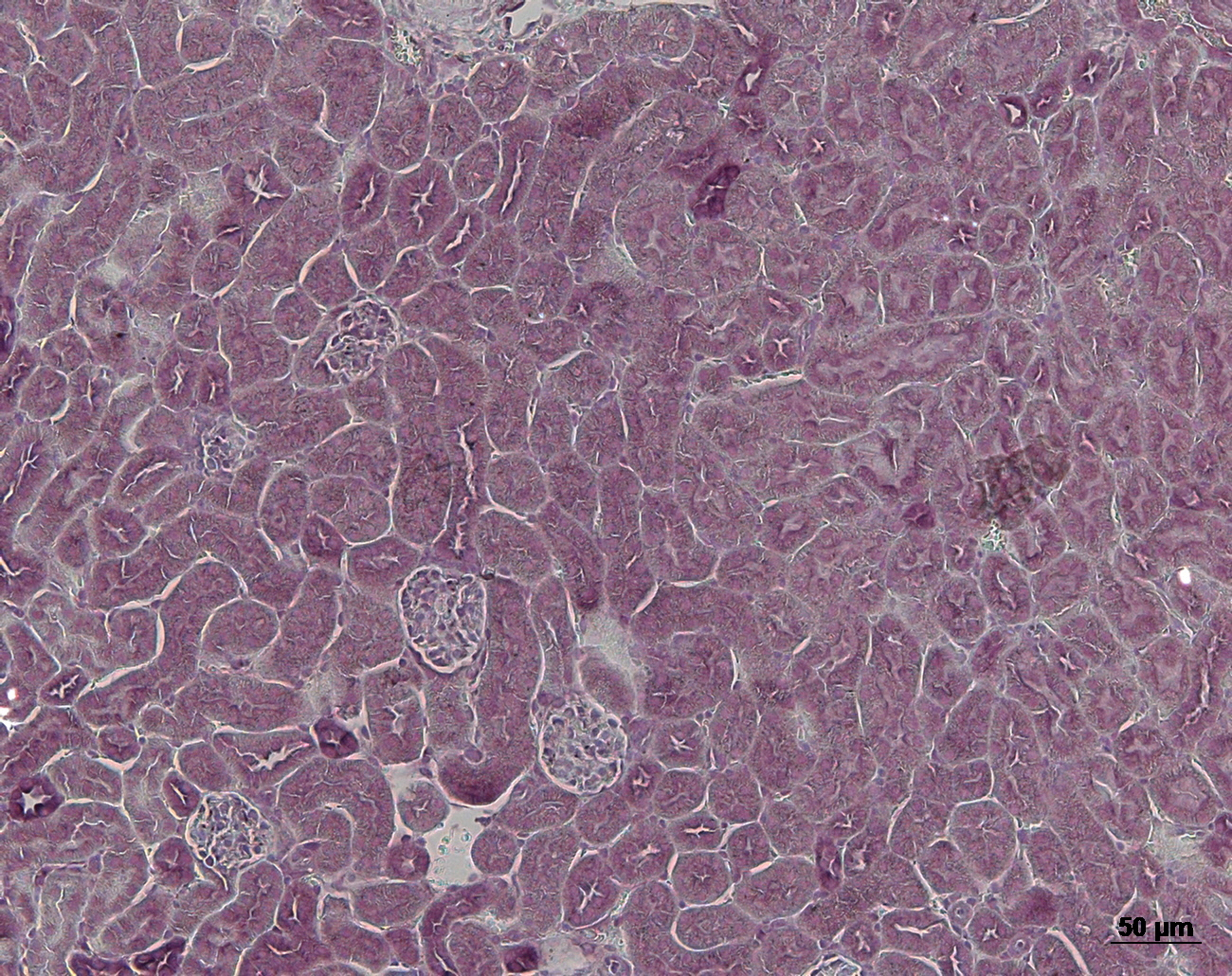

Supplement: Supplementary file 14 — Source data Fig. 6 [file 44321_2025_360_MOESM14_ESM.zip › EMM-2025-22130_SourceDataForFigure 6A-C 10-28-25/6B/WT pAKT 20X.tif]

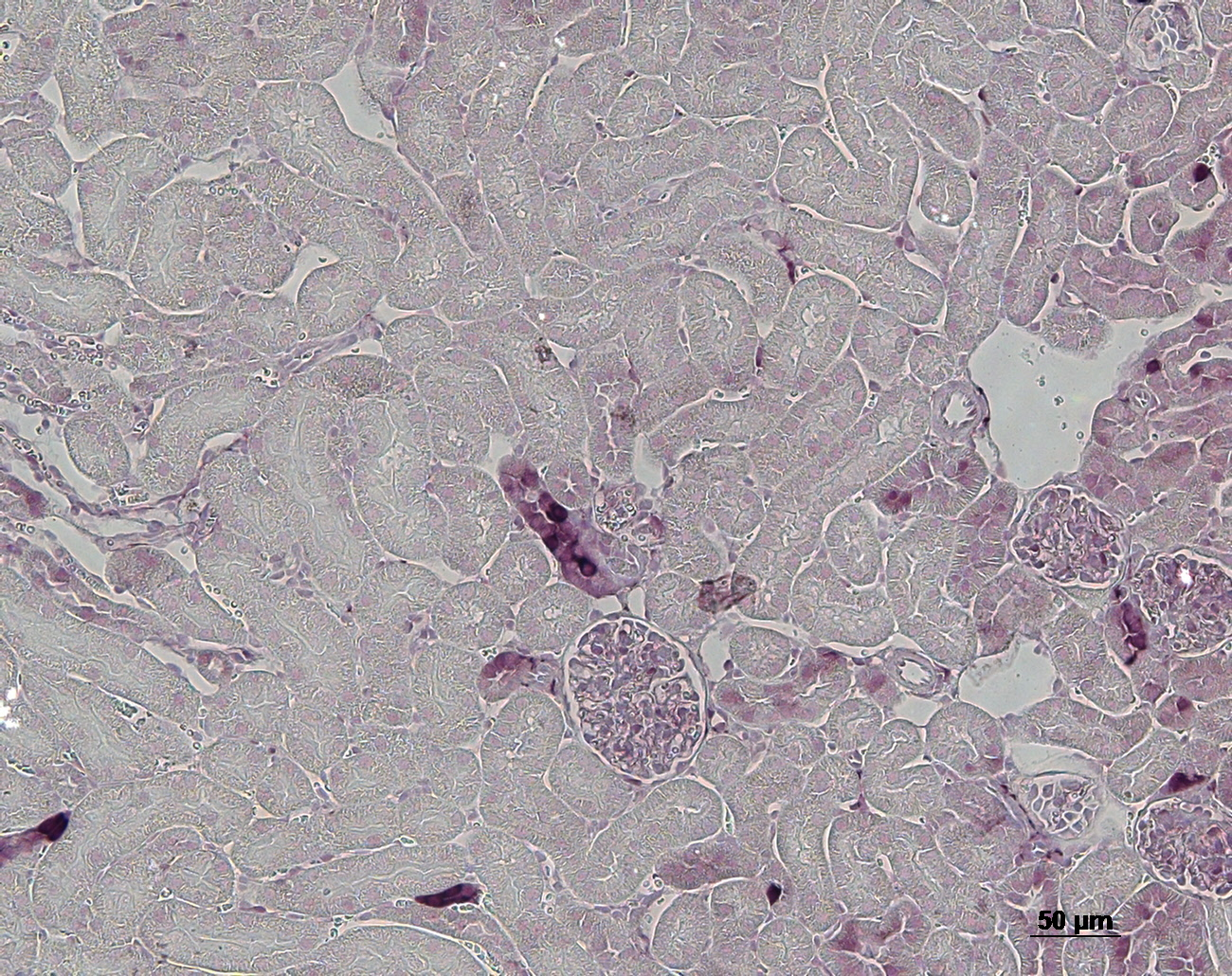

Supplement: Supplementary file 14 — Source data Fig. 6 [file 44321_2025_360_MOESM14_ESM.zip › EMM-2025-22130_SourceDataForFigure 6A-C 10-28-25/6B/WT pERK1-2 20X.tif]

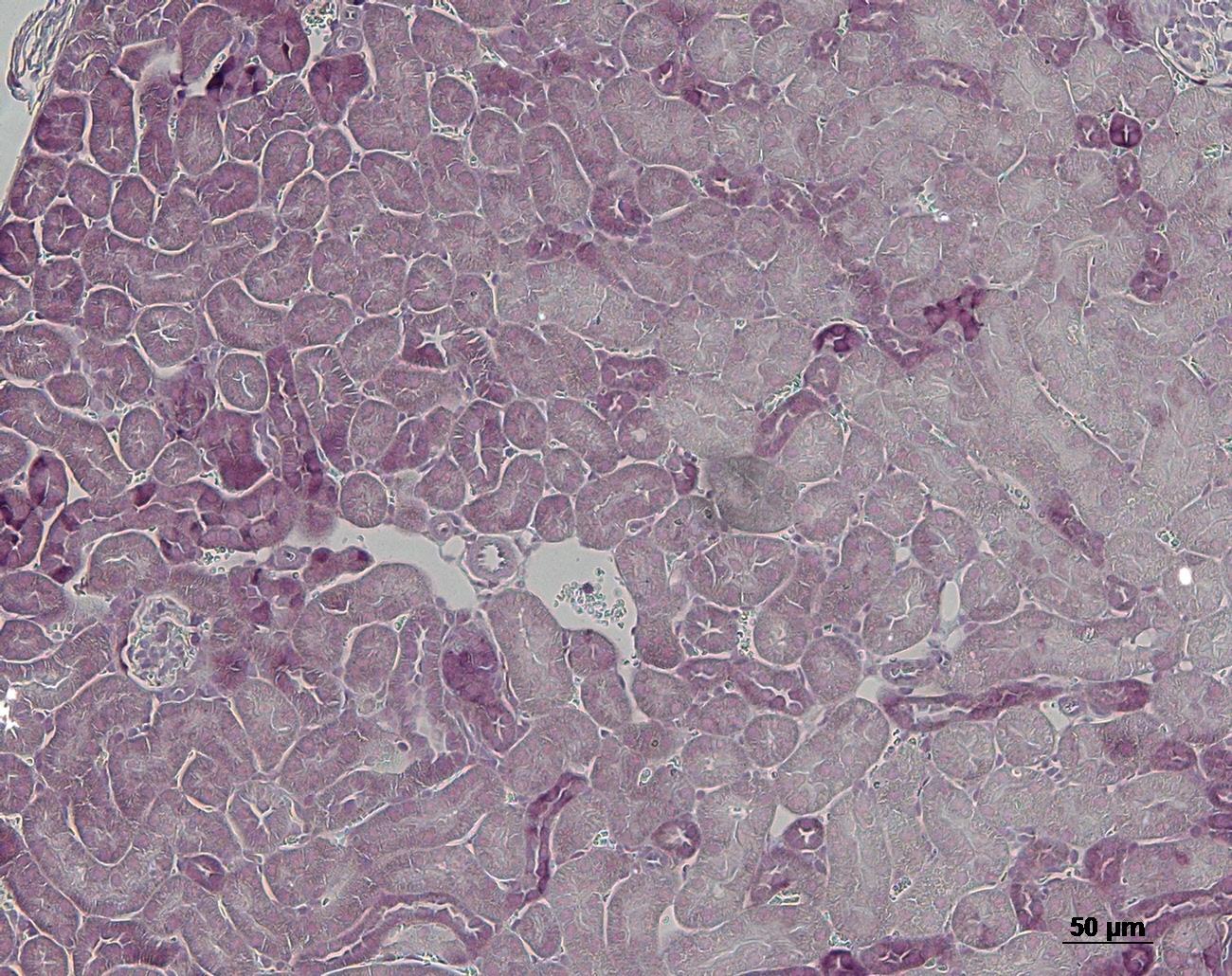

Supplement: Supplementary file 14 — Source data Fig. 6 [file 44321_2025_360_MOESM14_ESM.zip › EMM-2025-22130_SourceDataForFigure 6A-C 10-28-25/6B/WT pRSK 20X.tif]

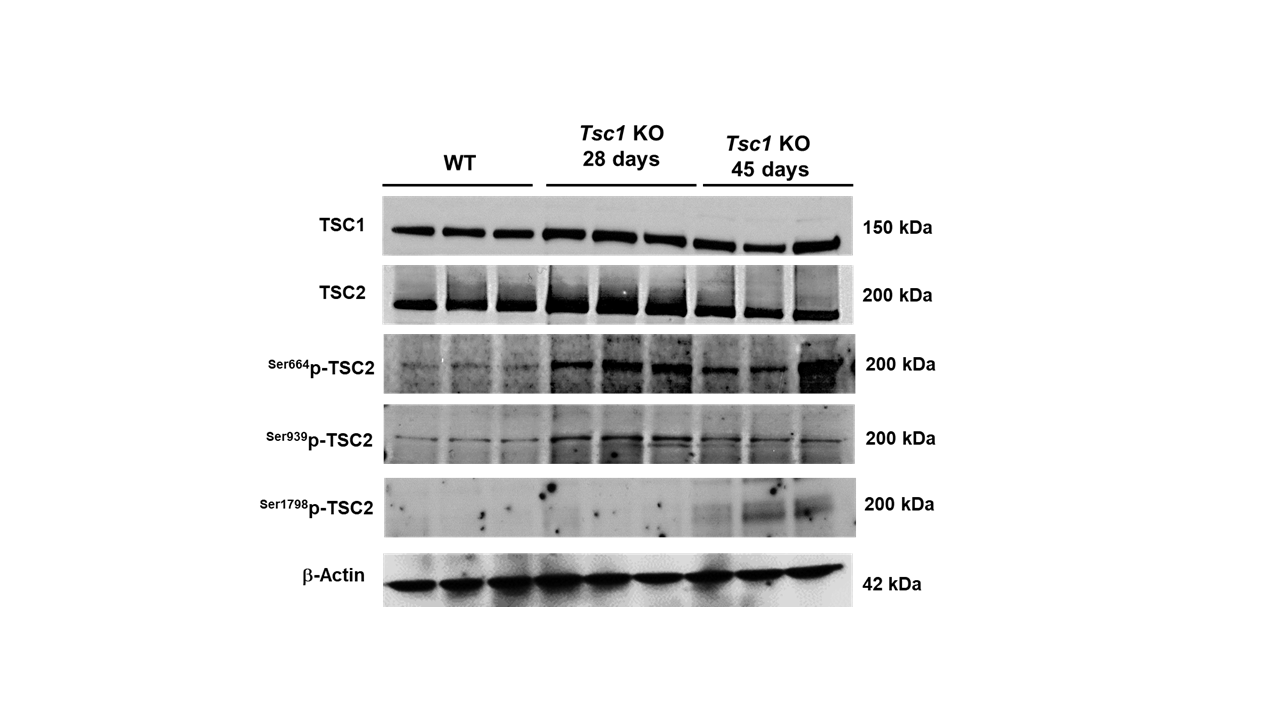

Supplement: Supplementary file 14 — Source data Fig. 6 [file 44321_2025_360_MOESM14_ESM.zip › EMM-2025-22130_SourceDataForFigure 6A-C 10-28-25/6C/Fig 6C Western Blot 10-29-25.tif]

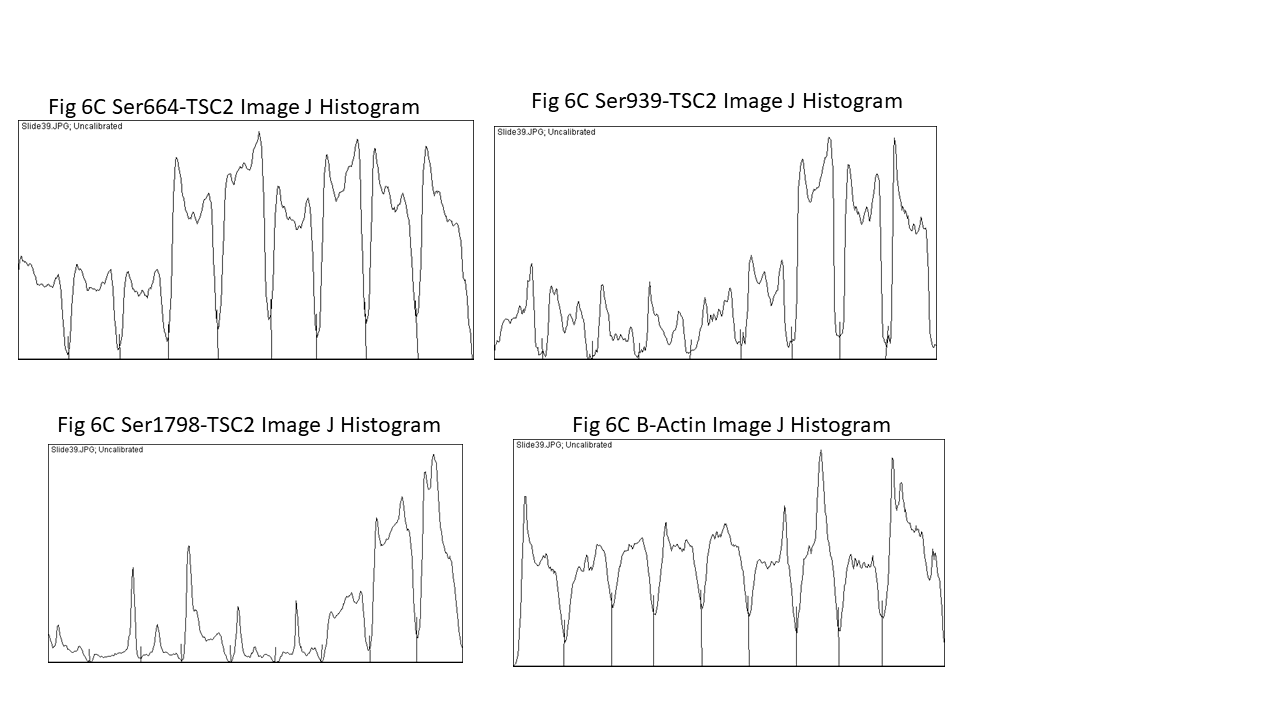

Supplement: Supplementary file 14 — Source data Fig. 6 [file 44321_2025_360_MOESM14_ESM.zip › EMM-2025-22130_SourceDataForFigure 6A-C 10-28-25/6C/Western Blot Image J Histograms/Slide1.TIF]

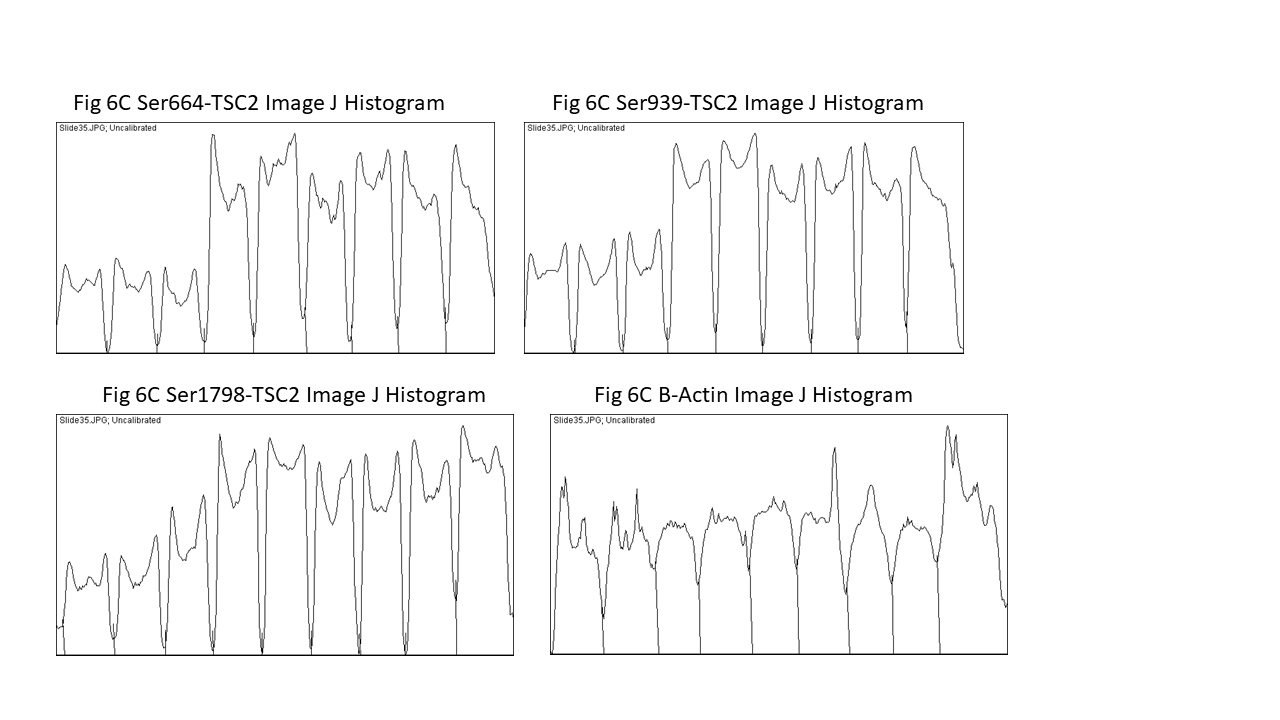

Supplement: Supplementary file 14 — Source data Fig. 6 [file 44321_2025_360_MOESM14_ESM.zip › EMM-2025-22130_SourceDataForFigure 6A-C 10-28-25/6C/Western Blot Image J Histograms/Slide2.TIF]

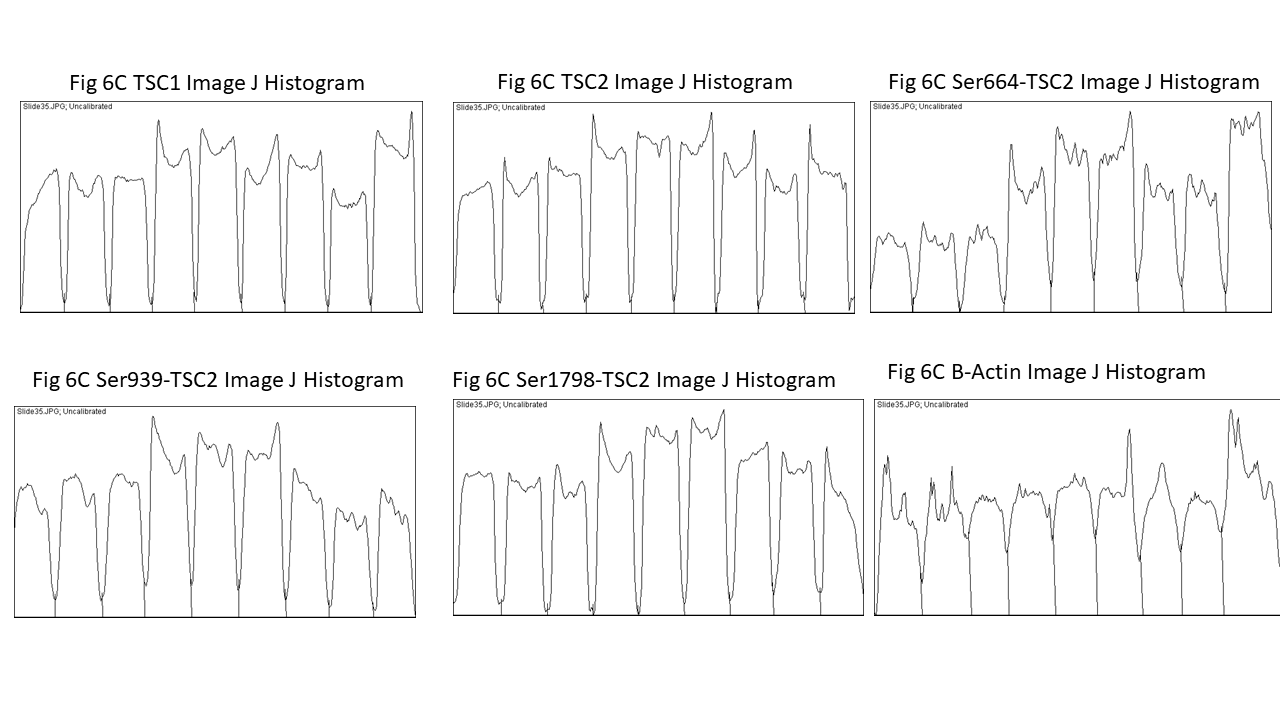

Supplement: Supplementary file 14 — Source data Fig. 6 [file 44321_2025_360_MOESM14_ESM.zip › EMM-2025-22130_SourceDataForFigure 6A-C 10-28-25/6C/Western Blot Image J Histograms/Slide3.TIF]

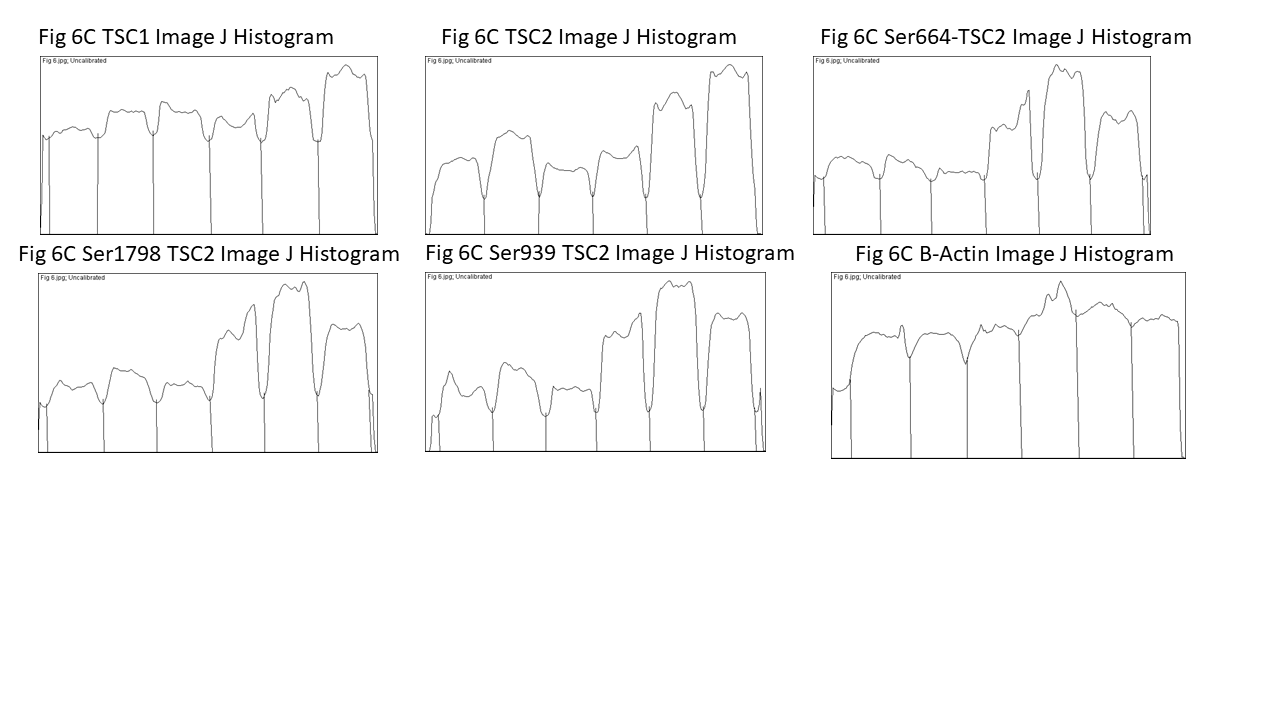

Supplement: Supplementary file 14 — Source data Fig. 6 [file 44321_2025_360_MOESM14_ESM.zip › EMM-2025-22130_SourceDataForFigure 6A-C 10-28-25/6C/Western Blot Image J Histograms/Slide4.TIF]

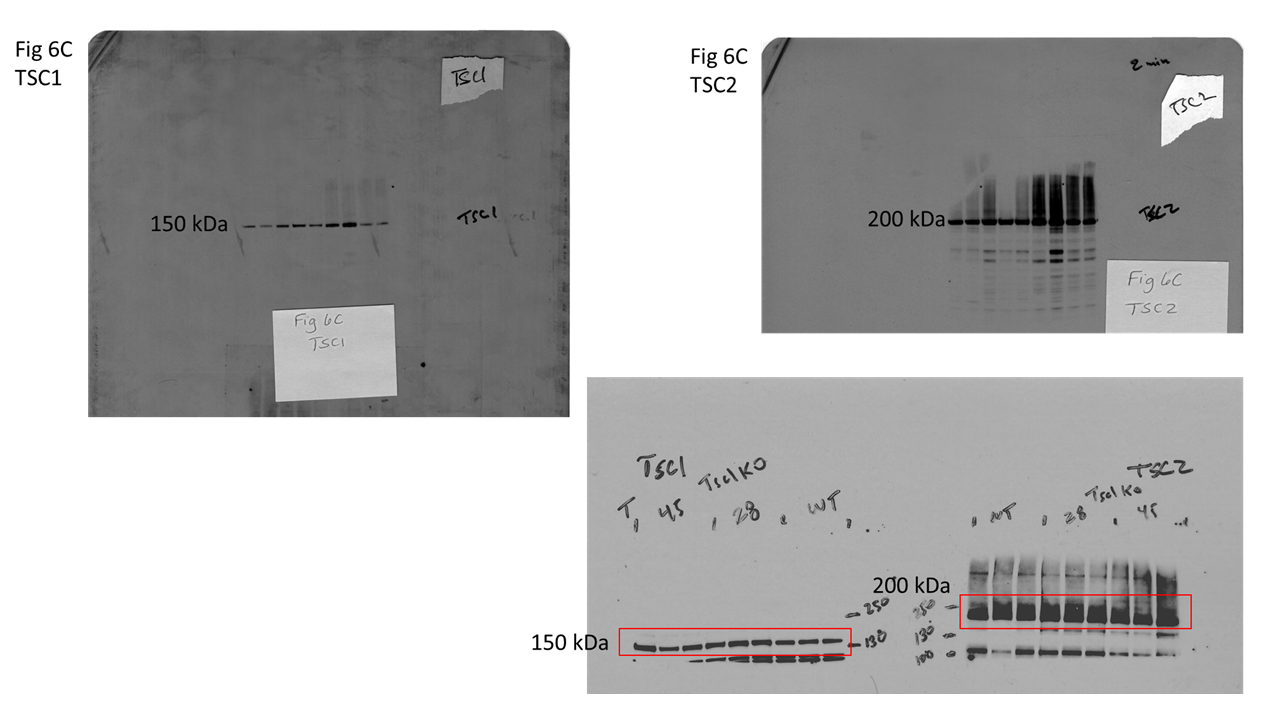

Supplement: Supplementary file 14 — Source data Fig. 6 [file 44321_2025_360_MOESM14_ESM.zip › EMM-2025-22130_SourceDataForFigure 6A-C 10-28-25/6C/Western Blot Source Data 10-29-25/Slide1.TIF]

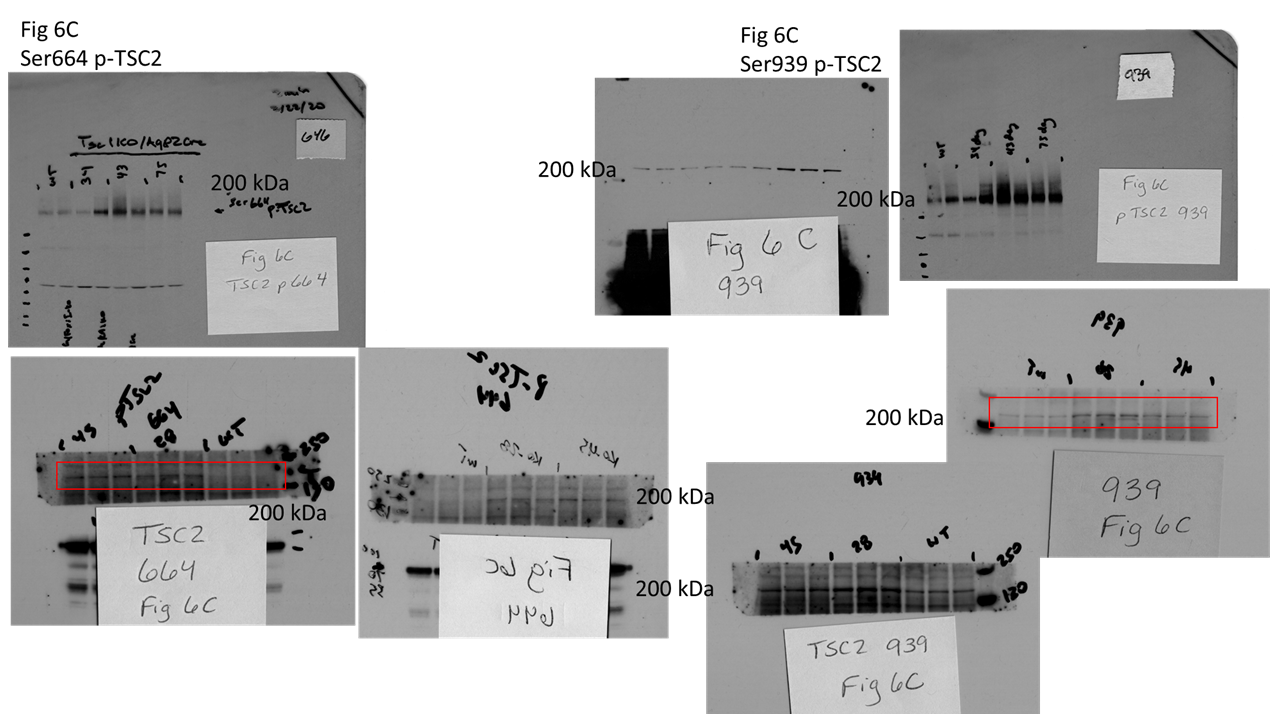

Supplement: Supplementary file 14 — Source data Fig. 6 [file 44321_2025_360_MOESM14_ESM.zip › EMM-2025-22130_SourceDataForFigure 6A-C 10-28-25/6C/Western Blot Source Data 10-29-25/Slide2.TIF]

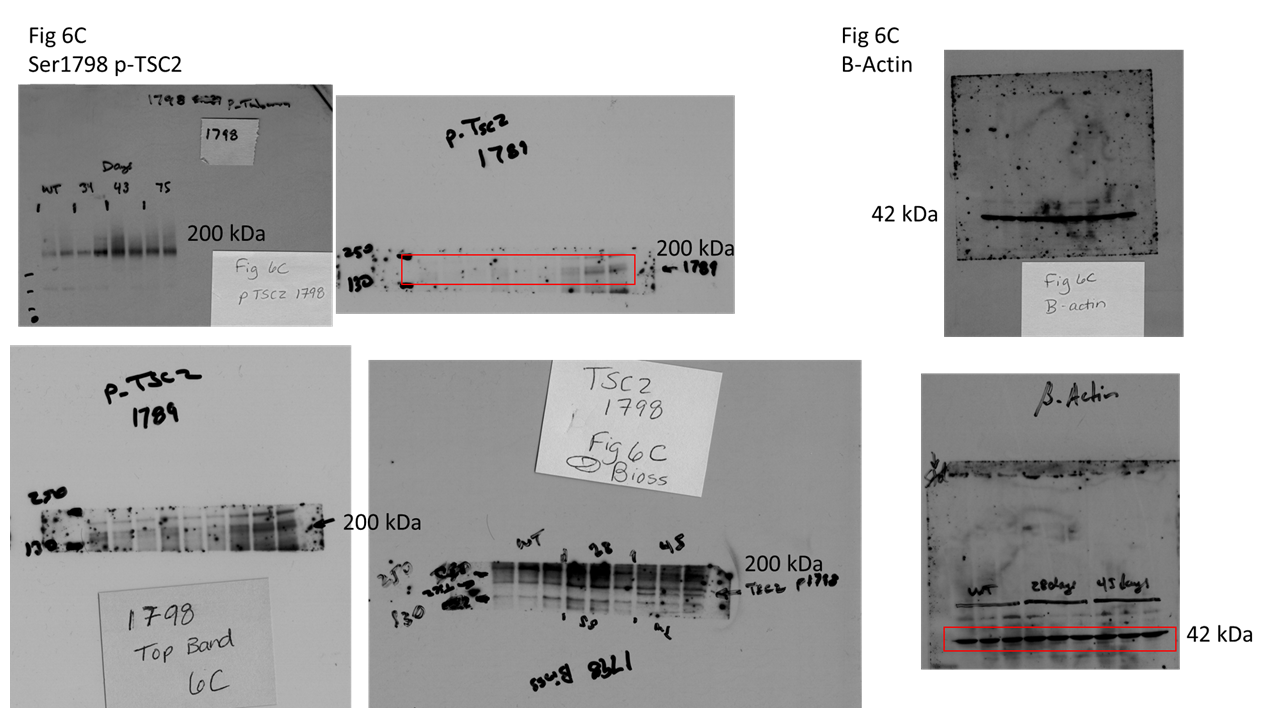

Supplement: Supplementary file 14 — Source data Fig. 6 [file 44321_2025_360_MOESM14_ESM.zip › EMM-2025-22130_SourceDataForFigure 6A-C 10-28-25/6C/Western Blot Source Data 10-29-25/Slide3.TIF]

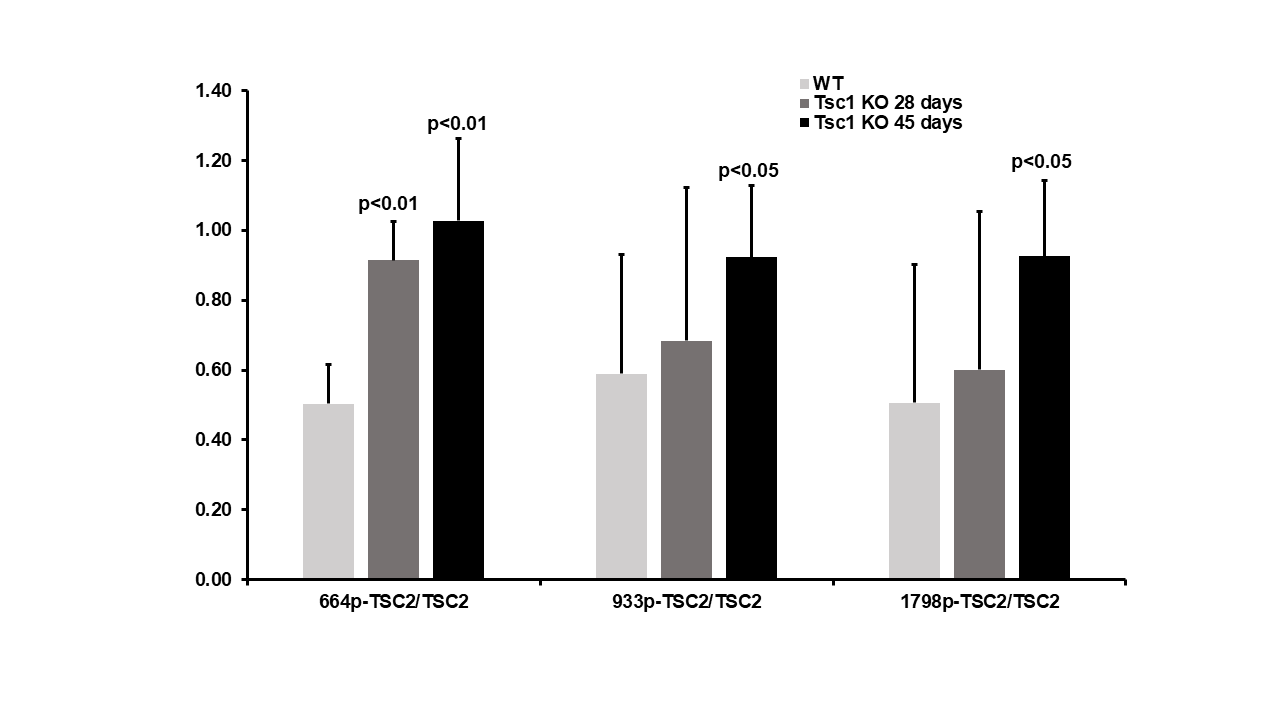

Supplement: Supplementary file 14 — Source data Fig. 6 [file 44321_2025_360_MOESM14_ESM.zip › EMM-2025-22130_SourceDataForFigure 6A-C 10-28-25/6C/Western Quantification.tif]

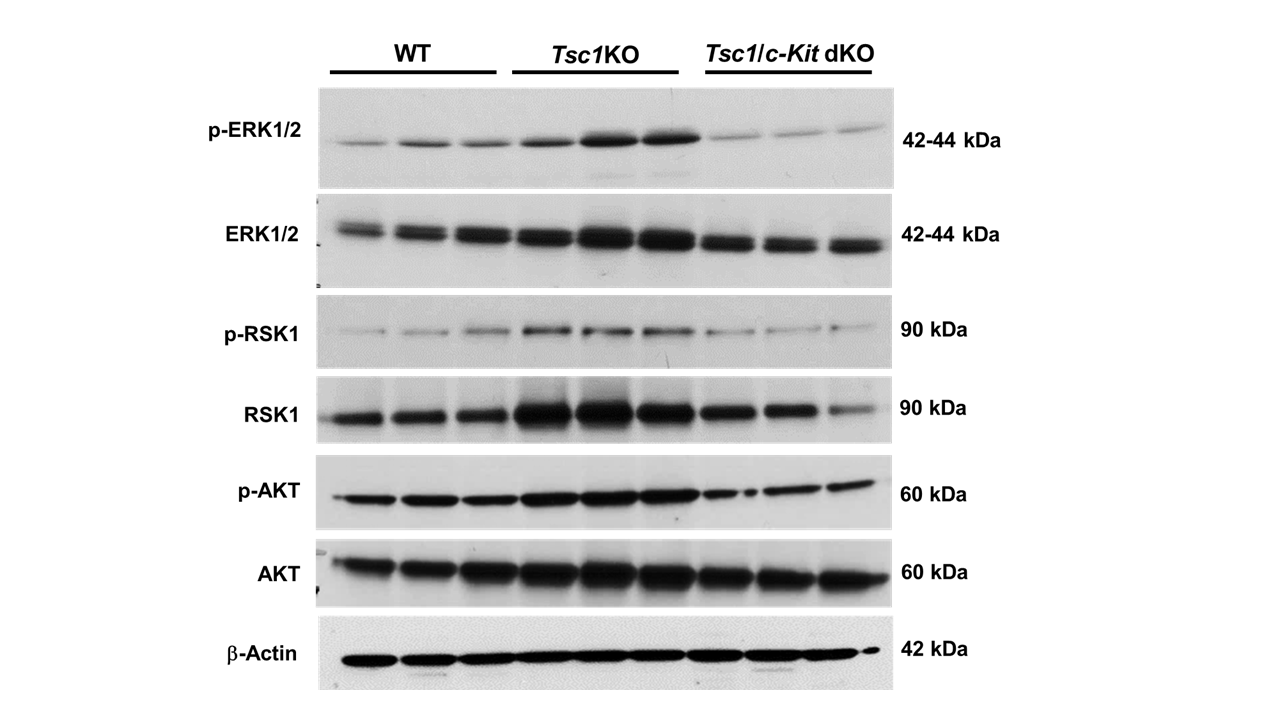

Supplement: Supplementary file 15 — Source data Fig. 7 [file 44321_2025_360_MOESM15_ESM.zip › EMM-2025-22130_SourceDataForFigure 7A-C 10-28-25/7A/Fig 7A Western Blot 10-28-25.tif]
