# Supplementary figures and images for: The critical role of the proto-oncogene c-Kit in TSC renal cystogenesis (part 2 of 2)
Source: EMBO Mol Med. 2025 Dec 22;18(2):575–98. doi: 10.1038/s44321-025-00360-x (PMC12905254; doi:10.1038/s44321-025-00360-x)

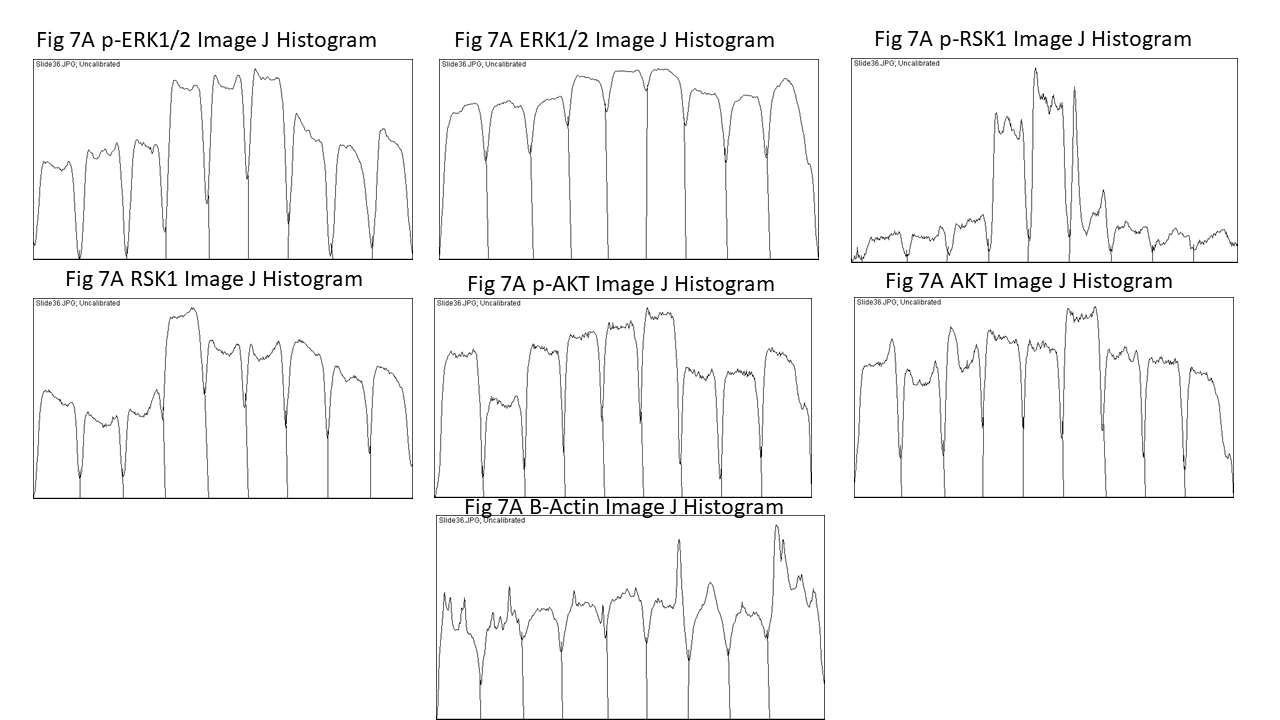

Supplement: Supplementary file 15 — Source data Fig. 7 [file 44321_2025_360_MOESM15_ESM.zip › EMM-2025-22130_SourceDataForFigure 7A-C 10-28-25/7A/Western Blot Image J Histograms/Slide1.TIF]

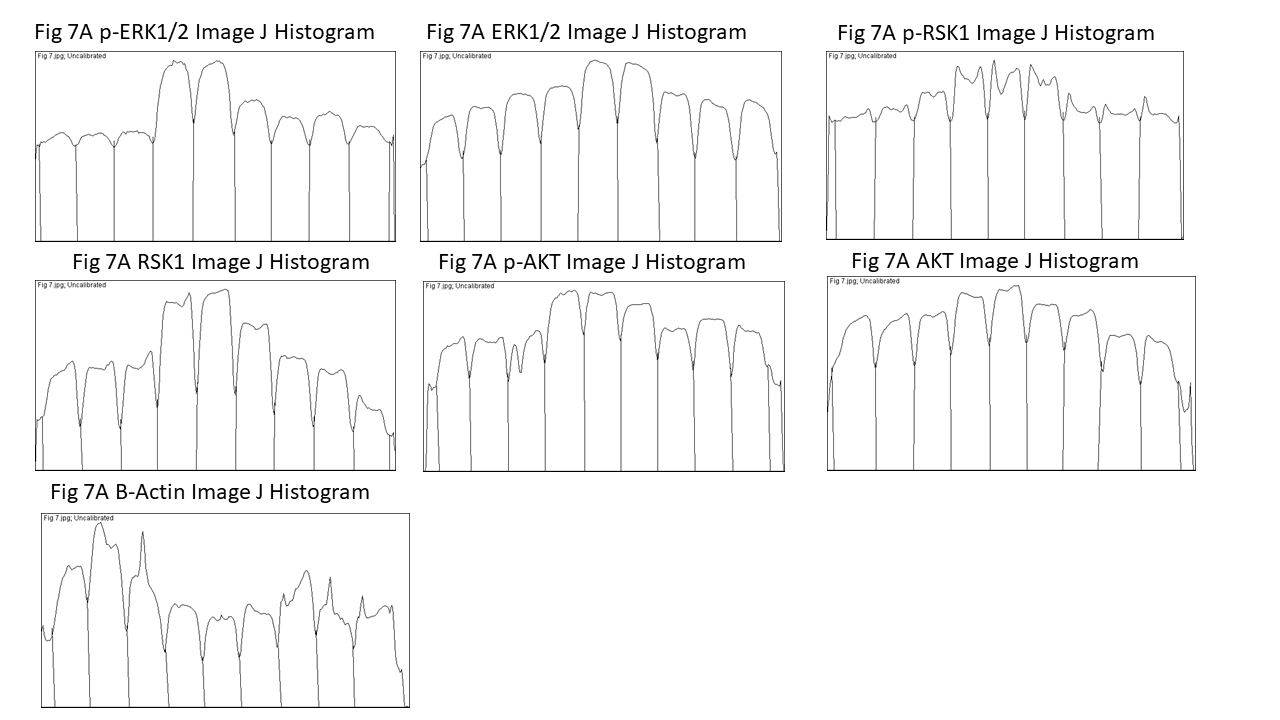

Supplement: Supplementary file 15 — Source data Fig. 7 [file 44321_2025_360_MOESM15_ESM.zip › EMM-2025-22130_SourceDataForFigure 7A-C 10-28-25/7A/Western Blot Image J Histograms/Slide2.TIF]

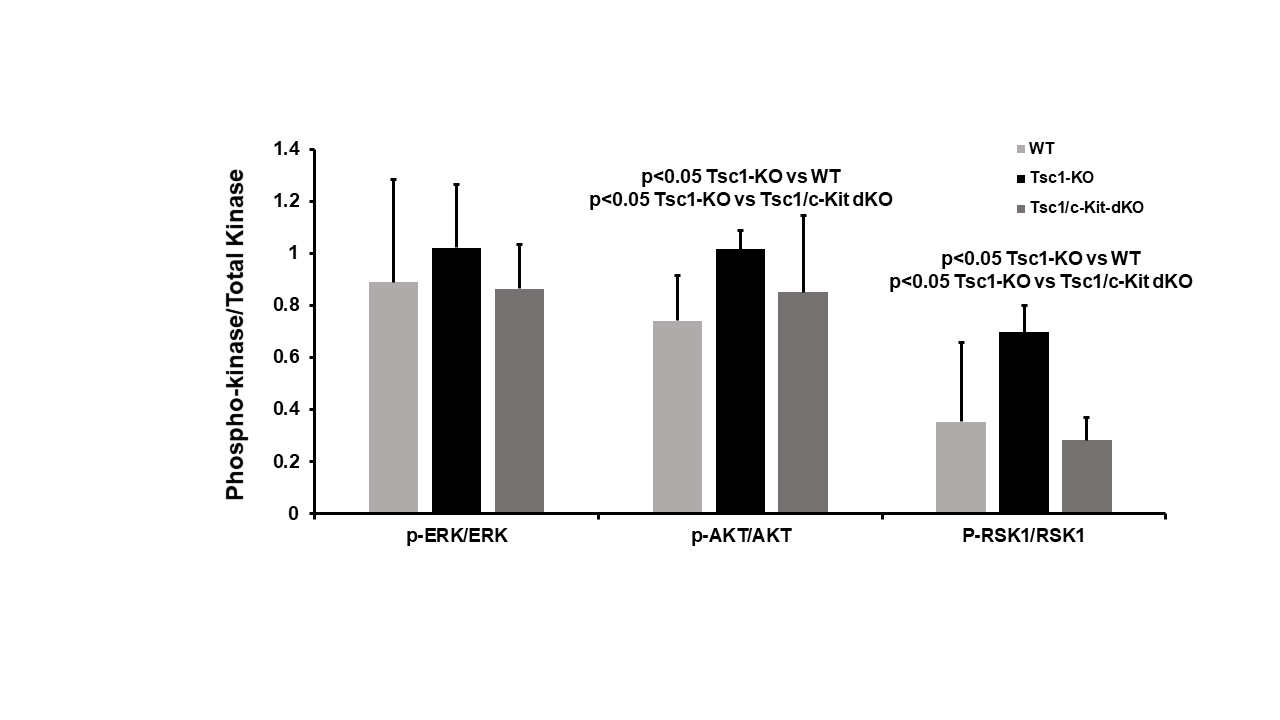

Supplement: Supplementary file 15 — Source data Fig. 7 [file 44321_2025_360_MOESM15_ESM.zip › EMM-2025-22130_SourceDataForFigure 7A-C 10-28-25/7A/Western Blot Quantification.tif]

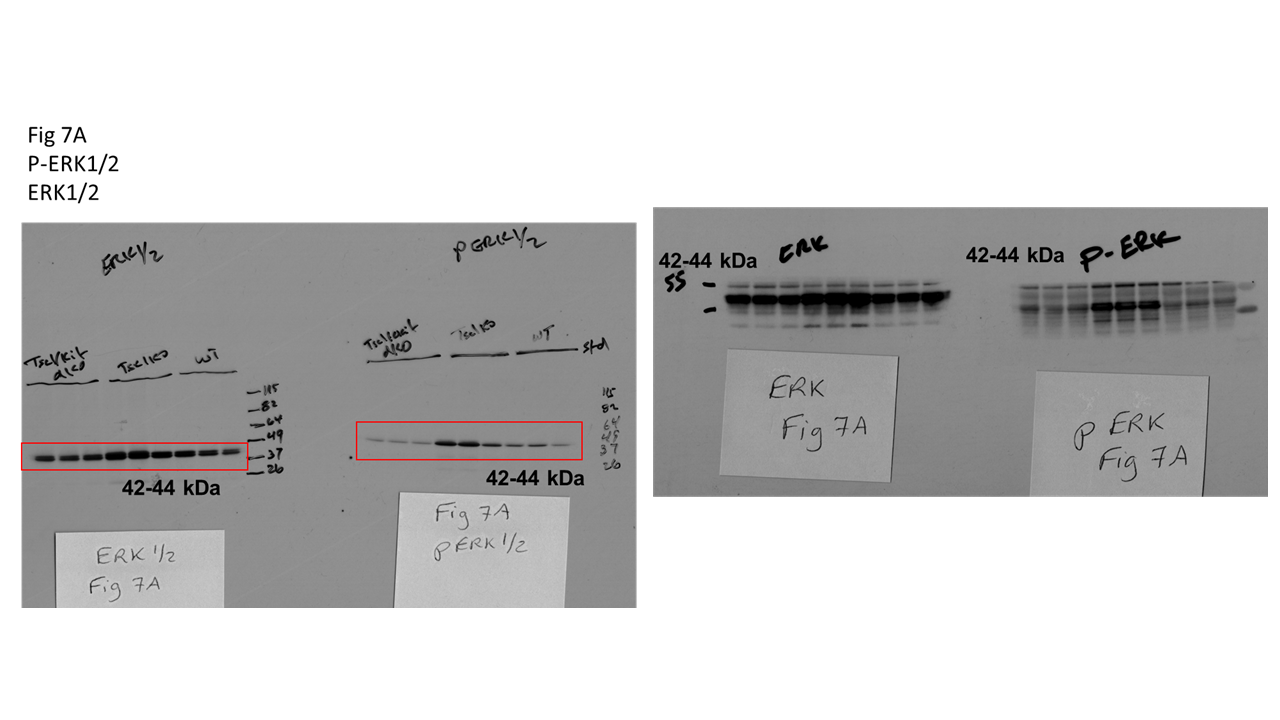

Supplement: Supplementary file 15 — Source data Fig. 7 [file 44321_2025_360_MOESM15_ESM.zip › EMM-2025-22130_SourceDataForFigure 7A-C 10-28-25/7A/Western Blot Source Data 10-29-25/Slide1.TIF]

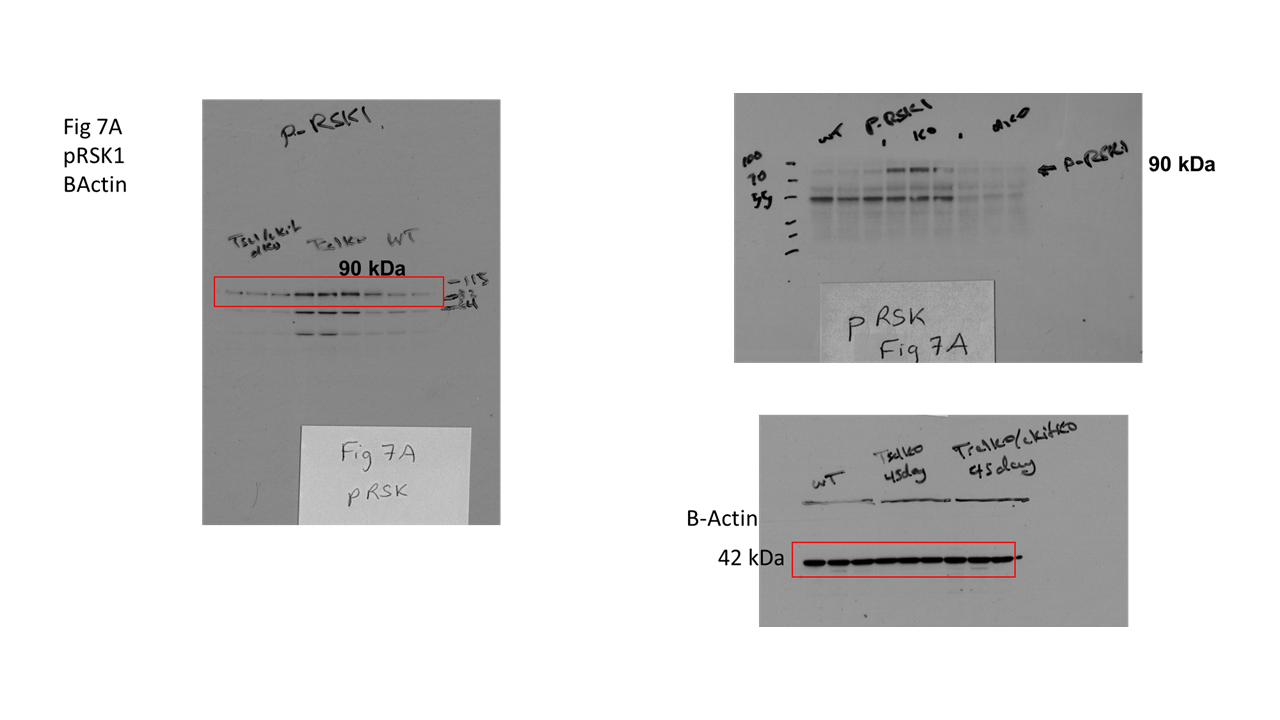

Supplement: Supplementary file 15 — Source data Fig. 7 [file 44321_2025_360_MOESM15_ESM.zip › EMM-2025-22130_SourceDataForFigure 7A-C 10-28-25/7A/Western Blot Source Data 10-29-25/Slide2.TIF]

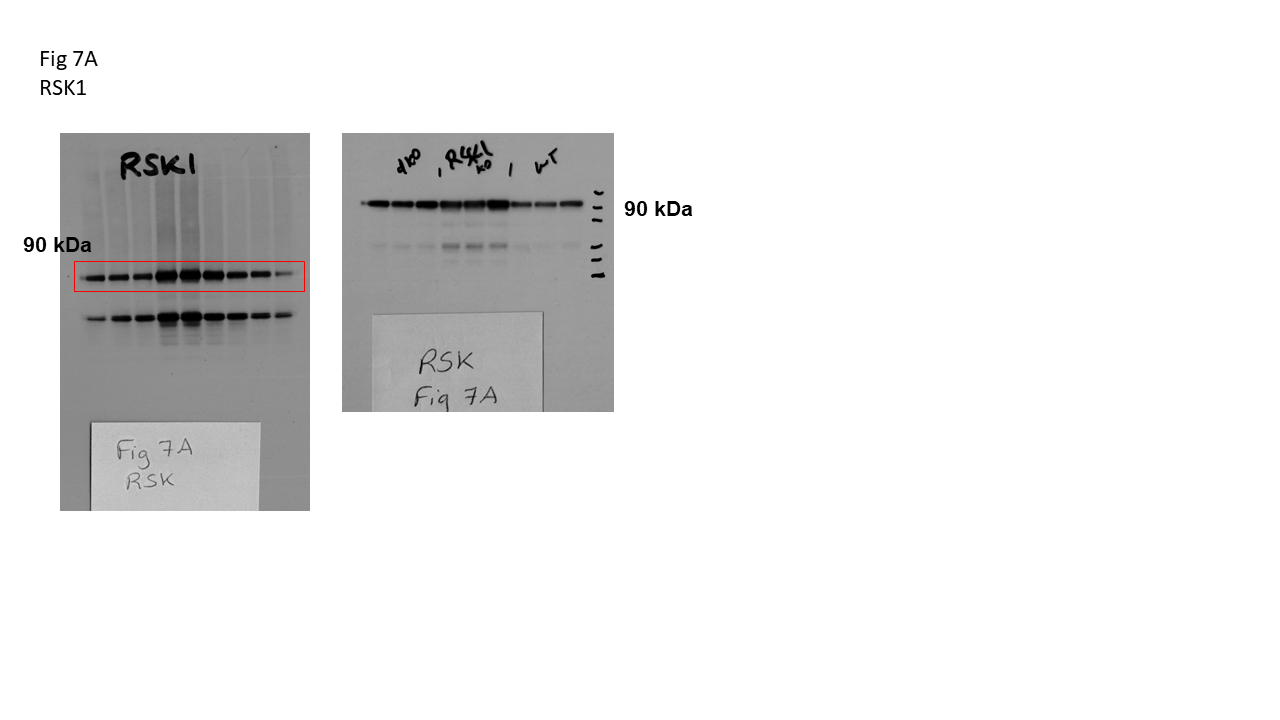

Supplement: Supplementary file 15 — Source data Fig. 7 [file 44321_2025_360_MOESM15_ESM.zip › EMM-2025-22130_SourceDataForFigure 7A-C 10-28-25/7A/Western Blot Source Data 10-29-25/Slide3.TIF]

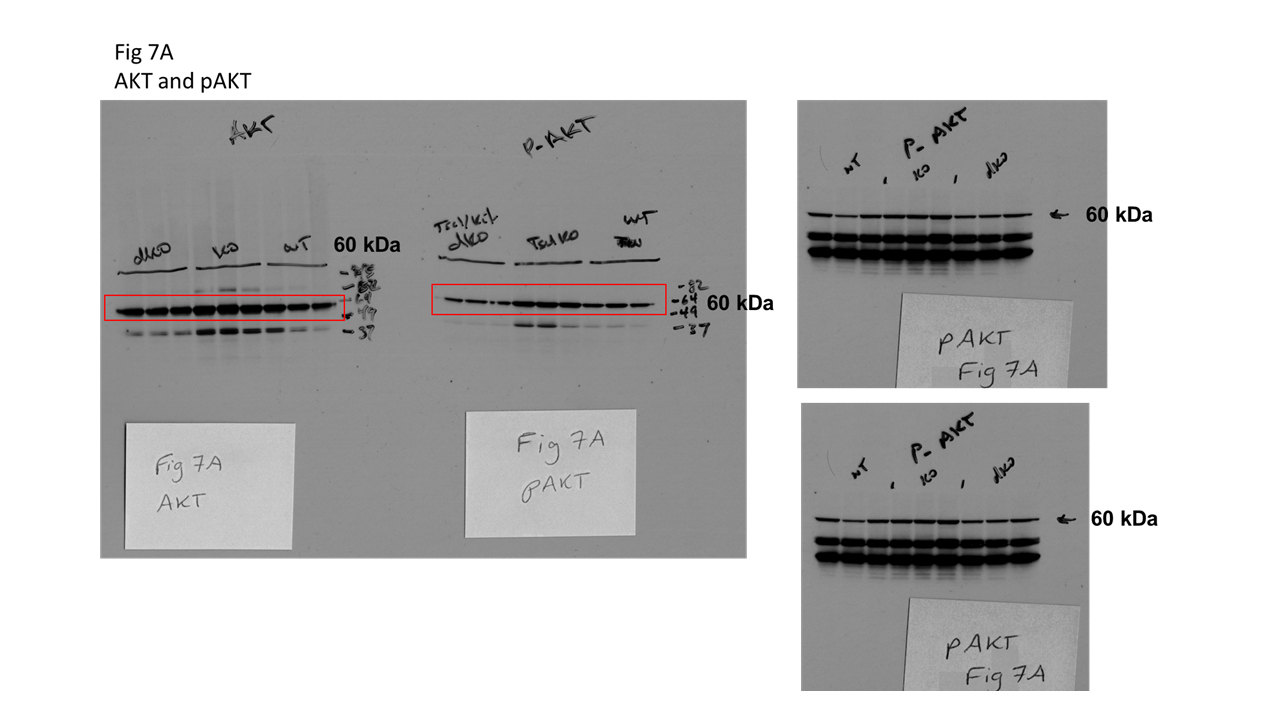

Supplement: Supplementary file 15 — Source data Fig. 7 [file 44321_2025_360_MOESM15_ESM.zip › EMM-2025-22130_SourceDataForFigure 7A-C 10-28-25/7A/Western Blot Source Data 10-29-25/Slide4.TIF]

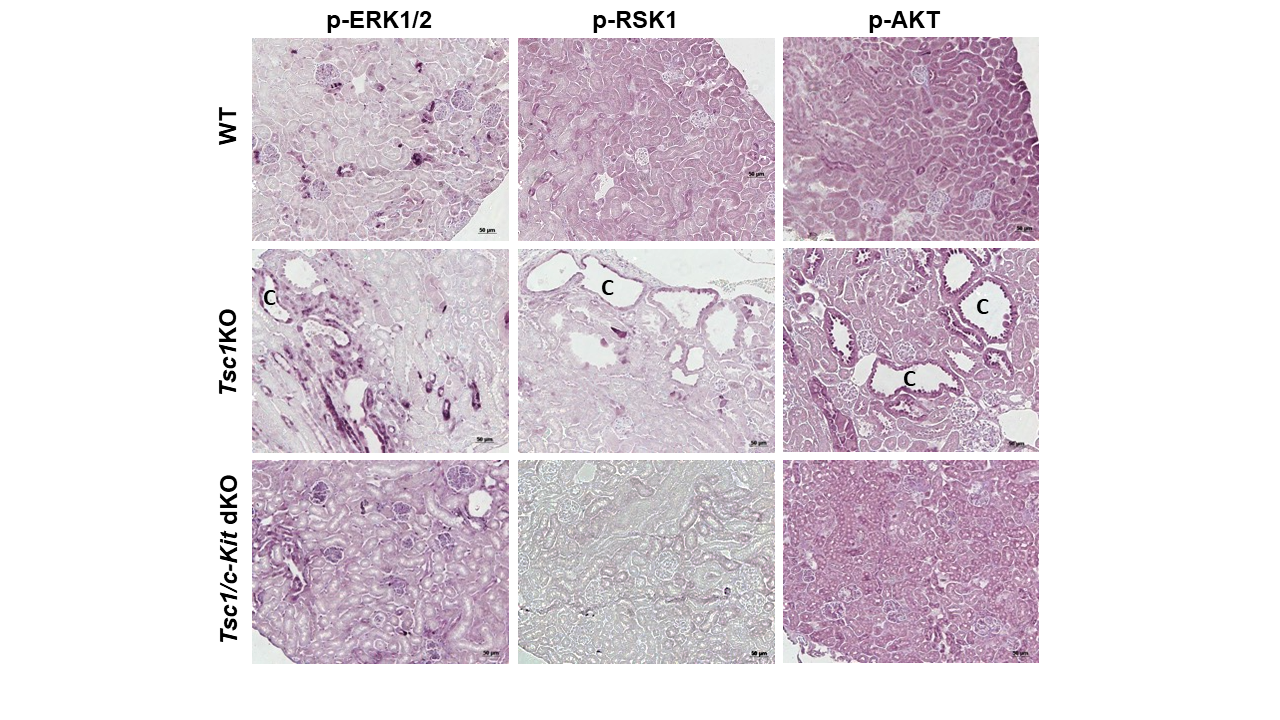

Supplement: Supplementary file 15 — Source data Fig. 7 [file 44321_2025_360_MOESM15_ESM.zip › EMM-2025-22130_SourceDataForFigure 7A-C 10-28-25/7B/Figure 7B.tif]

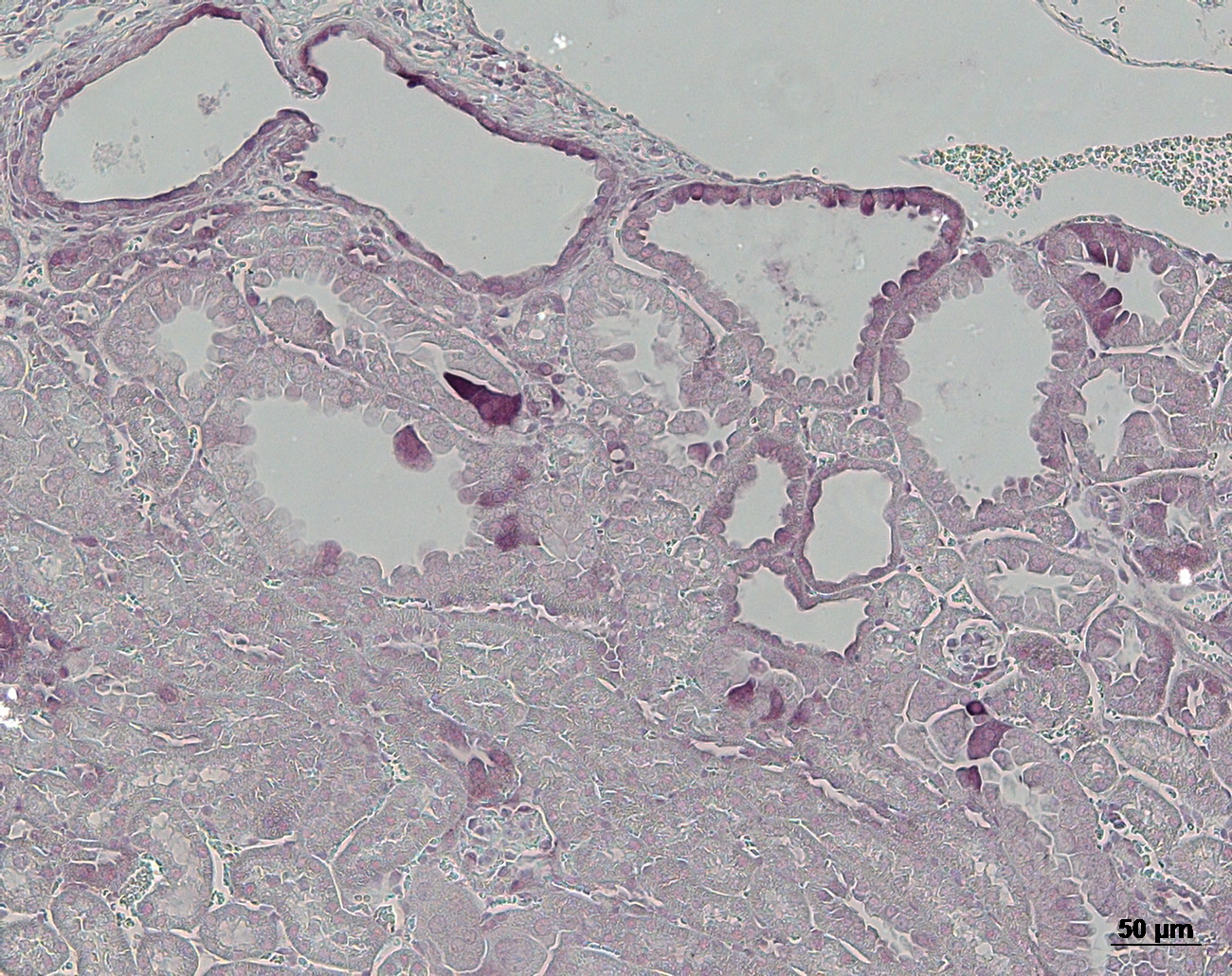

Supplement: Supplementary file 15 — Source data Fig. 7 [file 44321_2025_360_MOESM15_ESM.zip › EMM-2025-22130_SourceDataForFigure 7A-C 10-28-25/7B/Tsc1 KO pRSK 20X.tif]

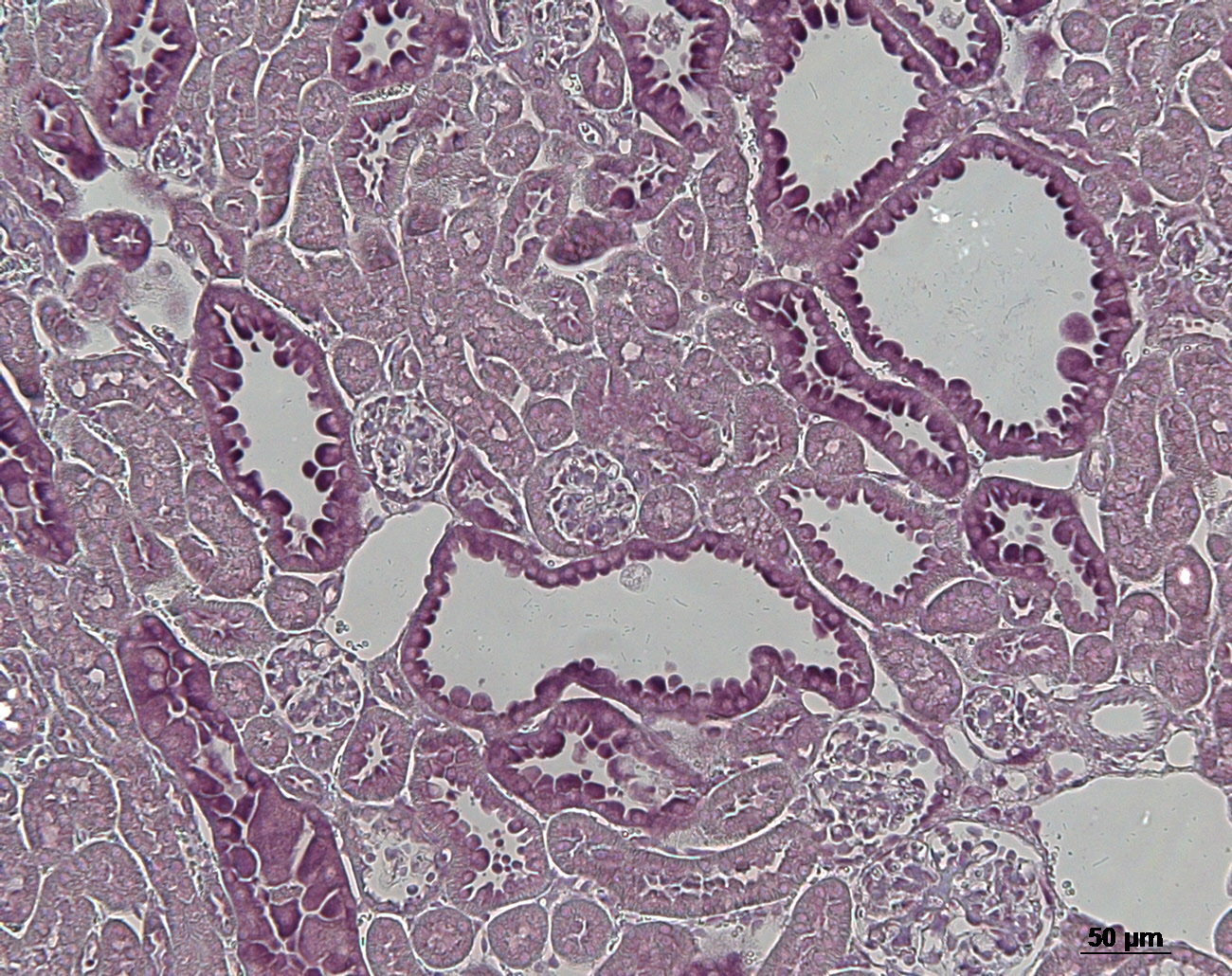

Supplement: Supplementary file 15 — Source data Fig. 7 [file 44321_2025_360_MOESM15_ESM.zip › EMM-2025-22130_SourceDataForFigure 7A-C 10-28-25/7B/Tsc1 KO pAKT 20X.tif]

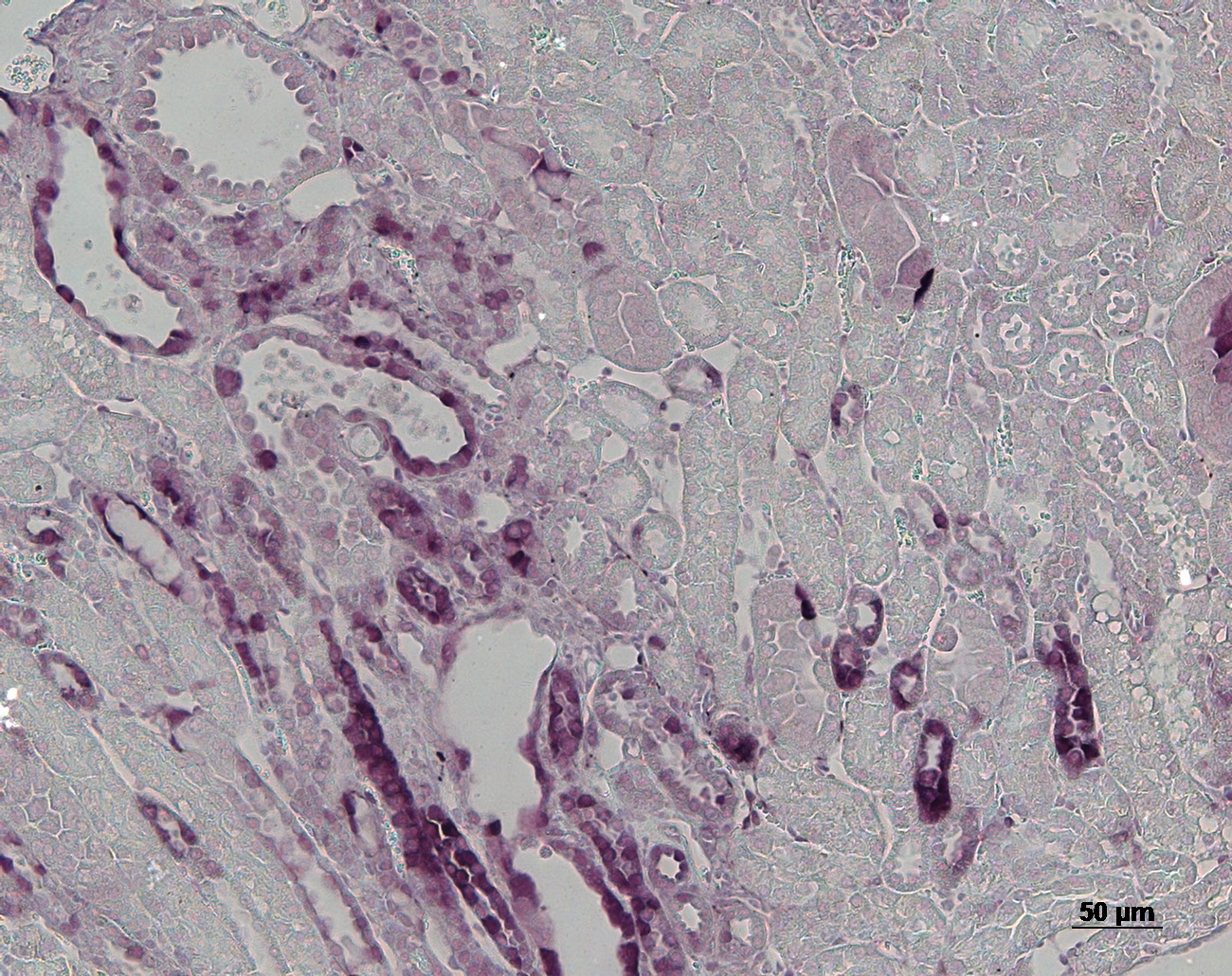

Supplement: Supplementary file 15 — Source data Fig. 7 [file 44321_2025_360_MOESM15_ESM.zip › EMM-2025-22130_SourceDataForFigure 7A-C 10-28-25/7B/Tsc1 KO pERK1-2 20X.tif]

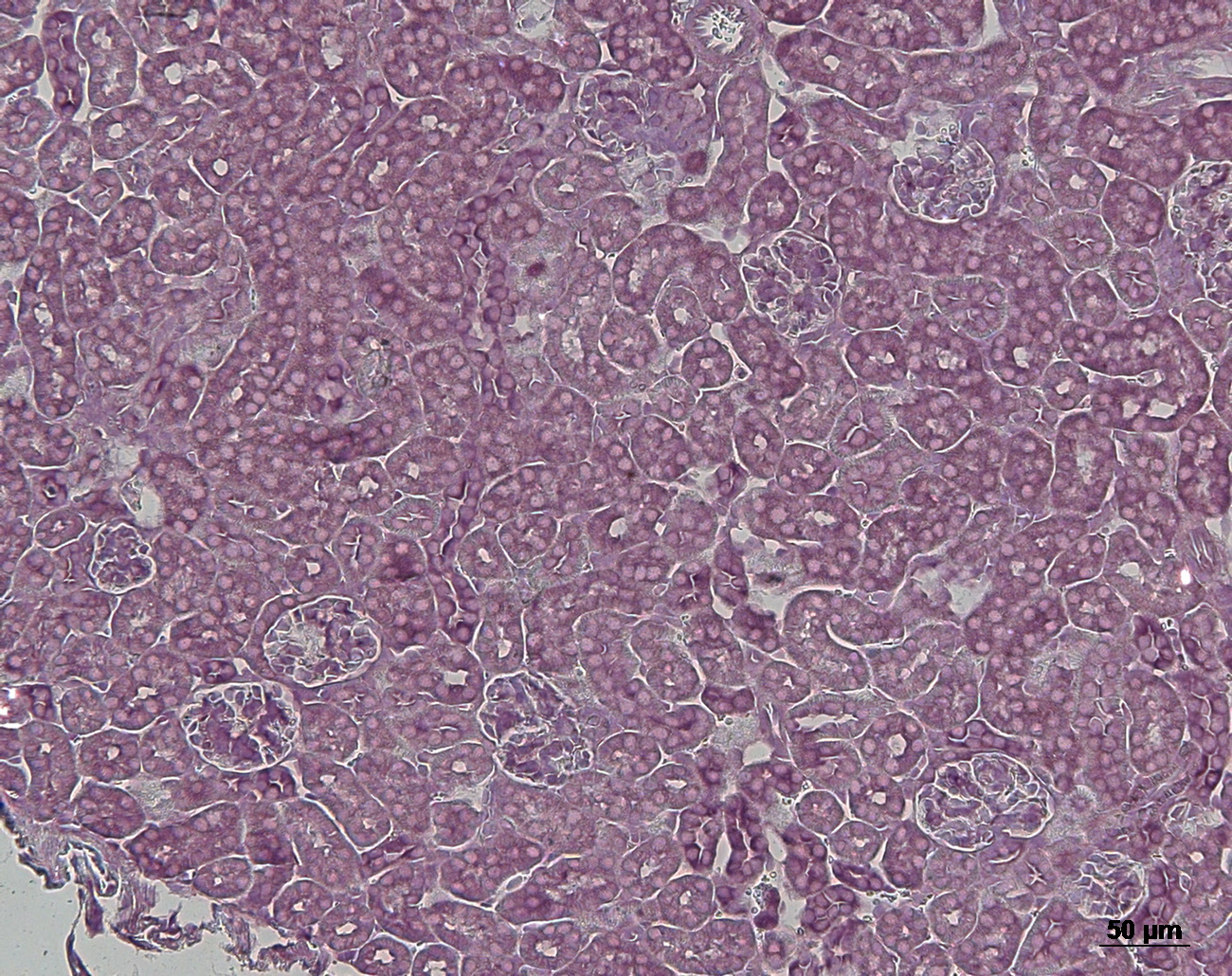

Supplement: Supplementary file 15 — Source data Fig. 7 [file 44321_2025_360_MOESM15_ESM.zip › EMM-2025-22130_SourceDataForFigure 7A-C 10-28-25/7B/Tsc1-cKIT dKO pAKT 20X.tif]

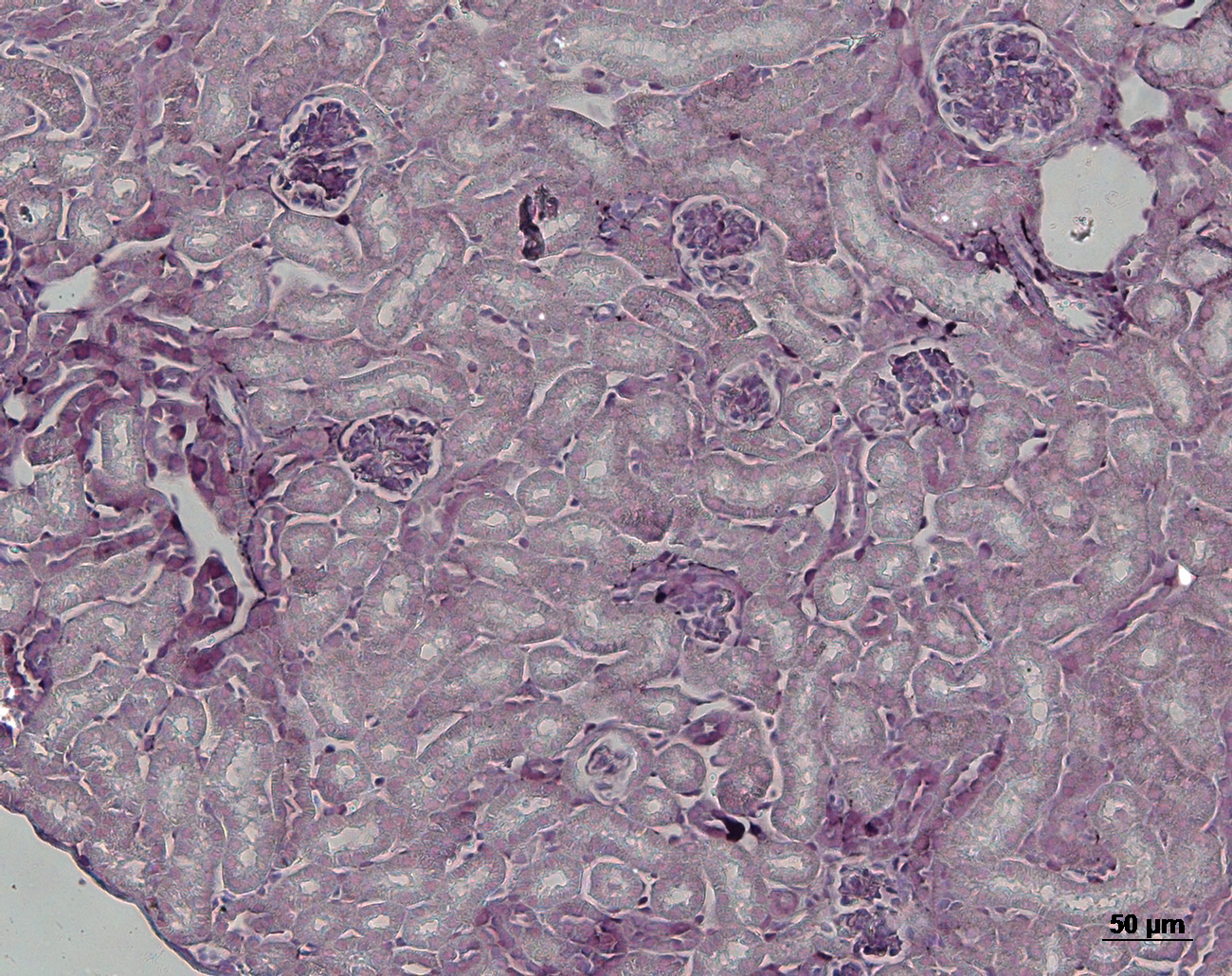

Supplement: Supplementary file 15 — Source data Fig. 7 [file 44321_2025_360_MOESM15_ESM.zip › EMM-2025-22130_SourceDataForFigure 7A-C 10-28-25/7B/Tsc1-cKIT dKO pERK1-2 20X.tif]

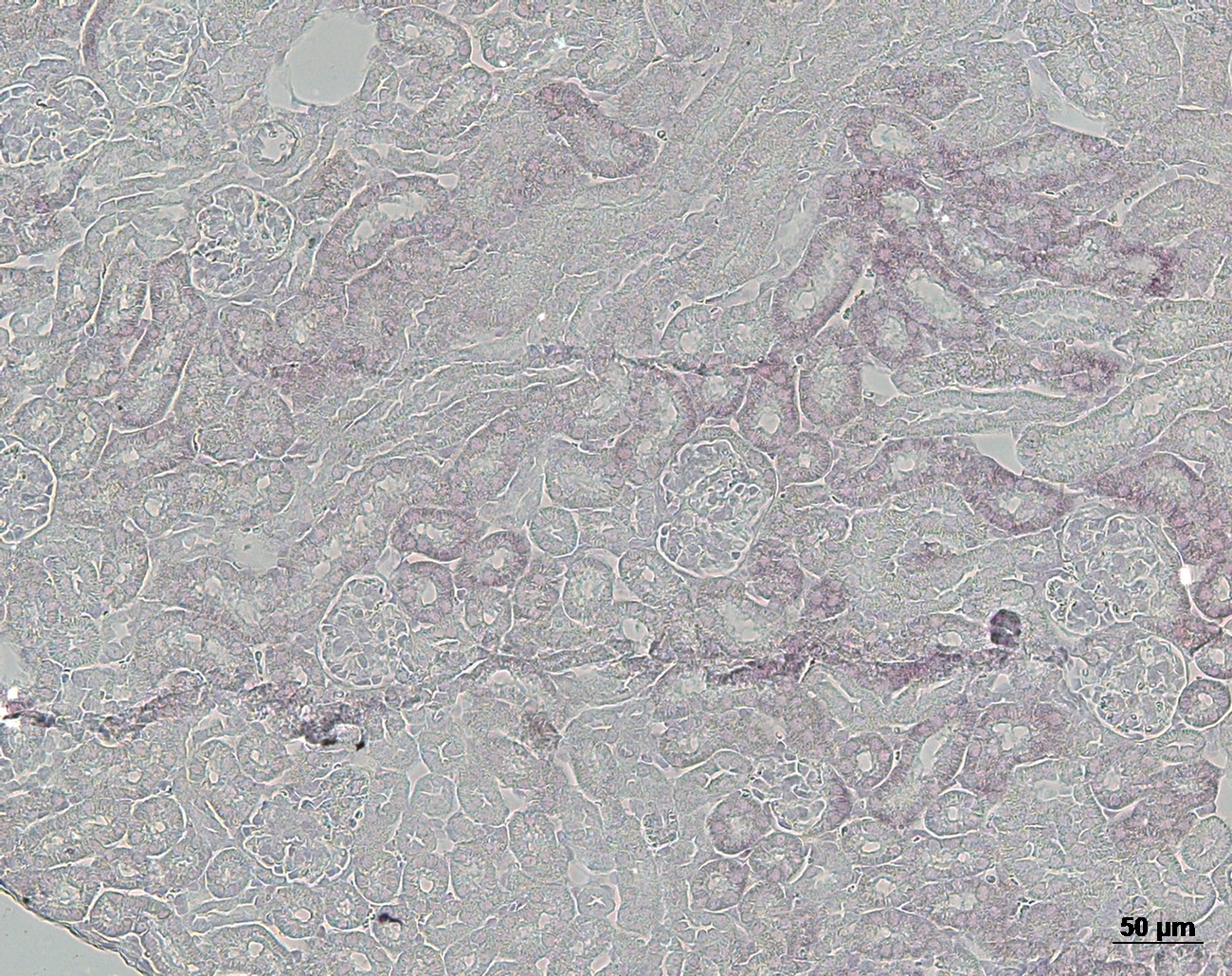

Supplement: Supplementary file 15 — Source data Fig. 7 [file 44321_2025_360_MOESM15_ESM.zip › EMM-2025-22130_SourceDataForFigure 7A-C 10-28-25/7B/Tsc1-cKIT dKO pRSK 20X.tif]

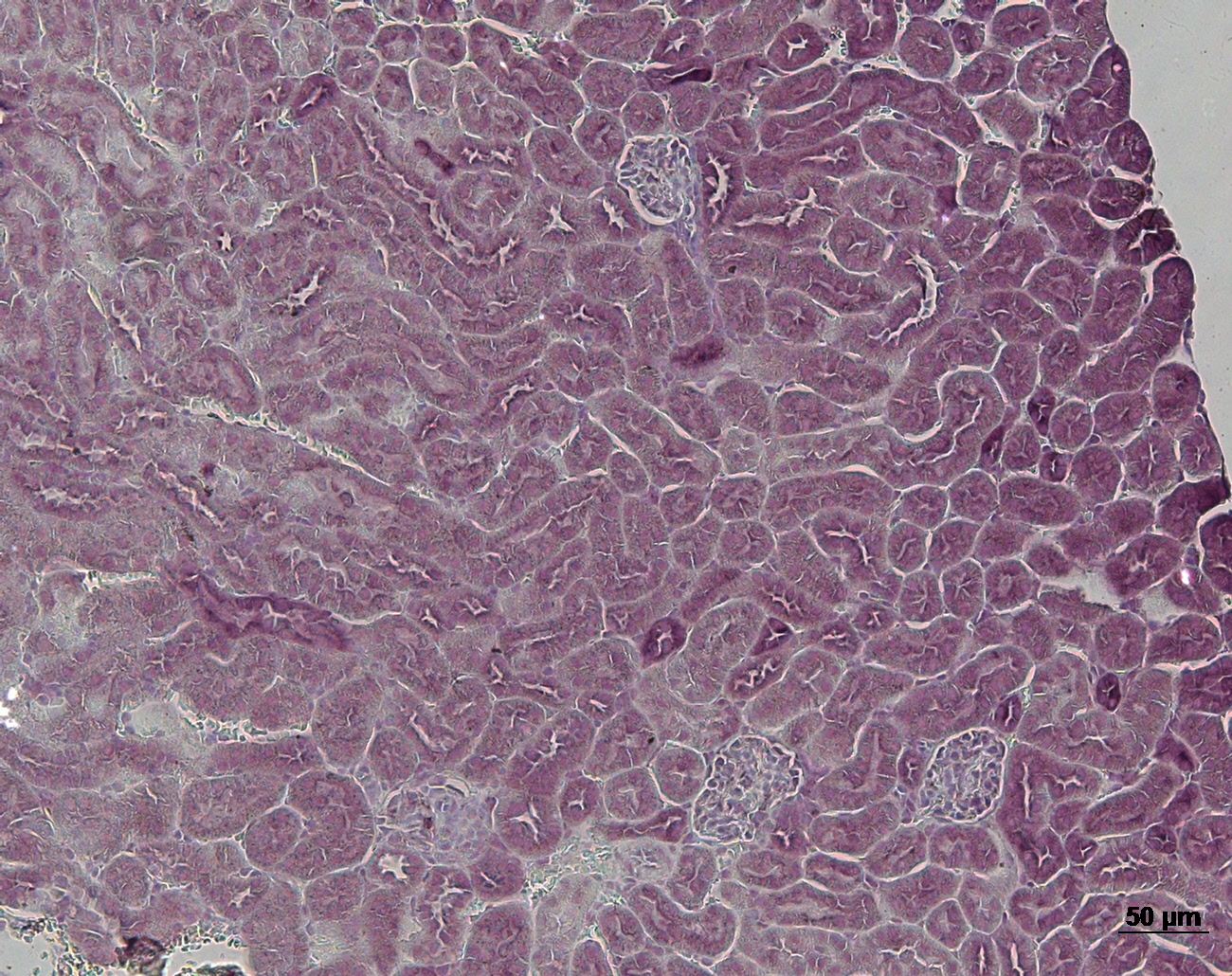

Supplement: Supplementary file 15 — Source data Fig. 7 [file 44321_2025_360_MOESM15_ESM.zip › EMM-2025-22130_SourceDataForFigure 7A-C 10-28-25/7B/WT pAKT 20X.tif]

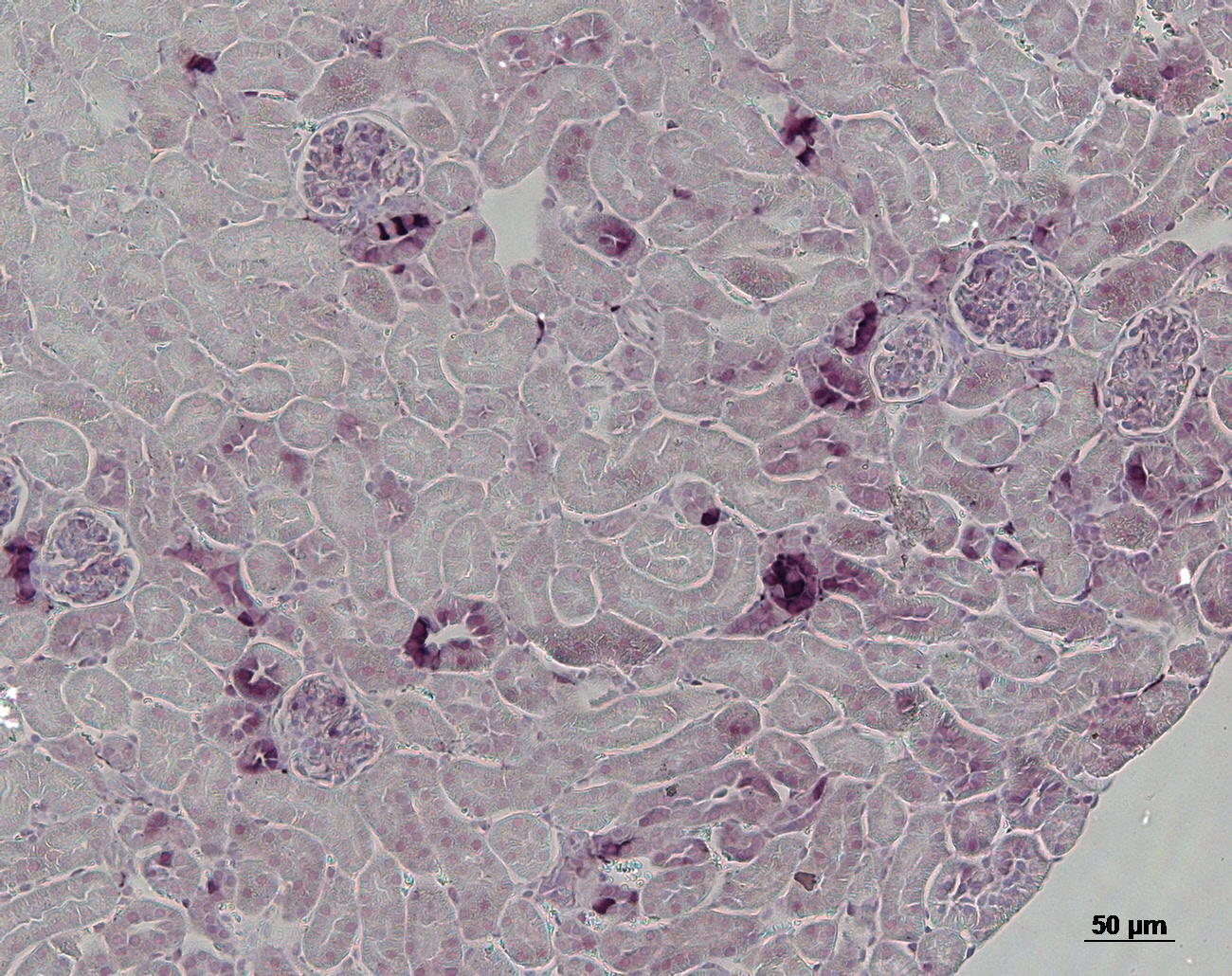

Supplement: Supplementary file 15 — Source data Fig. 7 [file 44321_2025_360_MOESM15_ESM.zip › EMM-2025-22130_SourceDataForFigure 7A-C 10-28-25/7B/WT pERK1-2 20X.tif]

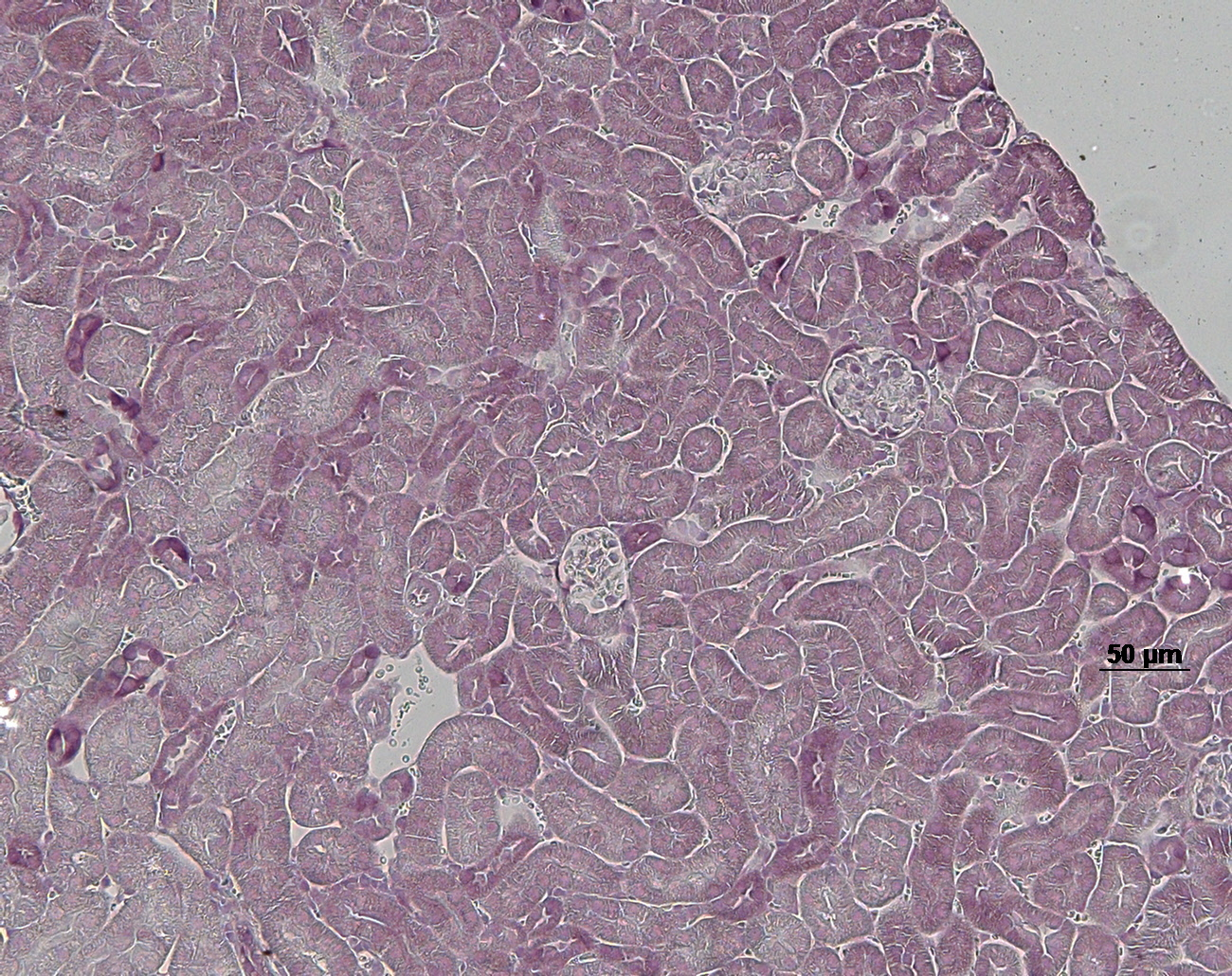

Supplement: Supplementary file 15 — Source data Fig. 7 [file 44321_2025_360_MOESM15_ESM.zip › EMM-2025-22130_SourceDataForFigure 7A-C 10-28-25/7B/WT pRSK 20X.tif]

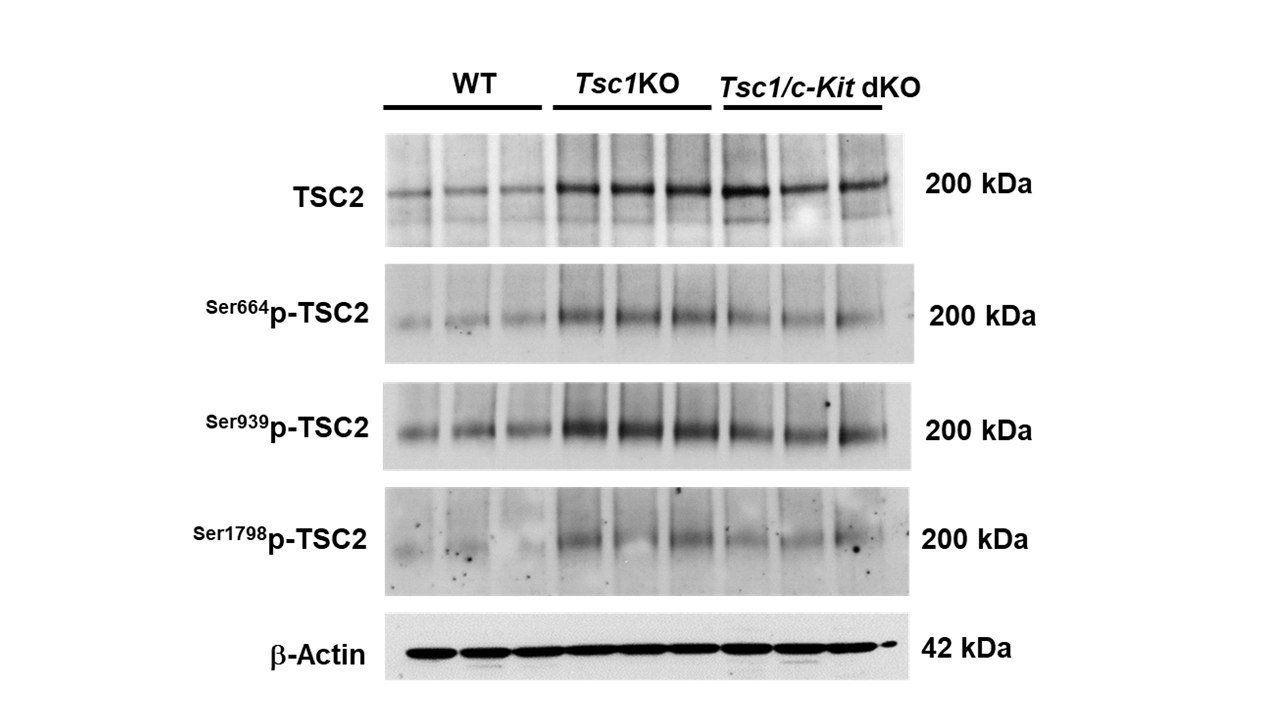

Supplement: Supplementary file 15 — Source data Fig. 7 [file 44321_2025_360_MOESM15_ESM.zip › EMM-2025-22130_SourceDataForFigure 7A-C 10-28-25/7C/Fig 7C Western Blot 10-29-25.tif]

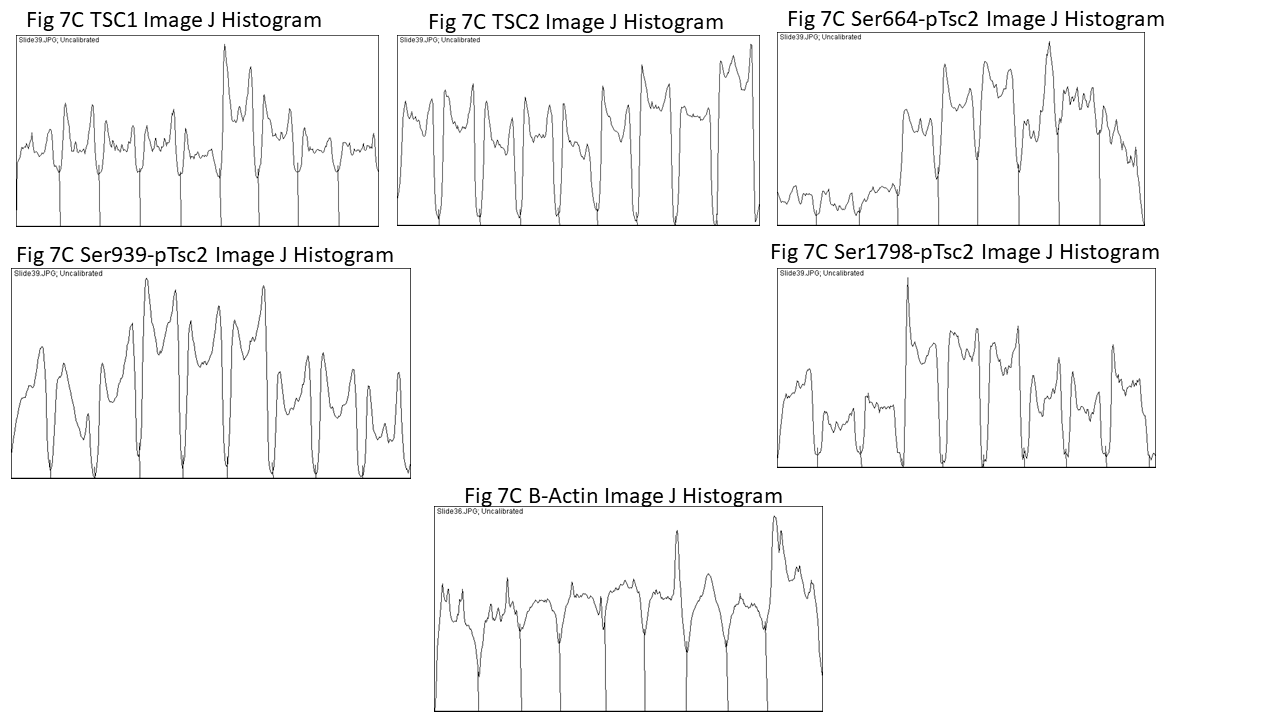

Supplement: Supplementary file 15 — Source data Fig. 7 [file 44321_2025_360_MOESM15_ESM.zip › EMM-2025-22130_SourceDataForFigure 7A-C 10-28-25/7C/Western Blot Image J Histograms/Slide1.TIF]

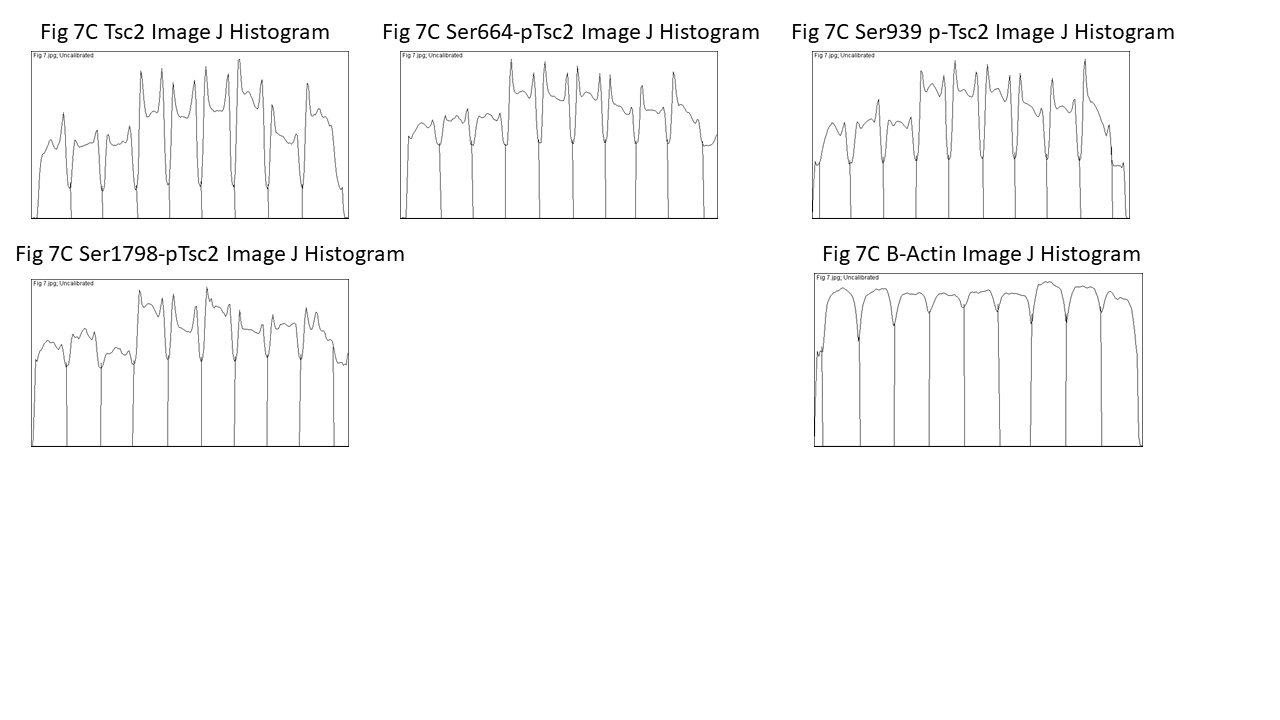

Supplement: Supplementary file 15 — Source data Fig. 7 [file 44321_2025_360_MOESM15_ESM.zip › EMM-2025-22130_SourceDataForFigure 7A-C 10-28-25/7C/Western Blot Image J Histograms/Slide2.TIF]

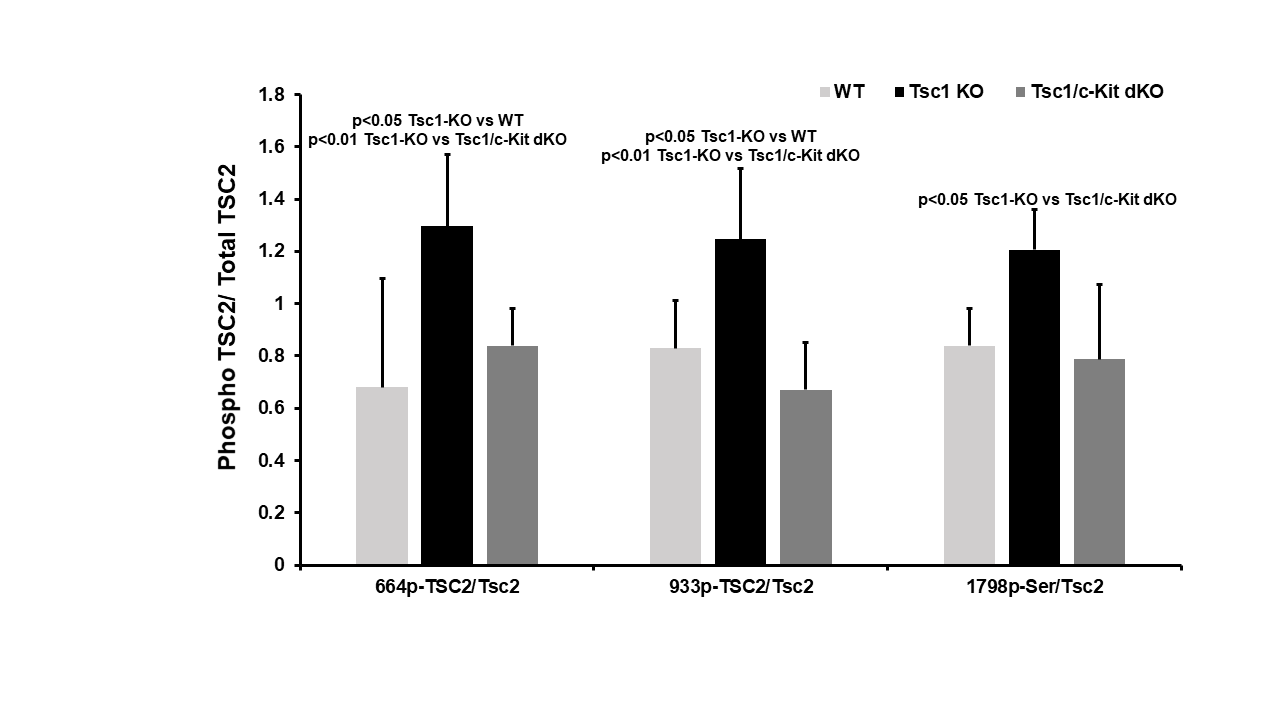

Supplement: Supplementary file 15 — Source data Fig. 7 [file 44321_2025_360_MOESM15_ESM.zip › EMM-2025-22130_SourceDataForFigure 7A-C 10-28-25/7C/Western Blot Quantification.tif]

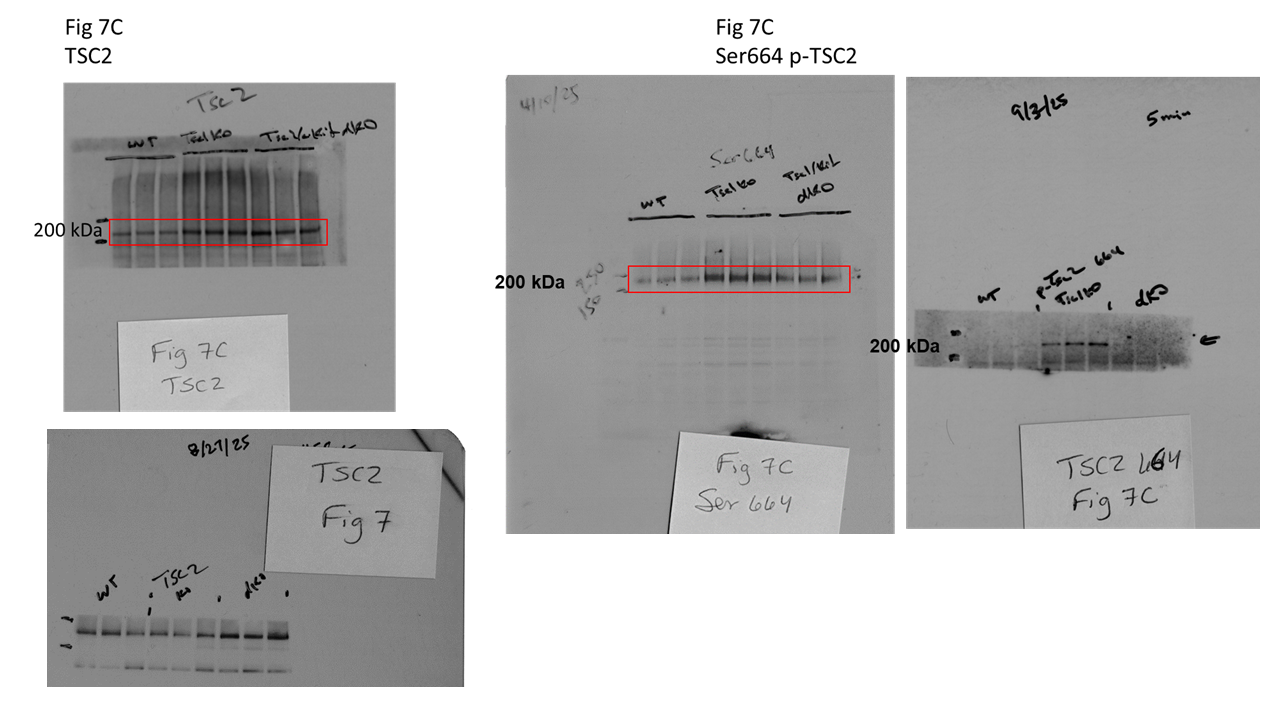

Supplement: Supplementary file 15 — Source data Fig. 7 [file 44321_2025_360_MOESM15_ESM.zip › EMM-2025-22130_SourceDataForFigure 7A-C 10-28-25/7C/Western Blot Source Data 10-29-25/Slide1.TIF]

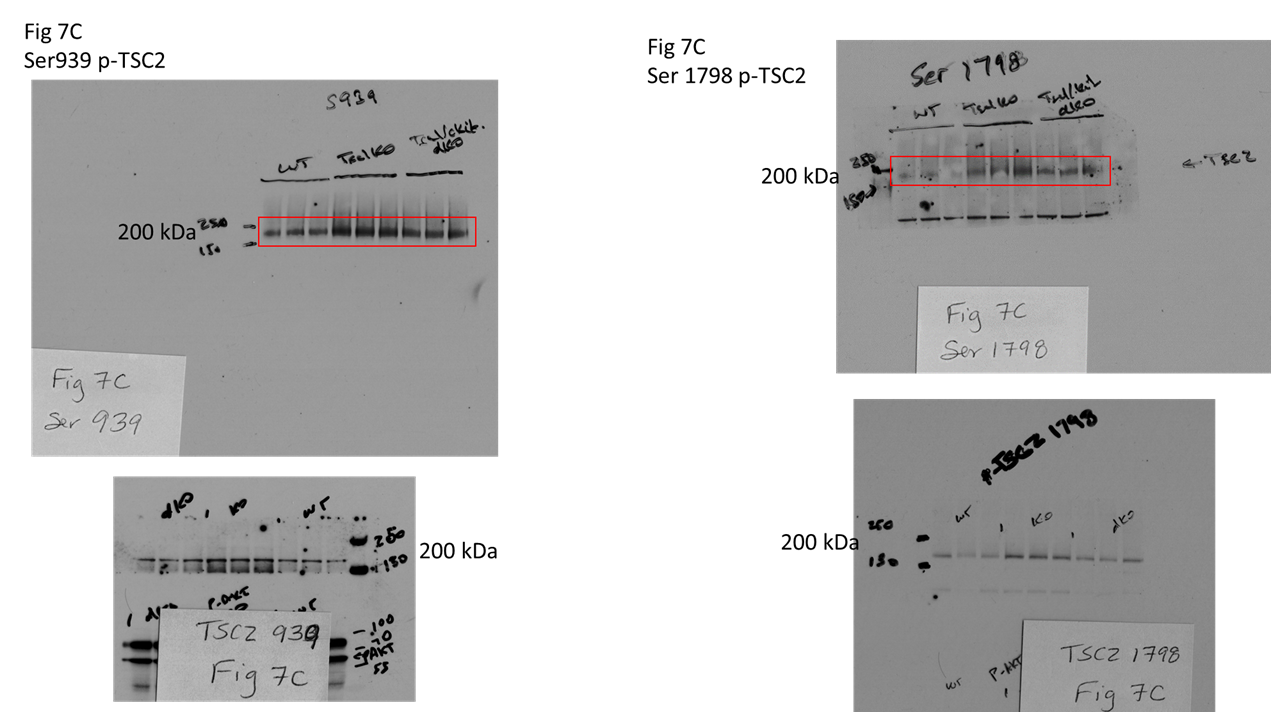

Supplement: Supplementary file 15 — Source data Fig. 7 [file 44321_2025_360_MOESM15_ESM.zip › EMM-2025-22130_SourceDataForFigure 7A-C 10-28-25/7C/Western Blot Source Data 10-29-25/Slide2.TIF]

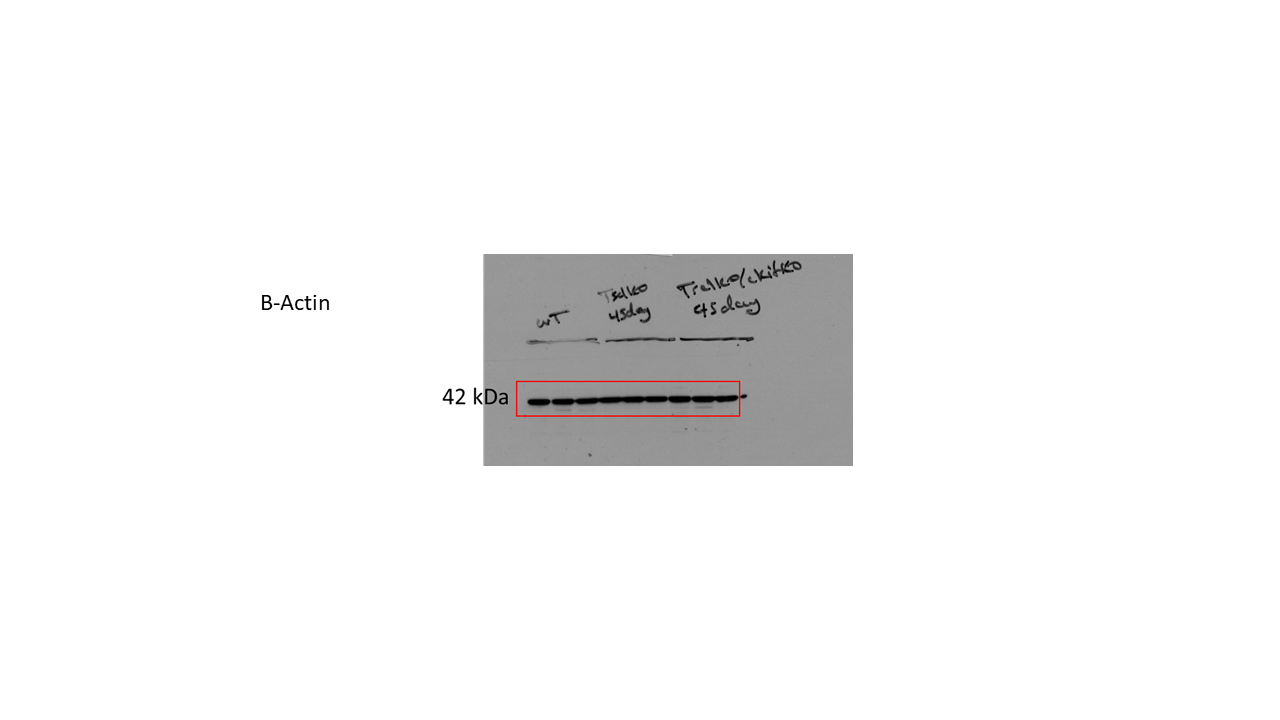

Supplement: Supplementary file 15 — Source data Fig. 7 [file 44321_2025_360_MOESM15_ESM.zip › EMM-2025-22130_SourceDataForFigure 7A-C 10-28-25/7C/Western Blot Source Data 10-29-25/Slide3.TIF]

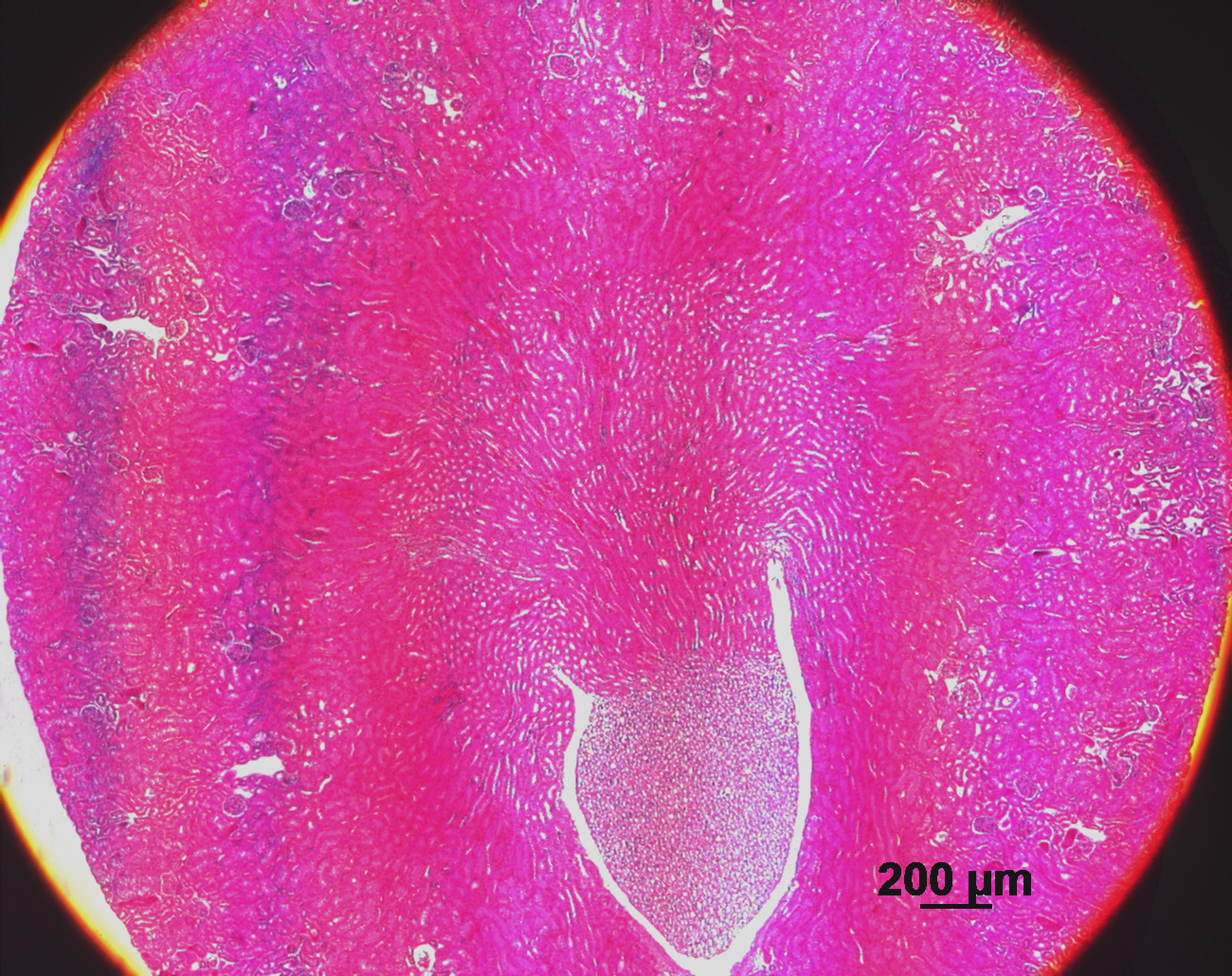

Supplement: Supplementary file 16 — Source data Fig. 8 [file 44321_2025_360_MOESM16_ESM.zip › EMM-2025-22130_SourceDataForFigure 8A-E 10-28-25/8A/Tsc1 KO Imatinib 4X.tif]

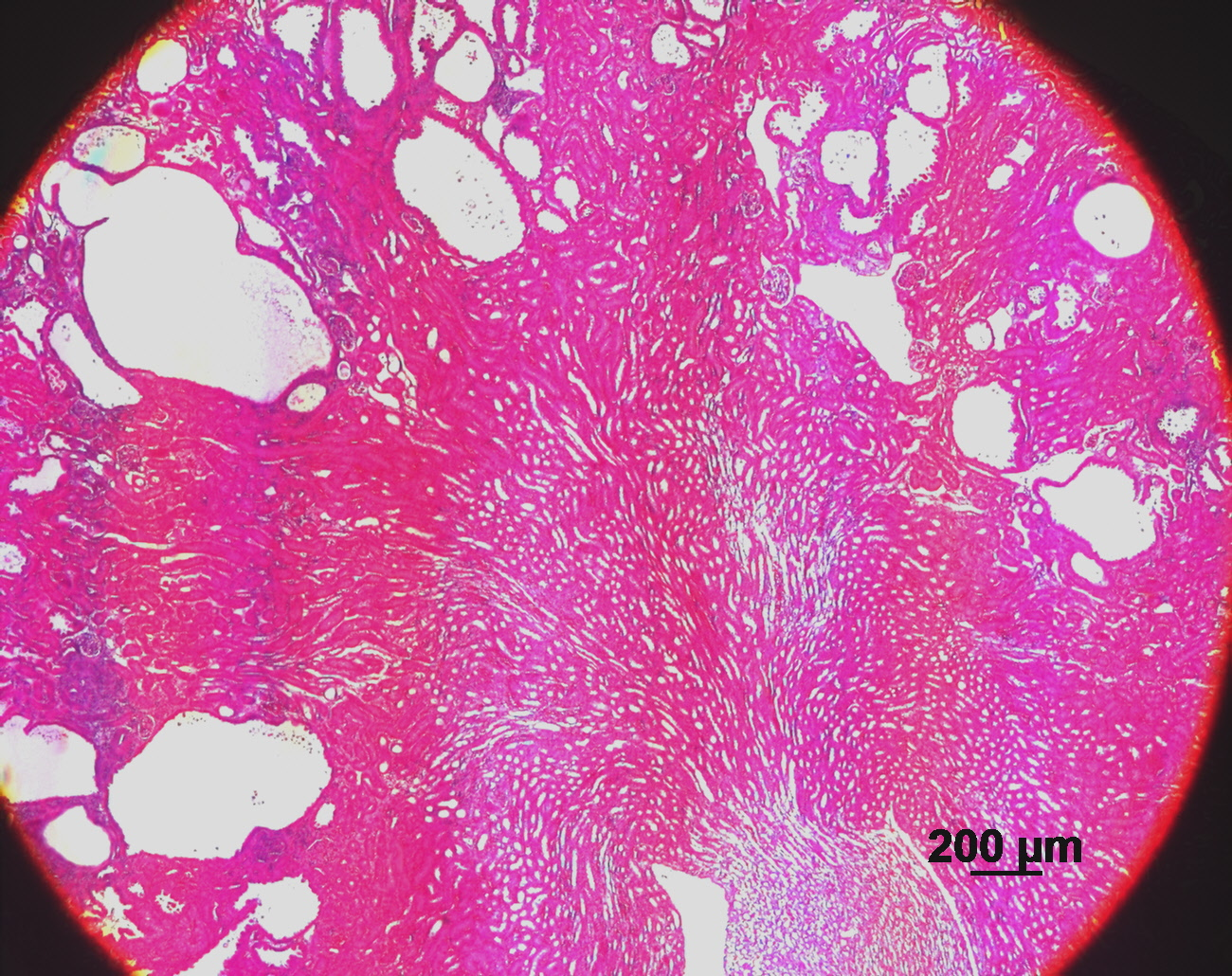

Supplement: Supplementary file 16 — Source data Fig. 8 [file 44321_2025_360_MOESM16_ESM.zip › EMM-2025-22130_SourceDataForFigure 8A-E 10-28-25/8A/Tsc1 KO Untreated 4X.tif]

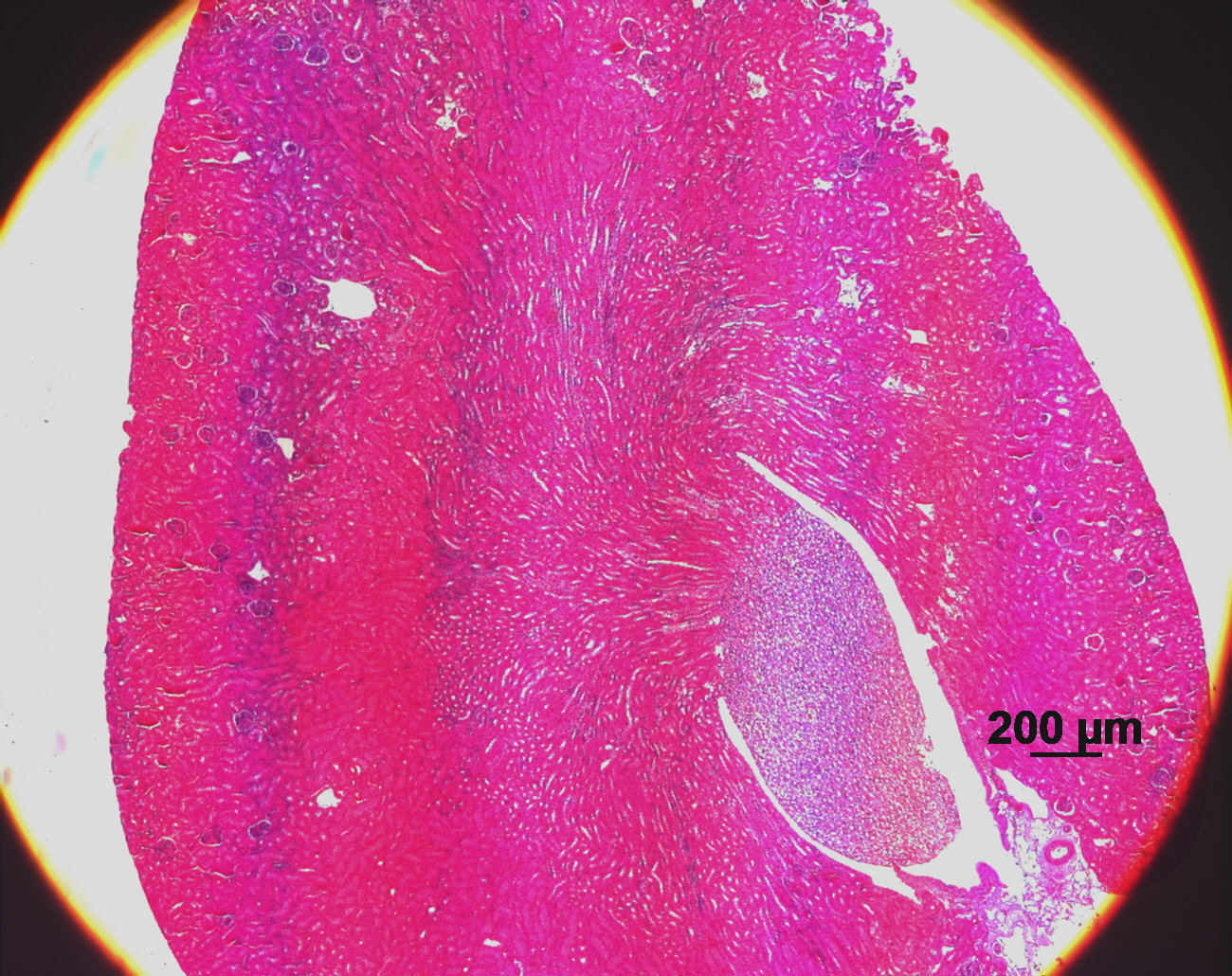

Supplement: Supplementary file 16 — Source data Fig. 8 [file 44321_2025_360_MOESM16_ESM.zip › EMM-2025-22130_SourceDataForFigure 8A-E 10-28-25/8A/WT Imatinib 4X.tif]

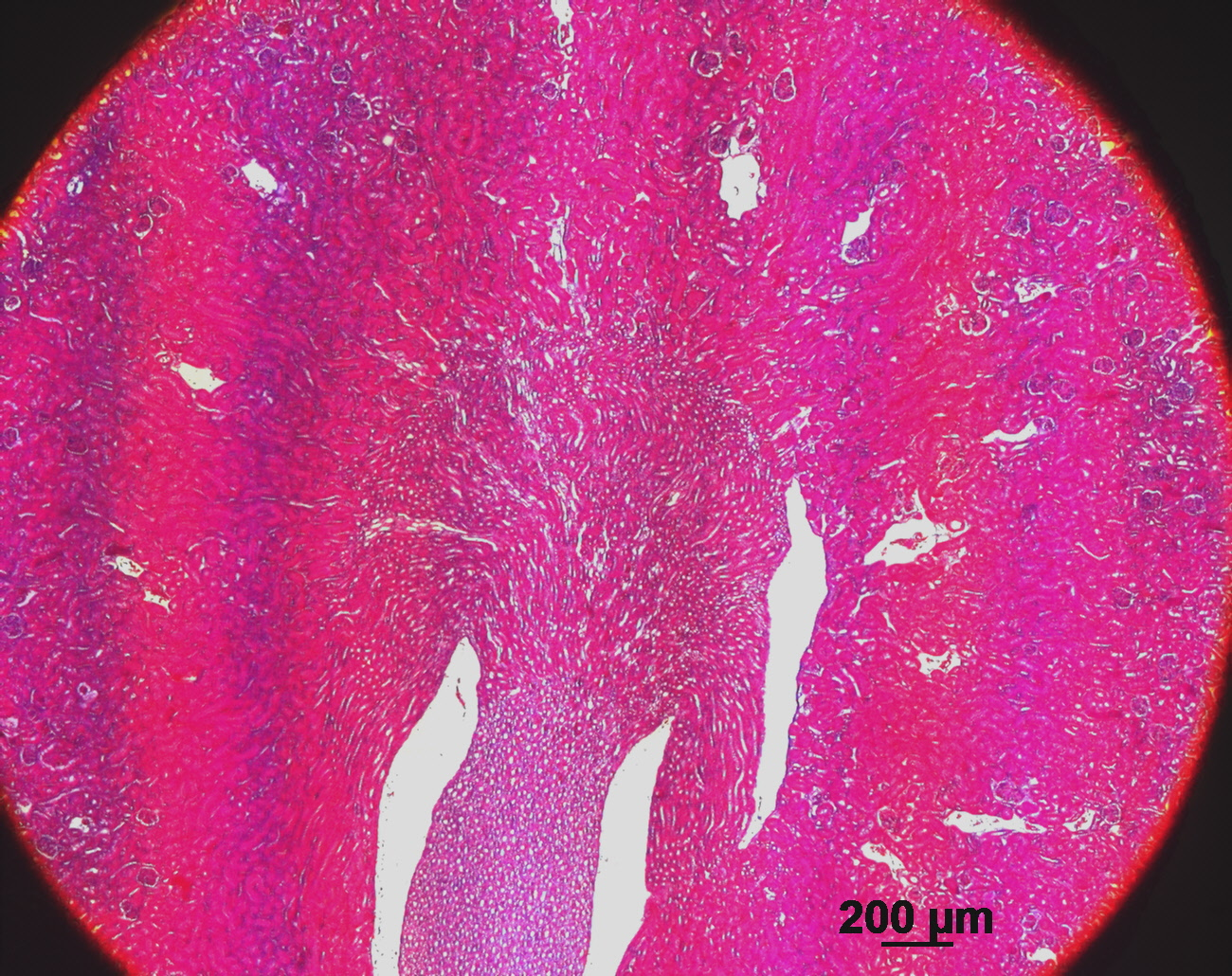

Supplement: Supplementary file 16 — Source data Fig. 8 [file 44321_2025_360_MOESM16_ESM.zip › EMM-2025-22130_SourceDataForFigure 8A-E 10-28-25/8A/WT Untreated 4X.tif]

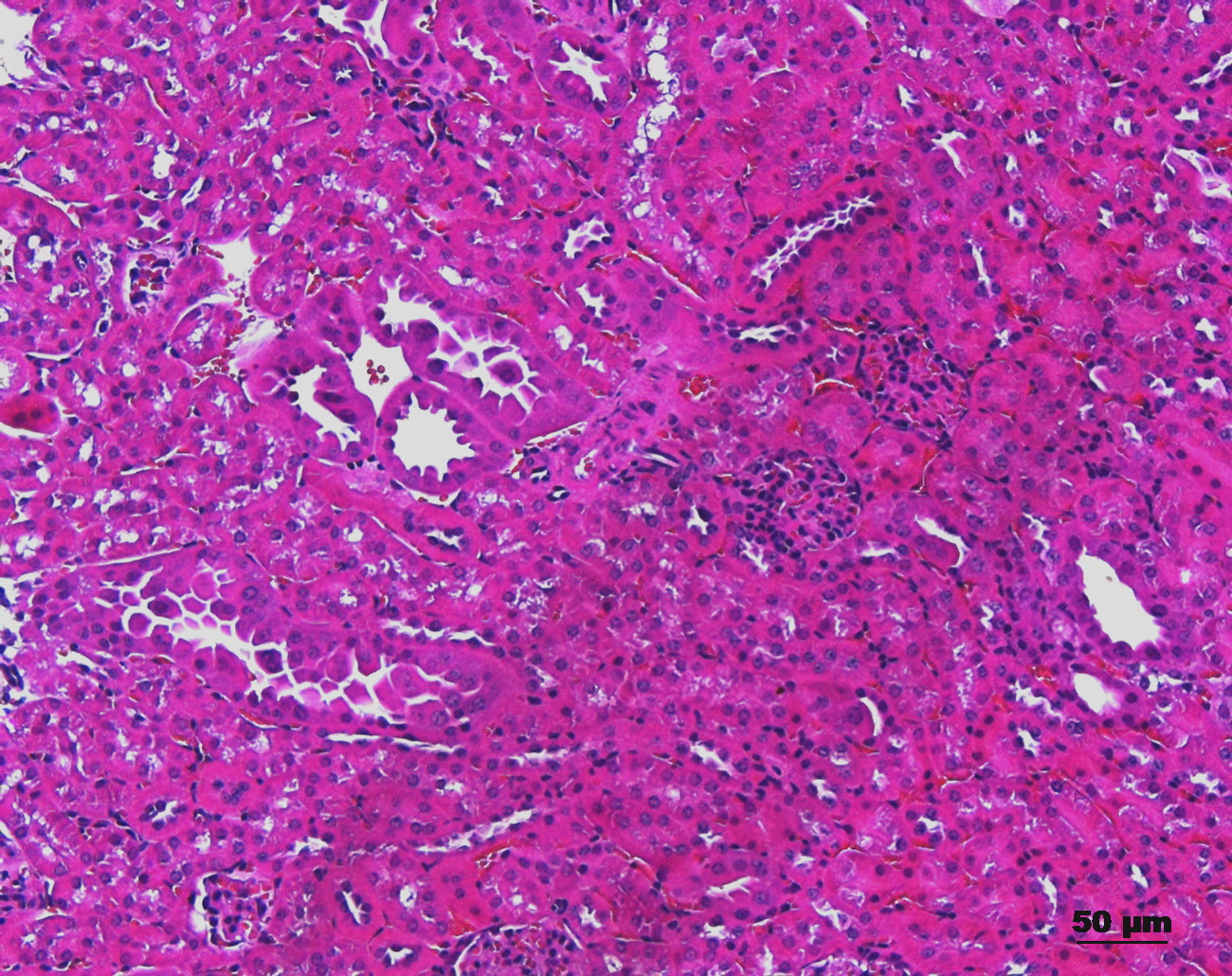

Supplement: Supplementary file 16 — Source data Fig. 8 [file 44321_2025_360_MOESM16_ESM.zip › EMM-2025-22130_SourceDataForFigure 8A-E 10-28-25/8B/Tsc1 KO Imatinib 20X #2.tif]

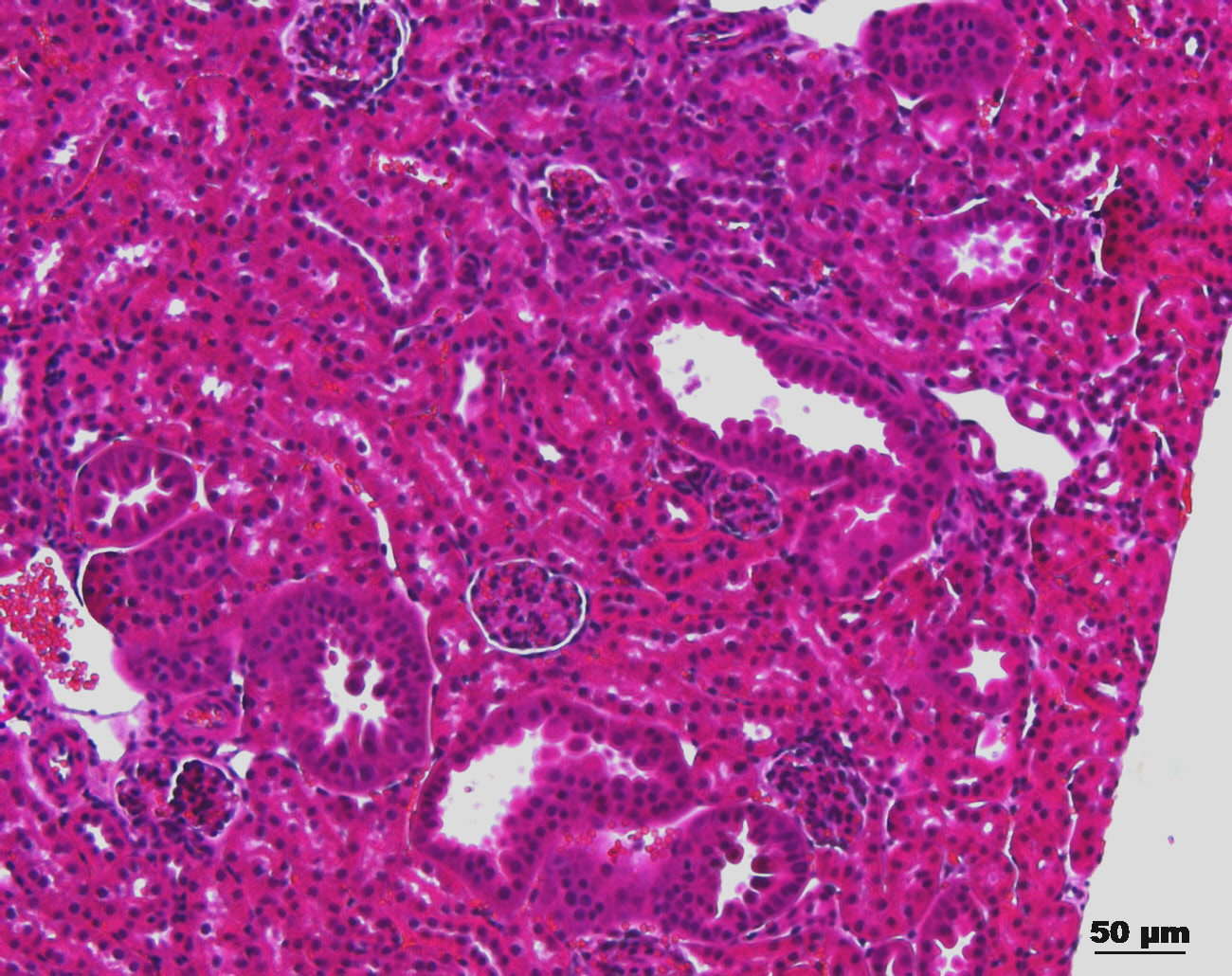

Supplement: Supplementary file 16 — Source data Fig. 8 [file 44321_2025_360_MOESM16_ESM.zip › EMM-2025-22130_SourceDataForFigure 8A-E 10-28-25/8B/Tsc1 KO Imatinib 20X.tif]

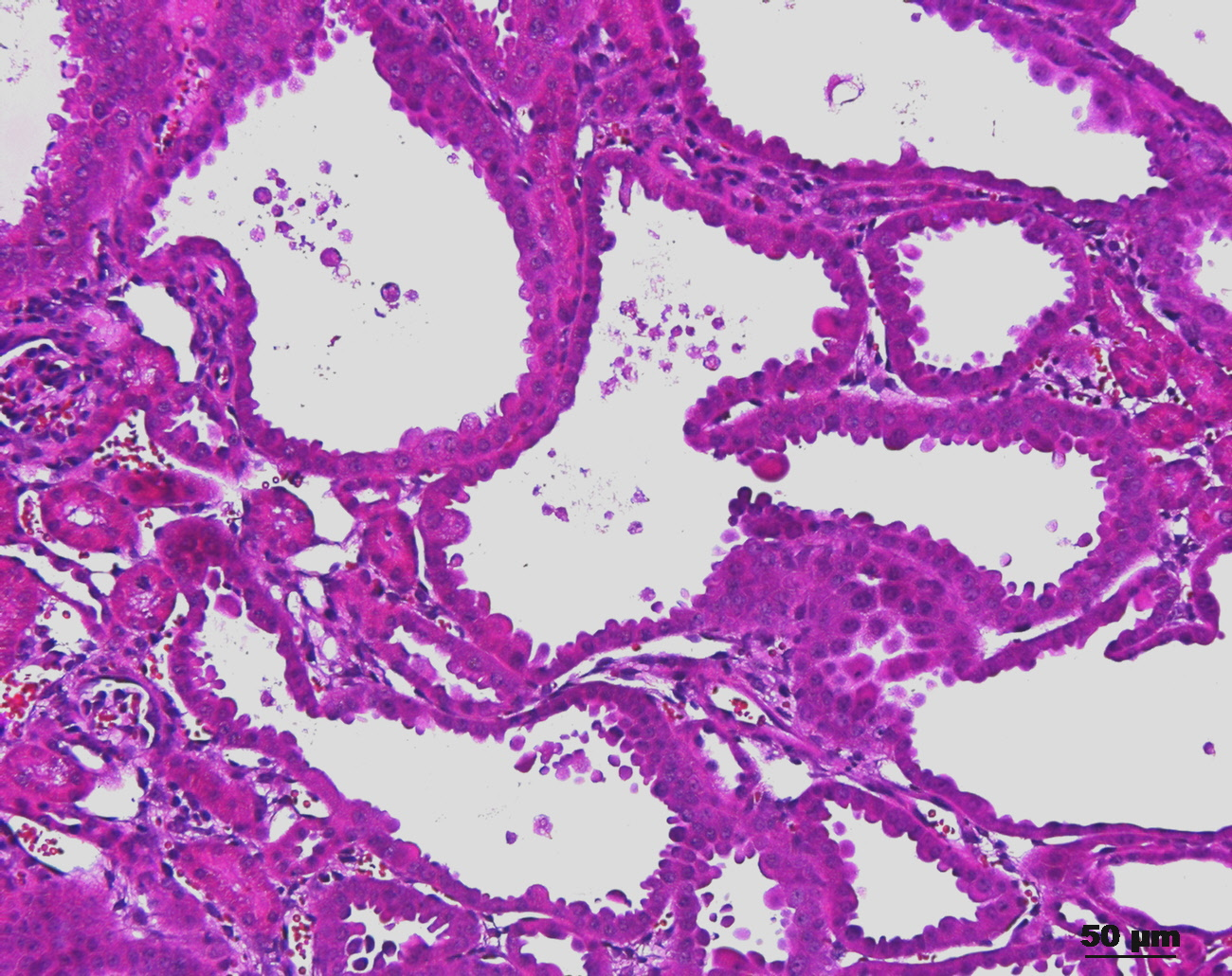

Supplement: Supplementary file 16 — Source data Fig. 8 [file 44321_2025_360_MOESM16_ESM.zip › EMM-2025-22130_SourceDataForFigure 8A-E 10-28-25/8B/Tsc1 KO Untreated 20X.tif]

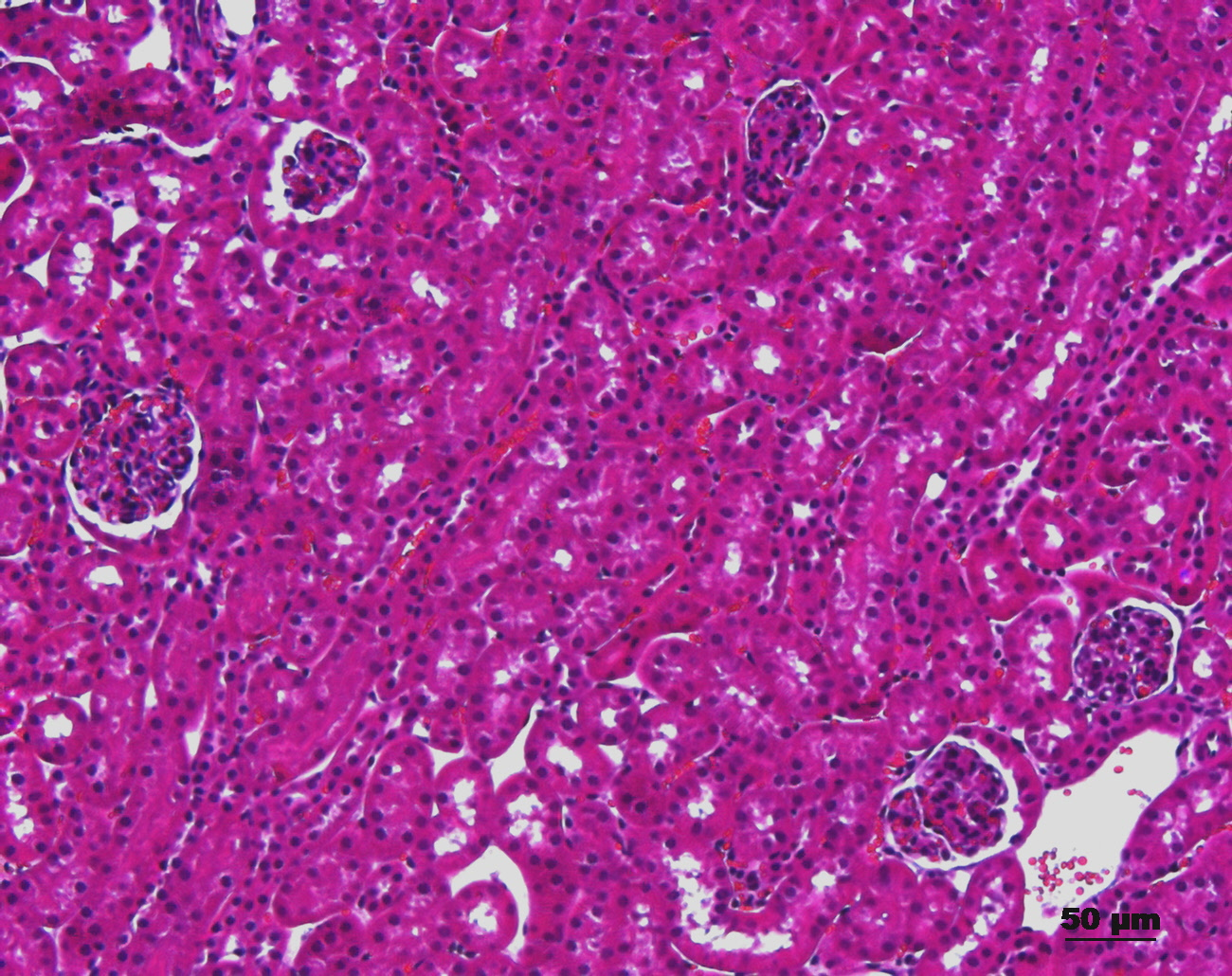

Supplement: Supplementary file 16 — Source data Fig. 8 [file 44321_2025_360_MOESM16_ESM.zip › EMM-2025-22130_SourceDataForFigure 8A-E 10-28-25/8B/WT Imatinib 20X.tif]

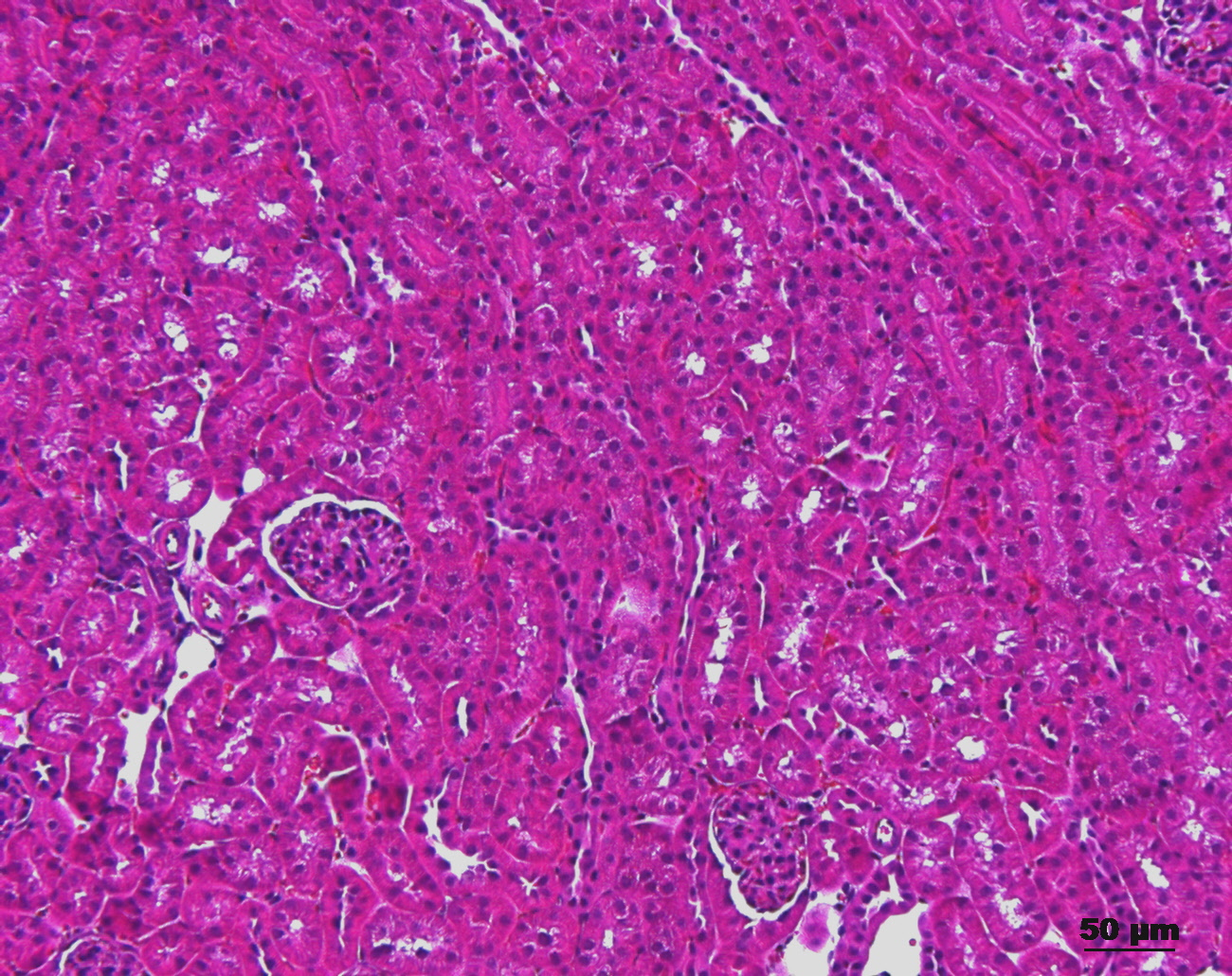

Supplement: Supplementary file 16 — Source data Fig. 8 [file 44321_2025_360_MOESM16_ESM.zip › EMM-2025-22130_SourceDataForFigure 8A-E 10-28-25/8B/WT Untreated 20X.tif]

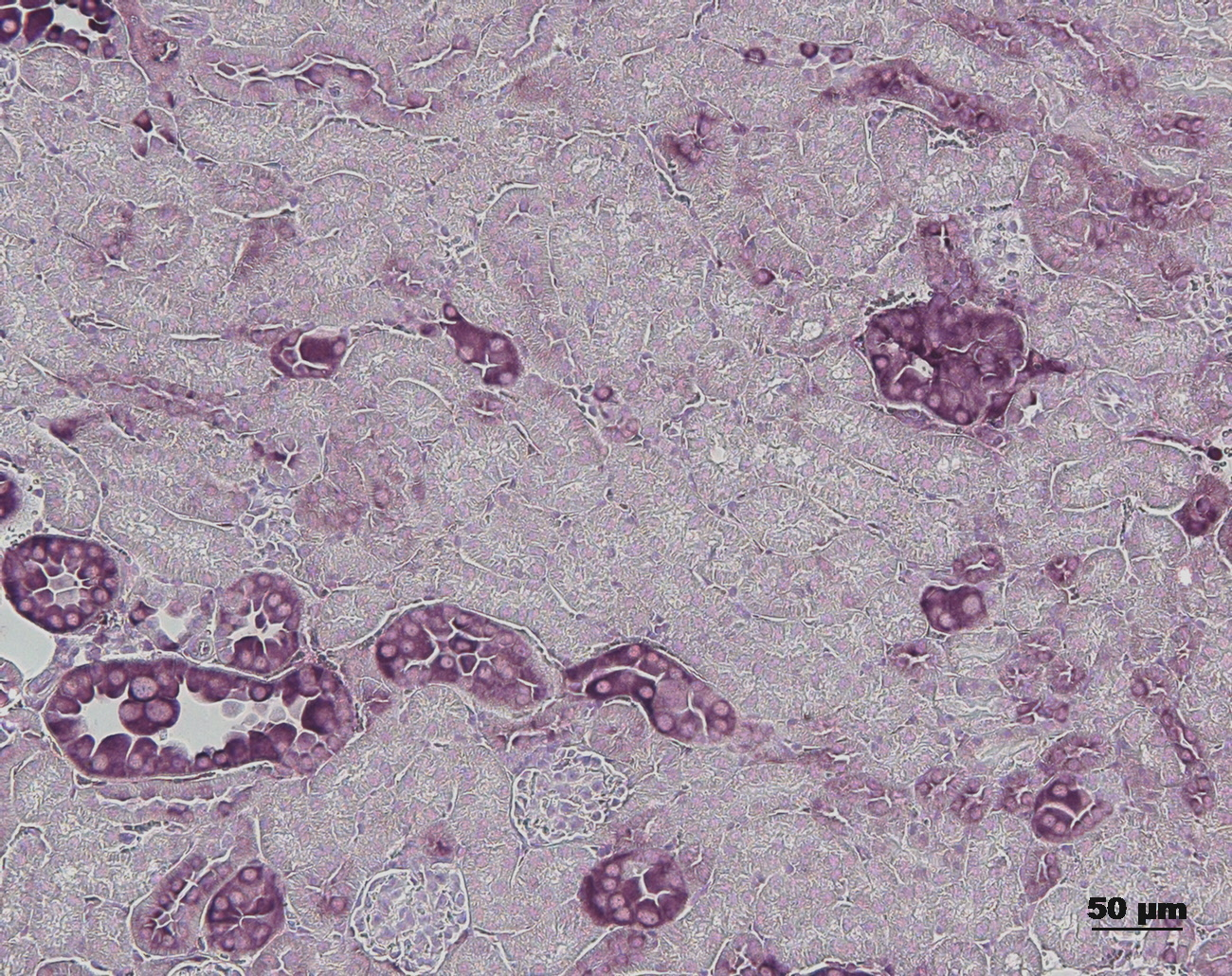

Supplement: Supplementary file 16 — Source data Fig. 8 [file 44321_2025_360_MOESM16_ESM.zip › EMM-2025-22130_SourceDataForFigure 8A-E 10-28-25/8C/Tsc1 KO Imatinib pS6 20X #2.tif]

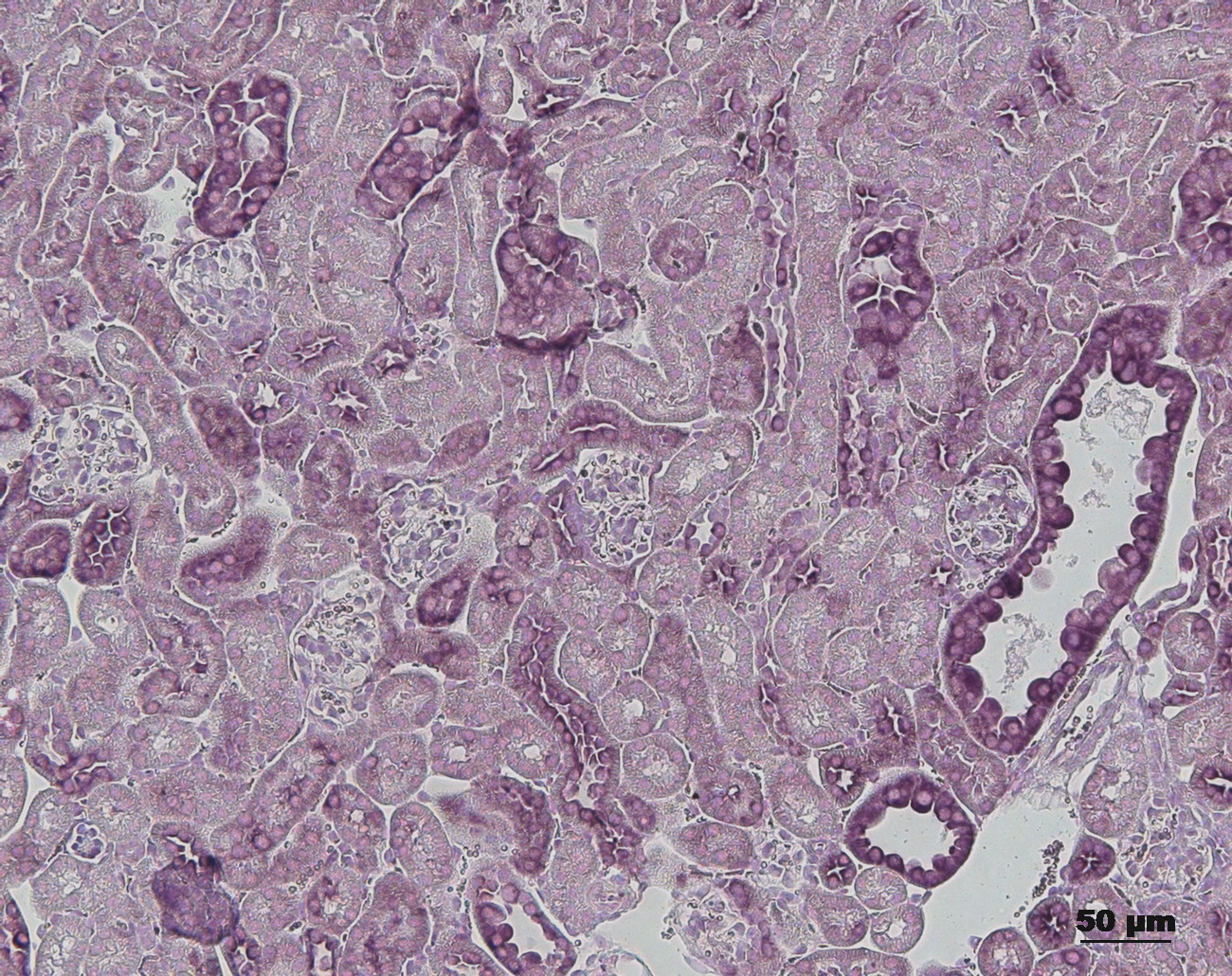

Supplement: Supplementary file 16 — Source data Fig. 8 [file 44321_2025_360_MOESM16_ESM.zip › EMM-2025-22130_SourceDataForFigure 8A-E 10-28-25/8C/Tsc1 KO Imatinib pS6 20X.tif]

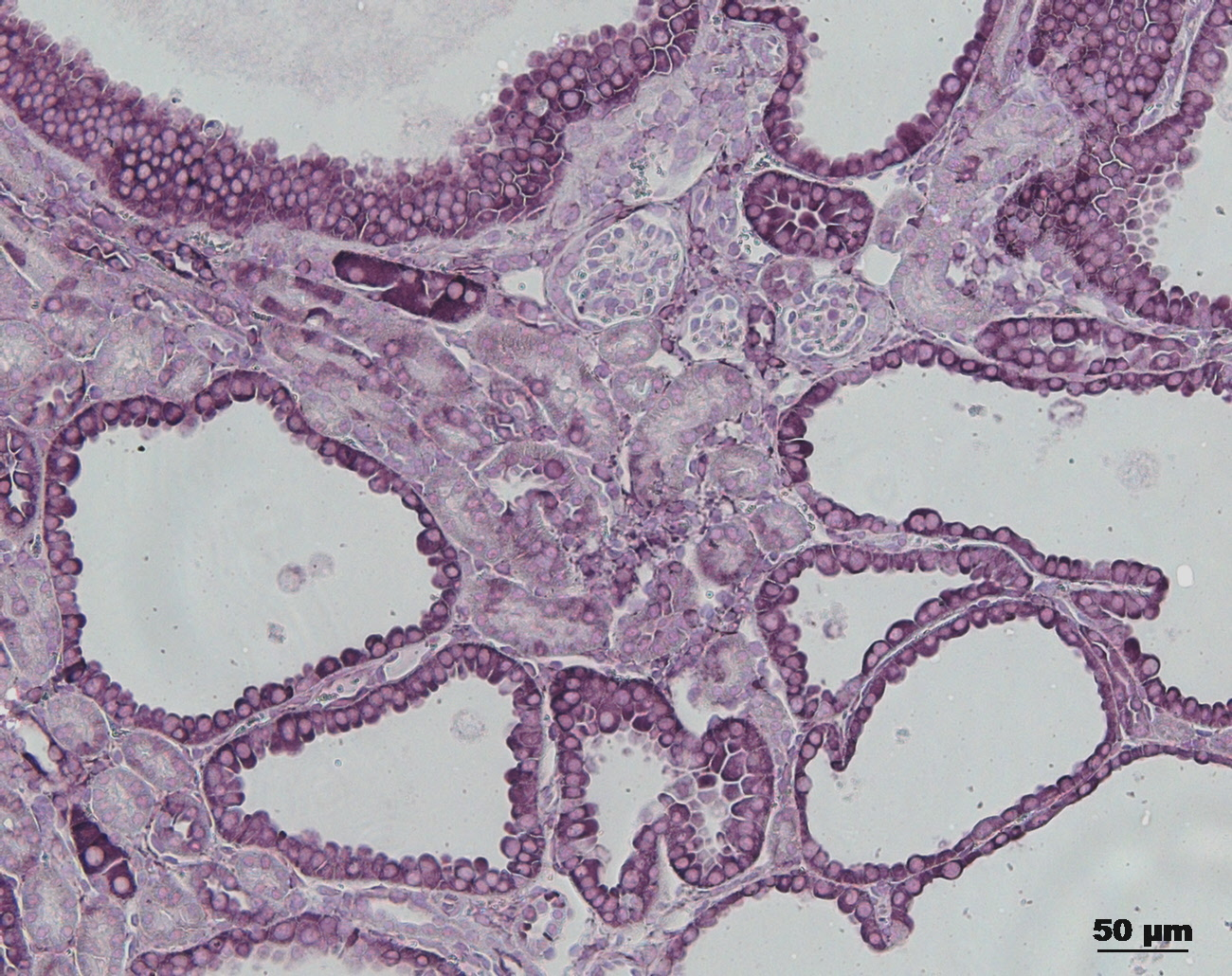

Supplement: Supplementary file 16 — Source data Fig. 8 [file 44321_2025_360_MOESM16_ESM.zip › EMM-2025-22130_SourceDataForFigure 8A-E 10-28-25/8C/Tsc1 KO Untreated pS6 20X.tif]

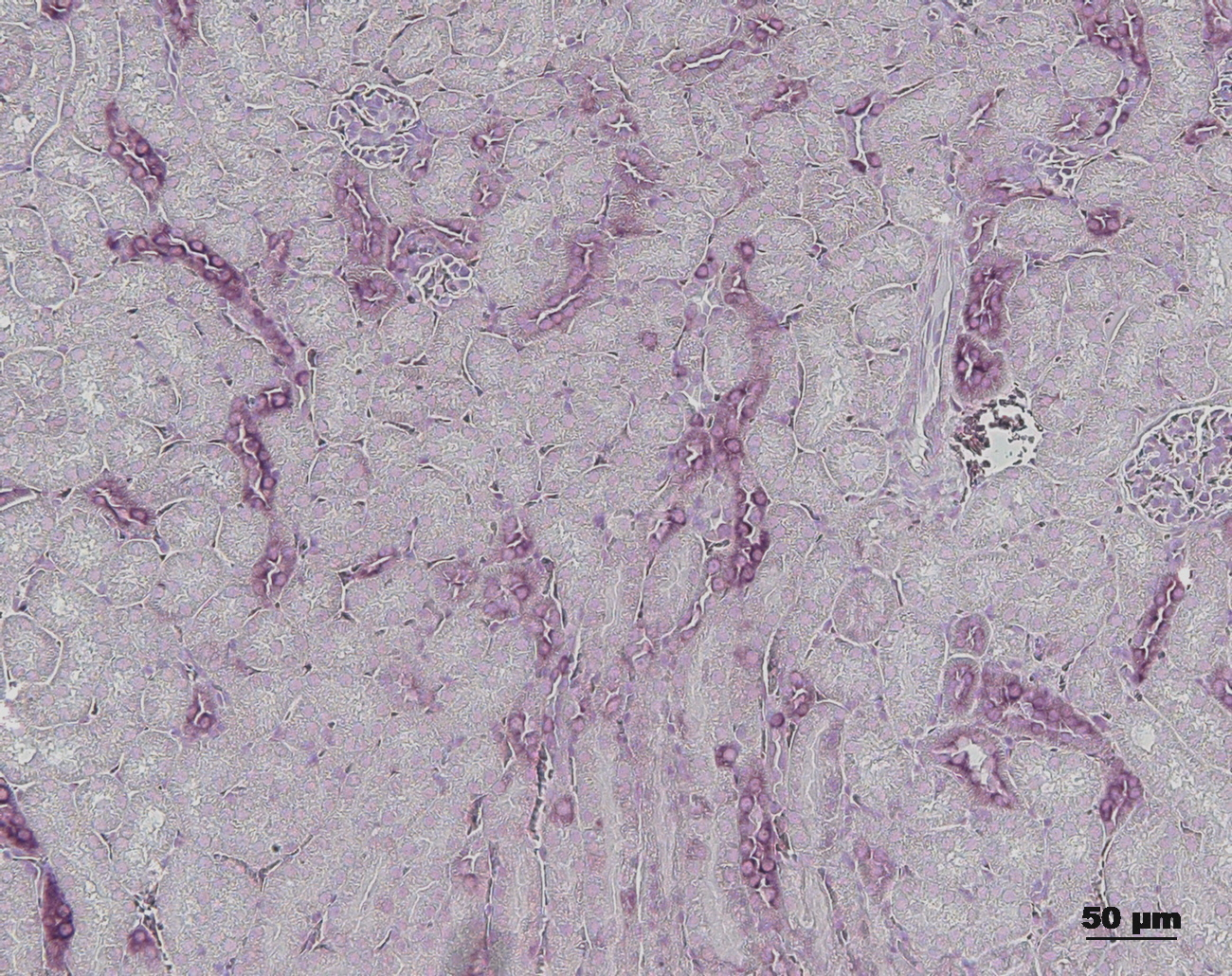

Supplement: Supplementary file 16 — Source data Fig. 8 [file 44321_2025_360_MOESM16_ESM.zip › EMM-2025-22130_SourceDataForFigure 8A-E 10-28-25/8C/WT Imatinib pS6 20X.tif]

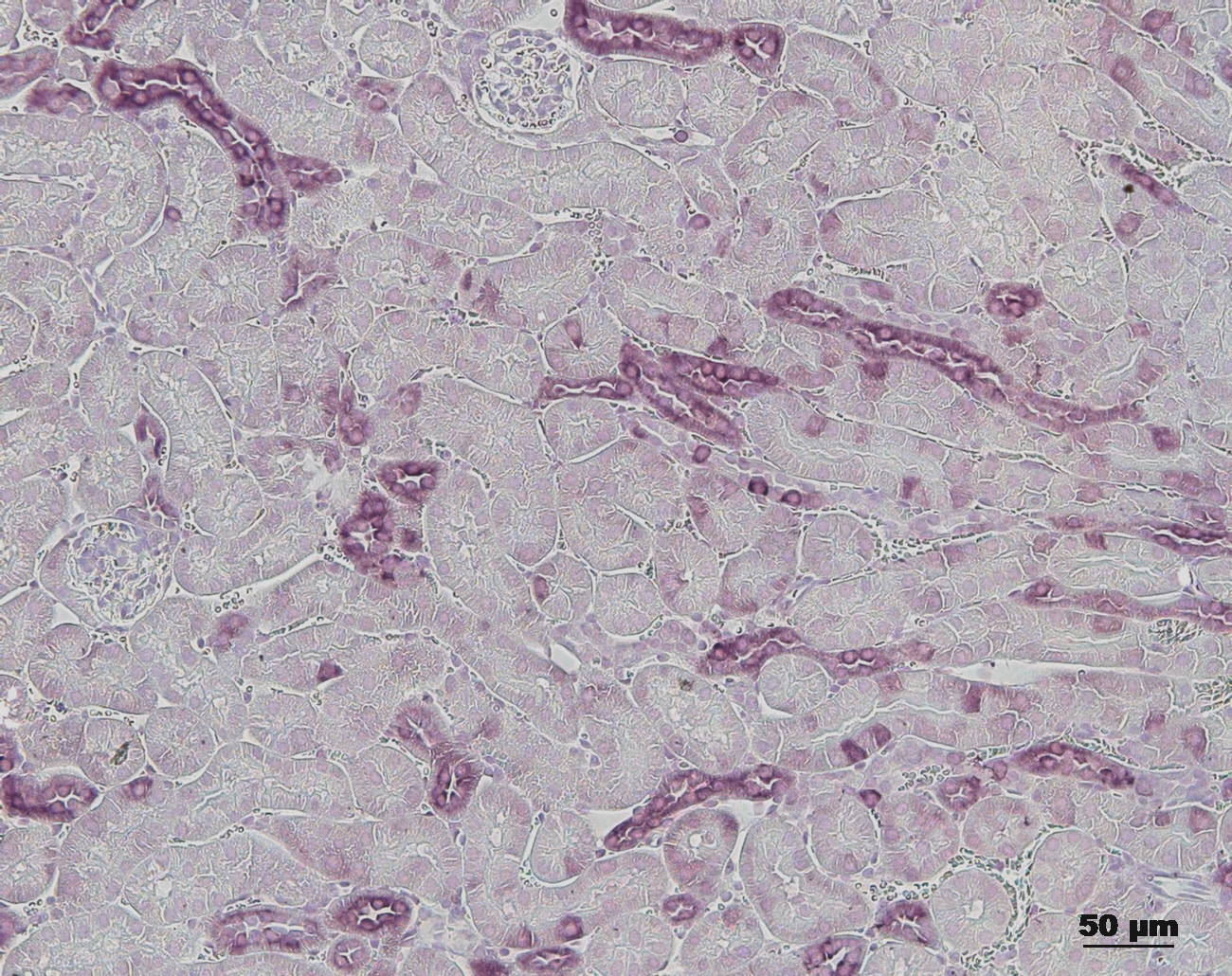

Supplement: Supplementary file 16 — Source data Fig. 8 [file 44321_2025_360_MOESM16_ESM.zip › EMM-2025-22130_SourceDataForFigure 8A-E 10-28-25/8C/WT Untreated pS6 20X.tif]

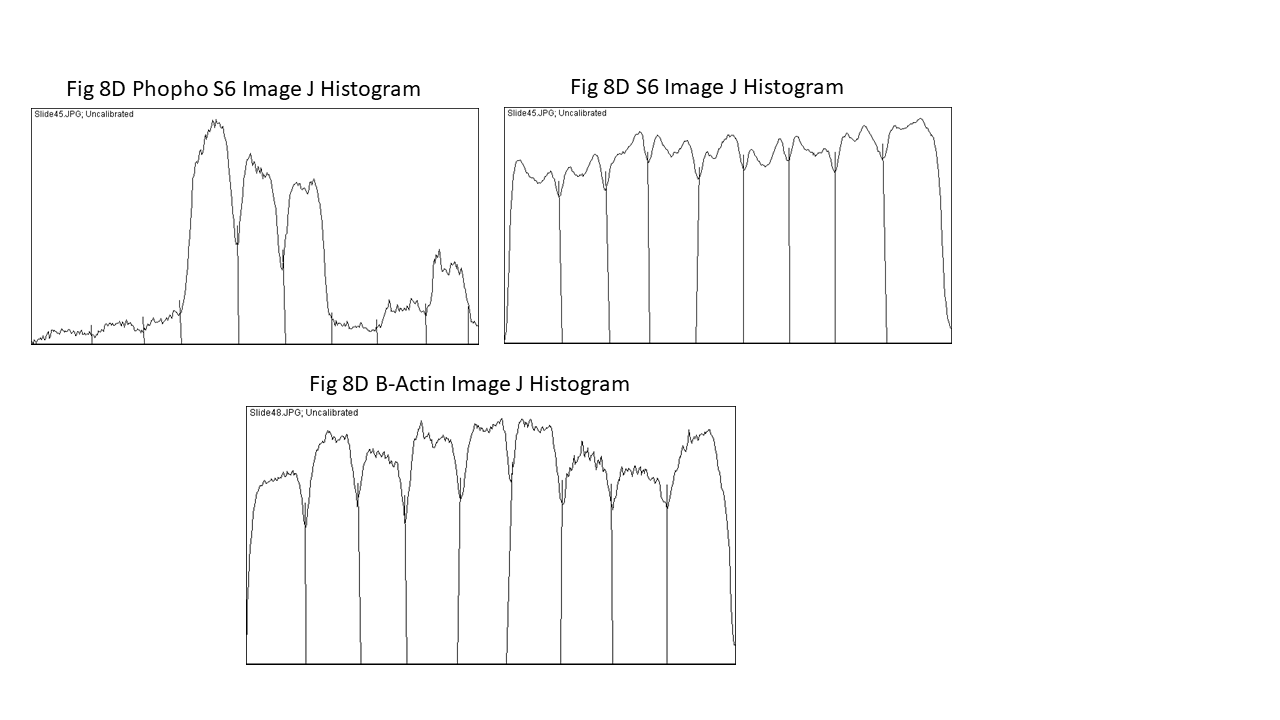

Supplement: Supplementary file 16 — Source data Fig. 8 [file 44321_2025_360_MOESM16_ESM.zip › EMM-2025-22130_SourceDataForFigure 8A-E 10-28-25/8D/Western Blot Image J Histograms.tif]

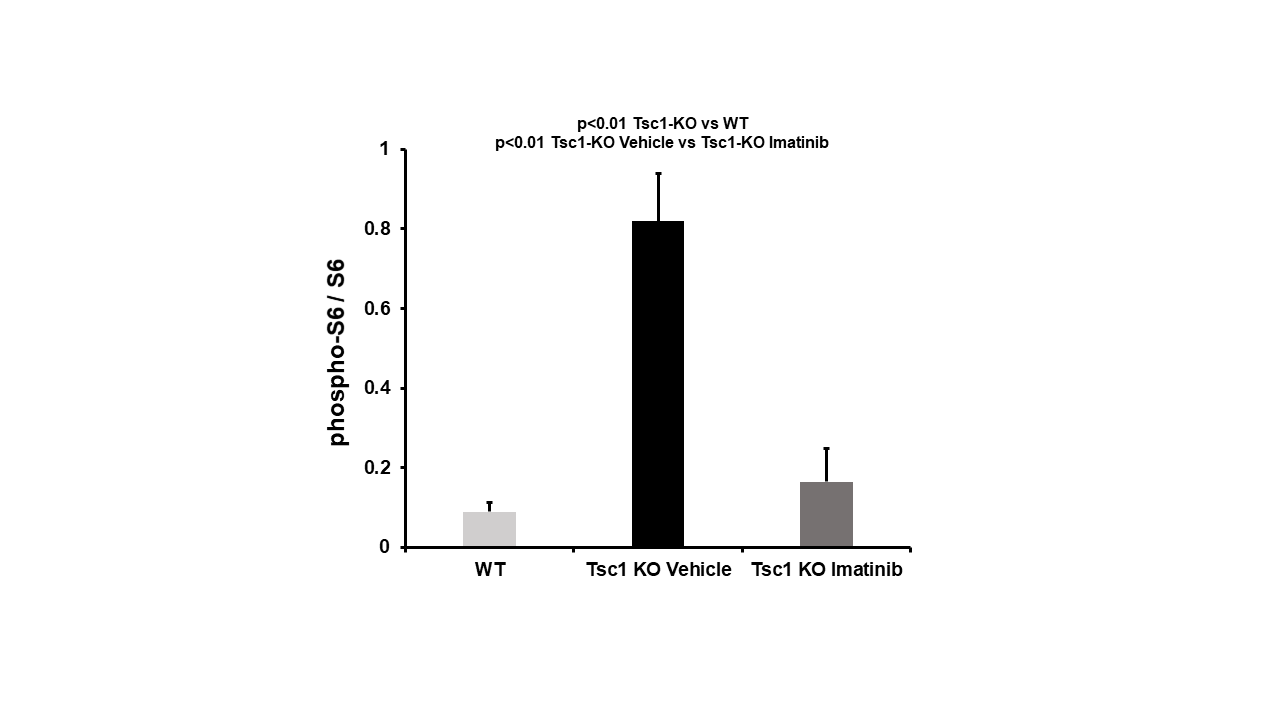

Supplement: Supplementary file 16 — Source data Fig. 8 [file 44321_2025_360_MOESM16_ESM.zip › EMM-2025-22130_SourceDataForFigure 8A-E 10-28-25/8D/Western Blot Quantification.tif]

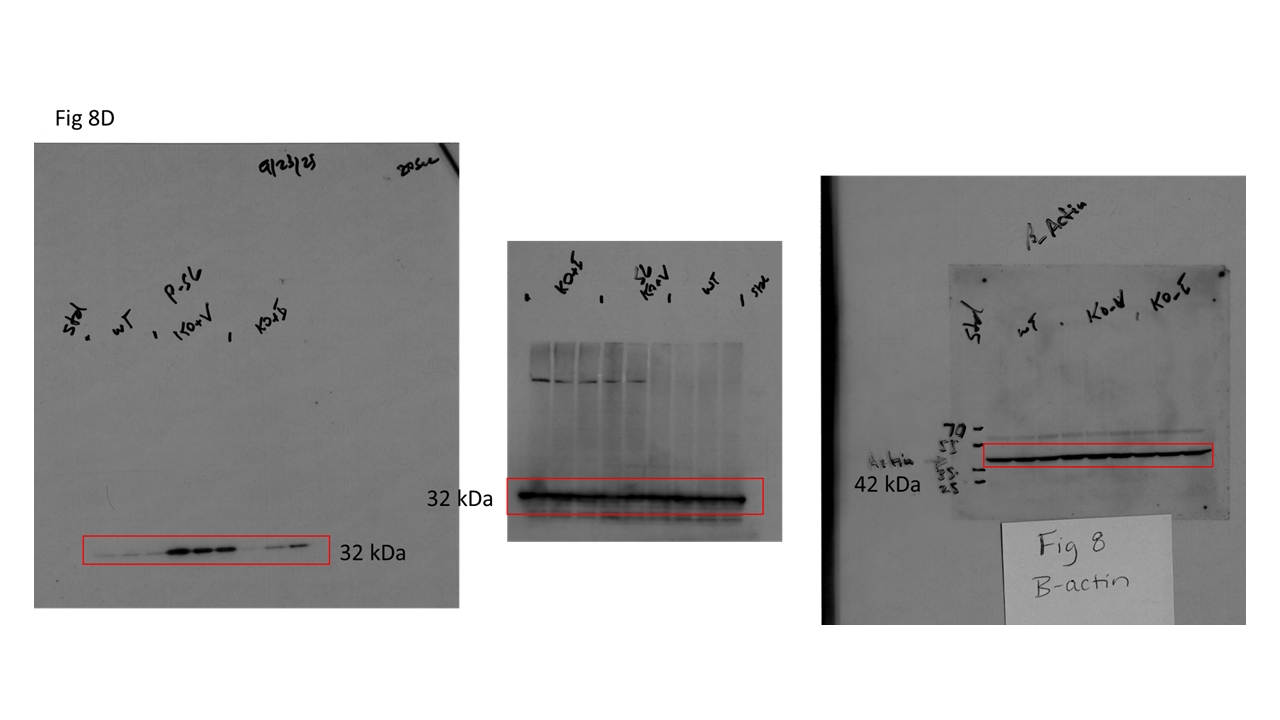

Supplement: Supplementary file 16 — Source data Fig. 8 [file 44321_2025_360_MOESM16_ESM.zip › EMM-2025-22130_SourceDataForFigure 8A-E 10-28-25/8D/Western Blot Source Data 10-28-25.tif]

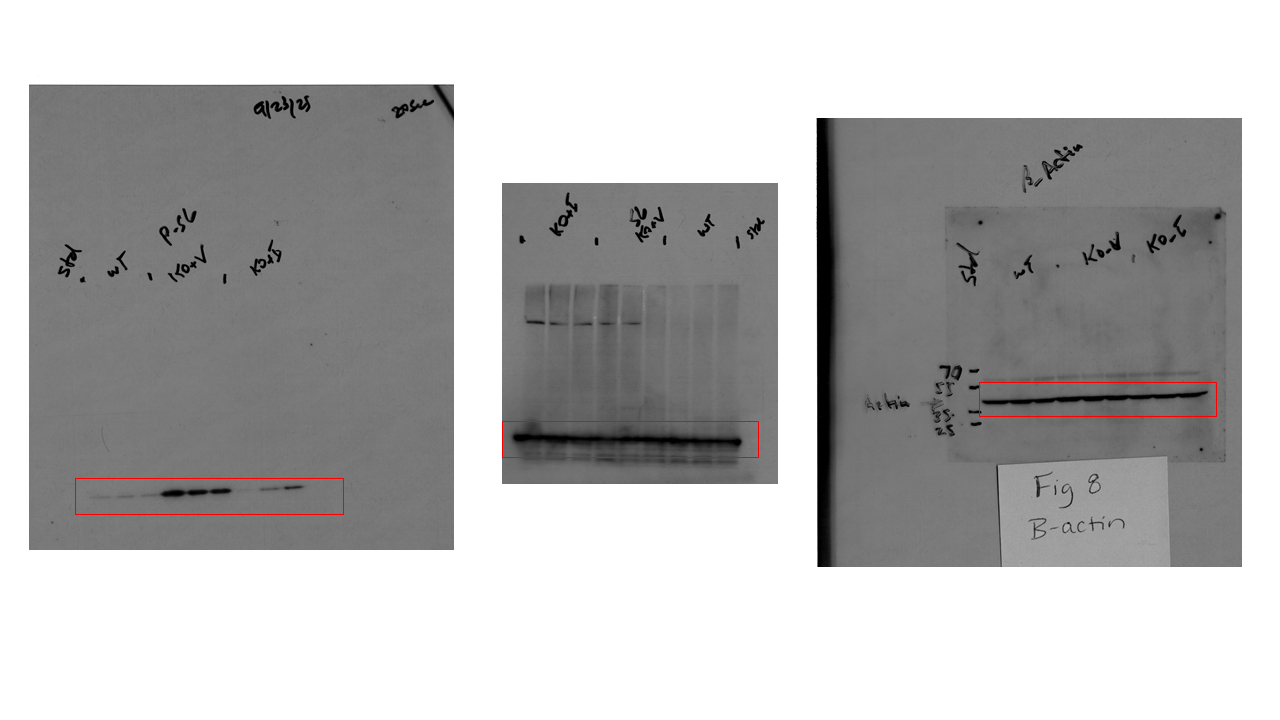

Supplement: Supplementary file 16 — Source data Fig. 8 [file 44321_2025_360_MOESM16_ESM.zip › EMM-2025-22130_SourceDataForFigure 8A-E 10-28-25/8D/Western Source Data.tif]

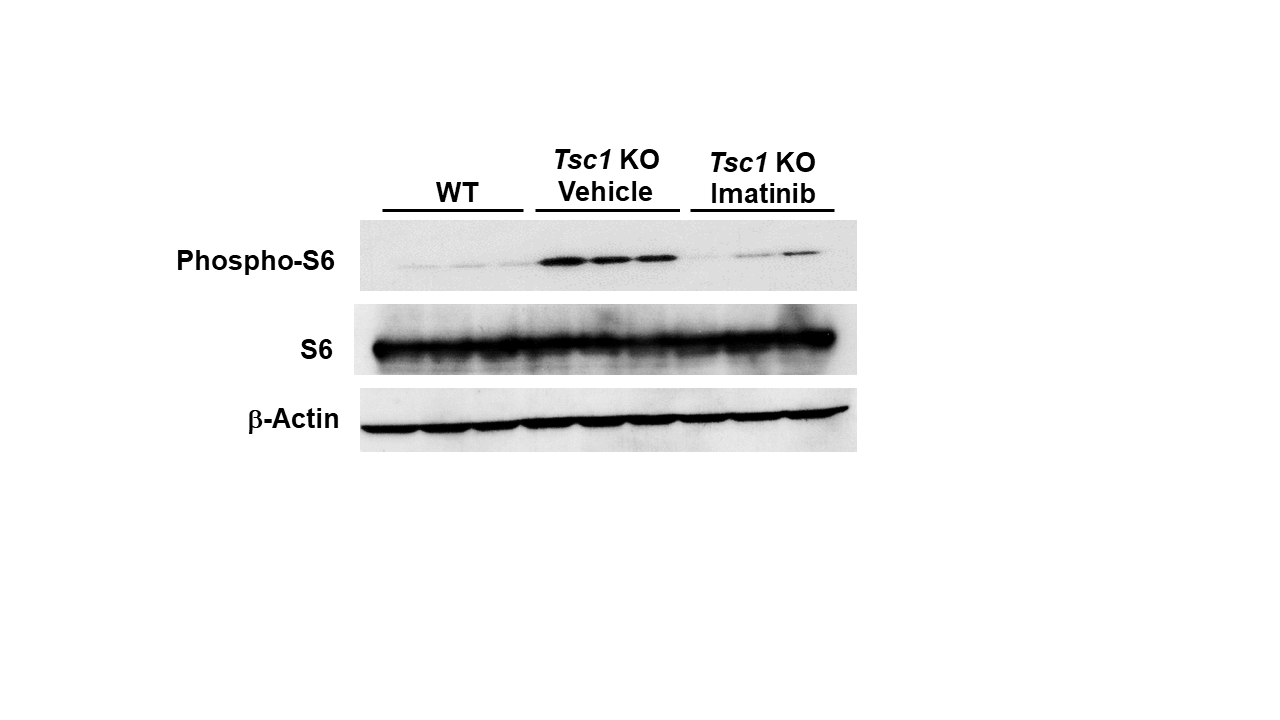

Supplement: Supplementary file 16 — Source data Fig. 8 [file 44321_2025_360_MOESM16_ESM.zip › EMM-2025-22130_SourceDataForFigure 8A-E 10-28-25/8D/Western.tif]

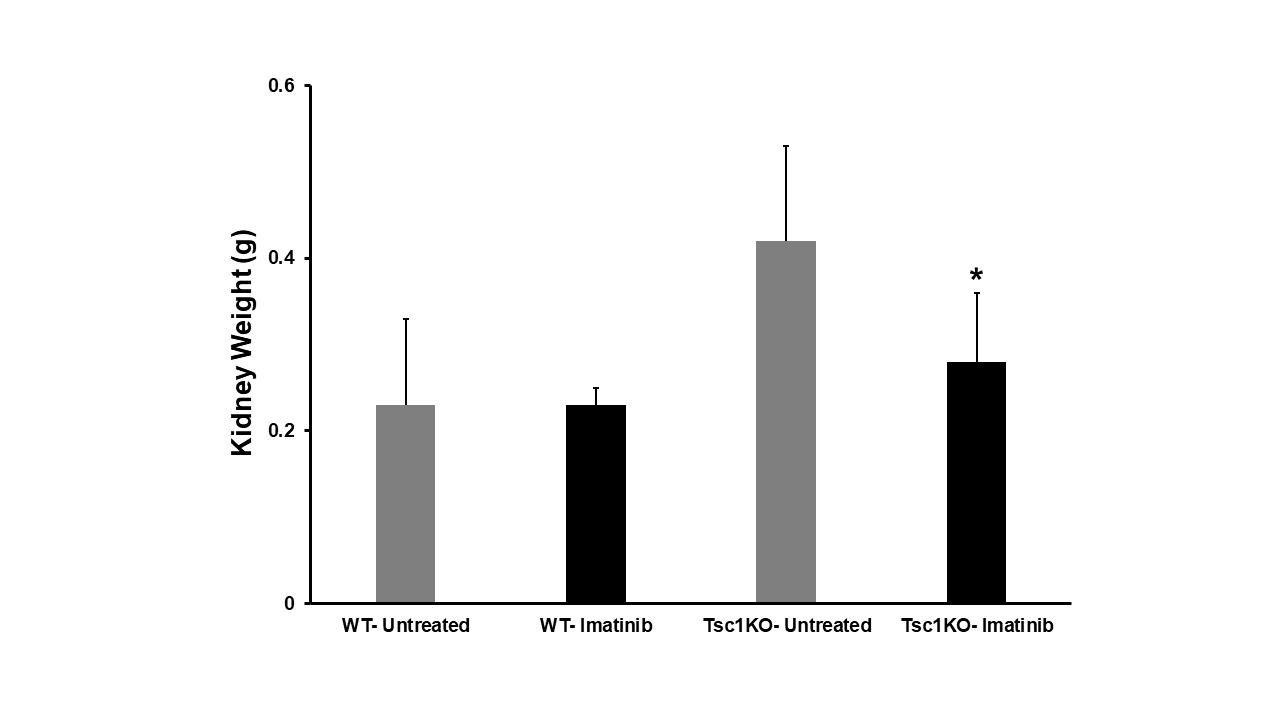

Supplement: Supplementary file 16 — Source data Fig. 8 [file 44321_2025_360_MOESM16_ESM.zip › EMM-2025-22130_SourceDataForFigure 8A-E 10-28-25/8E/Numerical Graph.tif]

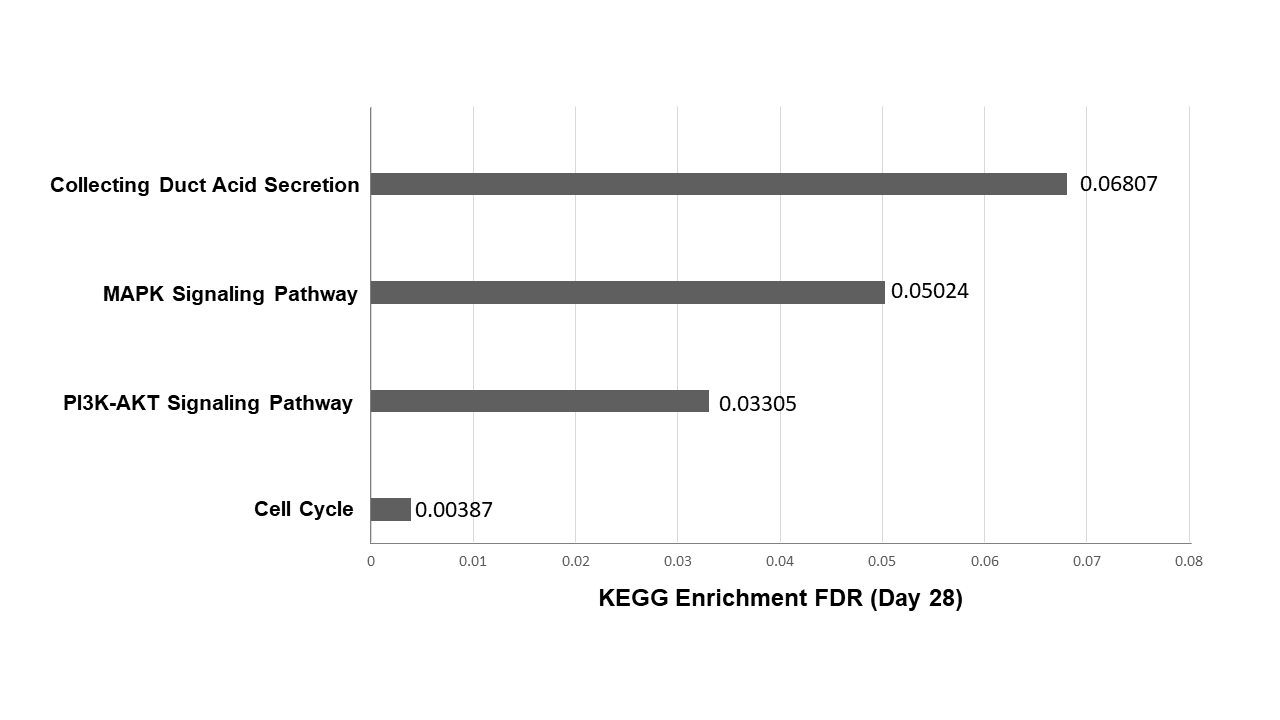

Supplement: Supplementary file 17 — Figure EV1 Source Data [file 44321_2025_360_MOESM17_ESM.zip › EMM-2025-22130_SourceDataForFigure EV1 10-28-25/1A/KEGG Enrichment.tif]

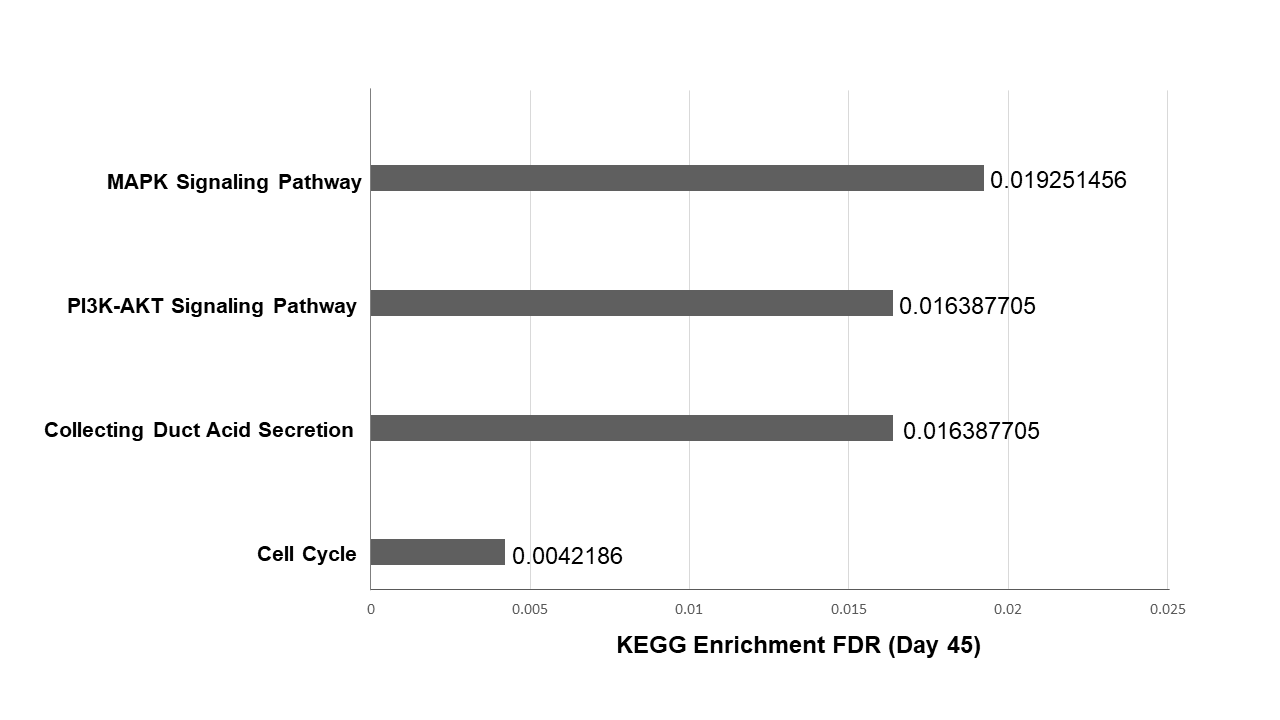

Supplement: Supplementary file 17 — Figure EV1 Source Data [file 44321_2025_360_MOESM17_ESM.zip › EMM-2025-22130_SourceDataForFigure EV1 10-28-25/1B/KEGG Enrichment.tif]

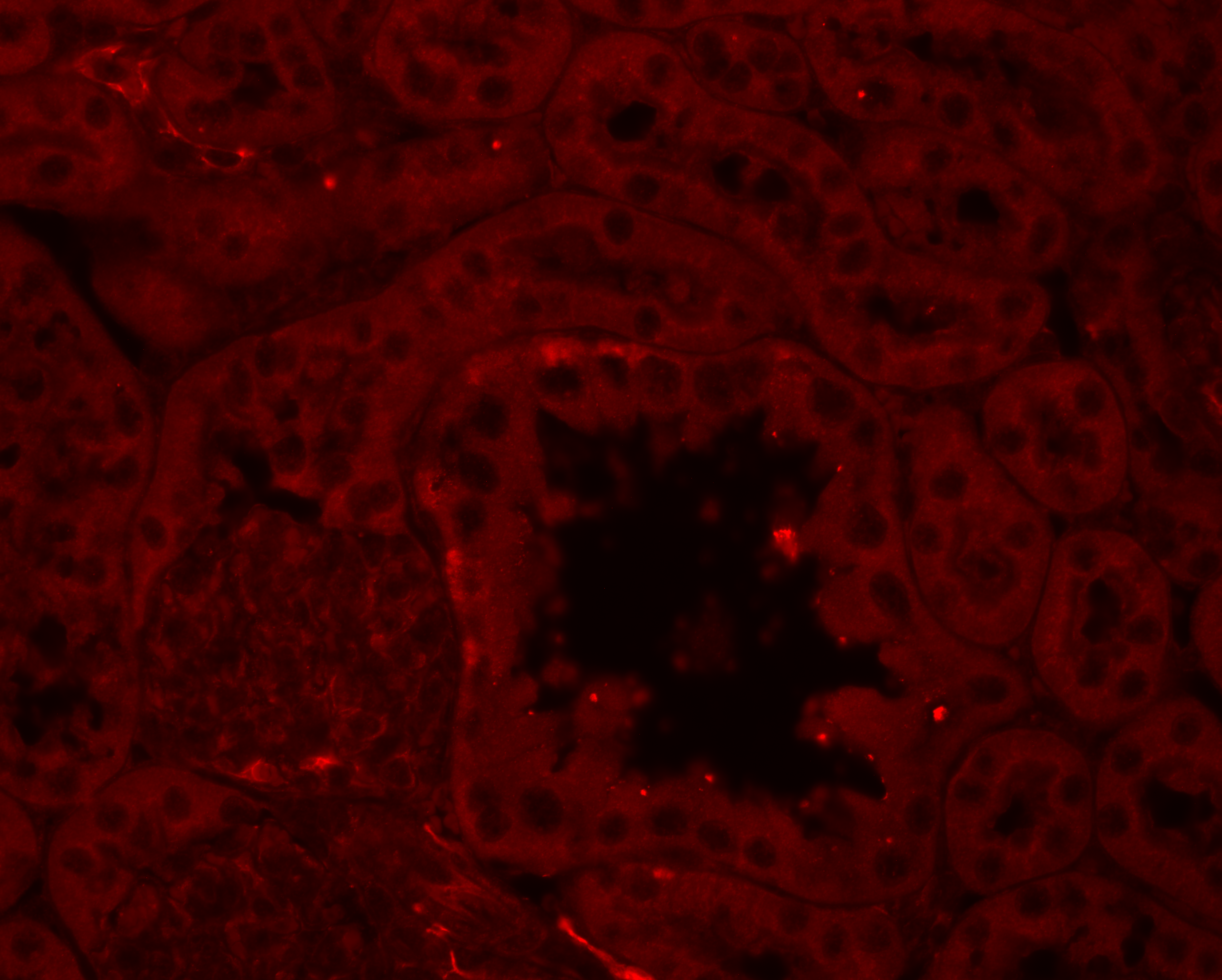

Supplement: Supplementary file 18 — Figure EV2 Source Data [file 44321_2025_360_MOESM18_ESM.zip › EMM-2025-22130_SourceDataForFigure EV2 10-28-25/Tsc1 ECE Aqp2 40X.tif]

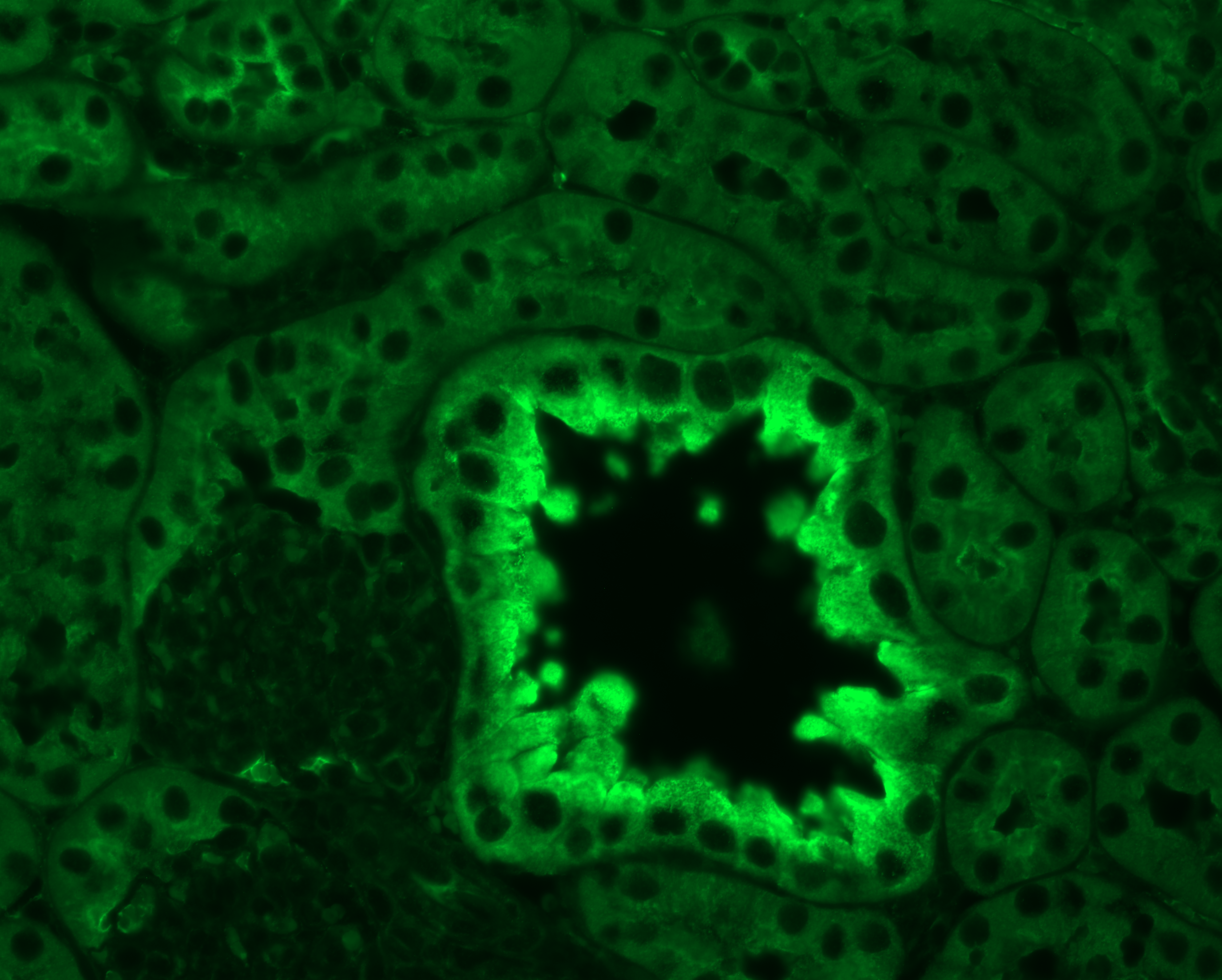

Supplement: Supplementary file 18 — Figure EV2 Source Data [file 44321_2025_360_MOESM18_ESM.zip › EMM-2025-22130_SourceDataForFigure EV2 10-28-25/Tsc1 ECE HATPase 40X.tif]

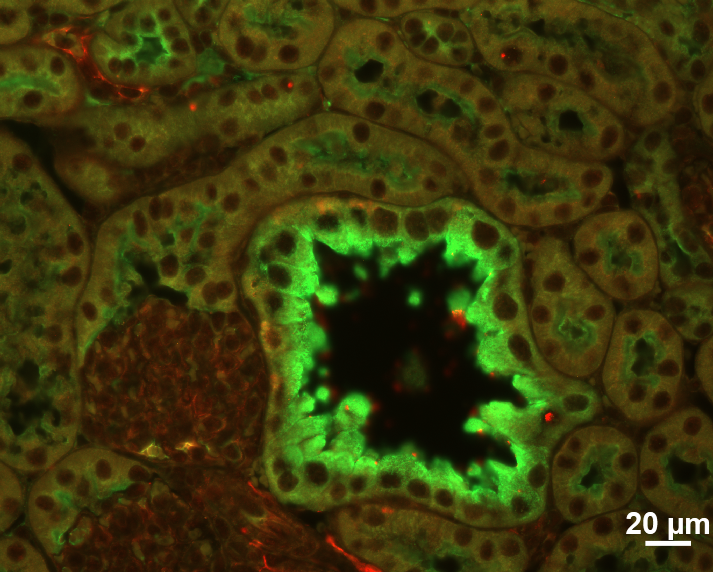

Supplement: Supplementary file 18 — Figure EV2 Source Data [file 44321_2025_360_MOESM18_ESM.zip › EMM-2025-22130_SourceDataForFigure EV2 10-28-25/Tsc1 ECE Merged Image 40X.tif]

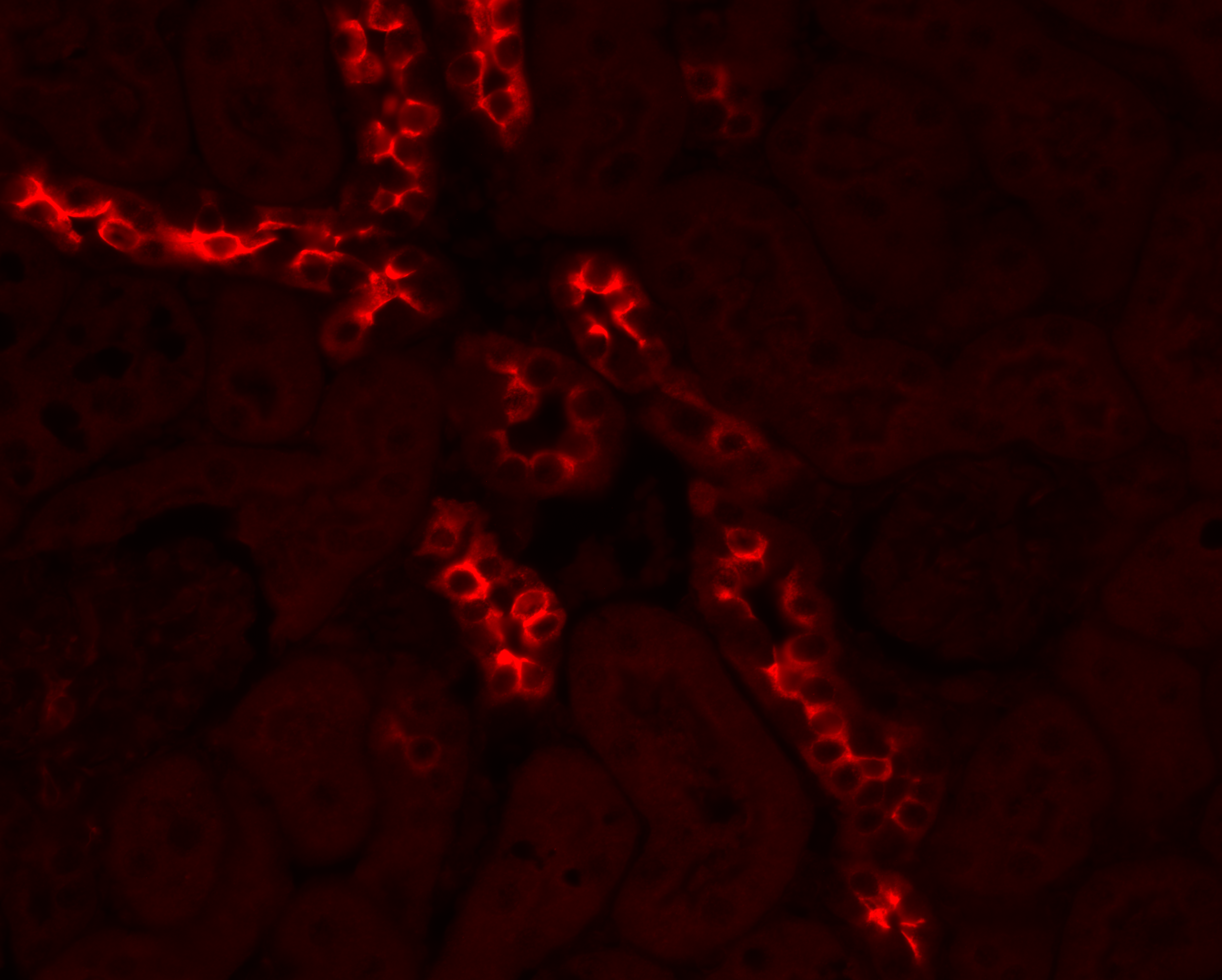

Supplement: Supplementary file 18 — Figure EV2 Source Data [file 44321_2025_360_MOESM18_ESM.zip › EMM-2025-22130_SourceDataForFigure EV2 10-28-25/WT Aqp2 40X.tif]

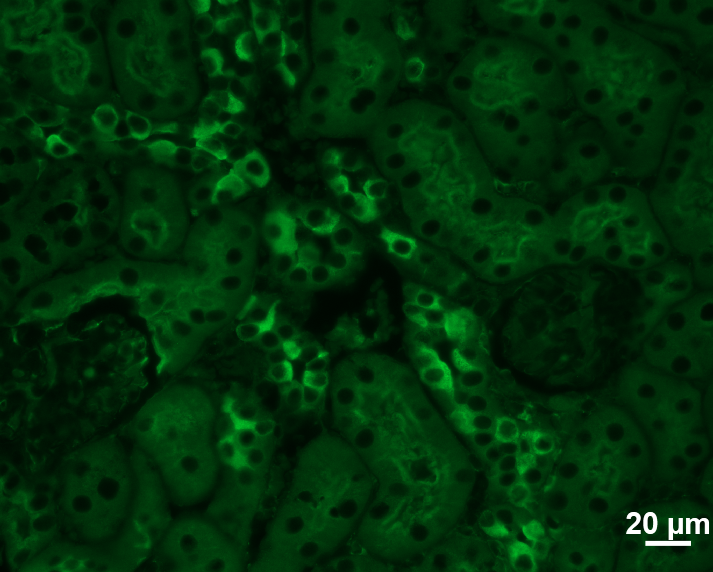

Supplement: Supplementary file 18 — Figure EV2 Source Data [file 44321_2025_360_MOESM18_ESM.zip › EMM-2025-22130_SourceDataForFigure EV2 10-28-25/WT HATPase 40X.tif]

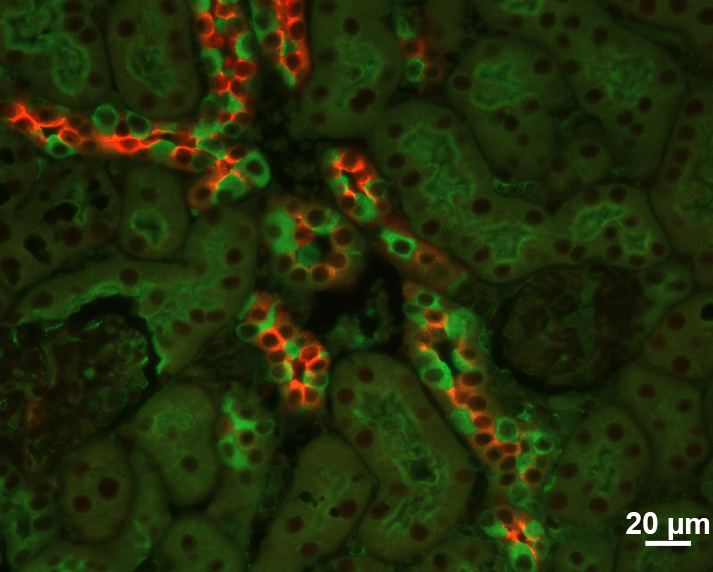

Supplement: Supplementary file 18 — Figure EV2 Source Data [file 44321_2025_360_MOESM18_ESM.zip › EMM-2025-22130_SourceDataForFigure EV2 10-28-25/WT Merged Image 40X.tif]

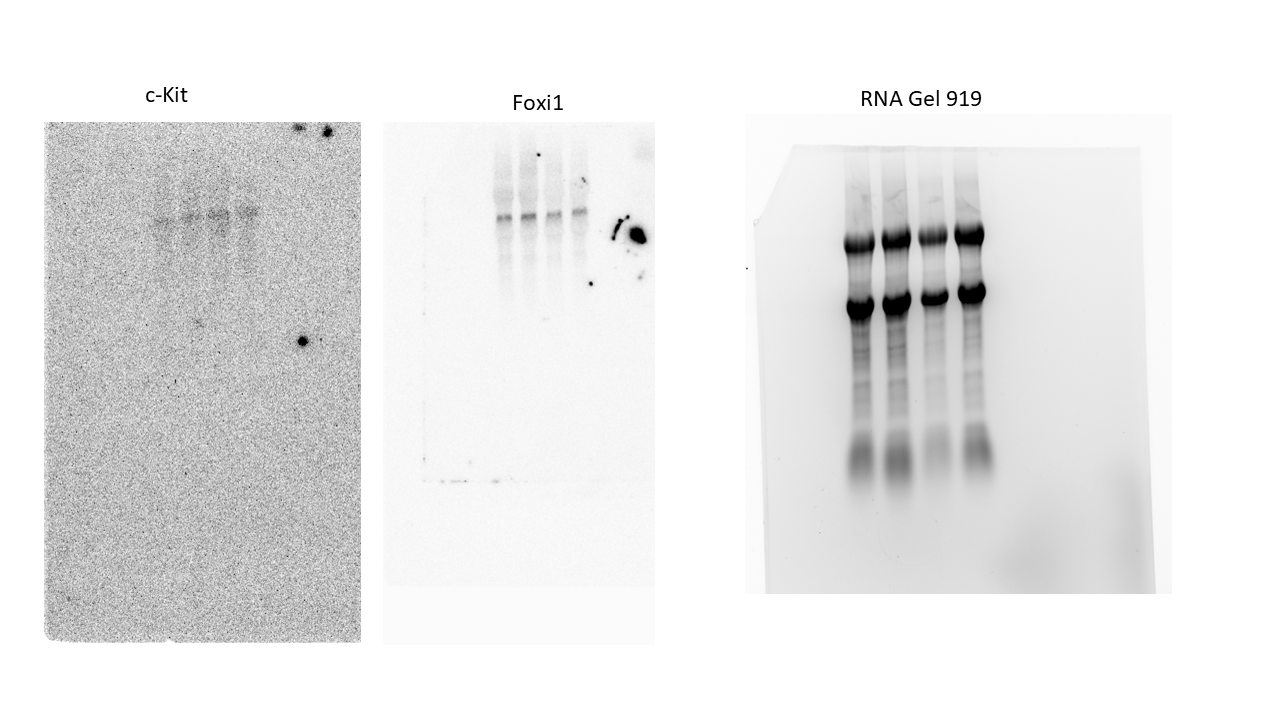

Supplement: Supplementary file 19 — Figure EV3 Source Data [file 44321_2025_360_MOESM19_ESM.zip › EMM-2025-22130_SourceDataForFigure EV3 10-28-25/Northern Source Data.tif]

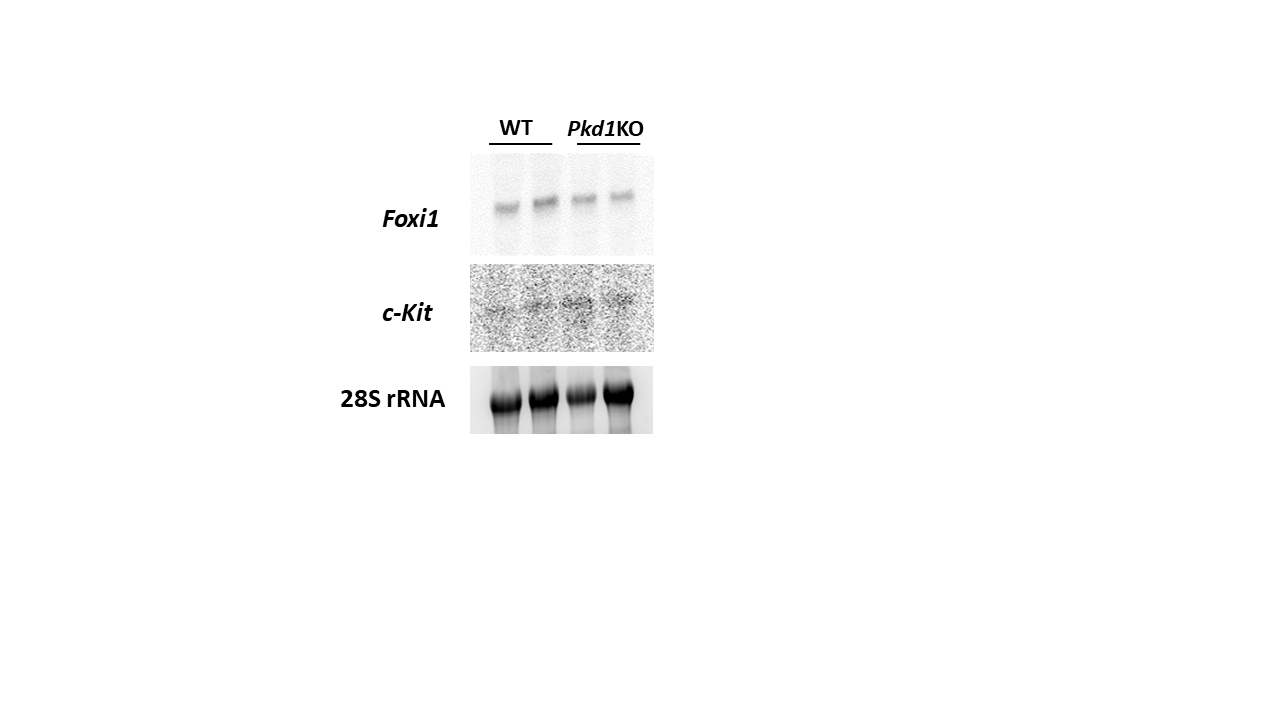

Supplement: Supplementary file 19 — Figure EV3 Source Data [file 44321_2025_360_MOESM19_ESM.zip › EMM-2025-22130_SourceDataForFigure EV3 10-28-25/Northern.tif]

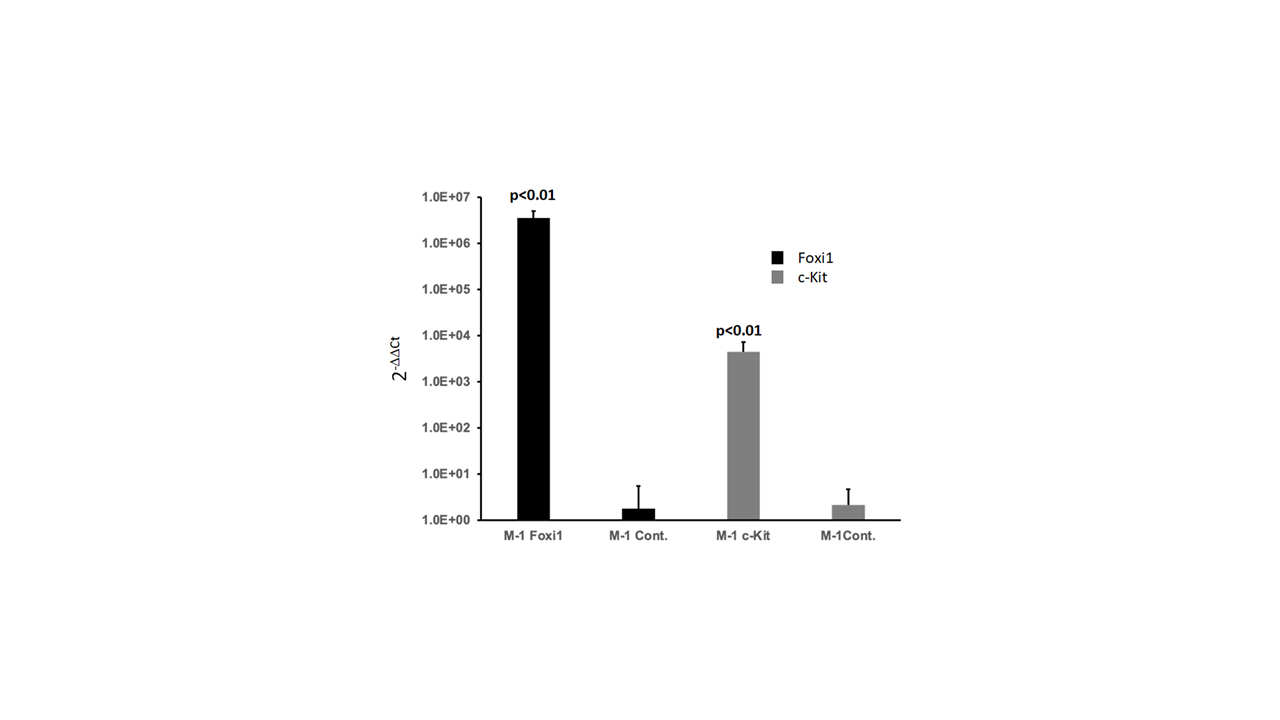

Supplement: Supplementary file 20 — Figure EV4 Source Data [file 44321_2025_360_MOESM20_ESM.zip › EMM-2025-22130_SourceDataForFigure EV4 10-28-25/Numerical Graph.tif]

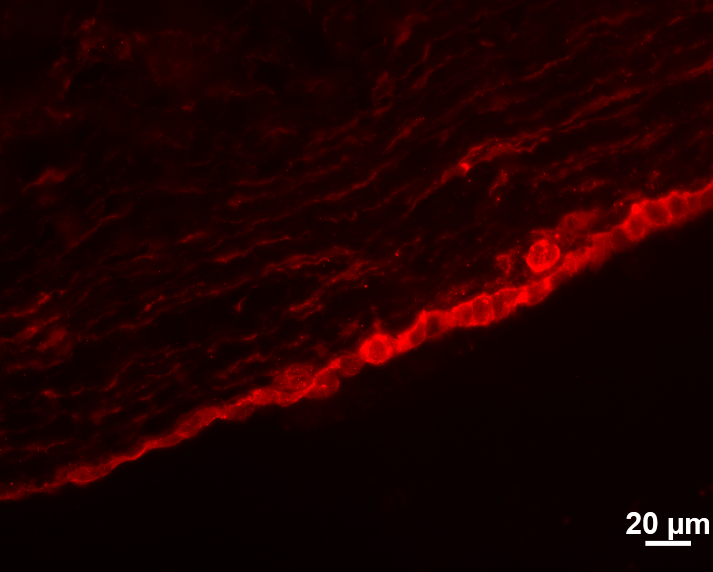

Supplement: Supplementary file 21 — Figure EV5 Source Data [file 44321_2025_360_MOESM21_ESM.zip › EMM-2025-22130_SourceDataForFigure EV5 10-28-25/c-KIT 40X.tif]

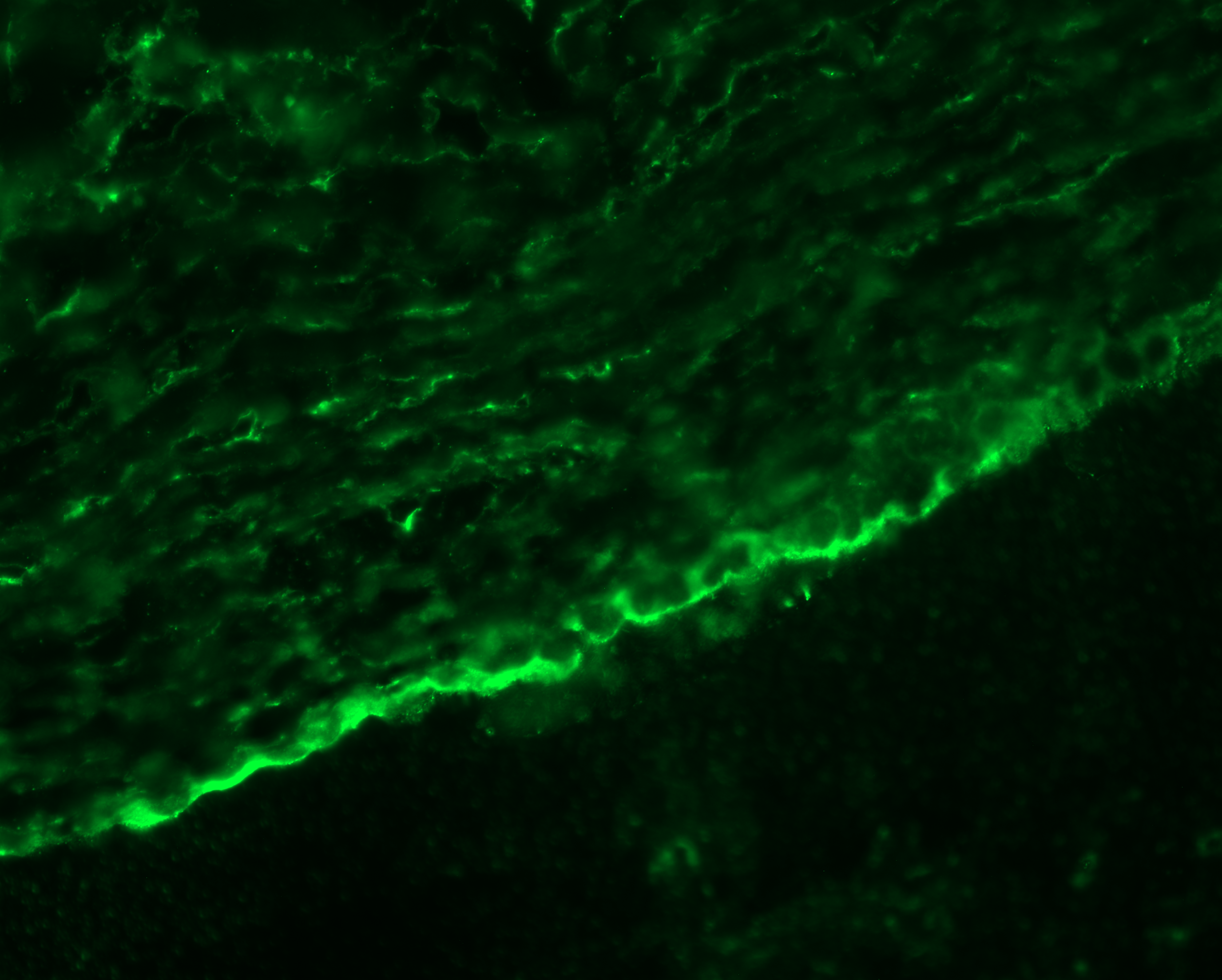

Supplement: Supplementary file 21 — Figure EV5 Source Data [file 44321_2025_360_MOESM21_ESM.zip › EMM-2025-22130_SourceDataForFigure EV5 10-28-25/HATPase 40X.tif]

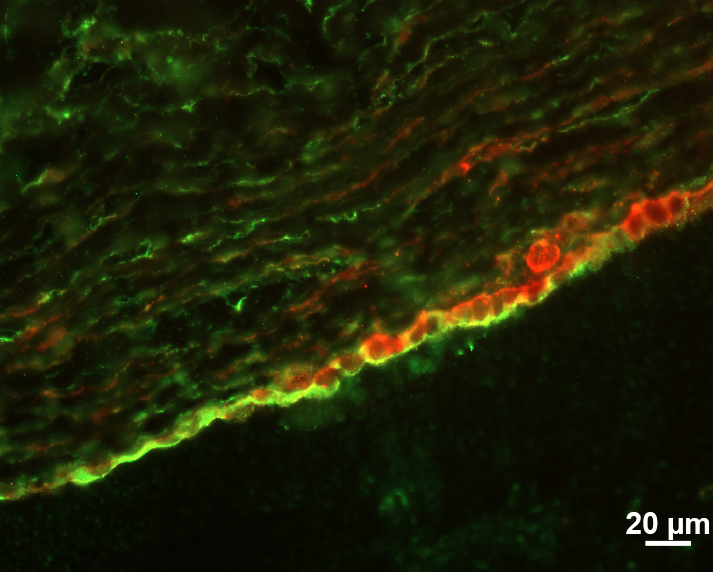

Supplement: Supplementary file 21 — Figure EV5 Source Data [file 44321_2025_360_MOESM21_ESM.zip › EMM-2025-22130_SourceDataForFigure EV5 10-28-25/Merged Image 40X.tif]

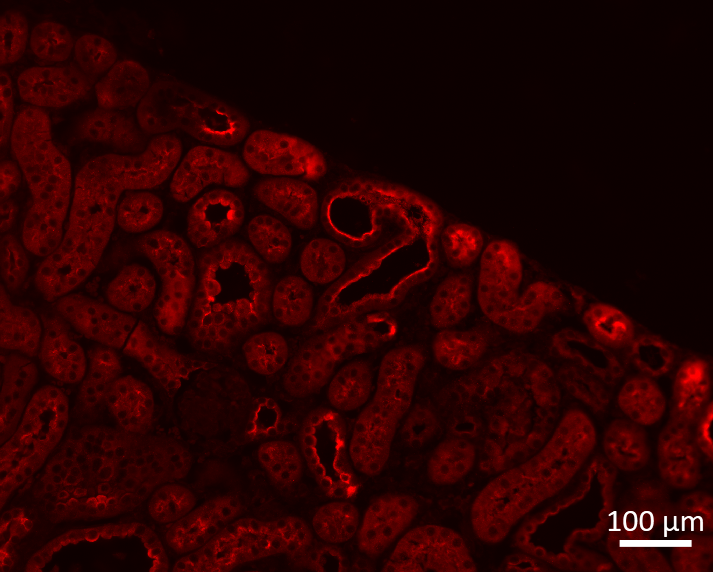

Supplement: Supplementary file 22 — Figure EV6 Source Data [file 44321_2025_360_MOESM22_ESM.zip › EMM-2025-22130_SourceDataForFigure EV6 10-28-25/HATPase 20X.tif]

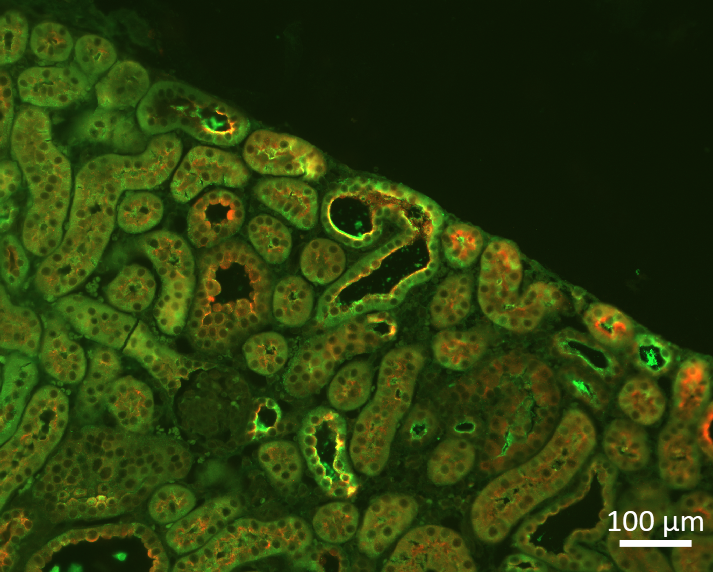

Supplement: Supplementary file 22 — Figure EV6 Source Data [file 44321_2025_360_MOESM22_ESM.zip › EMM-2025-22130_SourceDataForFigure EV6 10-28-25/Merged Image 20X.tif]

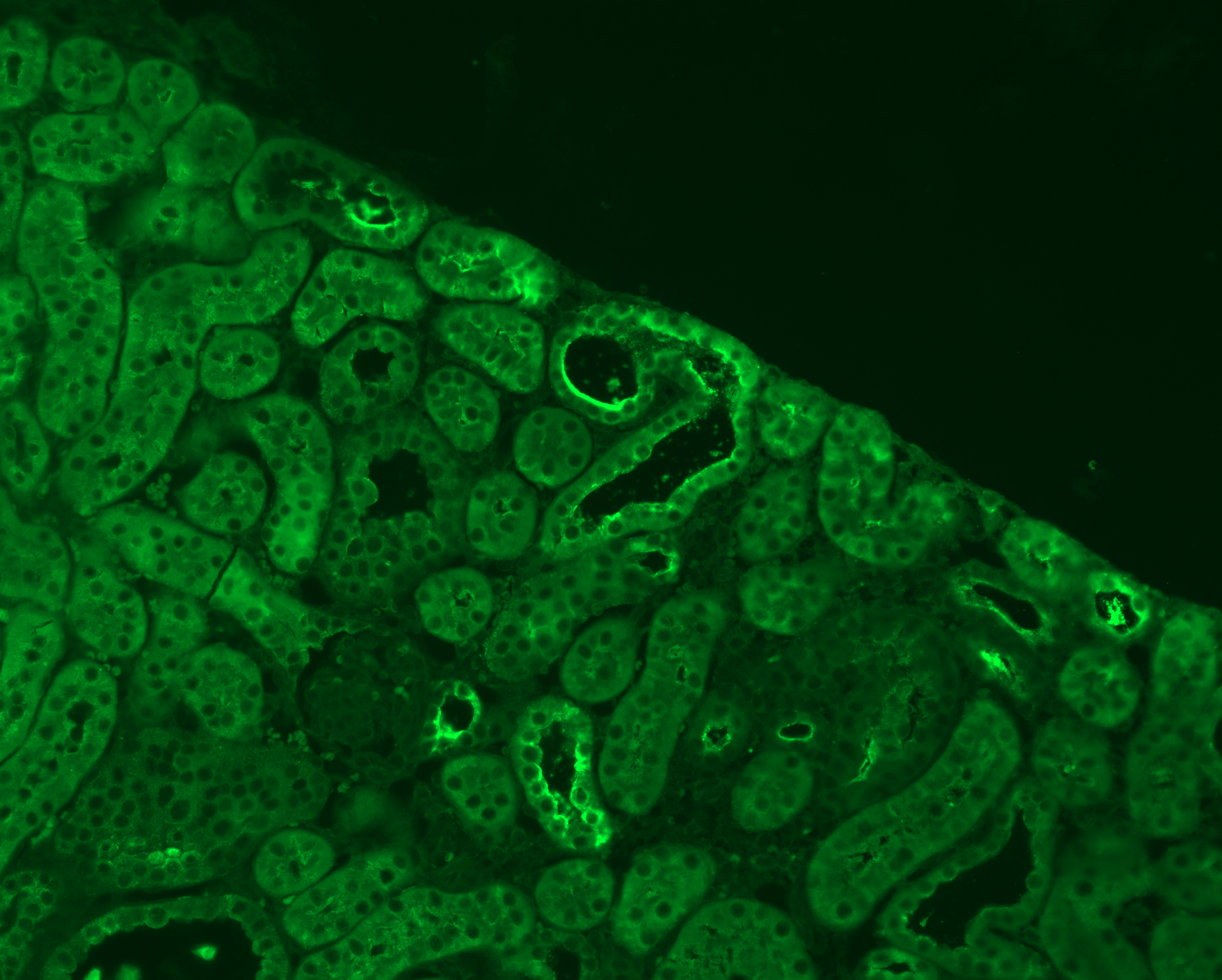

Supplement: Supplementary file 22 — Figure EV6 Source Data [file 44321_2025_360_MOESM22_ESM.zip › EMM-2025-22130_SourceDataForFigure EV6 10-28-25/pTSC2 1798 20X.tif]
