# Supplementary material for: The critical role of the proto-oncogene c-Kit in TSC renal cystogenesis
Source: EMBO Mol Med. 2025 Dec 22;18(2):575–98. doi: 10.1038/s44321-025-00360-x (PMC12905254; doi:10.1038/s44321-025-00360-x)
Supplement: Supplementary file 23 — Expanded View Figures [file 44321_2025_360_MOESM23_ESM.pdf]

Expanded View Figures

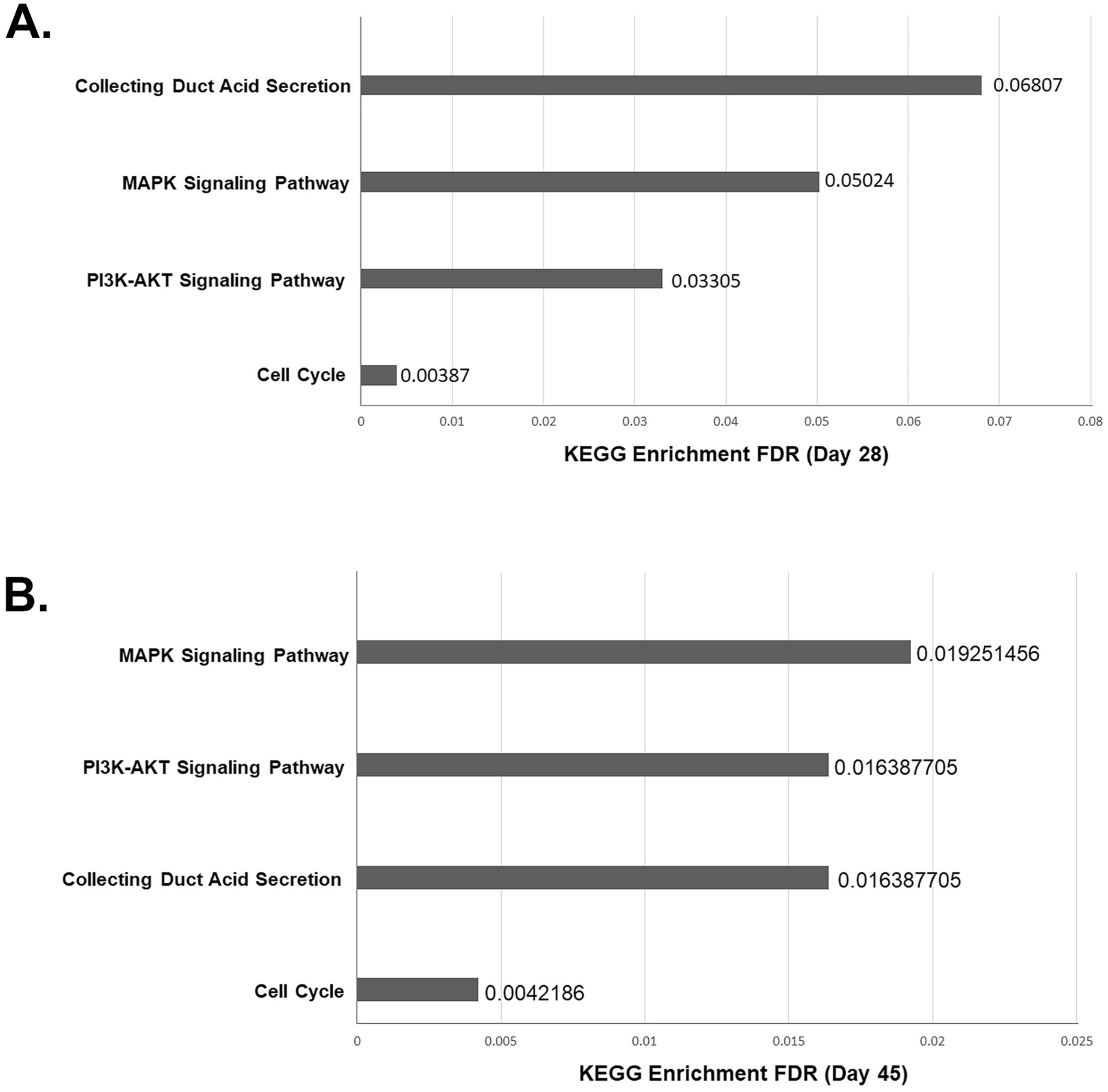

Figure EV1. KEGG enrichment analysis.

KEGG enrichment analysis was performed on (**A**) 28-day *Tsc1-KO* mice and (**B**) 45-day *Tsc1-KO* mice using DEG with fold induction of greater than 1.3 and FDR < 0.05. The results indicate that collecting duct acid secretion, MAPK, PI3K-AKT, and cell cycle pathways were significantly enriched. Source data are available online for this figure.

© The Author(s)

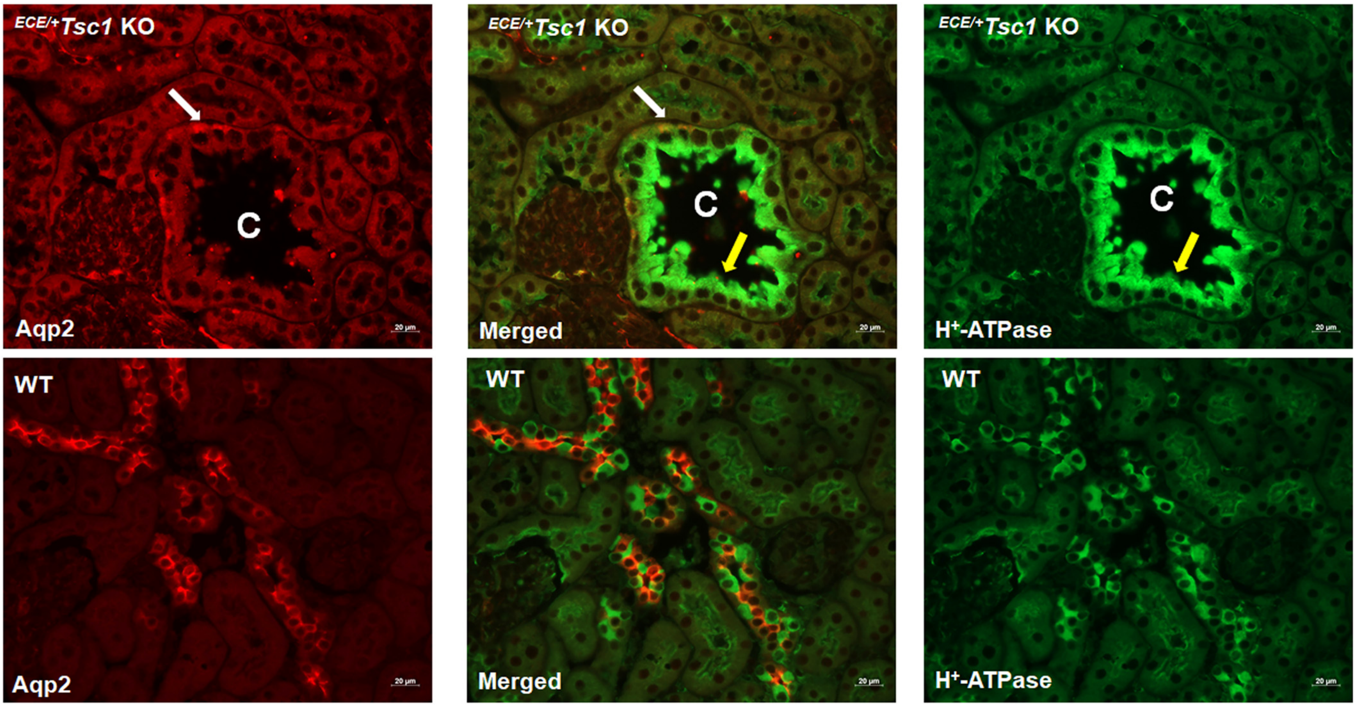

Figure EV2. Localization of AQP2 and H^{+}-ATPase in $^{ECE+/-}$*Tsc1*KO.

Double immunofluorescence images of *Tsc1-KO* (upper row) and WT (lower row) mice stained with anti-AQP2 (red; left upper and lower panels) and H^{+}-ATPase (green; right upper and lower panels) antibodies. A merged image illustrating co-localization of both antibodies is presented in the middle panel. White arrows point to basolateral AQP2 expression, while yellow arrows indicate apical H^{+}-ATPase. "C" represents cysts. Scale bar equals 20 µm. Source data are available online for this figure.

 © The Author(s)

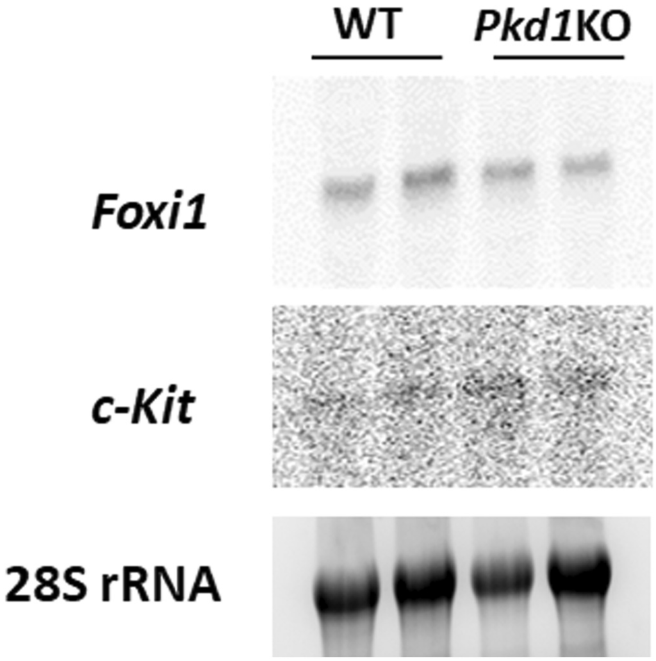

Figure EV3. Comparison of expression of *Foxi1* and *c-Kit* in *Pkd1-KO* mice.

Northern blot comparing *Foxi1* and *c-Kit* expression in *Pkd1-KO* vs. WT mice. Unlike *Tsc1-KO* mice, there is little to no expression of either of the two genes. Source data are available online for this figure.

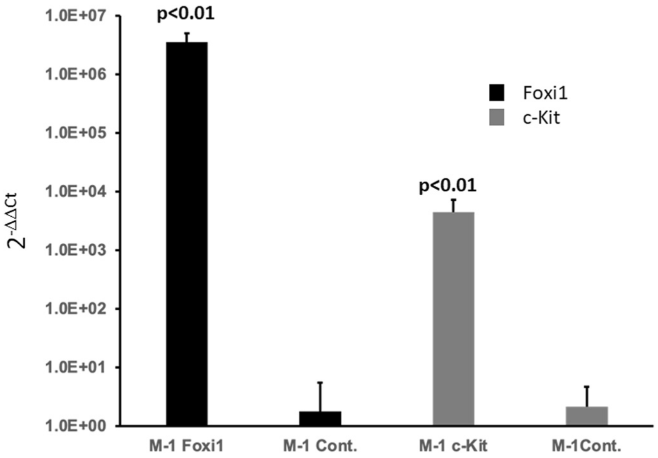

Figure EV4. Examination of *Foxi1* and *c-Kit* expression in transfected M-1 cells.

M-1 collecting duct cell lines were stably transfected with *Foxi1*, *c-Kit* and control (empty) expression vectors. The expression of *Foxi1*(black) and *c-Kit* (gray) were compared in clonally purified stable transfectants. The studies represent values obtained from 5 clones for each transfected cell line ($n = 2$ replicas/clonal isolates). The "*p*" values were determined using two-tailed student t-test. Source data are available online for this figure.

 © The Author(s)

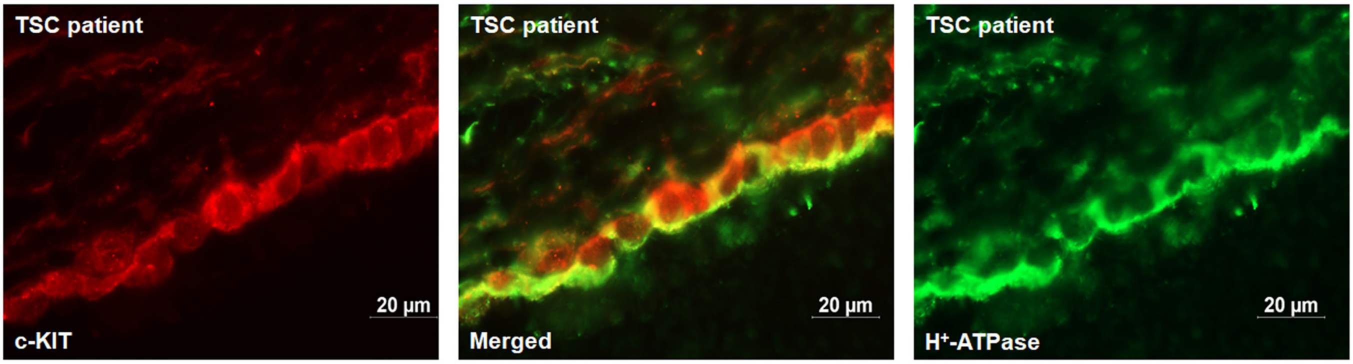

Figure EV5. c-KIT and H^+-ATPase expression in individuals with TSC.

Similar to our TSC mouse models, individuals with TSC also have a basolateral localization of c-KIT (red; left panel) and apical distribution of H^+-ATPase (green; right panel) in their cystic epithelium. Scale bar equals 20 µm. Source data are available online for this figure.

© The Author(s)

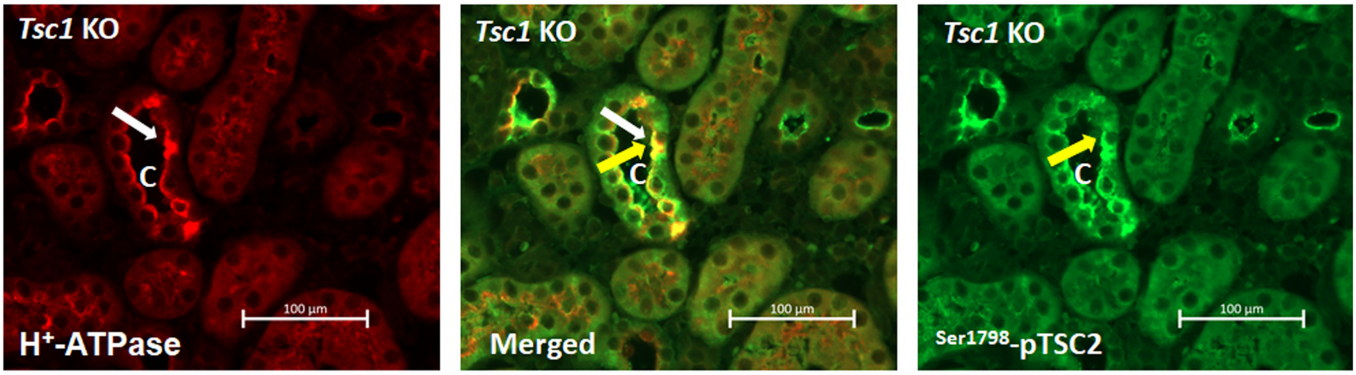

Figure EV6. Localization of H^+-ATPase and ^{Ser1798}-pTsc2 in *Tsc1-KO* mice.

Double immunofluorescence images of apical H^+-ATPase (red; left panel) and apical ^{Ser1798}-pTsc2 (green; right panel) in *Tsc1-KO* mice. A merged image illustrating co-localization of H^+-ATPase and ^{Ser1798}-pTSC2 is presented in the middle panel. White arrows point to apical H^+-ATPase expression, while yellow arrows indicate apical and subapical ^{Ser1798}-pTSC2 localization. "C" represents cysts. Scale bar equals 100 µm. Source data are available online for this figure.

© The Author(s)
